# Avian responses to climate extremes: insights into abundance curves and species sensitivity using the UK Breeding Bird Survey

**DOI:** 10.1007/s00442-023-05504-9

**Published:** 2024-01-20

**Authors:** Pietro Tirozzi, Dario Massimino, Luciano Bani

**Affiliations:** 1 Department of Earth and Environmental Sciences, University of Milano-Bicocca, Piazza Della Scienza 1, 20126 Milan, Italy; 2 National Biodiversity Future Center, NBFC, 90133 Palermo, Italy; 3 British Trust for Ornithology, BTO, The Nunnery, Thetford, Norfolk IP24 2PU UK

**Keywords:** Birds, Climate change, Extreme events, Temperature, Rainfall

## Abstract

Climate change remains one of the most urgent challenges for biodiversity conservation. Recent studies have highlighted that climate extremes (CLEXs) can lead to widespread and negative effects across all taxa and ecological levels, but most of these studies are based on short-term periods and small spatial scales and lack a multi-species approach. Here, using generalised additive models (GAMs) and the UK Breeding Bird Survey (BBS), we described response curves for the abundance of 100 resident bird species over large spatial and temporal scales and identified the species showing a greater sensitivity to CLEXs. We used five climatic indices computed at 1-km spatial resolution as proxies of CLEXs during the winter or breeding season and considered both 1- and 2-year lagged effects. The results demonstrated widespread and significant effects of CLEXs on bird abundances at both time lags and in both seasons. Winter frost days (FD0), summer days (SU25) during the breeding season and simple precipitation intensity index (SDII) during the breeding season mainly showed negative effects. Daily temperature range (DTR) in both winter and breeding season and dry days (DD) during the breeding season led to diversified responses across the species, with a prevalence of positive effects. A large proportion of species showed a high sensitivity to CLEXs, highlighting that these species may deserve attention in future studies aimed at biodiversity conservation. We demonstrated that CLEXs can represent a significant driver affecting population abundances over large spatial and temporal scales, emphasising the need for understanding mechanistic processes at the basis of the observed effects.

**Supplementary Information:**

The online version contains supplementary material available at 10.1007/s00442-023-05504-9.

## Introduction

Climate change has been recognised as one of the major threats for biodiversity and species conservation (Urban 2015; Wiens 2016). Biological responses can include several related outcomes (Maxwell et al. 2019), such as shift in distribution (Chen et al. 2011), changes in population size (Cruz-McDonnell and Wolf 2016; Stephens et al. 2016), modifications in the Grinnellian niche (Tirozzi et al. 2022a) and variations in phenology (Thackeray et al. 2016), behaviour (Saino et al. 2011) and fitness (Sanz et al. 2003). Most research effort had been focused on ecological and biological effects of long-term changes in climatic means, but in the last two decades, climate extremes (CLEXs) (e.g. extreme temperatures, heavy rainfall, prolonged drought; hereafter CLEXs) have attracted increasing concern among ecologists (Bailey and van de Pol 2016). Indeed, some studies pointed out that CLEXs can lead to greater biological consequences compared to changes in climatic means (Maron et al. 2015; Bailey and van de Pol 2016; Marcelino et al. 2020), with widespread negative effects across all taxa and ecological levels (Maxwell et al. 2019). CLEXs are expected to increase in frequency in the future (IPCC 2013), stressing the importance of assessing their impact on ecological systems and biodiversity. However, defining CLEXs and evaluating their effects on biological systems are still a challenge for ecologists (Bailey and van de Pol 2016). Climatic indices have been successfully used as a measure of CLEXs to assess biological responses in wild populations (Morrison et al. 2016; Cady et al. 2019). Some of these indices are the result of the work of joint scientific committees, such as the former Expert Team on Climate Change and Detection and Indices (https://www.wcrp-climate.org/etccdi), or the European Climate Assessment & Dataset project (https://www.ecad.eu). Despite the increasing research effort on measuring the consequences of CLEXs on species and biodiversity, there are still gaps: (i) assessing lagged effects over time, (ii) carrying out studies on multiple species and (iii) using biological datasets covering large spatial and temporal scales. In relation to the first issue, species might display a temporal delay in biological responses to climate change (Saunders et al. 2021), and overlooking such delays could mask the real effect size of CLEXs, potentially leading to an underestimation of their effects. Second, investigating multi-species responses can contribute to a more exhaustive perspective on how CLEXs act across species and on communities (Palmer et al. 2017) and identify the main climatic drivers affecting multiple species, thus providing information and guidance for effective conservation policies. Third, as generalisation of results beyond time- and space-specific contexts is often risky and needs to be evaluated carefully, using large datasets collected over wide spatial and temporal scale (which are typical of long-term national monitoring programmes) helps reduce stochastic noise and the dependency of results on the specific circumstances characterising datasets collected at small spatio-temporal scales.

To address these gaps, birds represent a suitable model taxon for investigating the effects of CLEXs (Cohen et al. 2020), other than to be useful for planning environmental policies aimed at the conservation of biodiversity (Virkkala et al. 2022). Birds are sensitive to climate change (Pautasso 2012) and could be affected by CLEXs in several ways. CLEXs can alter the viability of local populations (McKechnie et al. 2021), provoke phenotypic selection (Acker et al. 2021), influence reproductive success (Cruz-McDonnell and Wolf 2016; Colón et al. 2017), survival rates (Robinson et al. 2007) or population growth (Morrison et al. 2016) and affect species’ distribution (Cohen et al. 2020). Furthermore, the existence of large-scale long-term data for birds represents an important source of structured data to take the aforementioned gaps into account.

Using the UK Breeding Bird Survey (BBS, Harris et al. 2022), the national long-term monitoring programme of breeding birds in the United Kingdom (western Europe, 59°–50°N, 8°O–2°E), we performed an analysis on multiple species aimed at: (i) describing response curves of relative abundance of bird populations to several types of CLEXs (at both 1- and 2-year time lag) over large geographic extents and temporal scales, (ii) assessing whether responses to CLEXs are similar across species and (iii) identifying species showing a greater sensitivity to the effects of CLEXs.

## Materials and methods

### Bird data

**Fig. 1:**
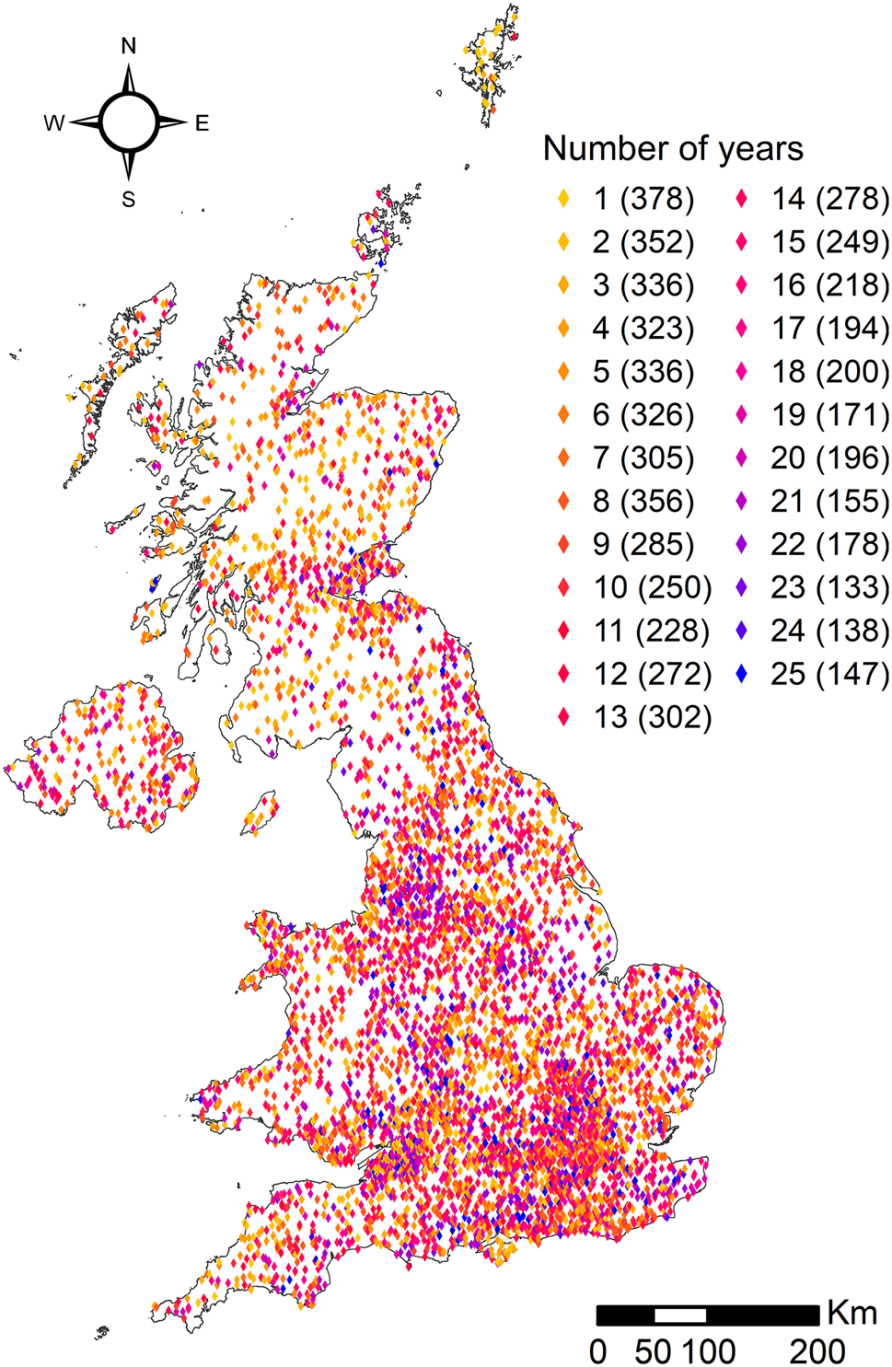
Spatial distribution of the UK BBS squares surveyed between 1994 and 2019 that have been used in this study. The number of years each square was surveyed during the whole period (number of years) is represented by a colour gradient from yellow to blue. The number of squares belonging to each class of frequency is reported in parentheses

Bird data were derived from the UK BBS, which employs a stratified random sampling protocol where 1 km^2^ (fixed sampling units) is surveyed following a line-transect method (Gregory et al. 2004) along two 1-km transects. Squares are visited twice per year, once in the early breeding season (April to early-May) and again in the late breading season (late-May to June). For this study, we used the maximum of the two seasonal counts as a measure of relative abundance (Morrison et al. 2016). We used data collected from 1994 to 2019, excluding those obtained in 2001 when an outbreak of foot-and-mouth disease restricted access to many areas (Risely et al. 2013). Since the survey began in 1994, the number of squares annually surveyed that were used in this study (Fig. 1) has increased from 1550 to 3982 in 2019 (*n* = 69,163, annual mean = 2767, standard deviation = 741).

We restricted the analyses to species having full or partial resident populations in the UK (McInerny et al. 2018) with a frequency of occurrence ≥ 2.5% throughout the study area and the entire time series.

Resident birds, spending the whole life cycle (both winter and breeding season) in the same region, are supposed to be influenced by local environmental conditions throughout the full year, although limited movements may occur in local populations from winter to breeding season, and resident birds could be indirectly affected by climatic conditions in non-breeding areas. Data for the feral pigeon (*Columba livia*) only included domestic populations established in the wild, excluding the rock dove (*C. livia*) populations. Furthermore, there are some limitations in the BBS sampling method for nocturnal species included in the analyses (the tawny owl *Strix aluco* and the western barn owl *Tyto alba*), as well as the fact that counts for the great cormorant (*Phalacrocorax carbo*), the grey heron (*Ardea cinerea*) and the little egret (*Egretta garzetta*) may contain a proportion of individuals away from breeding sites. Finally, counts for the Eurasian oystercatcher (*Haematopus ostralegus*), the northern lapwing (*Vanellus vanellus*), the Eurasian curlew (*Numenius arquata*), the common snipe (*Gallinago gallinago*), the common redshank (*Tringa totanus*) and the European golden plover (*Pluvialis apricaria*) may include individuals from non-breeding flocks. We excluded gulls (genus *Larus* and the black-headed gull *Chroicocephalus ridibundus*) from the analyses because of the presence of an unknown number of non-breeding, migratory and off-duty individuals breeding at colonies many kilometres from the BBS squares during the time of survey and over the whole study area.

### Climate extremes (CLEXs)

As proxies of several types of CLEXs, we used five climatic indices, four of which belong to the suite of the core indices developed by the former Expert Team on Climate Change Detection and Indices (https://www.wcrp-climate.org/etccdi) (see Table 1 for definitions). Three indices (summer days: SU25; frost days: FD0; daily temperature range: DTR) are temperature based (T-based), while two of them (simple precipitation intensity index: SDII; dry days: DD) are rainfall based (R-based). We computed these indices over two distinct periods: winter season (1st December–28/29th February) and breeding season (1st April–31st July). Specifically, SU25, SDII and DD were calculated over the breeding season, while FD0 was calculated over the winter. DTR was computed separately over both the breeding and the winter seasons. Bird data in the year *t* were associated with the indices of the preceding winter or breeding season in the year *t − 1* and to those of the two previous year *t − 2* of the corresponding 1-km square to investigate potential lagged effects. Climatic indices were computed starting from daily maximum and minimum temperature (TX and TN, respectively) and daily precipitation (RR) at 1 × 1 km gridded resolution using climatic data compiled by MetOffice and available on CEDA archive as netCDF format (Hollis et al. 2021, downloadable at https://archive.ceda.ac.uk/). Indices were computed in R software (R Core Team 2022) using the package *raster* (Hijmans 2021), then spatially and temporally matched with bird data through ArcMap 10.7.1 (ESRI 2019). Between 1961 and 2018, DTR (in both seasons) and SU25 showed statistically significant increasing trends, SDII showed an increasing but non-statistically significant trend, DD a stable trajectory and FD0 a statistically decreasing trend. However, the assessment of trends for the indices during the period 1992–2018, i.e. years linked to bird data, showed stable trajectories for all of them (Supplementary Information Fig. S1a–f).

**Table 1:** Indices of climate extremes (CLEXs)

Index	Index name	Definition	Unit	Season	Source
FD0	Frost days	Count of days when TN < 0 °C	Day	W	https://www.climdex.org/
SU25	Summer days	Count of day when TX > 25 °C	Day	B	https://www.climdex.org/
DTR	Daily temperature range	Mean difference between daily TX and TN	°C	W; B	https://www.climdex.org/
SDII	Simple precipitation intensity index	Amount of precipitation (RR) on wet days (RR ≥ 1 mm)	mm/day	B	https://www.climdex.org/
DD	Dry days	Count of days when RR < 1 mm	Day	B	https://www.climdex.org/

*TN* daily minimum temperature, *TX* daily maximum temperature, *RR* daily amount of precipitation, *W* winter, *B* breeding. Each index was calculated in both *t* − *1* and *t* − *2*

### Modelling framework and statistical analyses

We fitted generalised additive models (GAMs, Hastie and Tibshirani 1986; Wood 2017) using the package *mgcv* (Wood 2021) in R (R Core Team 2022). GAMs allow detection of both linear and non-linear relationships between the response variable and predictors through a data-driven approach using splines (Wood 2017). We fitted models at single-species level separately (i.e. for each species, we ran separate models), using a shared framework. To control for potential confounding effects that can affect species abundance, for each square, we included the elevation at 1-km resolution (m), habitat cover (%; nine classes: woodland, scrubland, semi-natural grassland and marsh, heathland and bogs, farmland, human sites, waterbodies, coastal, inland rock) and a space–time smoother resulting from the interaction among northing, easting and year of survey that also accounted for potential spatial and temporal autocorrelation in the observed counts (Harrison et al. 2014; Oedekoven et al. 2017) (see also Supplementary Information Table S1 for the details on covariates). Habitat covers were recorded from surveyors in each year of the annual bird census by describing the main type of habitat for each 200-m transect section (Gregory and Bashford 1996); then, for each transect and each year of sampling, we calculated the percentage of habitat cover by dividing the number of times each type of cover occurred by the total number of sections of the transect. For each species, to account for overdispersion in count data (i.e. the variance is larger than the mean; Zuur et al. 2009), we fitted two models assuming either a Poisson or a negative binomial distribution for the count data and used the Akaike information criterion (AIC; Burnham and Anderson 2002) to select the best model (Tirozzi et al. 2022b).

For each species, the model can be expressed as:

$${\text{log}}\left(E\left[{Y}_{i,j,t}\right]\right)={f}_{s}\left({{{\text{northing}}}_{j},{{\text{easting}}}_{j},{\text{year}}}_{t}\right)+{f}_{s}\left({{\text{elev}}}_{j}\right)+{\sum }_{h=1}^{k}{f}_{s}\left({{\text{Hab}}}_{h,j,t}\right)+{\sum }_{m=1}^{n}{f}_{s}\left({C}_{m,j,t-1}\right)+{\sum }_{m=1}^{n}{f}_{s}\left({C}_{m,j,t-2}\right),$$

where log(*E[Y*_*i,j,t*_*]*) is the expected count for the species *i* in the site *j* and in the year *t* on the log-scale of predictors, *f*_*s*_ are smooth functions, Hab represents the habitat cover for each type of habitat *h* in the site *j* and in the year *t* and *C* each climatic variables *m* of interest in the site *j* and in the years *t − 1* and *t − 2* (12 climatic variables overall). After running the models, we assessed the concurvity for each pair of predictors, and the results showed no significant problems (Supplementary Information Table S2). Furthermore, GAMs work well also at high level of collinearity (Dormann et al. 2013) and the smooth estimation procedure in *mgcv* guarantees the reliability of the estimated parameters even in the presence of concurvity (Wood 2008). For all explanatory variables, we used the thin plate regression spline as a method of smoothing and penalised the smoothing process through the shrinkage method to avoid overfitting and exclude non-significant variables by decreasing the level of the estimated degrees of freedom close to zero (Wood 2017). To guarantee a reasonable ecological interpretation, we set the maximum possible effective degree of freedoms (edf) at two (*k* = 3), for each variable (Maggini et al. 2011; Massimino et al. 2015). The space–time smoother was handled as a full tensor product applying the shrinkage and setting the maximum edf to 26 (i.e. *k* = 3 for each interacting variable, a similar level used in Oedekoven et al. 2017). We used the restricted maximum likelihood (REML) as the smoothing parameter estimation method (Wood 2017). Smoothed effects of the climatic indices on counts (log-scale) were classified in the following categories: n.s. = non-significant effect (*p*-value > 0.05; Wood 2017); positive = monotonic and increasing functions also including asymptotic functions; negative = monotonic decreasing functions also including asymptotic functions; decreasing-increasing = functions showing a decrease first and then an increase (e.g. parabola with upward concavity) and increasing–decreasing = functions showing an increase first and then a decrease (e.g. parabola with downward concavity). Effects for smooth functions with *p*-value ≤ 0.05 were assessed through a visual inspection and by computing their first derivative using the function *derivatives* in the R package *gratia* (Simpson 2022). Moreover, uncertainty around the estimated smooths and the uncertainty around the first derivatives were considered to better characterise the relationship. We specified, ‘high uncertainty’ when the confidence interval around the first derivative included zero over the whole range of values of the explanatory variable, ‘moderate uncertainty’ when the confidence interval around the first derivative included zero for a subset of the range and ‘low uncertainty’ otherwise (Supplementary Information Fig. S2). Furthermore, we identified the species showing a greater sensitivity to CLEXs (considering the whole set of 12 indices, *t − 1* and *t − 2* separately, winter T-based, breeding T-based indices and R-based indices) as those significantly affected (*p*-value of the smooth ≤ 0.05) by at least two-thirds (66%) of the indices on the total (we also adopted two more conservative criteria, 75% and 100% on the total of the indices for each category). For these species, the prevalent type of relationship for each group of indices was assigned to a specific category among the four ones (positive, negative, decreasing-increasing and increasing–decreasing) when at least 50% of all statistically significant effects were included in the specific category. In cases of equal split for the type of relationship (i.e. 50% of the effects shared between two categories), both of them were assigned.

## Results

**Fig. 2:**
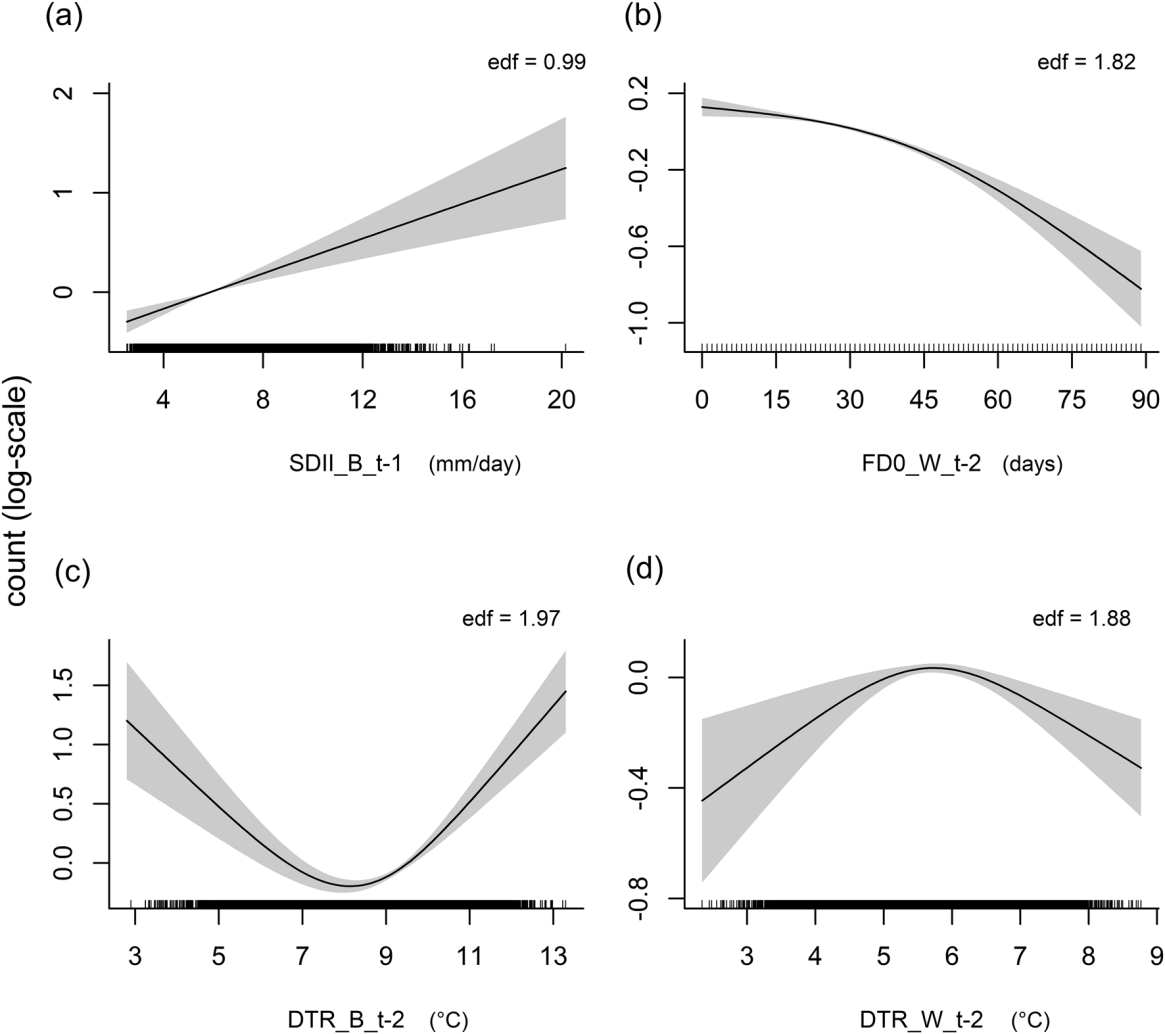
Examples of the four types of response curves in relation to the indices of CLEXs. Partial effects plots describe the relationship between the expected count (*y*-axis, log-scale with the smooth function centred around zero) and the climatic indices. Edf (*p*-value < 0.001 in the showed cases) represents the edf estimated for the smooth function. Rugs on the *x*-axis represent the distribution of values of the explanatory variable. The grey area represents the 95% confidence interval for the regression line. A positive effect of the simple precipitation intensity index (SDII) is shown for the Eurasian siskin (*Spinus spinus*) in (**a**), a negative effect of frost days (FD0) for the white wagtail (*Motacilla alba*) in (**b**), a decreasing–increasing effect of the daily temperature range (DTR) for the northern raven (*Corvus corax*) in (**c**) and an increasing–decreasing effect of DTR for the Eurasian bullfinch (*Pyrrhula pyrrhula*) in (**d**). After the acronym of the climatic index, B indicates the breading season, W the winter season and *t* − *1* and *t* − *2* the year used for the association between the climatic index and bird counts

**Fig. 3:**
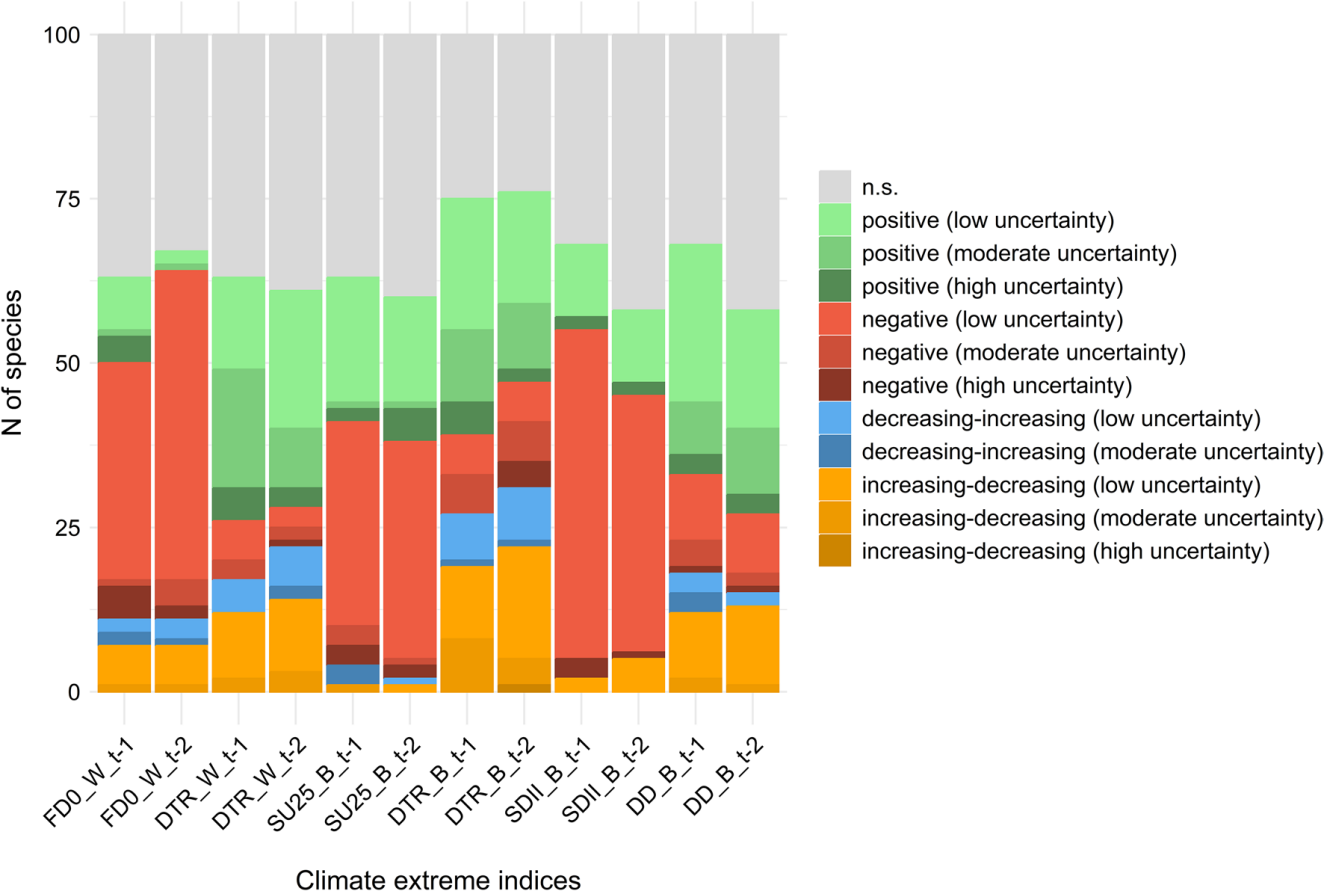
Bar chart showing the repartition of the types of effects for the indices of CLEXs across the 100 species under study. *FD0* frost days, *DTR* daily temperature range, *SU25* summer days, *SDII* simple precipitation intensity index, *DD* dry days, *W* winter season, *B* breading season; *t* − *1* and *t* − *2* indicate the year used for the association between the climatic indices and bird counts. See Table 1 in ‘Materials and methods’ for details on the climatic indices. N.s. (in grey) indicates that the effect was not statistically significant. Statistically significant effects are classified into four main categories (positive: green, negative: red, decreasing–increasing: blue, increasing–decreasing: orange, see ‘Materials and methods’ for the explanation), and reported with the corresponding degree of uncertainty (from low to high) (see ‘Materials and methods’ and Supplementary Information Fig. S2) (color figure online)

We analysed 100 bird species overall. For all of them, based on AIC, negative binomial GAMs outperformed Poisson GAMs, revealing the presence of overdispersion in count data for all species (estimated overdispersion parameter *θ* in negative binomial GAMs: mean = 0.568, range = 0.002–3.323) (Supplementary Information Table S3). The explained deviance (median = 32.61%) ranged from 4.33% for the Eurasian sparrowhawk (*Accipiter nisus*) to 88.42% for the rose-ringed parakeet (*Psittacula krameri*) (Supplementary Information Table S4). Habitat and elevation, together, explained on average a larger proportion of the total deviance (median = 38.56%, range = 0.41–73.79%) compared to CLEXs (median = 5.17%, range = 0.20–50.40%). All species were affected (*p*-value of the estimated smooth ≤ 0.05) by one climatic index at least (median number of statistically significant effects of the climatic indices for each species = 8, range = 1–12). In the case of statistically significant effects, negative effects (e.g. Fig. 2a) prevailed for FD0, SU25 and SDII, while positive effects (e.g. Fig. 2b) were mainly found for daily temperature range (DTR) in both seasons and DD (Fig. 3, Appendix S1). Decreasing–increasing effects (e.g. Fig. 2c) were rarer, while increasing–decreasing effects (e.g. Fig. 2d) were mainly detected for DTR (in both seasons) and DD (Fig. 3, Appendix S1). Indices of CLEXs widely influenced (*p*-value ≤ 0.05) the expected counts (log-scale) both at time *t − 1* and *t − 2* (number of species affected at *t − 1*: median = 65.5, range = 63–75; *t − *2: median: 60.5, range = 58–76). Two-year lagged effects (indices at time *t − 2*) affected a similar number of species compared to the indices of the previous year *t − 1*, except for SDII and DD where a lesser number of significant effects were detected (58 species for both indices in *t − 2* while 68 in *t − 1*) (Fig. 3). FD0 showed negative effects for a greater number of species in *t − 2* compared to *t − 1* (53 vs. 39, respectively), while the opposite was found for SDII (40 for *t − 2* and 53 for *t − 1*). For most of the species, the response curve for the same climatic index was quite similar when comparing *t − 1* and *t − 2*, but there were some exceptions (Appendix S1). In some cases, significant effects were found in *t − 1* but not in *t − 2* (e.g. SU25 for the willow ptarmigan *Lagopus lagopus*), or vice versa (e.g. FD0 for the yellowhammer *Emberiza citrinella*) (Appendix S1). Less frequently, responses were different between *t − 1* and *t − 2* and in most of cases with small differences (e.g. DTR in the breeding season for the European robin *Erithacus rubecula,* Appendix S1).

Fifty-eight species showed a greater sensitivity to CLEXs overall (i.e. species where at least two-thirds of the 12 climate indices affected the response; *p*-value ≤ 0.05; this number was reduced to 41 and 7 species with a 75% and 100% threshold, respectively). Among them, the prevalent relationship, for species for which at least a half of the total significant effects were assigned to a unique category, was positive for 14 species and negative for 18 species (Table 2). Within the taxonomic orders with at least four species, all species of Columbiformes (Eurasian collared dove *Streptopelia decaocto*, common wood pigeon *Columba palumbus*, feral pigeon, stock dove *Columba oenas*), half of the species of Accipitriformes and Charadriiformes, 74% of species belonged to Passeriformes showed to be sensitive to all indices. Conversely, Strigiformes were not particularly sensitive to CLEXs overall, and only the short-eared owl (*Asio flammeus*) and the tawny owl were largely and negatively influenced by T-based climatic indices in the breeding season (Table 2). When considering indices in relation to 1 (*t − 1*) or 2-year (*t − 2*) lagged effects separately, we found 68 species (39 and 16 species with a 75% and 100% threshold, respectively) showing a greater sensitivity for *t − 1* and 61 species (35 and 16 species with a 75% and 100% threshold, respectively) for *t − 2*. In the first case, the prevalent relationship was positive for 26 species, negative for 31 species and decreasing–increasing in two ones. Similarly, the prevalent relationship in *t − 2* was positive for 20 species, negative for 29 species and increasing–decreasing in 6 ones (Table 2). Fifty-three species (53 and 26 species with a 75% and 100% threshold, respectively) exhibited a greater sensitivity for winter T-based indices, with 22 prevalent positive responses, 29 negative, three decreasing–increasing and six increasing–decreasing. Sixty-one species (61 and 32 species with a 75% and 100% threshold, respectively) showed a greater sensitivity for breeding T-based indices, with 31 positive relationships, 30 negative, three decreasing–increasing and seven increasing–decreasing. Fifty-five species (55 and 26 species with a 75% and 100% threshold, respectively) displayed a greater sensitivity for R-based indices, with 24 positive prevalent relationships, 40 negative, one decreasing–increasing and seven increasing–decreasing (Table 2). Columbiformes showed a high sensitivity to each group of climatic indices (three out of four species showed significant responses to winter T-based indices, and all species to breeding T-based and R-based indices), and Anseriformes showed a high sensitivity to R-based indices (6 out of 11 species). Passeriformes, which in our study included 18 different families and 46% of the analysed species, showed a high sensitivity to breeding T-based indices (80% of them showed significant responses to two-thirds of the indices belonging to this group). In addition, they also showed considerable responses to breeding R-based and winter T-based indices (63% of the species for both cases). Among these species, 45% of the responses were negative in the case of winter T-based indices (Table 2).

**Table 2:** Number of statistically significant effects of CLEXs and type of relationship for the 100 species analysed

Species	Order	CLEXs (tot)	CLEXs (*t − 1*)	CLEXs (*t − 2*)	T-based (W)	T-based (B)	R-based (B)
Little grebe	Podicipediformes	5	4(P)^a^	1	2	1	2
Great crested grebe	Podicipediformes	5	3	2	3(N)^a,b^	2	0
Great cormorant	Suliformes	6	3	3	2	1	3(N)^a,b^
Little egret	Pelecaniformes	7	4(N)^a^	3	3(N)^a,b^	1	3^a,b^
Grey heron	Pelecaniformes	11(P)^a,b^	6(P)^a,b,c^	5(P)^a,b^	4(P,N)^a,b,c^	4(P)^a,b,c^	3(N)^a,b^
Mute swan	Anseriformes	9(P)^a,b^	4(P,N)^a^	5(P)^a,b^	3(P)^a,b^	3(P)^a,b^	3(N)^a,b^
Greylag goose	Anseriformes	8^a^	4^a^	4(N)^a^	2	3(∪)^a,b^	3(N)^a,b^
Canada goose	Anseriformes	7	4(P,N)^a^	3	3(N)^a,b^	2	2
Egyptian goose	Anseriformes	8(P)^a^	4(P)^a^	4(P,N)^a^	3(P)^a,b^	2	3(N)^a,b^
Common shelduck	Anseriformes	7	4(∪)^a^	3	2	3(N)^a,b^	2
Mandarin duck	Anseriformes	3	2	1	1	1	1
Gadwall	Anseriformes	5	3	2	1	2	2
Eurasian teal	Anseriformes	3	3	0	1	1	1
Mallard	Anseriformes	9^a,b^	4(P,N)^a^	5^a,b^	4(P,N)^a,b,c^	2	3(N)^a,b^
Tufted duck	Anseriformes	6	3	3	2	1	3(N)^a,b^
Common merganser	Anseriformes	8(P)^a^	3	5^a,b^	2	3(P)^a,b^	3(P)^a,b^
Red kite	Accipitriformes	11^a,b^	6^a,b,c^	5^a,b^	4(P)^a,b,c^	4(P, ∪)^a,b,c^	3(N)^a,b^
Western marsh harrier	Accipitriformes	3	1	2	0	3^a,b^	0
Hen harrier	Accipitriformes	3	1	2	2	1	0
Northern goshawk	Accipitriformes	5	2	3	2	2	1
Eurasian sparrowhawk	Accipitriformes	8(N)^a^	4(N)^a^	4(N)^a^	2	2	4(P,N)^a,b,c^
Common buzzard	Accipitriformes	8^a^	4(P)^a^	4(N)^a^	3(∩)^a,b^	4(P,N)^a,b,c^	1
Common kestrel	Falconiformes	6	3	3	1	2	3(N)^a,b^
Merlin	Falconiformes	5	4(N)^a^	1	3(N)^a,b^	1	1
Peregrine falcon	Falconiformes	5	4(P)^a^	1	1	2	2
Willow ptarmigan	Galiiformes	8(N)^a^	5(N)^a,b^	3	2	3(∩)^a,b^	3(N)^a,b^
Black grouse	Galiiformes	3	3	0	1	1	1
Red-legged partridge	Galiiformes	7	3	4^a^	3(P)^a,b^	2	2
Grey partridge	Galiiformes	5	3	2	1	1	3(P)^a,b^
Common pheasant	Galiiformes	9^a,b^	5^a,b^	4(∩)^a^	1	4(P,N)^a,b,c^	4(∩)^a,b,c^
Indian peafowl	Galiiformes	2	1	1	0	2	0
Common moorhen	Gruiformes	10(P)^a,b^	6(P,N)^a,b,c^	4(P)^a^	3(P)^a,b^	3^a,b^	4(P,N)^a,b,c^
Eurasian coot	Gruiformes	10(P)^a,b^	6^a,b,c^	4(P)^a^	4(N)^a,b,c^	3(P)^a,b^	3(P)^a,b^
Eurasian oystercatcher	Charadriiformes	9(N)^a,b^	4(N, ∪)^a^	5(N)^a,b^	3(∪)^a,b^	3(N)^a,b^	3(N)^a,b^
Common ringed plover	Charadriiformes	4	1	3	1	2	1
European golden plover	Charadriiformes	7	3	4(N)^a^	3^a,b^	2	2
Northern lapwing	Charadriiformes	7	3	4(N)^a^	2	1	4(N, ∩)^a,b,c^
Common snipe	Charadriiformes	8^a^	4(P)^a^	4(N)^a^	2	2	4(N, ∩)^a,b,c^
Eurasian woodcock	Charadriiformes	1	1	0	0	0	1
Eurasian curlew	Charadriiformes	8^a^	5^a,b^	3	3(∪)^a,b^	3(∩)^a,b^	2
Common redshank	Charadriiformes	9^a,b^	5^a,b^	4(N)^a^	3(∪)^a,b^	4(N)^a,b,c^	2
Feral pigeon	Columbiformes	11^a,b^	5(N)^a,b^	6^a,b,c^	3^a,b^	4(P,N)^a,b,c^	4(P,N)^a,b,c^
Stock dove	Columbiformes	8(N)^a^	4(P,N)^a^	4(N)^a^	2	3(N)^a,b^	3(N)^a,b^
Common wood pigeon	Columbiformes	12^a,b,c^	6^a,b,c^	6(P)^a,b,c^	4(P, ∩)^a,b,c^	4^a,b,c^	4(P,N)^a,b,c^
Eurasian collared dove	Columbiformes	10(N)^a,b^	4(P,N)^a^	6(N)^a,b,c^	3(P)^a,b^	3(N)^a,b^	4(P,N)^a,b,c^
Rose-ringed parakeet	Psittaciformes	5	3	2	3(N)^a,b^	1	1
Western barn owl	Strigiformes	4	2	2	2	0	2
Little owl	Strigiformes	4	1	3	2	2	0
Tawny owl	Strigiformes	7	4(P,N)^a^	3	2	4(N)^a,b,c^	1
Short-eared owl	Strigiformes	7	3	4(P,N)^a^	2	3(N)^a,b^	2
Common kingfisher	Coraciiformes	8(P)^a^	5(P)^a,b^	3	4(P,N)^a,b,c^	3(P)^a,b^	1
European green woodpecker	Piciformes	10(N)^a,b^	6(N)^a,b,c^	4(N)^a^	4(N)^a,b,c^	3(P)^a,b^	3(N)^a,b^
Great spotted woodpecker	Piciformes	10(P)^a,b^	5(P)^a,b^	5(N)^a,b^	4(P)^a,b,c^	3(P)^a,b^	3(N)^a,b^
Lesser spotted woodpecker	Piciformes	2	1	1	0	1	1
Eurasian skylark	Passeriformes	11^a,b^	6^a,b,c^	5^a,b^	4(∩)^a,b,c^	4(N, ∪)^a,b,c^	3(P)^a,b^
Meadow pipit	Passeriformes	12(N)^a,b,c^	6(N)^a,b,c^	6(P,N)^a,b,c^	4(N)^a,b,c^	4(N)^a,b,c^	4(P)^a,b,c^
Grey wagtail	Passeriformes	10(P)^a,b^	5(P)^a,b^	5(P)^a,b^	4(P,N)^a,b,c^	3(P)^a,b^	3(N)^a,b^
White wagtail	Passeriformes	10(N)^a,b^	5^a,b^	5(N)^a,b^	3(N)^a,b^	4(N)^a,b,c^	3(P)^a,b^
White-throated dipper	Passeriformes	9(N)^a,b^	4(P,N)^a^	5(N)^a,b^	4(P,N)^a,b,c^	3(P)^a,b^	2
Eurasian wren	Passeriformes	12^a,b,c^	6(N)^a,b,c^	6(∩)^a,b,c^	4(P,N)^a,b,c^	4(∩)^a,b,c^	4(N, ∩)^a,b,c^
Dunnock	Passeriformes	10(N)^a,b^	5(N)^a,b^	5(N)^a,b^	4(N)^a,b,c^	2	4(P,N)^a,b,c^
European robin	Passeriformes	12(N)^a,b,c^	6(N)^a,b,c^	6^a,b,c^	4(N)^a,b,c^	4(P)^a,b,c^	4(N)^a,b,c^
European stonechat	Passeriformes	10(N)^a,b^	5^a,b^	5(N)^a,b^	3(N)^a,b^	4(N)^a,b,c^	3(P)^a,b^
Common blackbird	Passeriformes	12(N)^a,b,c^	6(N)^a,b,c^	6(N, ∩)^a,b,c^	4(N)^a,b,c^	4(N)^a,b,c^	4(N, ∩)^a,b,c^
Fieldfare	Passeriformes	8(P)^a^	4(P)^a^	4(P)^a^	3(P)^a,b^	3(P)^a,b^	2
Song thrush	Passeriformes	11^a,b^	5(N)^a,b^	6(∩)^a,b,c^	3(N)^a,b^	4(N)^a,b,c^	4(∩)^a,b,c^
Redwing	Passeriformes	6	3	3	1	1	4(P,N)^a,b,c^
Mistle thrush	Passeriformes	7	3	4(P)^a^	3(N)^a,b^	3(P)^a,b^	1
Cetti’s warbler	Passeriformes	6	2	4(P,N)^a^	3(N)^a,b^	1	2
Goldcrest	Passeriformes	10^a,b^	5^a,b^	5^a,b^	4(N)^a,b,c^	4(P)^a,b,c^	2
Long-tailed tit	Passeriformes	7	3	4(P)^a^	3(P)^a,b^	2	2
Marsh tit	Passeriformes	6	4(P)^a^	2	2	3(P)^a,b^	1
Willow tit	Passeriformes	5	2	3	1	3(P)^a,b^	1
Coal tit	Passeriformes	10(P)^a,b^	6(P)^a,b,c^	4(P)^a^	2	4(P,N)^a,b,c^	4(P)^a,b,c^
Eurasian blue tit	Passeriformes	10(P)^a,b^	6(P)^a,b,c^	4(P,N)^a^	4(∩)^a,b,c^	3(P)^a,b^	3(N)^a,b^
Great tit	Passeriformes	11^a,b^	5^a,b^	6(P)^a,b,c^	4(P)^a,b,c^	4(P)^a,b,c^	3(N)^a,b^
Eurasian nuthatch	Passeriformes	8^a^	4(P)^a^	4(N)^a^	1	3(P)^a,b^	4(N, ∩)^a,b,c^
Eurasian treecreeper	Passeriformes	10(P,N)^a,b^	5(N)^a,b^	5(P)^a,b^	4(P,N)^a,b,c^	4(P,N)^a,b,c^	2
Eurasian jay	Passeriformes	8(P)^a^	5^a,b^	3	2	4(P)^a,b,c^	2
Eurasian magpie	Passeriformes	11(N)^a,b^	5(N)^a,b^	6(N)^a,b,c^	4(N)^a,b,c^	4(P)^a,b,c^	3(N)^a,b^
Western jackdaw	Passeriformes	11^a,b^	6(N)^a,b,c^	5^a,b^	4(P)^a,b,c^	3(N)^a,b^	4(P,N)^a,b,c^
Rook	Passeriformes	11^a,b^	5^a,b^	6(P)^a,b,c^	3(P)^a,b^	4(N)^a,b,c^	4(P,N)^a,b,c^
Carrion crow	Passeriformes	7	4(N)^a^	3	2	4(P,N)^a,b,c^	1
Hooded crow	Passeriformes	9(N)^a,b^	4(N)^a^	5^a,b^	2	3(N)^a,b^	4(∪)^a,b,c^
Northern raven	Passeriformes	7	2	5^a,b^	3(N)^a,b^	2	2
Common starling	Passeriformes	12^a,b,c^	6(P)^a,b,c^	6(∩)^a,b,c^	4^a,b,c^	4(P, ∩)^a,b,c^	4(N)^a,b,c^
House sparrow	Passeriformes	11^a,b^	5^a,b^	6(N)^a,b,c^	4(P,N)^a,b,c^	3(∩)^a,b^	4(P,N)^a,b,c^
Eurasian tree sparrow	Passeriformes	10^a,b^	5^a,b^	5^a,b^	2	4(N, ∩)^a,b,c^	4(P,N)^a,b,c^
Common chaffinch	Passeriformes	8^a^	4(P)^a^	4(N)^a^	3(∩)^a,b^	4(P,N)^a,b,c^	1
European greenfinch	Passeriformes	12(N)^a,b,c^	6(N)^a,b,c^	6(N)^a,b,c^	4(P,N)^a,b,c^	4(N)^a,b,c^	4(P,N)^a,b,c^
European goldfinch	Passeriformes	8^a^	5^a,b^	3	2	3(∩)^a,b^	3(N)^a,b^
Eurasian siskin	Passeriformes	9^a,b^	5(N)^a,b^	4(P)^a^	1	4(P,N)^a,b,c^	4(P)^a,b,c^
Common linnet	Passeriformes	10(N)^a,b^	4(N)^a^	6(N)^a,b,c^	2	4(N)^a,b,c^	4(P,N)^a,b,c^
Twite	Passeriformes	2	1	1	0	2	0
Lesser redpoll	Passeriformes	7	4(N)^a^	3	1	3(N)^a,b^	3(N)^a,b^
Red crossbill	Passeriformes	6	2	4(P)^a^	2	2	2
Eurasian bullfinch	Passeriformes	8^a^	4(P)^a^	4(∩)^a^	2	4(P,N)^a,b,c^	2
Yellowhammer	Passeriformes	10^a,b^	4^a^	6(N)^a,b,c^	3(∩)^a,b^	4(N)^a,b,c^	3(P)^a,b^
Common reed bunting	Passeriformes	8^a^	5^a,b^	3	4(N)^a,b,c^	2	2
Corn bunting	Passeriformes	6	4(P,N)^a^	2	1	2	3(P)^a,b^

Species are listed with the common name, following the taxonomic order and according to the International Ornithological Committee (IOC) World Bird List (Gill et al. 2022). In each column, the number of statistically significant effects is reported with a number, and the type of relationship is shown in parenthesis. ‘CLEXs (tot)’ includes all the 12 indices of CLEXs, ‘CLEXs (*t* − *1*)’ the six indices at time *t* − *1* and ‘CLEXs (*t* − *2*)’ the six indices at time *t* − *2*. ‘T-based (W)’ includes the four temperature-based indices during the winter season, ‘T-based (B)’ the four temperature-based indices during the breading season and ‘R-based (B)’ the four rainfall-based indices during the breading season. For these last three indices, both time *t* − *1* and *t* − *2* were considered. The type of relationship has been reported when at least two-thirds of the indices in the group had affected the species abundance (*p*-value ≤ 0.05), and there was a prevalent type of relationship (i.e. ≥ 50% of the total significant effects was assigned to the same category; see ‘Materials and methods’ for the applied criterion). When the type of relationship was equally split between two categories, both of them were reported. *N* negative, *P* positive, ∪ = decreasing–increasing, ∩ = increasing–decreasing. Superscripts indicate species sensitivity according to the three criteria: a = 66% threshold (i.e. at least two-thirds of the indices affected species abundance), b = 75% threshold (i.e. at least three-quarter of the indices affected species abundance), c = 100% threshold (i.e. all the indices affected species abundance)

## Discussion

Climate change remains one of the most important challenges for biodiversity conservation, and understanding how CLEXs interact with wild populations is critical for planning adequate strategies and to predict future biodiversity changes (Roberts et al. 2019). Here we assessed, at single-species level, the effects of CLEXs on relative abundance (annual counts of individuals at survey sites) for 100 resident bird species over large geographic extents and temporal scale using the UK BBS (> 69,000 sampled sites over 25 years) and identified the species showing a greater sensitivity to CLEXs among resident birds. Although our analyses do not test the overall responses of CLEXs on the entire pool of species while accounting for the inter-specific variations (e.g. through implementing a generalised additive mixed model that included the species entity as random intercept) nor do they rely on a modelling framework at community level (e.g. Hierarchical Modelling of Species Communities, Ovaskainen et al. 2017), we found a robust evidence of widespread and significant effects of CLEXs on bird-relative abundances, with both 1- and 2-year lagged effects. The flexible GAM framework allowed detection of both linear and non-linear response curves that may often characterise species responses to climatic variables (Pearce-Higgins and Crick 2019). In several cases, response curves revealed the presence of threshold-like responses, the identification of which is crucial for assessing the effect of environmental pressures on biological system (Bailey and van de Pol 2016).

### Effects of climate extremes

Our findings demonstrated the existence of widespread effects of CLEXs on bird populations. Number of winter FD0, a measure of winter severity, showed a clear negative effect for most of the species. Winter severity can negatively affect the survival of individuals, with greater effects on first-year birds compared to adults (Robinson et al. 2004, 2007). For FD0, we found a greater proportion of negative effects for the 2-year lagged index compared to 1-year lagged one. This might depend on the fact that roughly one-third of the analysed species reach the age of reproduction later than the first year (Storchová and Hořák, 2018), thus resulting in a delayed effect over time. In addition, we cannot exclude that winter severity might indirectly influence population dynamics through bottom-up processes (e.g. food availability) that may act on older individuals as well. As the long-term trend for the number of FD0 (Supplementary Information Fig. S1a) has been negative in our study area, the negative effect of winter severity could lessen in the future. SU25, used as a proxy of prolonged extreme hot temperatures during the breeding season, showed a large proportion of negative effects (36% and 37% of the total of the species in *t − 1* and *t − 2*, respectively). Some previous studies highlighted that hot temperatures in summer could negatively affect abundance in birds (Beale et al. 2006; Franks et al. 2017), although many species show overall positive responses between spring temperature and population growth rates (Pearce-Higgins et al. 2015). High temperatures during the breeding season or in summer could affect the reproductive success of adults through direct effects on the reproductive performance (Conrey et al. 2016; Pattinson et al. 2022), but also through bottom-up processes affecting the abundance or availability of food resources (Pearce-Higgins 2010; Pearce-Higgins et al. 2010). However, we also found a consistent proportion of positive effects of SU25 (22% in both *t − 1* and *t − 2*). For example, abundance of some corvids (Corvidae) such as the Eurasian magpie (*Pica pica*), the carrion crow (*Corvus corone*) and the Eurasian jay (*Garrulus glandarius*), as well as the common starling (*Sturnus vulgaris*) and the great tit (*Parus major*), positively responded to SU25. These species are often present in urban environments, and the effects of hot temperatures could be influenced by habitat characteristics with dampened or reverse effects in urban areas (Pipoly et al. 2022). Differently from FD0 and SU25, the daily temperature range (DTR) showed a smaller proportion of negative effects, especially during the winter season. DTR may be an important predictor for species distribution and occurrence in animals (Sutton et al. 2022), but how wild animal populations respond to this climatic parameter remains barely investigated in ecological studies on climate change. Our results revealed prevalent positive effects of DTR on bird-relative abundance, and a significant proportion (12–22%) of an initial positive effect followed by a negative effect (increasing–decreasing response curve). These findings suggested that beyond a threshold (roughly 5–6 ℃ in winter and 8–10 ℃ in the breeding season, Appendix S1), the positive effects disappeared and a further increase of DTR leads to clear negative responses in abundance, maybe linked to the increase of physiological stress that can vary in relation to foraging environment and thermal condition (Briga and Verhulst 2015). However, the degree of uncertainty of the response curve was greater for DTR compared to the other indices (Fig. 3), and the ecological response around the limit values of the index needs to be assessed prudently. In relation to the effects of precipitation on bird abundance during the breeding season, our findings highlighted that the intensity of rainfall, measured by the SDII, extensively and negatively influenced bird counts, while responses to drought, evaluated by the number of DD, varied across species. The precipitation intensity can affect birds in several ways: acting on survival, physiology, behaviour and perception of the surrounding environment including prey–predator detections (Sergio 2003; Whittingham et al. 2004; Wilson et al. 2004; Schöll and Hille 2020; Yorzinski 2020). On the contrary, several studies linked drought to negative consequences on reproductive success in birds (Robinson et al. 2004; Colón et al. 2017) and survival of adults (Robinson et al. 2004), with strong negative effects at higher trophic levels (Prugh et al. 2018). Severe droughts can also act through indirect ways, for example, by altering habitat conditions and structures where a species lives (Hinojosa-Huerta et al. 2013), or by favouring brood parasitism or nest predation in bird species (Colón et al. 2017). However, our findings did not show widespread negative effects of droughts on bird abundance, rather we found a prevalence of positive effects (35% and 31% of the species in *t − 1* and in *t − 2*, respectively). Palmer et al. (2017) highlighted that drought conditions might have weaker negative effects upon birds. In wetlands, for example, bird assemblages could be favoured by moderate drought conditions, especially those species feeding on aquatic and benthic fauna as a consequence of the surfacing of new foraging areas leading to a temporary increase in the size of feeding areas and food availability (Jitariu et al. 2022). In our study, wetland birds belonging to Anatidae, Rallidae, Scolopacidae, Haematopodidae, Charadriidae, Ardeidae, Podicipedidae and Phalacrocoracidae, Alcedinidae (27 species overall) were poorly affected by drought conditions (33% and 59% of non-significant effects, respectively in *t − 1* and *t − 2*) or showed positive responses in 26% of the cases in both *t − 1* and *t − 2*.

The results also showed widespread 2-year lagged effects. Bird population responses to climate change can show delays due to ecological and demographic processes (Jenouvrier 2013). Lagged effects can act in several ways, for example, through food webs (Ockendon et al. 2014), but also directly by affecting offspring recruitment (Sandvik et al. 2012; Saunders et al. 2021). Effects of climatic variables on a species can remain similar across years (or detected only in a single year), as we found for most of the analysed species, although they can sometimes be different (Sandvik et al. 2012). Contrasting effects may be more difficult to be explained and may depend on diverse processes involved. For example, we found contrasting effects of FD0 for the western barn owl, with a positive effect in the preceding winter (*t − 1*) and a negative effect in the 2-year preceding winter (*t − 2*) (Appendix S1). The negative effect could be partially explained by differential selective pressures acting between adults and juveniles (Altwegg et al. 2006), as well as by ecological factors (e.g. prey availability), whilst the positive effect at 1-year time lag suggests that other mechanisms could be involved.

### Species sensitivity

Species showing widespread responses to the whole set of 12 climatic variables (58%) belong to several and different avian taxonomic orders. It suggests CLEXs may affect avian populations independently from phylogenetic constraints, but further analyses should be developed to specifically test such a hypothesis. Many previous works had been focused on iconic or endangered birds (e.g. Conrey et al. 2016; Colón et al. 2017; Cleeland et al. 2020), but little attention has been given to multi-species studies (but see Cohen et al. 2020, 2021). Assessing the sensitivity and responses of multiple species to CLEXs, which are expected to be more and more common in the future (IPCC 2013), is crucial to identify how climate change could act on biological and ecological systems in the future. In this study, among the sensitive species to the whole set of 12 climate indices, 18 species showed negative responses, while 14 species showed positive responses. This means that such birds could be eligible sentinel species for studying CLEXs, and their sensitivity should be further assessed in future studies. Moreover, multi-species analyses can reveal overlooked patterns that are valuable for wildlife conservation. Despite that common or non-threatened species may currently need less conservation effort compared to threatened or rare ones, it does not mean that in a near future such species may suffer from a significant decline due to increasingly recurring extreme weather conditions. Some common and widespread species such as the common blackbird (*Turdus merula*) and the European robin, characterised by positive long-term population trends (Harris et al. 2022), were affected by negative responses to CLEXs, which emphasises that also such species could be impacted by future climate change. Their short-term population trends 2010–2020 (Harris et al. 2022), for example, highlighted a reduction in population growth rate (European robin) or a weak decline (common blackbird), that might (but it needs to be tested) depend on climate-induced effects. On the contrary, some species could benefit from extreme climatic conditions (Maxwell et al. 2019), likely due to local adaptations, higher tolerances, ecological plasticity or greater resilience capacity (Renton et al. 2018; Cooper et al. 2020; Pipoly et al. 2022).

When considering the effects of climatic indices with 1- or 2-year lagged effects separately, we found widespread effects across all taxonomic orders. Columbiformes showed high sensitivity in responses to CLEXs. Previous studies stressed heat tolerance capacity for this group (Pollock et al. 2021), but tolerances can vary greatly within avian orders (McKechnie et al. 2017), as we found for the response patterns in this taxon (Appendix S1). The high sensitivity of Anseriformes to rainfall-based indices, which was mainly characterised by negative responses (Table 2), may be linked to their dependency on water. Such species depend on water habitat for feeding, nesting and rearing chicks, and increases of water surface can negatively affect their abundance (Canepuccia et al. 2007). The increase of rainfall intensity during restricted time intervals could cause unexpected rise of the water table during vulnerable phases of the life cycle (i.e. breeding season), leading to significant negative consequences likely involving offspring recruitment. Observed responses to SDII for this group (Appendix S1) seem to confirm such a negative pattern.

Finally, Passeriformes showed a high sensitivity to each of the three groups of indices (winter T-based, breeding T-based and breeding R-based), especially to breeding T-based indices for which they reached 80% of significant responses. Furthermore, 45% of responses to winter T-based indices across the 29 sensitive species of Passeriformes was negative. One of the reasons of sensitivity in Passeriformes could be linked to their relative body size. Smaller birds could deeply suffer from hot and cold thermal physiological stress, resulting in higher fitness costs (Albright et al. 2017; Brodin et al. 2017) that likely make small birds more susceptible to severe temperatures during both the breeding and the winter seasons, but responses could vary in relation to habitat characteristics (Freeman et al. 2022).

## Conclusions

Assessing species responses to CLEXs could represent a critical goal to predict population dynamics and species distributions. The findings highlighted widespread and significant effects of CLEXs on bird-relative abundances, with both 1- and 2-year lagged effects. Identifying suitable proxies to measure the magnitude of CLEXs in a biological perspective is crucial for future research. Indices used in this study, which are defined by a climatological point of view, were suitable for assessing biological responses. This work also identified several species that were more susceptible to the effects of CLEXs and the direction of responses. Such species may undergo greater biological consequences due to their higher sensitivity to CLEXs. Moreover, they could be an assortment of species from which testing the suitability as bioindicators of climate extremes in future studies. CLEXs could exacerbate biological responses of avian populations placing new challenges for their conservation but also for biodiversity and ecological processes in general, because of the importance of birds in ecosystems and biological communities (Şekercioğlu et al. 2016). Understanding both direct and indirect mechanisms through which CLEXs can affect wild populations should be a primary goal. For this purpose, future studies need to focus on mechanistic processes of CLEXs, analysing effects on population demographic parameters, including bottom-up effects resulting from both species interactions and variation in resource availability (Pearce-Higgins et al. 2010). Furthermore, climate change can also interact with other factors, such as changes in land use and habitat loss, with synergistic actions whose effects on populations or biodiversity could be unexpected from individual analyses of these drivers (Mantyka-pringle et al. 2012; Rocchia et al. 2018; Bani et al. 2019). Investigating the consequences of such interactions and disentangling their relative contribution would allow obtaining essential data for a better and more exhaustive understanding of species responses to a changing environment.

### Supplementary Information

Below is the link to the electronic supplementary material.

Supplementary file1 (PDF 21894 KB)

## Data Availability

The data that support the findings of this study are available from the British Trust for Ornithology, BTO, The Nunnery, Thetford, Norfolk, IP24 2PU, UK, upon reasonable request at the following link: https://www.bto.org/our-science/data/data-request-system.
