# Supplementary material for: Avian responses to climate extremes: insights into abundance curves and species sensitivity using the UK Breeding Bird Survey
Source: Oecologia. 2024 Jan 20;204(1):241–55. doi: 10.1007/s00442-023-05504-9 (PMC10830718; doi:10.1007/s00442-023-05504-9)

Supplementary Information

Figure S1. Long-term trends 1961–2018 and short-term trends 1992-2018 for (a) FD0, (b) DTR_W, (c) SU25, (d) DTR_B, (e) SDII, (f) DD throughout the study area (the UK).

Table S1. Covariates used in the models in addition to the climatic variables.

Table S2. Concurvity matrix for each pair of predictors included in the models according to the most pessimistic method.

Figure S2. Criterion used for the assignment of "low uncertainty", "moderate uncertainty" or "high uncertainty" in addition to the main categories describing the type of relationship between bird counts and climate extremes.

Table S3. AIC comparison between Poisson and negative binomial GAMs.

Table S4. Explained deviance (%) for negative binomial GAMs.

Appendix S1. Effects of the climate extreme indices on bird relative abundance.

Figure S1. Long-term trends 1961–2018 and short-term trends 1992-2018 for (a) FD0_W, (b) DTR_W, (c) SU25_W, (d) DTR_B, (e) SDII_B, (f) DD_B throughout the study area (the UK). W= winter season, B= breeding season. Trends were assessed by generalised additive models (GAMs), using the thin plate regression spline as method of smoothing and the restricted maximum likelihood (REML) to estimate the smoothing parameter. Annual median values throughout the whole of the UK at 1-km square resolution (1,305,000 values for each year) were used to assess trends. The estimated degree of the smooth function (edf) and the corresponding p-value are showed at the top of the graph.

(a) Winter frost days (FD0_W).

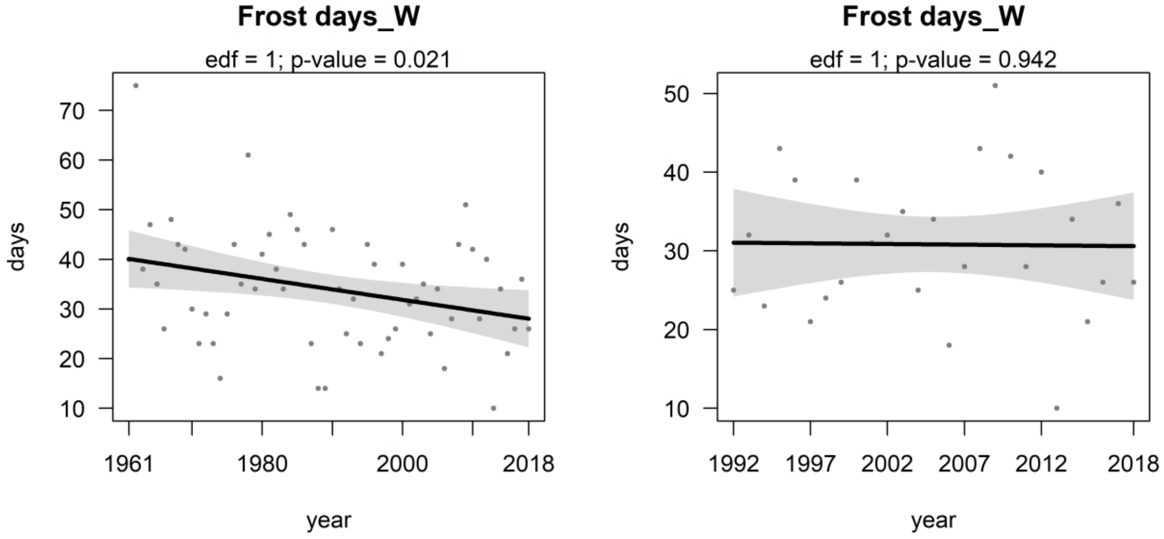

(b) Winter daily temperature range (DTR_W)

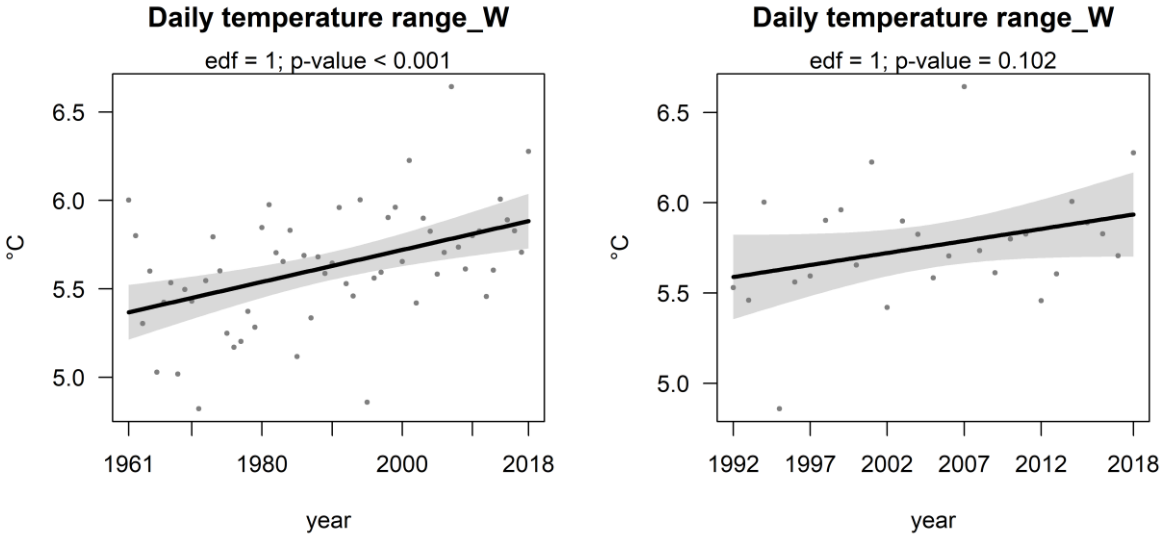

(c) Breeding summer days (SU25_B).

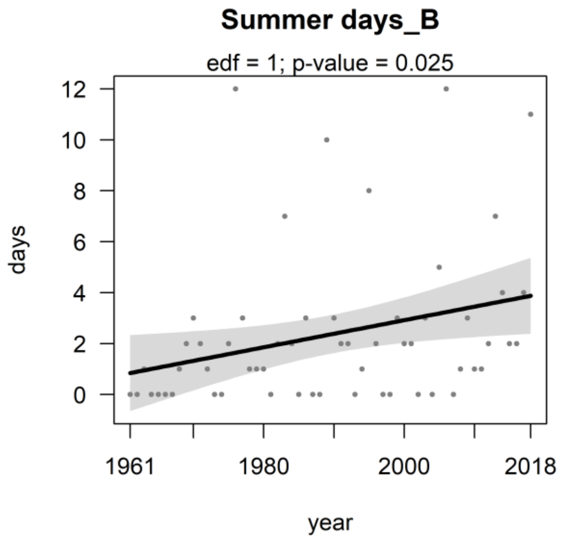

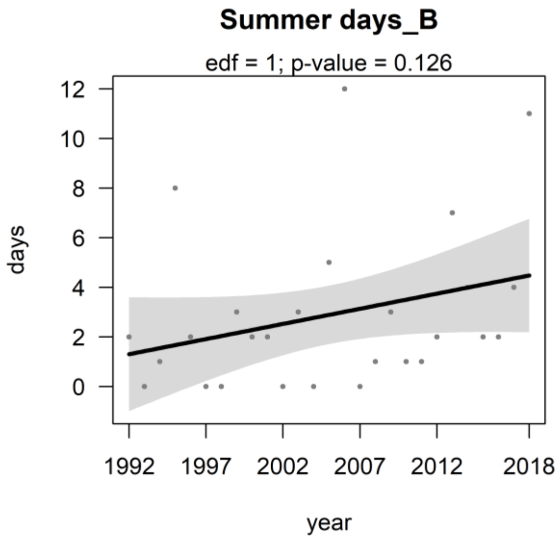

(d) Breeding daily temperature range (DTR_B).

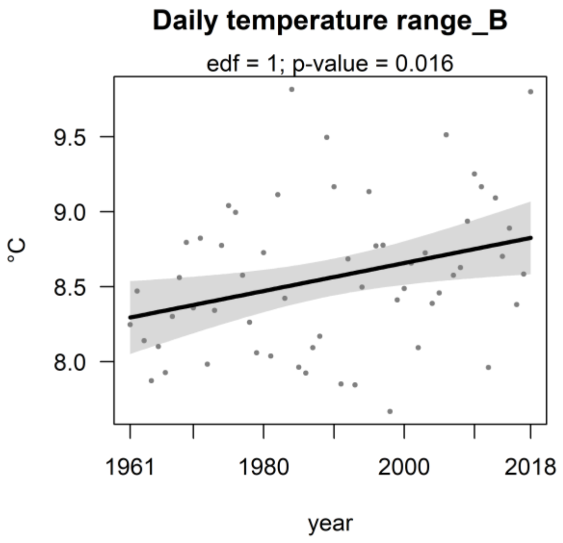

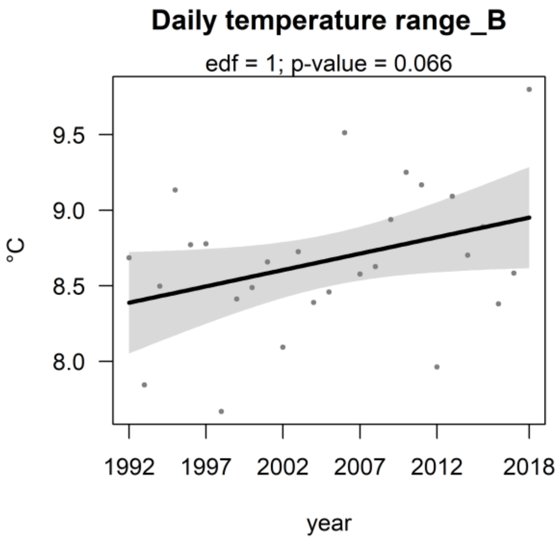

(e) Breeding simple intensity precipitation index (SDII_B).

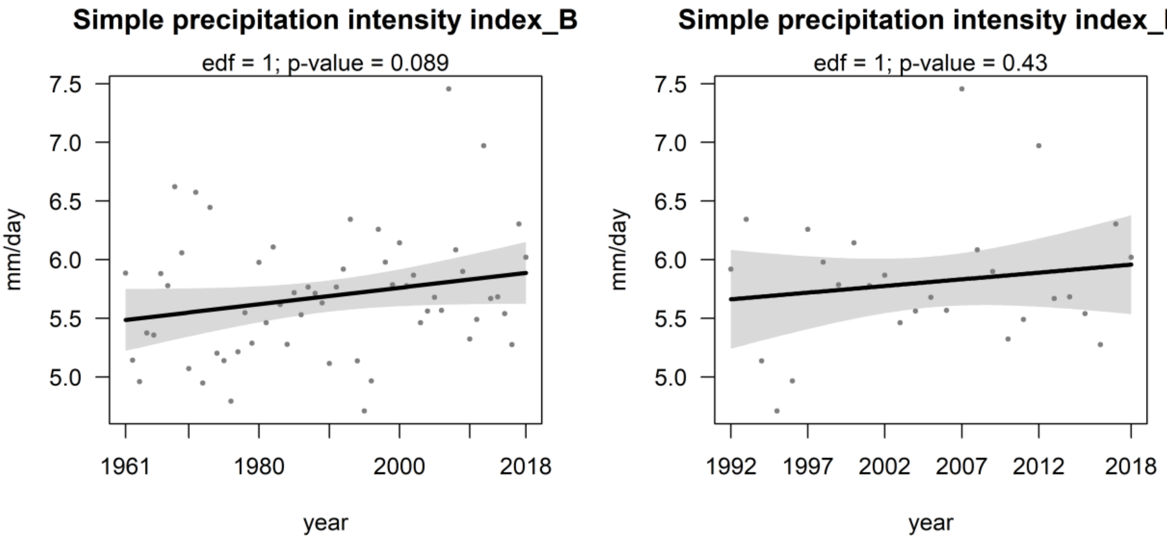

(f) Breeding dry days (DD_B).

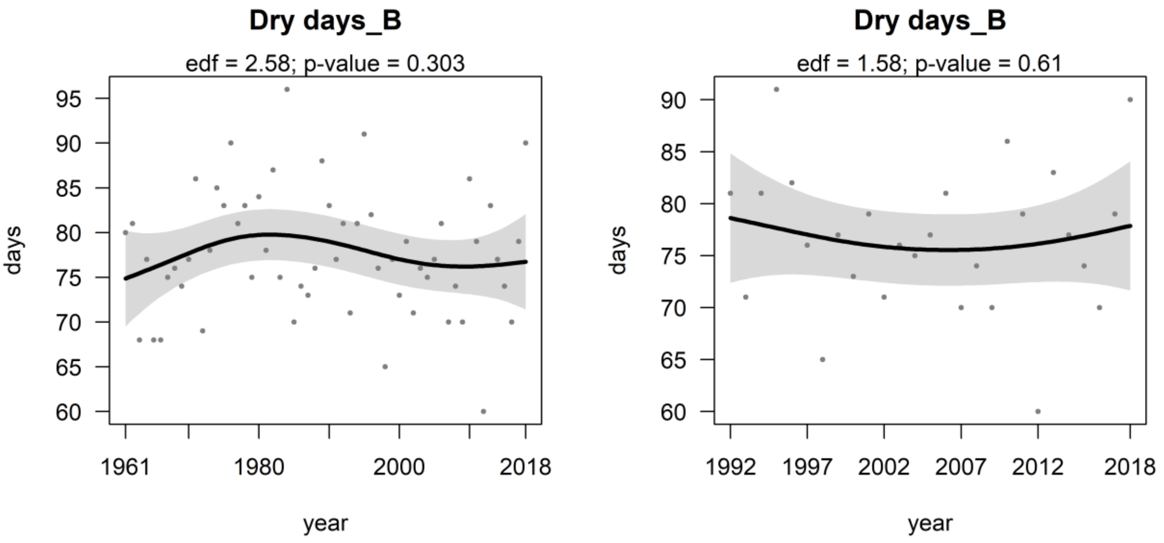

Table S1. Covariates used in the models in addition to the climatic variables.

Habitat covers were calculated from information collected by surveyors describing the main types of habitats characterising each transect within each 1 ×1 km square.

Covariate	Definition	Unit
Elev	Elevation at 1-km resolution in the BBS square	m
Hab A	Woodland	%
Hab B	Scrubland	%
Hab C	Semi-natural grassland and marsh	%
Hab D	Heathland and bogs	%
Hab E	Farmland	%
Hab F	Human sites	%
Hab G	Waterbodies	%
Hab H	Coastal	%
Hab I	Inland rock	%
Northing	Northing of the bottom left corner of the sampling unit (1-km square) in the projected British National Grid system (Datum OSGB 1936)	km
Easting	Easting of the bottom left corner of the sampling unit (1-km square) in the projected British National Grid system (Datum OSGB 1936)	km
Year	Year of survey	-

Table S2. Concurvity matrix for each pair of predictors included in the models according to the most pessimistic method (Wood, 2021). In all cases (except for the pair DTR_B_t-1 and DTR_B_t-2) values were low or moderate. See “Materials and methods” and Table S1 for acronyms.

Variable	space_ time	elev	Hab.A	Hab.B	Hab.C	Hab.D	Hab.E	Hab.F	Hab.G	Hab.H	Hab.I	FD0_ W_t_1	FD0_ W_t_2	DTR_ W_t_1	DTR_ W_t_2	SU25_ B_t_1	SU25_ B_t_2	DTR_ B_t_1	DTR_ B_t_2	DD_B _t_1	DD_B _t_2	SDII_ B_t_1	SDII_ B_t_2
space_time	1	0.21	0.02	0.01	0.04	0.22	0.09	0.08	0.01	0.02	0.01	0.19	0.2	0.24	0.22	0.36	0.38	0.42	0.42	0.37	0.35	0.13	0.12
elev	0.21	1	0.02	0.01	0.12	0.25	0.09	0.08	0.01	0.02	0.03	0.29	0.31	0.18	0.18	0.12	0.14	0.09	0.1	0.17	0.18	0.14	0.14
Hab.A	0.02	0.02	1	0.03	0.01	0.02	0.22	0.03	0	0	0	0	0	0.01	0.01	0	0	0.01	0.01	0	0	0.01	0.01
Hab.B	0.01	0.01	0.03	1	0.01	0.01	0.05	0.01	0	0	0	0	0	0	0	0	0	0	0	0	0	0	0
Hab.C	0.04	0.12	0.01	0.01	1	0.04	0.1	0.02	0	0	0.02	0.02	0.03	0.07	0.07	0.03	0.03	0.04	0.04	0.04	0.04	0.05	0.05
Hab.D	0.22	0.25	0.02	0.01	0.04	1	0.15	0.04	0	0	0.02	0.14	0.15	0.1	0.1	0.07	0.08	0.09	0.1	0.11	0.12	0.04	0.04
Hab.E	0.09	0.09	0.22	0.05	0.1	0.15	1	0.24	0.04	0.01	0.01	0.05	0.05	0.06	0.06	0.01	0.02	0.05	0.06	0.04	0.04	0.03	0.04
Hab.F	0.08	0.08	0.03	0.01	0.02	0.04	0.24	1	0.02	0	0	0.04	0.04	0.01	0.01	0.04	0.05	0.01	0.01	0.04	0.04	0.02	0.01
Hab.G	0.01	0.01	0	0	0	0	0.04	0.02	1	0	0	0	0	0	0	0	0.01	0	0	0	0	0	0
Hab.H	0.02	0.02	0	0	0	0	0.01	0	0	1	0	0.03	0.03	0.02	0.02	0	0.01	0.05	0.05	0	0	0	0
Hab.I	0.01	0.03	0	0	0.02	0.02	0.01	0	0	0	1	0.01	0.01	0.03	0.03	0.01	0.01	0.02	0.02	0.01	0.01	0.02	0.02
FD0_W_t_1	0.19	0.29	0	0	0.02	0.14	0.05	0.04	0	0.03	0.01	1	0.35	0.08	0.07	0.04	0.07	0.1	0.17	0.1	0.11	0.02	0.03
FD0_W_t_2	0.2	0.31	0	0	0.03	0.15	0.05	0.04	0	0.03	0.01	0.35	1	0.05	0.07	0.05	0.03	0.15	0.09	0.04	0.11	0.01	0.02
DTR_W_t_1	0.24	0.18	0.01	0	0.07	0.1	0.06	0.01	0	0.02	0.03	0.08	0.05	1	0.47	0.09	0.2	0.45	0.45	0.08	0.07	0.07	0.07
FD0_W_t_2	0.22	0.18	0.01	0	0.07	0.1	0.06	0.01	0	0.02	0.03	0.07	0.07	0.47	1	0.08	0.08	0.38	0.44	0.03	0.08	0.06	0.07
SU25_B_t_1	0.36	0.12	0	0	0.03	0.07	0.01	0.04	0	0	0.01	0.04	0.05	0.09	0.08	1	0.28	0.46	0.12	0.42	0.07	0.09	0.04
SU25_B_t_2	0.38	0.14	0	0	0.03	0.08	0.02	0.05	0.01	0.01	0.01	0.07	0.03	0.2	0.08	0.28	1	0.39	0.43	0.24	0.38	0.06	0.11
DTR_B_t_1	0.42	0.09	0.01	0	0.04	0.09	0.05	0.01	0	0.05	0.02	0.1	0.15	0.45	0.38	0.46	0.39	1	0.71	0.42	0.15	0.11	0.05
DTR_B_t_2	0.42	0.1	0.01	0	0.04	0.1	0.06	0.01	0	0.05	0.02	0.17	0.09	0.45	0.44	0.12	0.43	0.71	1	0.08	0.41	0.05	0.13
DD_B_t_1	0.37	0.17	0	0	0.04	0.11	0.04	0.04	0	0	0.01	0.1	0.04	0.08	0.03	0.42	0.24	0.42	0.08	1	0.16	0.16	0.05
DD_B_t_2	0.35	0.18	0	0	0.04	0.12	0.04	0.04	0	0	0.01	0.11	0.11	0.07	0.08	0.07	0.38	0.15	0.41	0.16	1	0.06	0.18
SDII_B_t_1	0.13	0.14	0.01	0	0.05	0.04	0.03	0.02	0	0	0.02	0.02	0.01	0.07	0.06	0.09	0.06	0.11	0.05	0.16	0.06	1	0.27
SDII_B_t_2	0.12	0.14	0.01	0	0.05	0.04	0.04	0.01	0	0	0.02	0.03	0.02	0.07	0.07	0.04	0.11	0.05	0.13	0.05	0.18	0.27	1

Figure S2. Criterion used for the assignment of “low uncertainty”, “moderate uncertainty” or “high uncertainty” in addition to the main categories describing the type of relationship between bird counts and climate extremes.

On the left, smooth functions with the estimated degree of freedom (edf) and the corresponding level of significance (***: p-value ≤ 0.001; **: 0.001 < p-value ≤ 0.01; *: 0.1; p-value ≤ 0.05) are showed (see Figure 2 in the main text for details on the features of the graphs). On the right, the first derivative with confidence interval of the function is showed, and the red line indicates the zero value where the slope is null. In the case a), the assessment of the effects was straightforward (“low uncertainty”) since the confidence intervals around both the estimated smooth and the first derivative of the smooth function were consistent. In the case b), the relationship was specified as “moderate uncertainty” because the confidence interval of the smooth showed opposite directions for a restricted interval of values of the explanatory variable and the confidence interval for the first derivative included the zero value for that interval. In the case c), along the entire domain of the function, the confidence interval of the smooth showed opposite directions and that of the first derivative always included the zero value.

a) The effect was specified as “low uncertainty” (song thrush *Turdus philomelos*, DD_B_t-1 = Dry days in the breading season at the time *t-1*).

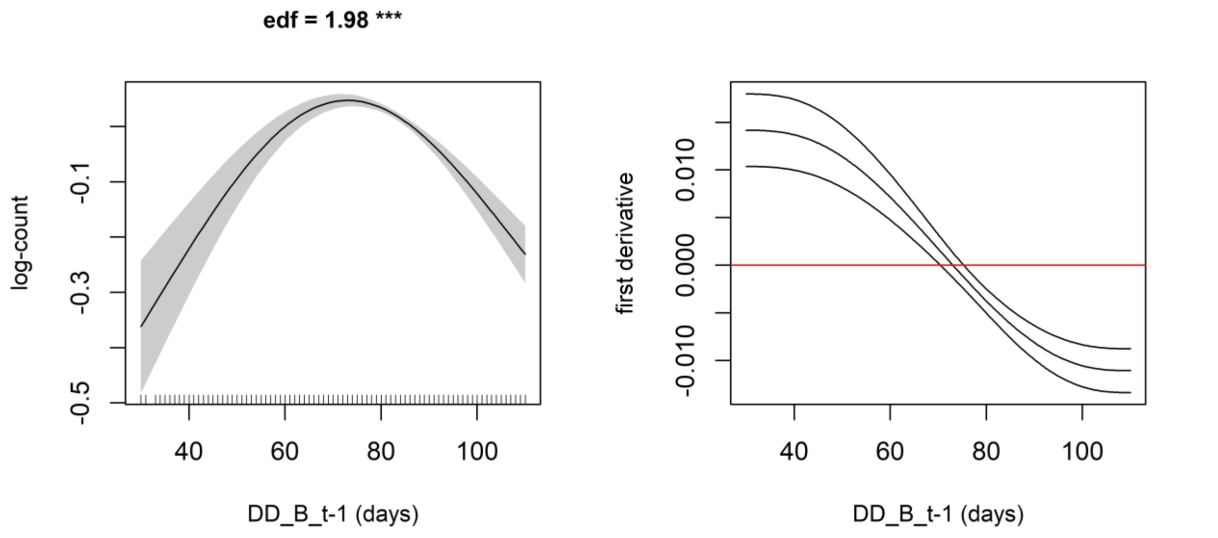

b) The effect was specified as "moderate uncertainty" (Eurasian wren *Troglodytes troglodytes*, DTR_B_t-1 = daily temperature range in the breading season at the time *t-1*)

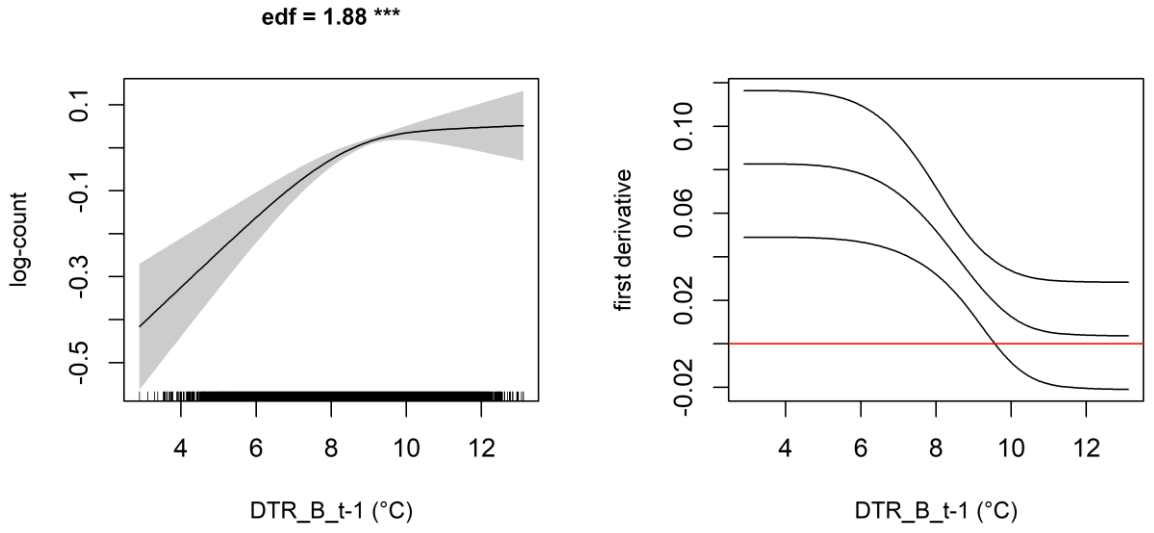

c) The effect was specified as "high uncertainty" (twite *Linaria flavirostris*, DTR_B_t-2 = daily temperature range in the breading season at the time *t-2*).

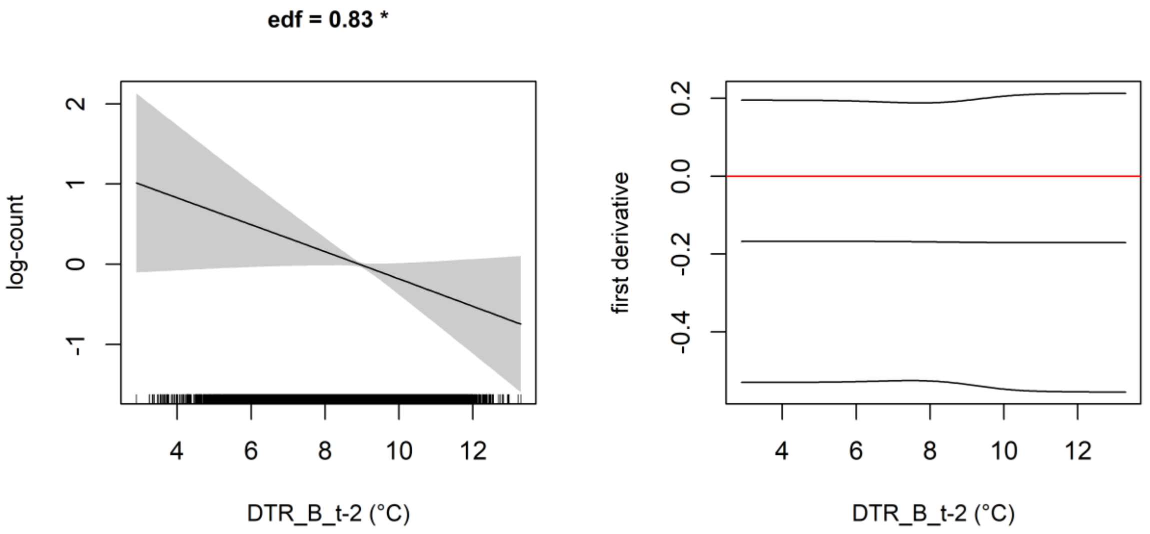

Table S3. AIC comparison between Poisson and negative binomial GAMs.

NB = negative binomial; POIS = Poisson. In the negative binomial GAMs, the variance is a quadratic function of the mean ($\sigma^2 = \mu + \mu^2/\theta$) (Zuur et al., 2009). The smaller the dispersion parameter θ, the larger the overdispersion. Bird species are listed according to the International Ornithological Committee (IOC) World Bird List (Gill et al., 2022) following the taxonomic order.

**Species**	**Model**	**AIC**	$\boldsymbol{\theta}$
Little Grebe	NB-GAM	19,318	0.042
Tachybaptus ruficollis	POIS-GAM	25,214	-
Great Crested Grebe	NB-GAM	20,973	0.031
Podiceps cristatus	POIS-GAM	38,686	-
Great Cormorant	NB-GAM	54,329	0.135
Phalacrocorax carbo	POIS-GAM	80,106	-
Little Egret	NB-GAM	13,243	0.099
Egretta garzetta	POIS-GAM	18,244	-
Grey Heron	NB-GAM	102,683	0.548
Ardea cinerea	POIS-GAM	114,531	-
Mute Swan	NB-GAM	65,444	0.075
Cygnus olor	POIS-GAM	169,388	-
Greylag Goose	NB-GAM	75307	0.044
Anser anser	POIS-GAM	297,005	-
Canada Goose	NB-GAM	132,710	0.084
Branta canadensis	POIS-GAM	468,442	-
Egyptian Goose	NB-GAM	8,305	0.032
Alopochen aegyptiaca	POIS-GAM	16,344	-
Common Shelduck	NB-GAM	41,204	0.065
Tadorna tadorna	POIS-GAM	122,801	-
Mandarin Duck	NB-GAM	10,330	0.017
Aix galericulata	POIS-GAM	16,340	-
Gadwall	NB-GAM	14,423	0.025
Mareca strepera	POIS-GAM	27,997	-
Eurasian Teal	NB-GAM	11,341	0.010
Anas crecca	POIS-GAM	26,371	-
Mallard	NB-GAM	258,548	0.360
Anas platyrhynchos	POIS-GAM	493,862	-
Tufted Duck	NB-GAM	48,722	0.028
Aythya fuligula	POIS-GAM	148,007	-
Common Merganser	NB-GAM	11,972	0.030
Mergus merganser	POIS-GAM	17,813	-
Red Kite	NB-GAM	40,861	0.150
Milvus milvus	POIS-GAM	53,751	-
Western Marsh Harrier	NB-GAM	5,495	0.436
Circus aeruginosus	POIS-GAM	5,743	-
Hen Harrier	NB-GAM	3,741	0.102
Circus cyaneus	POIS-GAM	4,002	-

Northern Goshawk	NB-GAM	2,910	0.283
Accipiter gentilis	POIS-GAM	2,940	-
Eurasian Sparrowhawk	NB-GAM	54,421	1.939
Accipiter nisus	POIS-GAM	54,584	-
Common Buzzard	NB-GAM	141,264	2.226
Buteo buteo	POIS-GAM	145,295	-
Common Kestrel	NB-GAM	87,368	2.926
Falco tinnunculus	POIS-GAM	87,745	-
Merlin	NB-GAM	4,112	0.199
Falco columbarius	POIS-GAM	4,213	-
Peregrine Falcon	NB-GAM	11,954	0.111
Falco peregrinus	POIS-GAM	12,572	-
Willow Ptarmigan	NB-GAM	27,002	0.435
Lagopus lagopus	POIS-GAM	41,289	-
Black Grouse	NB-GAM	3,734	0.033
Lyrurus tetrix	POIS-GAM	6,018	-
Red-legged Partridge	NB-GAM	112,058	0.321
Alectoris rufa	POIS-GAM	149,418	-
Grey Partridge	NB-GAM	48,490	0.122
Perdix perdix	POIS-GAM	64,188	-
Common Pheasant	NB-GAM	293,605	1.032
Phasianus colchicus	POIS-GAM	411,557	
Common Moorhen	NB-GAM	107,452	0.434
Gallinula chloropus	POIS-GAM	125,333	-
Eurasian Coot	NB-GAM	68,608	0.069
Fulica atra	POIS-GAM	153,660	-
Eurasian Oystercatcher	NB-GAM	77,239	0.158
Haematopus ostralegus	POIS-GAM	155,803	-
Common Ringed Plover	NB-GAM	8,386	0.018
Charadrius hiaticula	POIS-GAM	23,071	-
European Golden Plover	NB-GAM	32,407	0.020
Pluvialis apricaria	POIS-GAM	197,996	-
Northern Lapwing	NB-GAM	150,104	0.149
Vanellus vanellus	POIS-GAM	385,827	-
Common Snipe	NB-GAM	33,099	0.210
Gallinago gallinago	POIS-GAM	42,192	-
Eurasian Woodcock	NB-GAM	3,360	0.044
Scolopax rusticola	POIS-GAM	3,562	-
Eurasian Curlew	NB-GAM	104,154	0.226
Numenius arquata	POIS-GAM	179,712	-
Common Redshank	NB-GAM	24,076	0.030
Tringa totanus	POIS-GAM	54,069	-
Feral Pigeon	NB-GAM	183,088	0.110
Columba livia	POIS-GAM	689,152	-
Stock Dove	NB-GAM	158,887	0.310
Columba oenas	POIS-GAM	236,477	-
Common Wood Pigeon	NB-GAM	524,360	1.749
Columba palumbus	POIS-GAM	124,9704	-

Eurasian Collared Dove	NB-GAM	227,306	0.753
Streptopelia decaocto	POIS-GAM	307,907	-
Rose-ringed Parakeet	NB-GAM	17,981	0.359
Psittacula krameri	POIS-GAM	31,866	-
Western Barn Owl	NB-GAM	11,131	0.466
Tyto alba	POIS-GAM	11,276	-
Little Owl	NB-GAM	18,054	0.361
Athene noctua	POIS-GAM	18,362	-
Tawny Owl	NB-GAM	19,720	0.393
Strix aluco	POIS-GAM	19,996	-
Short-eared Owl	NB-GAM	4,265	0.090
Asio flammeus	POIS-GAM	4,664	-
Common Kingfisher	NB-GAM	12,554	0.221
Alcedo atthis	POIS-GAM	12,998	-
European Green Woodpecker	NB-GAM	109,915	1.786
Picus viridis	POIS-GAM	113,137	-
Great Spotted Woodpecker	NB-GAM	145,758	2.083
Dendrocopos major	POIS-GAM	150,021	-
Lesser Spotted Woodpecker	NB-GAM	5,646	0.048
Dryobates minor	POIS-GAM	6,113	-
Eurasian Skylark	NB-GAM	322,014	0.691
Alauda arvensis	POIS-GAM	520,001	-
Meadow Pipit	NB-GAM	185,448	0.348
Anthus pratensis	POIS-GAM	338,906	-
Grey Wagtail	NB-GAM	45,711	0.166
Motacilla cinerea	POIS-GAM	51,817	-
White Wagtail	NB-GAM	176,680	1.010
Motacilla alba	POIS-GAM	192,952	-
White-throated Dipper	NB-GAM	13,359	0.167
Cinclus cinclus	POIS-GAM	14,279	-
Eurasian Wren	NB-GAM	408,065	2.140
Troglodytes troglodytes	POIS-GAM	525,328	-
Dunnock	NB-GAM	289,119	2.010
Prunella modularis	POIS-GAM	326,508	-
European Robin	NB-GAM	366,071	2.692
Erithacus rubecula	POIS-GAM	436,402	-
European Stonechat	NB-GAM	34,746	0.167
Saxicola rubicola	POIS-GAM	44,675	-
Common Blackbird	NB-GAM	419,651	3.323
Turdus merula	POIS-GAM	537,834	-
Fieldfare	NB-GAM	16,189	0.004
Turdus pilaris	POIS-GAM	207,284	-
Song Thrush	NB-GAM	269,617	2.183
Turdus philomelos	POIS-GAM	294,758	-
Redwing	NB-GAM	4,981	0.002
Turdus iliacus	POIS-GAM	27,780	-
Mistle Thrush	NB-GAM	170,635	0.685
Turdus viscivorus	POIS-GAM	195,102	-

Cetti's Warbler	NB-GAM	7,694	0.119
Cettia cetti	POIS-GAM	9,523	-
Goldcrest	NB-GAM	141,265	0.498
Regulus regulus	POIS-GAM	177,541	-
Long-tailed Tit	NB-GAM	178,589	0.406
Aegithalos caudatus	POIS-GAM	241,179	-
Marsh Tit	NB-GAM	30,988	0.161
Poecile palustris	POIS-GAM	36,197	-
Willow Tit	NB-GAM	11,864	0.040
Poecile montanus	POIS-GAM	14,646	-
Coal Tit	NB-GAM	146,565	0.557
Periparus ater	POIS-GAM	183,423	-
Eurasian Blue Tit	NB-GAM	383,630	1.978
Cyanistes caeruleus	POIS-GAM	512,345	-
Great Tit	NB-GAM	325,694	2.137
Parus major	POIS-GAM	384,297	-
Eurasian Nuthatch	NB-GAM	92,844	0.523
Sitta europaea	POIS-GAM	106,637	-
Eurasian Treecreeper	NB-GAM	64,959	0.418
Certhia familiaris	POIS-GAM	70,455	-
Eurasian Jay	NB-GAM	117,423	0.957
Garrulus glandarius	POIS-GAM	124,645	-
Eurasian Magpie	NB-GAM	291,250	1.444
Pica pica	POIS-GAM	357,679	
Western Jackdaw	NB-GAM	401,354	0.409
Coloeus monedula	POIS-GAM	1,180,042	-
Rook	NB-GAM	356,882	0.185
Corvus frugilegus	POIS-GAM	2,153,213	-
Carrion Crow	NB-GAM	436,559	1.207
Corvus corone	POIS-GAM	854,899	-
Hooded Crow	NB-GAM	24,395	0.733
Corvus cornix	POIS-GAM	31,770	-
Northern Raven	NB-GAM	64,714	0.282
Corvus corax	POIS-GAM	84,583	-
Common Starling	NB-GAM	401,004	0.363
Sturnus vulgaris	POIS-GAM	1,466,556	-
House Sparrow	NB-GAM	360,725	0.475
Passer domesticus	POIS-GAM	869,844	-
Eurasian Tree Sparrow	NB-GAM	51,610	0.071
Passer montanus	POIS-GAM	101,802	-
Common Chaffinch	NB-GAM	422,530	2.479
Fringilla coelebs	POIS-GAM	551,913	-
European Greenfinch	NB-GAM	270,762	0.902
Chloris chloris	POIS-GAM	366,628	-
European Goldfinch	NB-GAM	298,947	0.882
Carduelis carduelis	POIS-GAM	414,138	-
Eurasian Siskin	NB-GAM	49,396	0.087
Spinus spinus	POIS-GAM	88,843	-

Common Linnet	NB-GAM	245,630	0.329
Linaria cannabina	POIS-GAM	464,910	-
Twite	NB-GAM	5,910	0.021
Linaria flavirostris	POIS-GAM	10,533	-
Lesser Redpoll	NB-GAM	42,279	0.066
Acanthis cabaret	POIS-GAM	80,791	-
Red Crossbill	NB-GAM	17,134	0.033
Loxia curvirostra	POIS-GAM	42,327	-
Eurasian Bullfinch	NB-GAM	113,174	0.411
Pyrrhula pyrrhula	POIS-GAM	128,298	-
Yellowhammer	NB-GAM	208,303	0.669
Emberiza citrinella	POIS-GAM	273,161	-
Common Reed Bunting	NB-GAM	99,024	0.272
Emberiza schoeniclus	POIS-GAM	128,530	-
Corn Bunting	NB-GAM	38,818	0.055
Emberiza calandra	POIS-GAM	74,884	-
Indian Peafowl	NB-GAM	8,934	0.012
Pavo cristatus	POIS-GAM	15,487	-

Table S4. Explained deviance (%) for negative binomial GAMs.

Bird species are listed according to the International Ornithological Committee (IOC) World Bird List (Gill et al., 2022) following the taxonomic order.

**Species**	**Explained deviance (%)**
Little Grebe<br>*Tachybaptus ruficollis*	19.65
Great Crested Grebe<br>*Podiceps cristatus*	36.34
Great Cormorant<br>*Phalacrocorax carbo*	31.02
Little Egret<br>*Egretta garzetta*	58.49
Grey Heron<br>*Ardea cinerea*	14.98
Mute Swan<br>*Cygnus olor*	38.47
Greylag Goose<br>*Anser anser*	22.70
Canada Goose<br>*Branta canadensis*	15.47
Egyptian Goose<br>*Alopochen aegyptiaca*	65.45
Common Shelduck<br>*Tadorna tadorna*	55.91
Mandarin Duck<br>*Aix galericulata*	32.77
Gadwall<br>*Mareca strepera*	45.25
Eurasian Teal<br>*Anas crecca*	30.06
Mallard<br>*Anas platyrhynchos*	17.46
Tufted Duck<br>*Aythya fuligula*	21.27
Common Merganser<br>*Mergus merganser*	36.74
Red Kite<br>*Milvus milvus*	38.01
Western Marsh Harrier<br>*Circus aeruginosus*	59.61
Hen Harrier<br>*Circus cyaneus*	42.07
Northern Goshawk<br>*Accipiter gentilis*	32.73
Eurasian Sparrowhawk<br>*Accipiter nisus*	4.33

Common Buzzard *Buteo buteo*	30.18
Common Kestrel *Falco tinnunculus*	9.99
Merlin *Falco columbarius*	36.50
Peregrine Falcon *Falco peregrinus*	17.63
Willow Ptarmigan *Lagopus lagopus*	85.88
Black Grouse *Lyrurus tetrix*	72.80
Red-legged Partridge *Alectoris rufa*	38.40
Grey Partridge *Perdix perdix*	32.49
Common Pheasant *Phasianus colchicus*	31.48
Common Moorhen *Gallinula chloropus*	25.41
Eurasian Coot *Fulica atra*	28.53
Eurasian Oystercatcher *Haematopus ostralegus*	50.00
Common Ringed Plover *Charadrius hiaticula*	64.67
European Golden Plover *Pluvialis apricaria*	38.14
Northern Lapwing *Vanellus vanellus*	27.60
Common Snipe *Gallinago gallinago*	52.19
Eurasian Woodcock *Scolopax rusticola*	26.59
Eurasian Curlew *Numenius arquata*	45.57
Common Redshank *Tringa totanus*	36.87
Feral Pigeon *Columba livia*	25.83
Stock Dove *Columba oenas*	20.93
Common Wood Pigeon *Columba palumbus*	45.55
Eurasian Collared Dove *Streptopelia decaocto*	40.51
Rose-ringed Parakeet *Psittacula krameri*	88.42
Western Barn Owl *Tyto alba*	26.95
Little Owl *Athene noctua*	18.96

Tawny Owl<br>*Strix aluco*	12.05
Short-eared Owl<br>*Asio flammeus*	46.21
Common Kingfisher<br>*Alcedo atthis*	25.46
European Green Woodpecker<br>*Picus viridis*	36.77
Great Spotted Woodpecker<br>*Dendrocopos major*	29.85
Lesser Spotted Woodpecker<br>*Dryobates minor*	22.75
Eurasian Skylark<br>*Alauda arvensis*	26.43
Meadow Pipit<br>*Anthus pratensis*	56.90
Grey Wagtail<br>*Motacilla cinerea*	13.91
White Wagtail<br>*Motacilla alba*	9.72
White-throated Dipper<br>*Cinclus cinclus*	35.45
Eurasian Wren<br>*Troglodytes troglodytes*	26.34
Dunnock<br>*Prunella modularis*	26.77
European Robin<br>*Erithacus rubecula*	37.86
European Stonechat<br>*Saxicola rubicola*	48.30
Common Blackbird<br>*Turdus merula*	50.04
Fieldfare<br>*Turdus pilaris*	19.30
Song Thrush<br>*Turdus philomelos*	24.83
Redwing<br>*Turdus iliacus*	24.04
Mistle Thrush<br>*Turdus viscivorus*	9.52
Cetti's Warbler<br>*Cettia cetti*	64.02
Goldcrest<br>*Regulus regulus*	32.43
Long-tailed Tit<br>*Aegithalos caudatus*	17.96
Marsh Tit<br>*Poecile palustris*	33.53
Willow Tit<br>*Poecile montanus*	27.39
Coal Tit<br>*Periparus ater*	36.81

Eurasian Blue Tit *Cyanistes caeruleus*	38.39
Great Tit *Parus major*	33.17
Eurasian Nuthatch *Sitta europaea*	39.00
Eurasian Treecreeper *Certhia familiaris*	21.14
Eurasian Jay *Garrulus glandarius*	24.21
Eurasian Magpie *Pica pica*	37.42
Western Jackdaw *Coloeus monedula*	13.55
Rook *Corvus frugilegus*	13.03
Carrion Crow *Corvus corone*	24.07
Hooded Crow *Corvus cornix*	86.88
Northern Raven *Corvus corax*	42.21
Common Starling *Sturnus vulgaris*	25.37
House Sparrow *Passer domesticus*	35.37
Eurasian Tree Sparrow *Passer montanus*	38.43
Common Chaffinch *Fringilla coelebs*	30.44
European Greenfinch *Chloris chloris*	34.74
European Goldfinch *Carduelis carduelis*	26.36
Eurasian Siskin *Spinus spinus*	43.01
Common Linnet *Linaria cannabina*	17.98
Twite *Linaria flavirostris*	63.33
Lesser Redpoll *Acanthis cabaret*	36.82
Red Crossbill *Loxia curvirostra*	57.48
Eurasian Bullfinch *Pyrrhula pyrrhula*	13.50
Yellowhammer *Emberiza citrinella*	38.84
Common Reed Bunting *Emberiza schoeniclus*	27.13
Corn Bunting *Emberiza calandra*	36.83

Indian Peafowl<br>*Pavo cristatus*	24.95

Appendix S1. Effects of the climate extreme indices (CLEXs) on bird relative abundance (counts). For each of the 100 species, partial effect plots describe the relationship between the expected count (y-axis, log-scale with the smooth function centred around zero) and each climate extreme index (x-axis). Indices of climate extremes: FD0 = frost days; DTR = daily temperature range; SU25 = summer days; SDII = simple precipitation intensity index; DD = dry days. After the variable name, "W" indicates the winter season, and "B" the breading season. Then, *t-1* indicates the association between counts in the year *t* and the weather variable of the preceding year, while *t-2* the association with the two-previous year (see "Materials and methods" in the main text). Rugs on the x-axis represent the distribution of values of the variable. The estimated degree of freedom (edf) and the statistical significance (***: p-value ≤ 0.001; **: 0.001 < p-value ≤ 0.01; *: 0.01 < p-value ≤ 0.05; n.s.: p-value > 0.05) for the smooths are shown on the top of the graph. The grey area represents the 95% confidence interval of the regression line.

Little Grebe *Tachybaptus ruficollis*

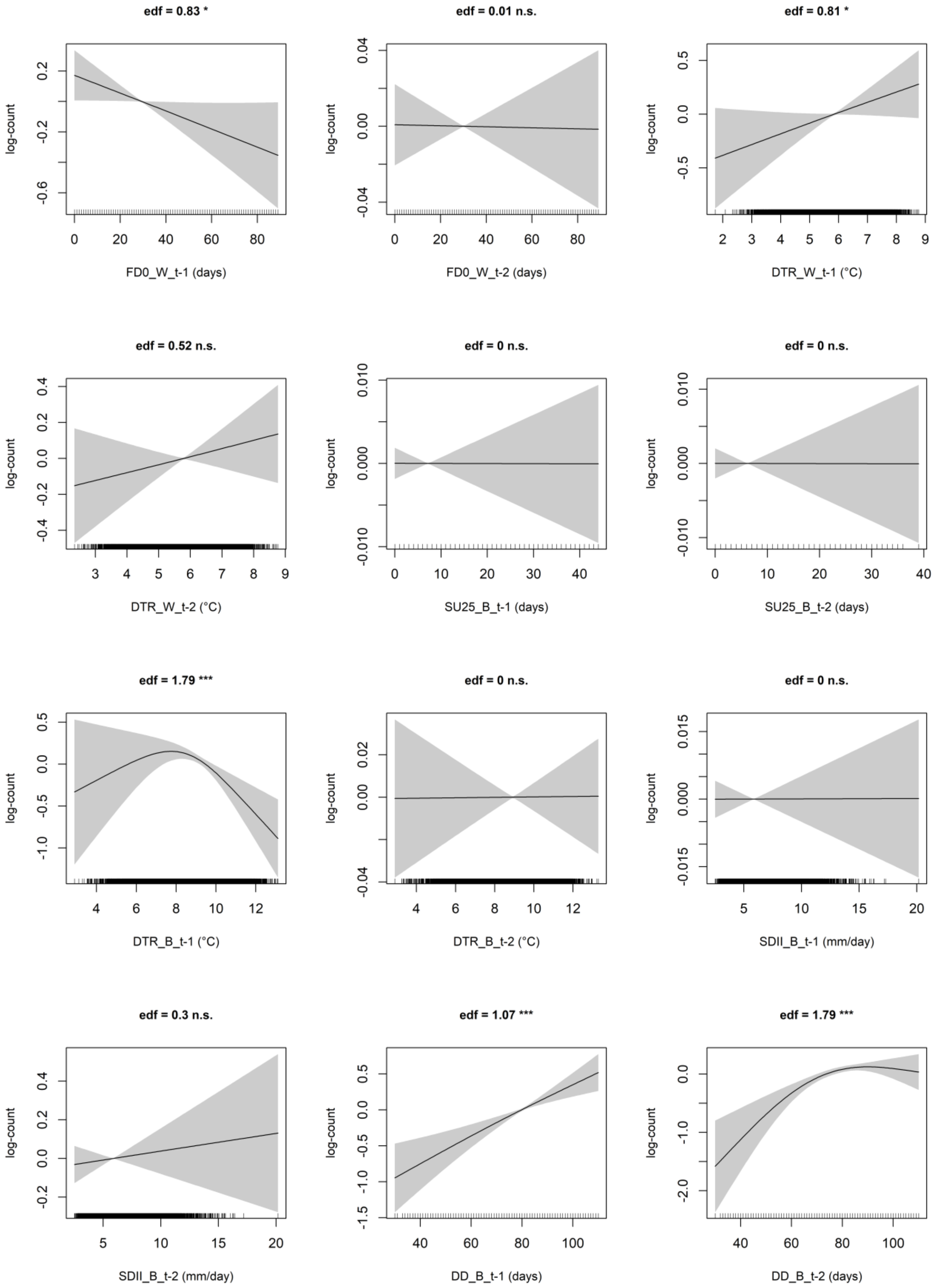

Great Crested Grebe *Podiceps cristatus*

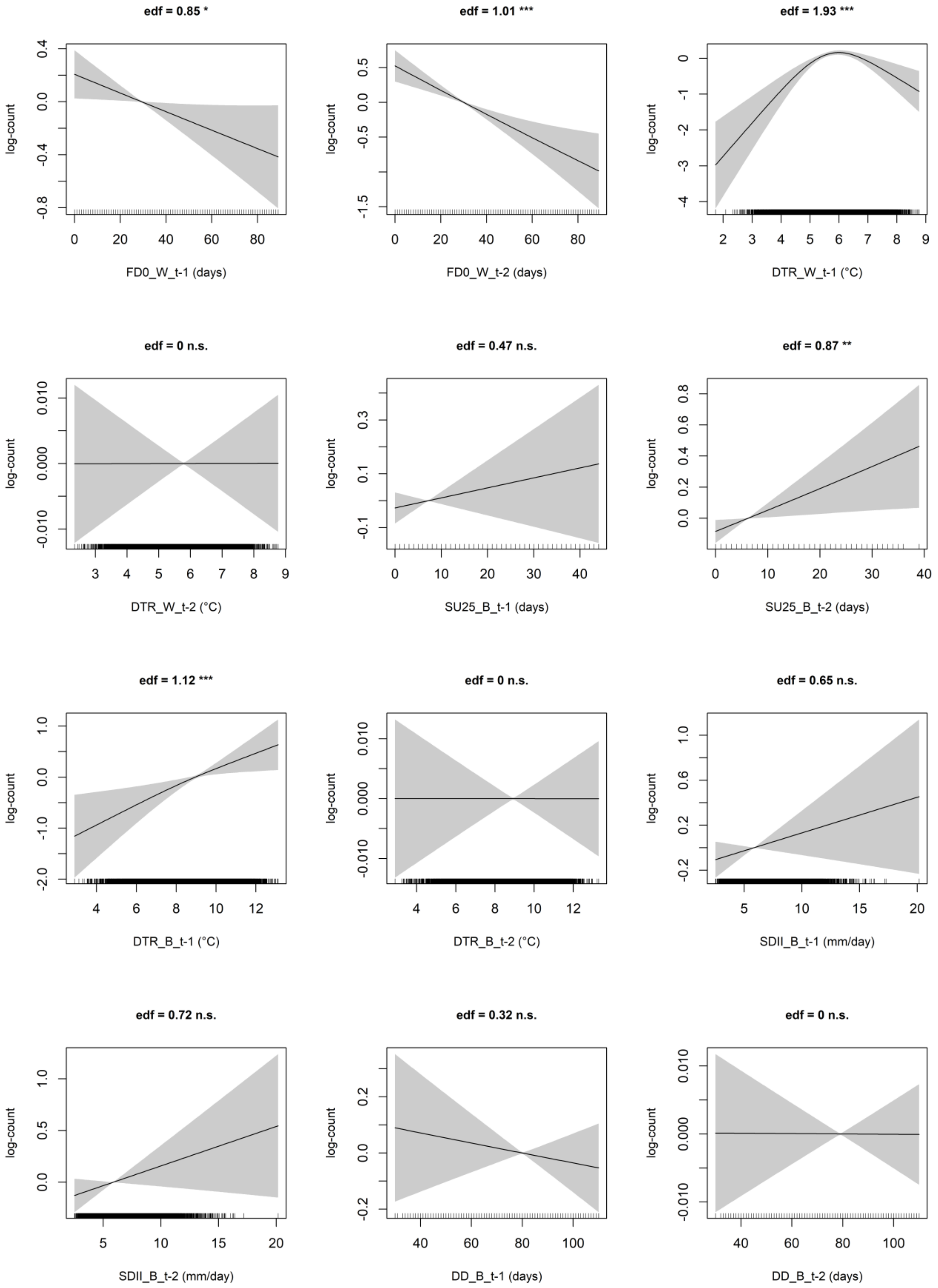

Great Cormorant *Phalacrocorax carbo*

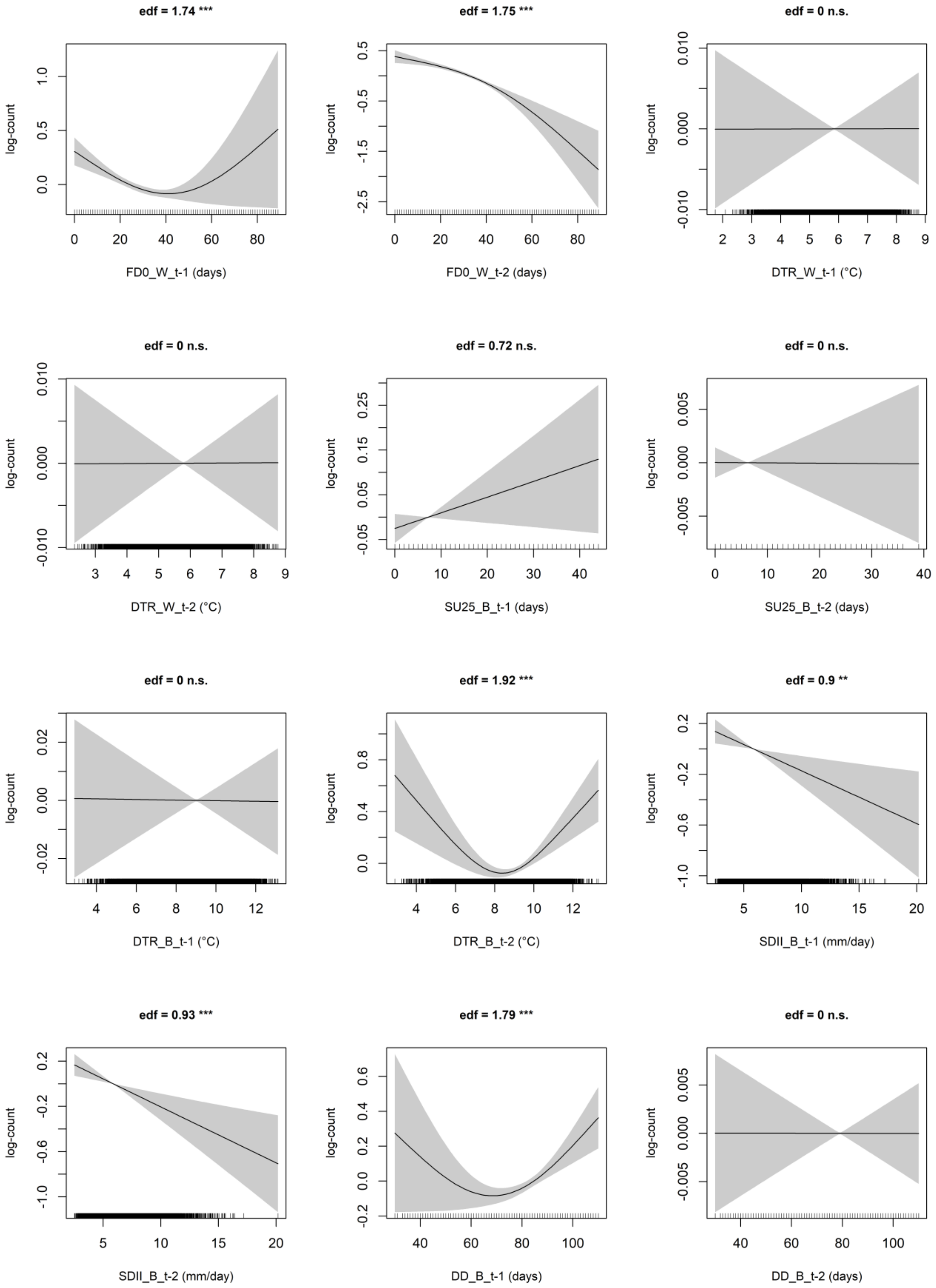

Little Egret *Egretta garzetta*

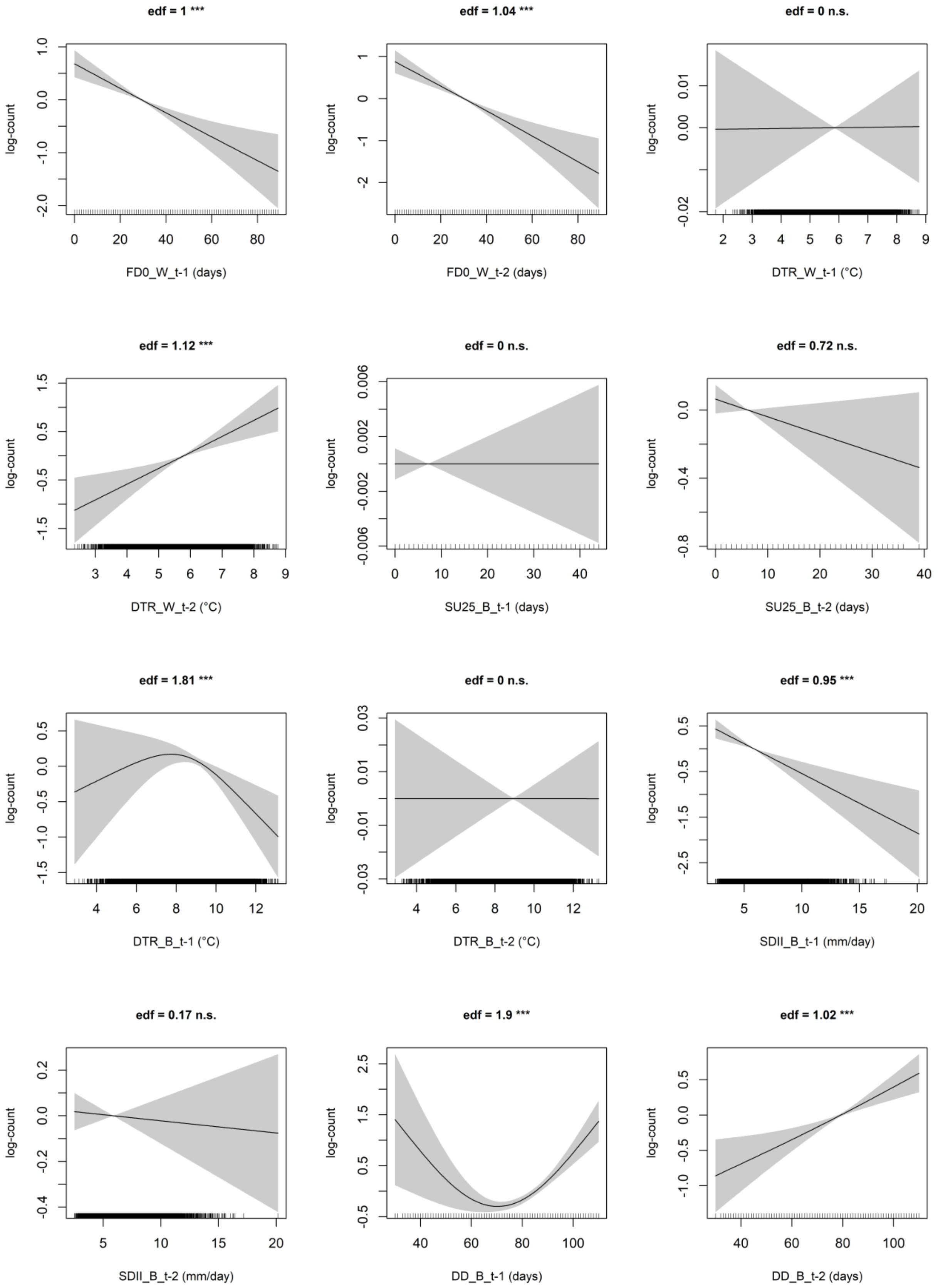

Grey Heron *Ardea cinerea*

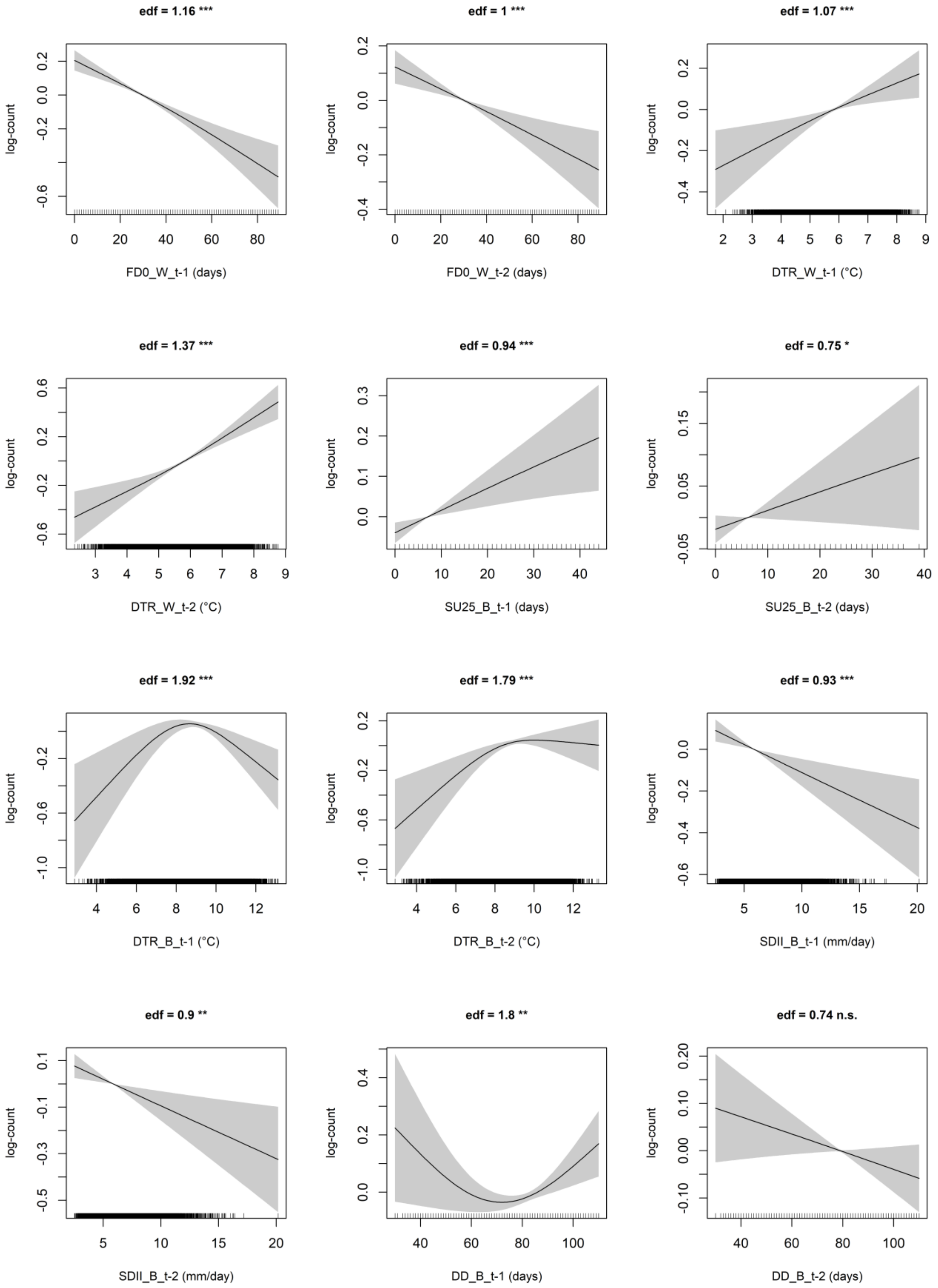

Mute Swan *Cygnus olor*

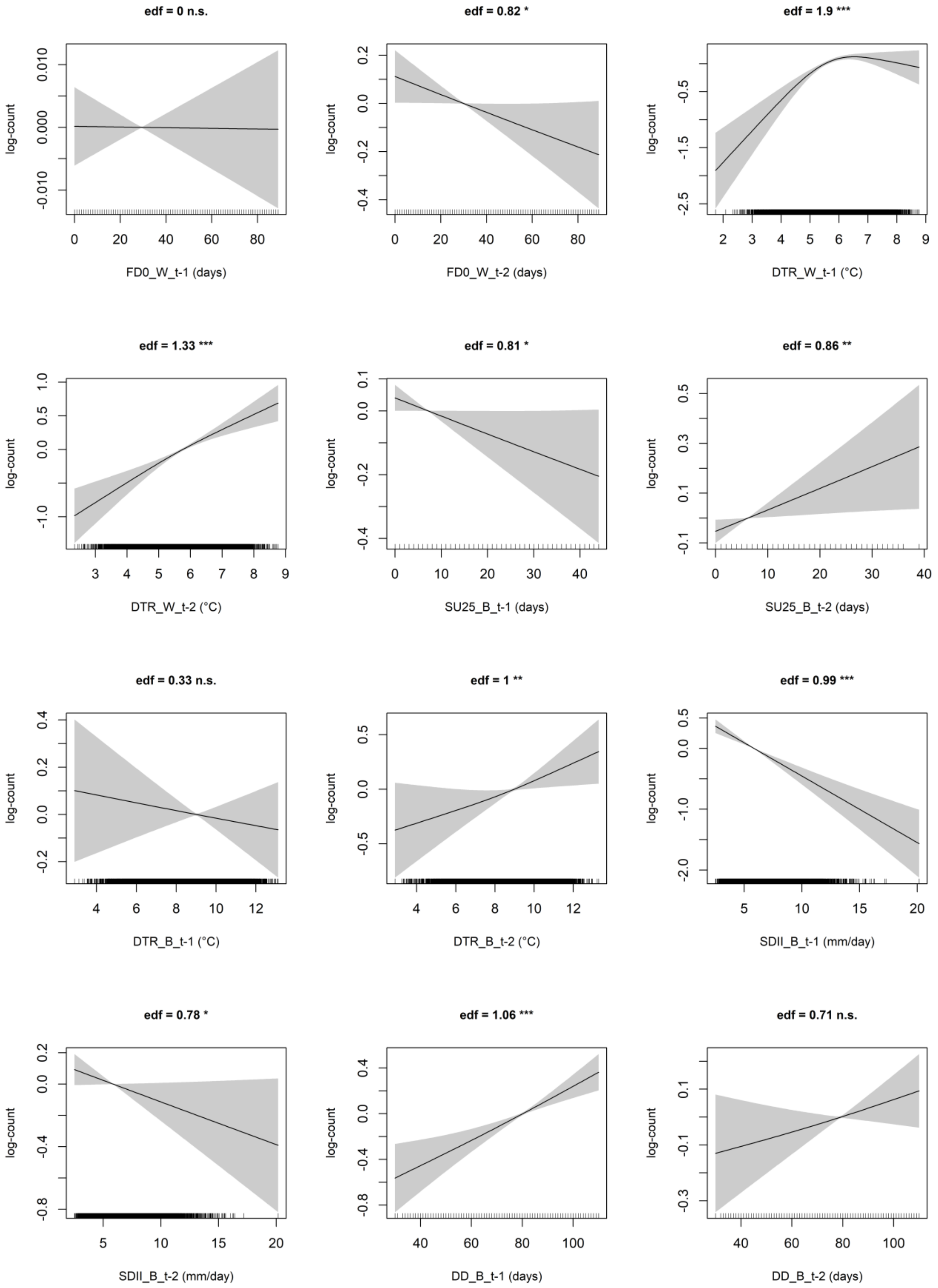

Greylag Goose *Anser anser*

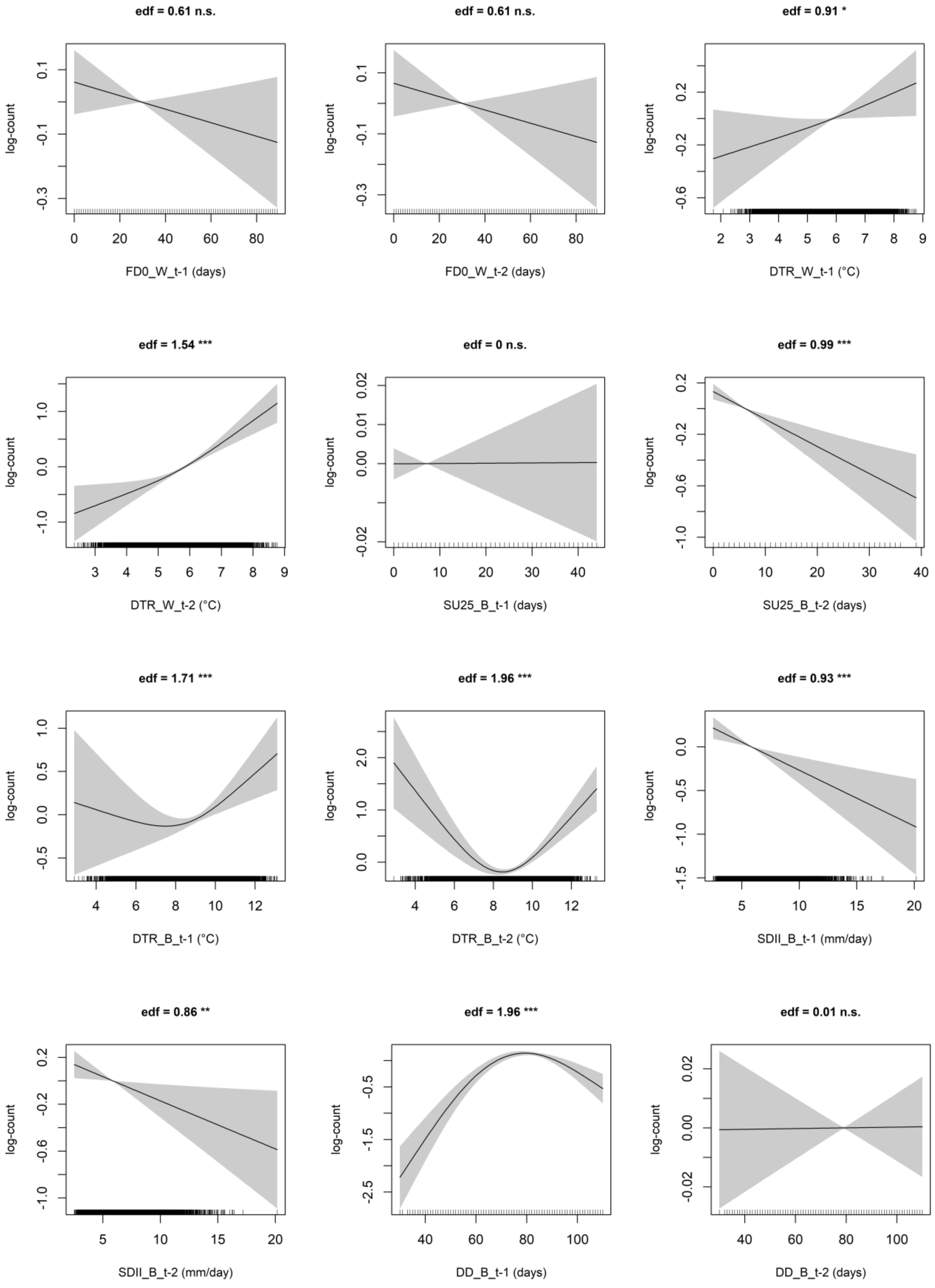

Canada Goose *Branta canadensis*

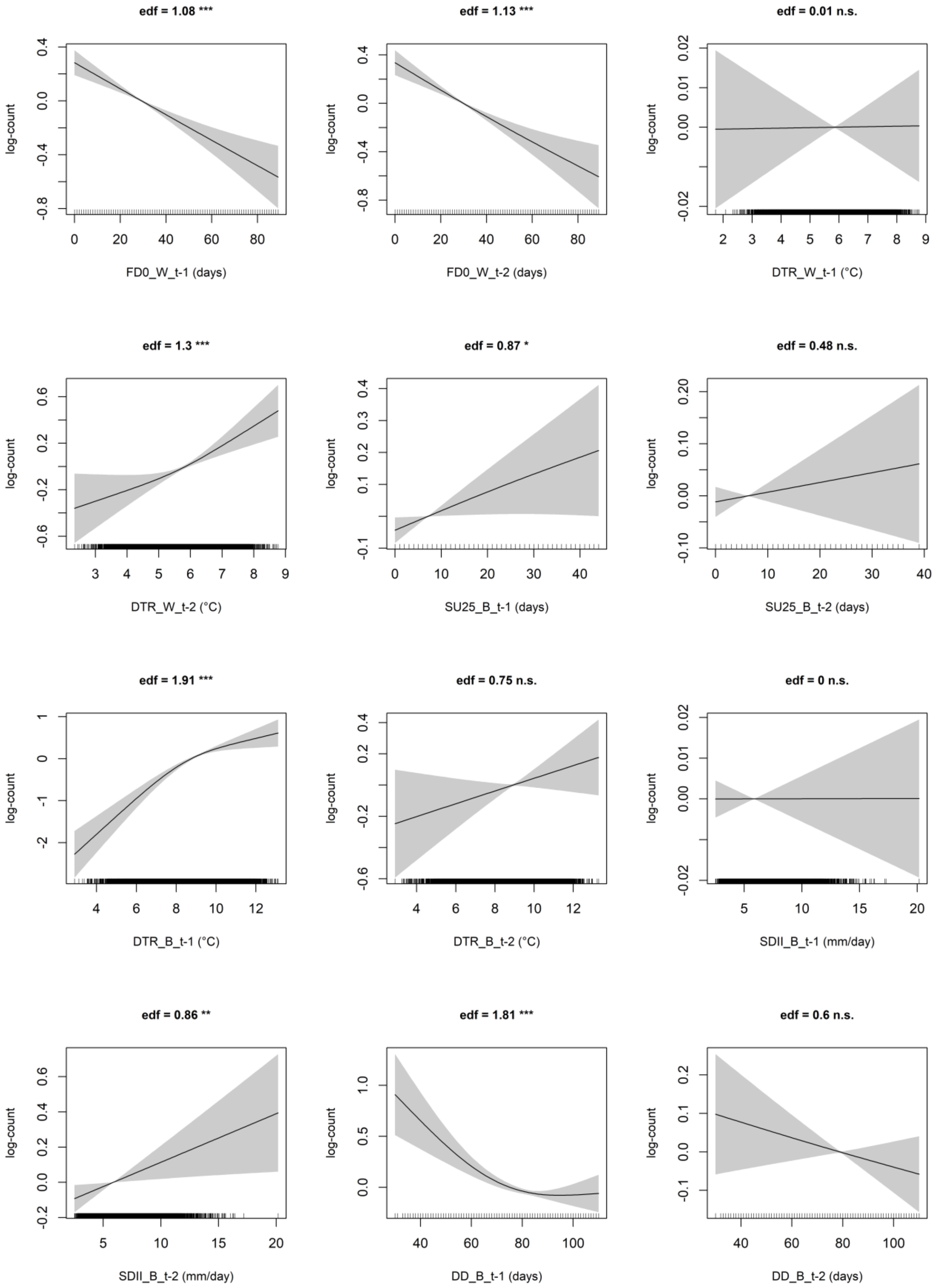

Egyptian Goose *Alopochen aegyptiaca*

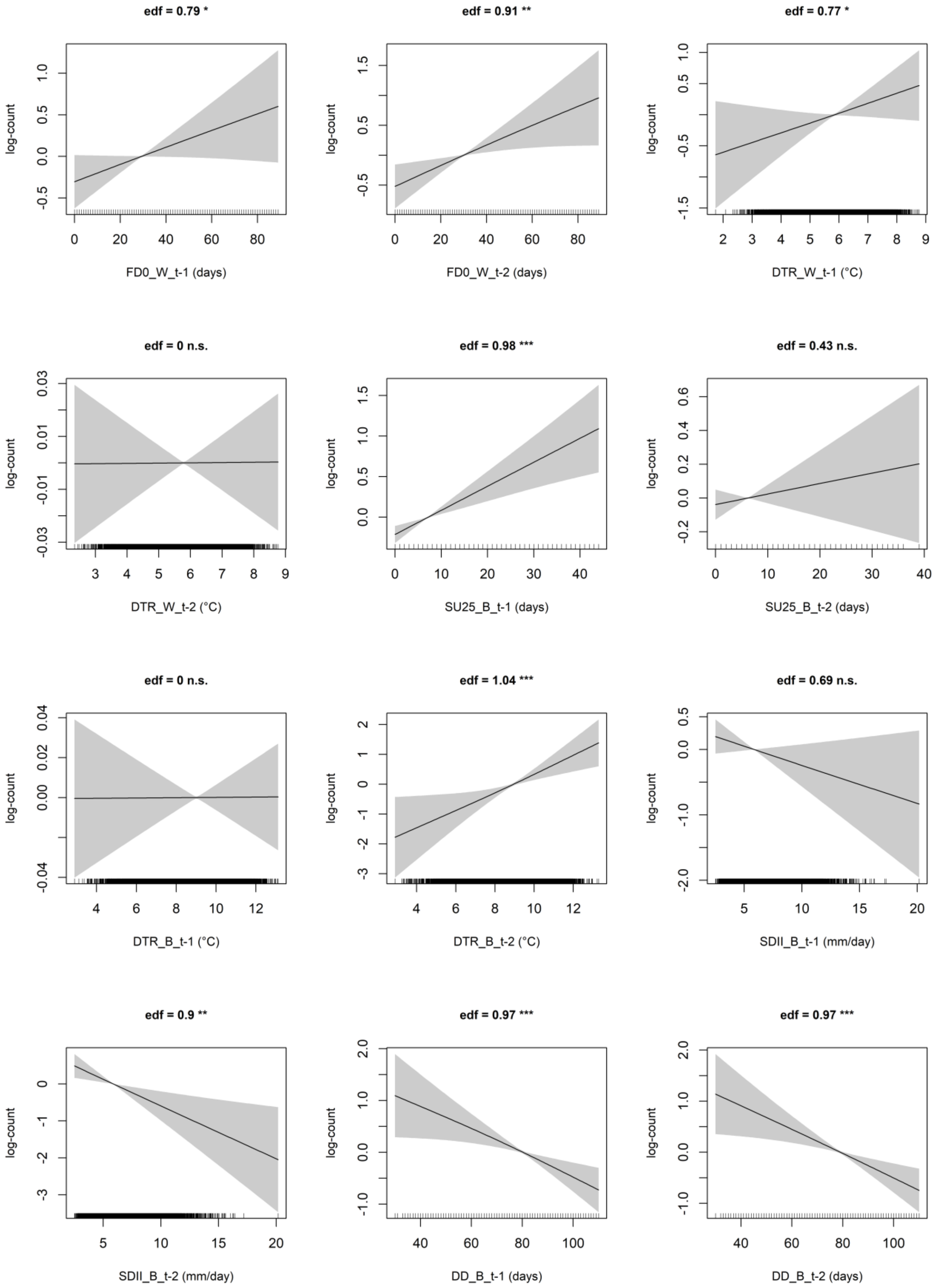

Common Shelduck *Tadorna tadorna*

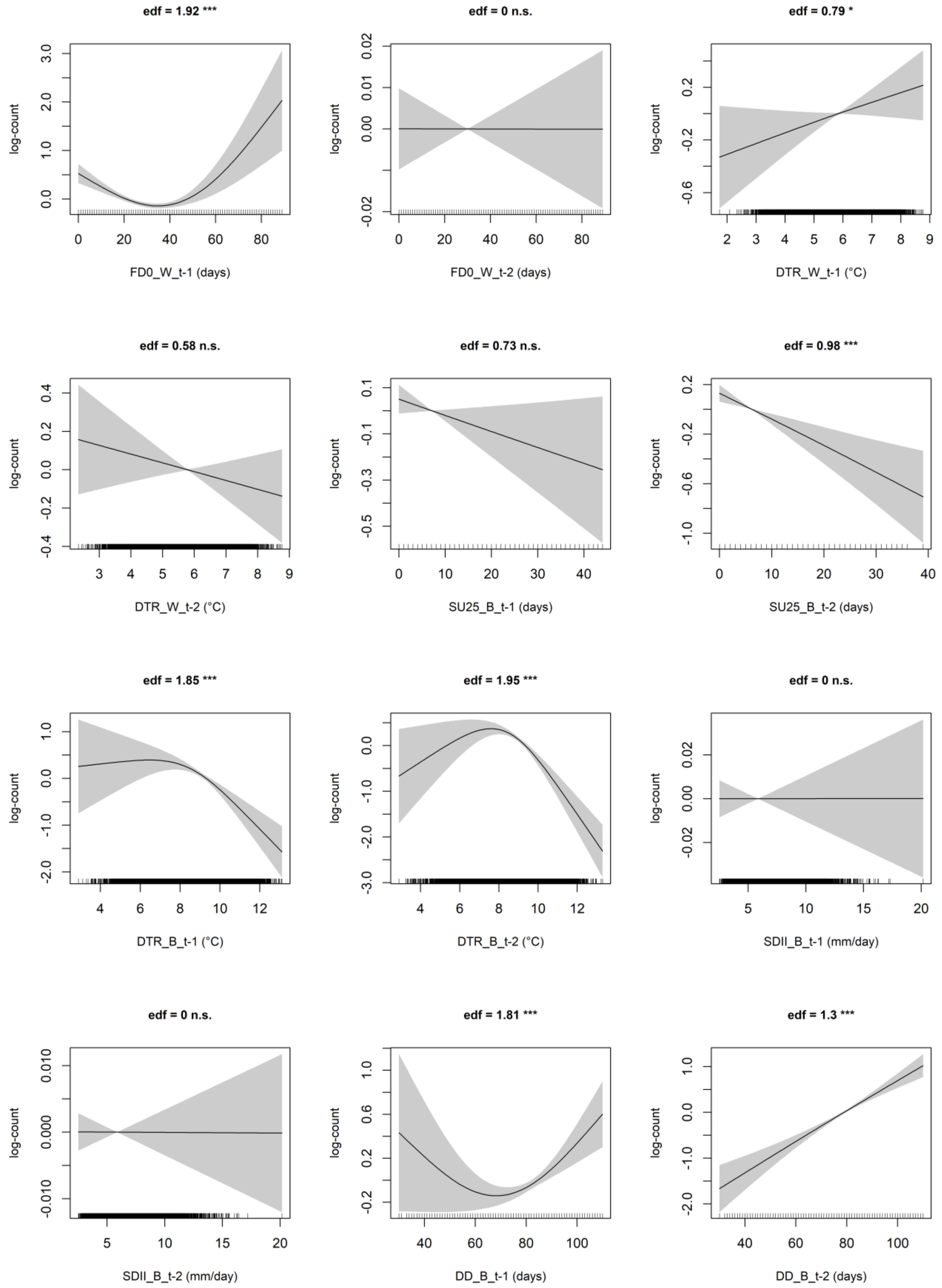

Mandarin Duck *Aix galericulata*

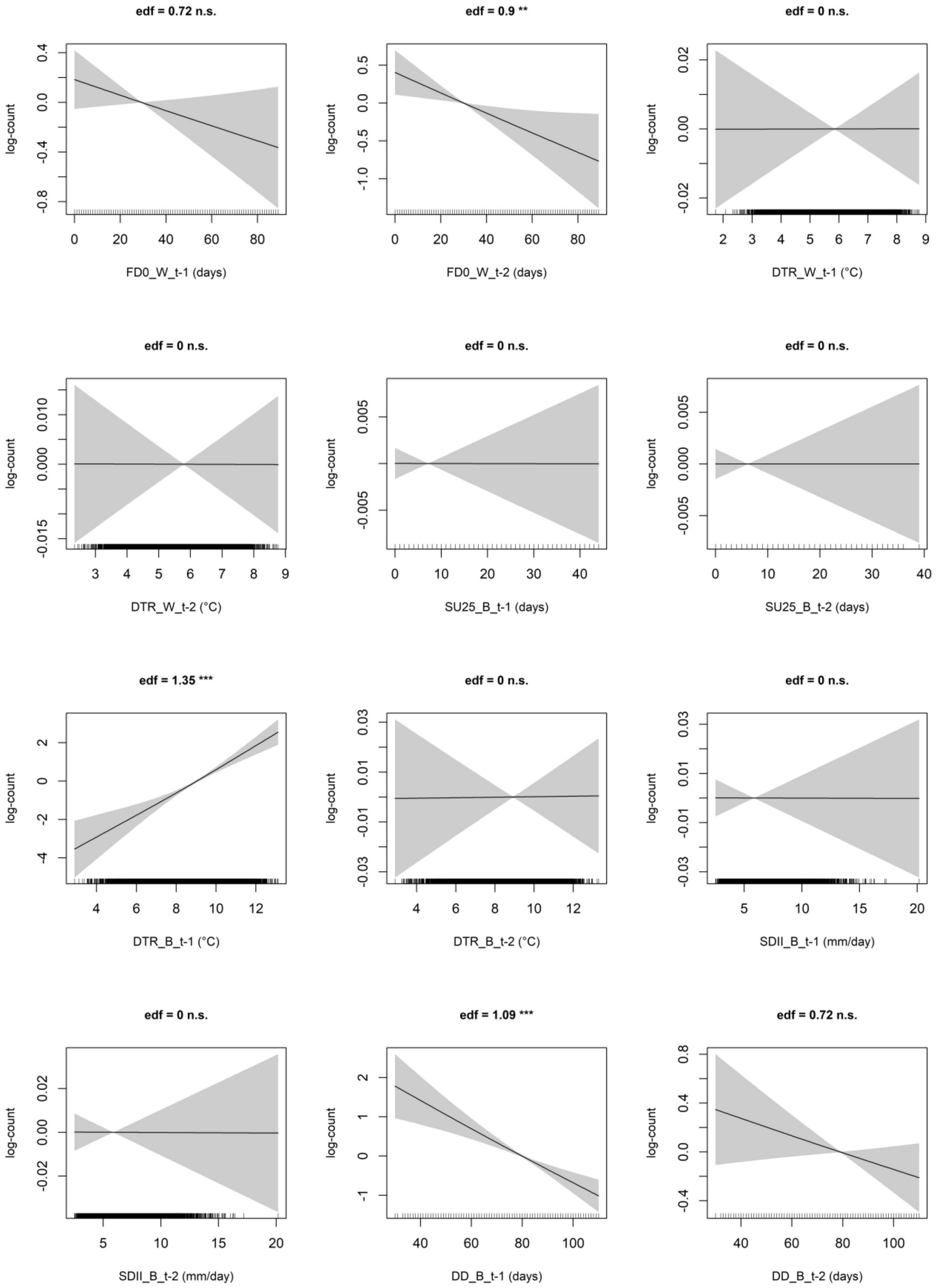

Gadwall *Mareca strepera*

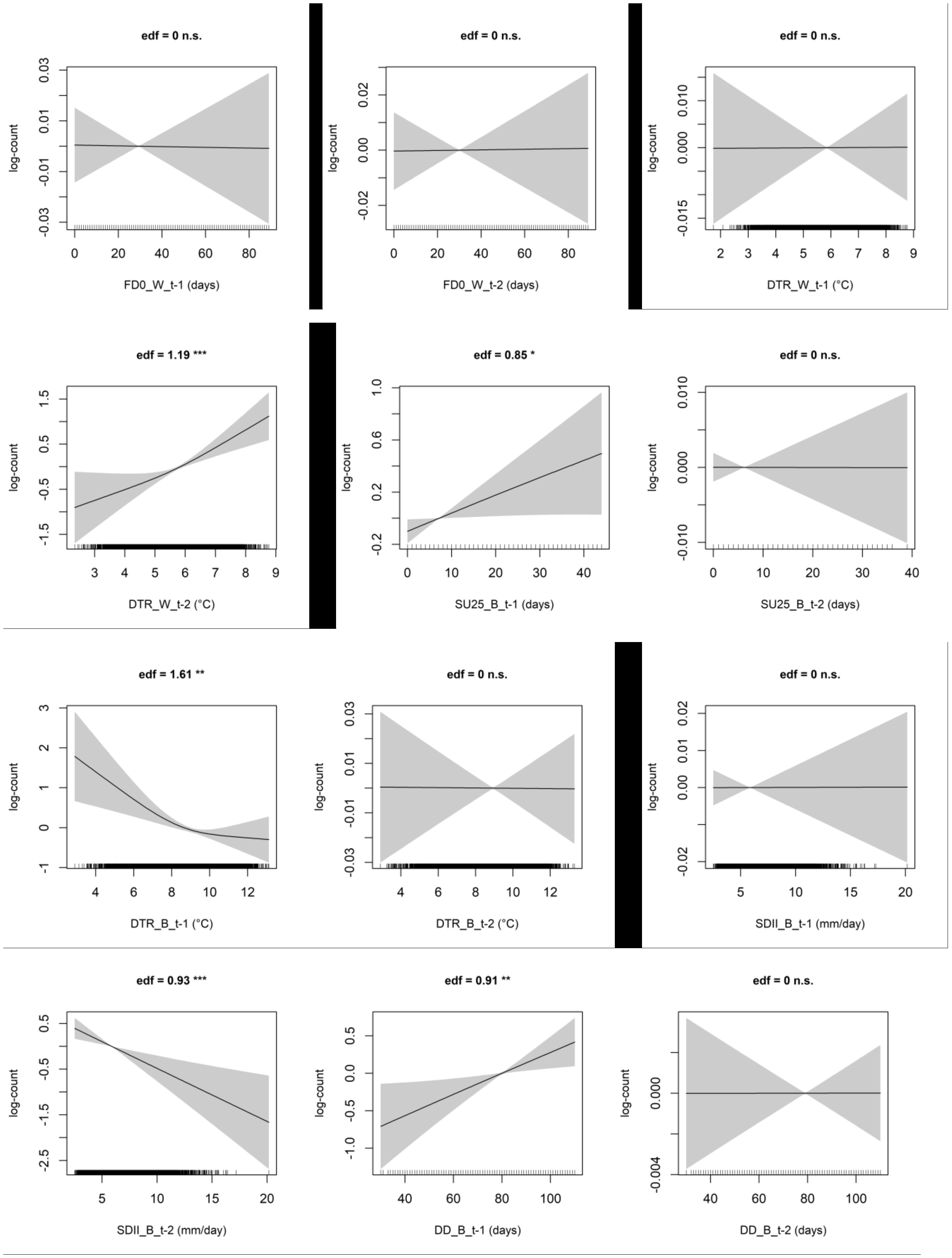

Eurasian Teal *Anas crecca*

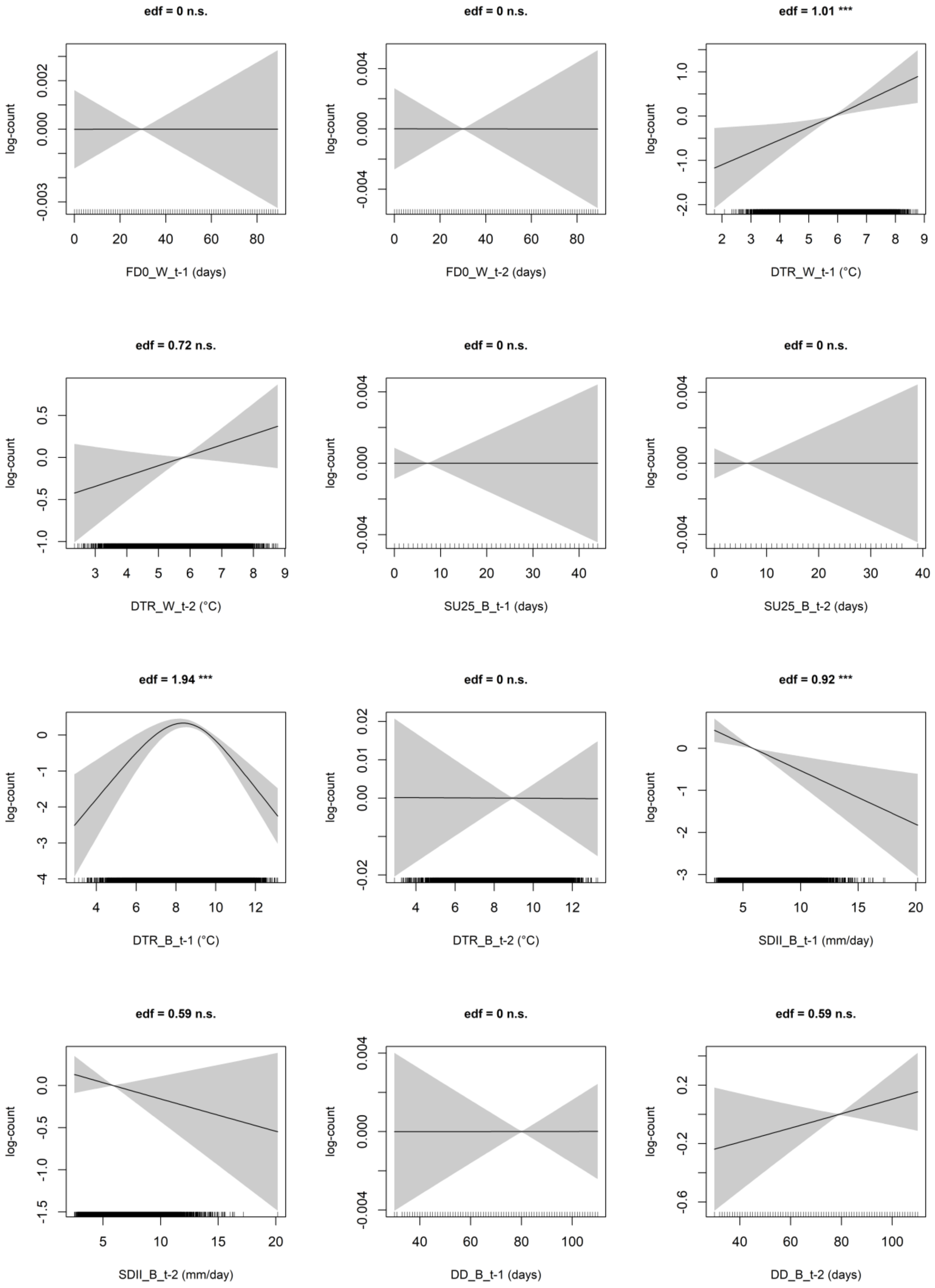

Mallard *Anas platyrhynchos*

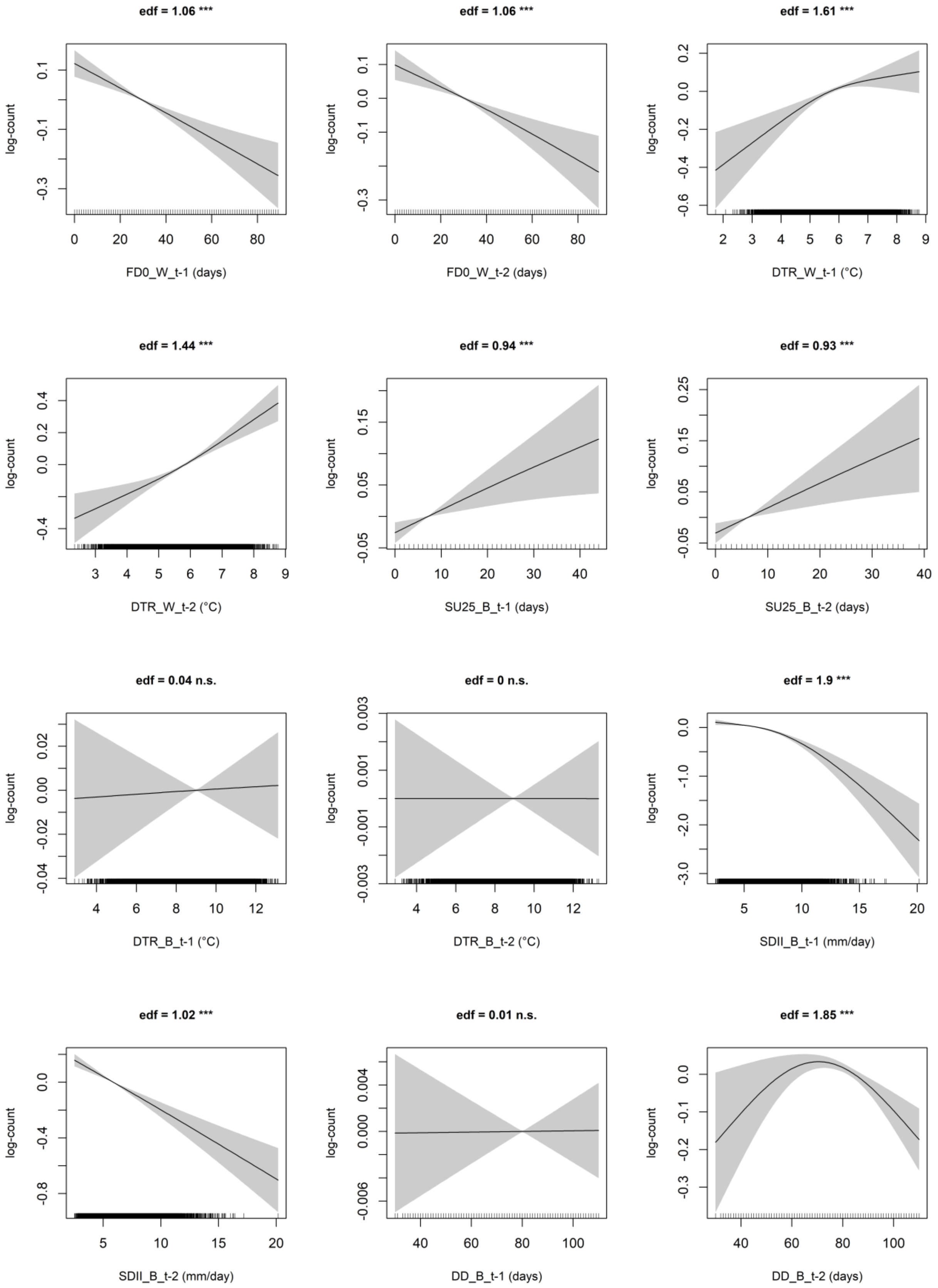

Tufted Duck *Aythya fuligula*

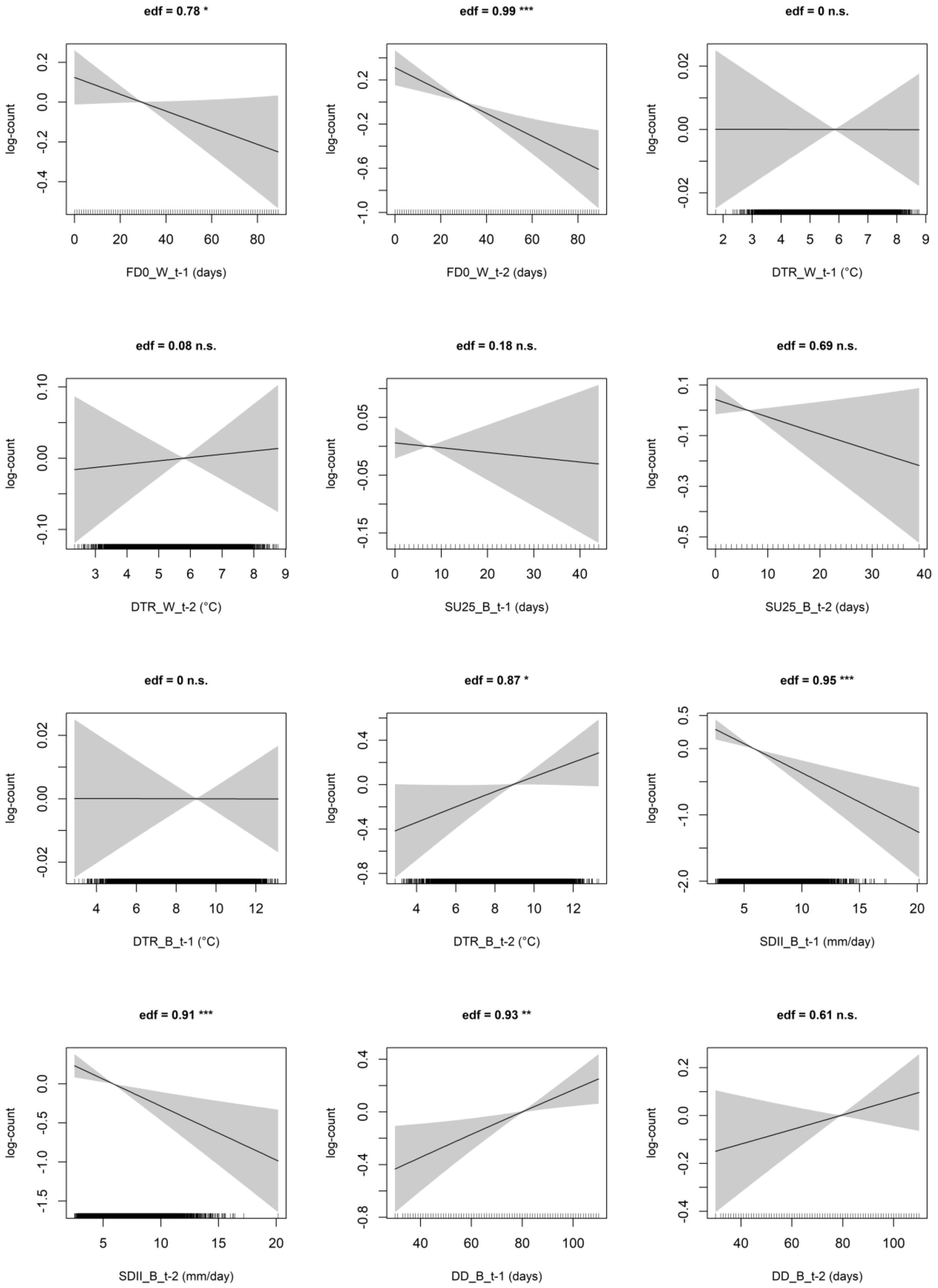

Common Merganser *Mergus merganser*

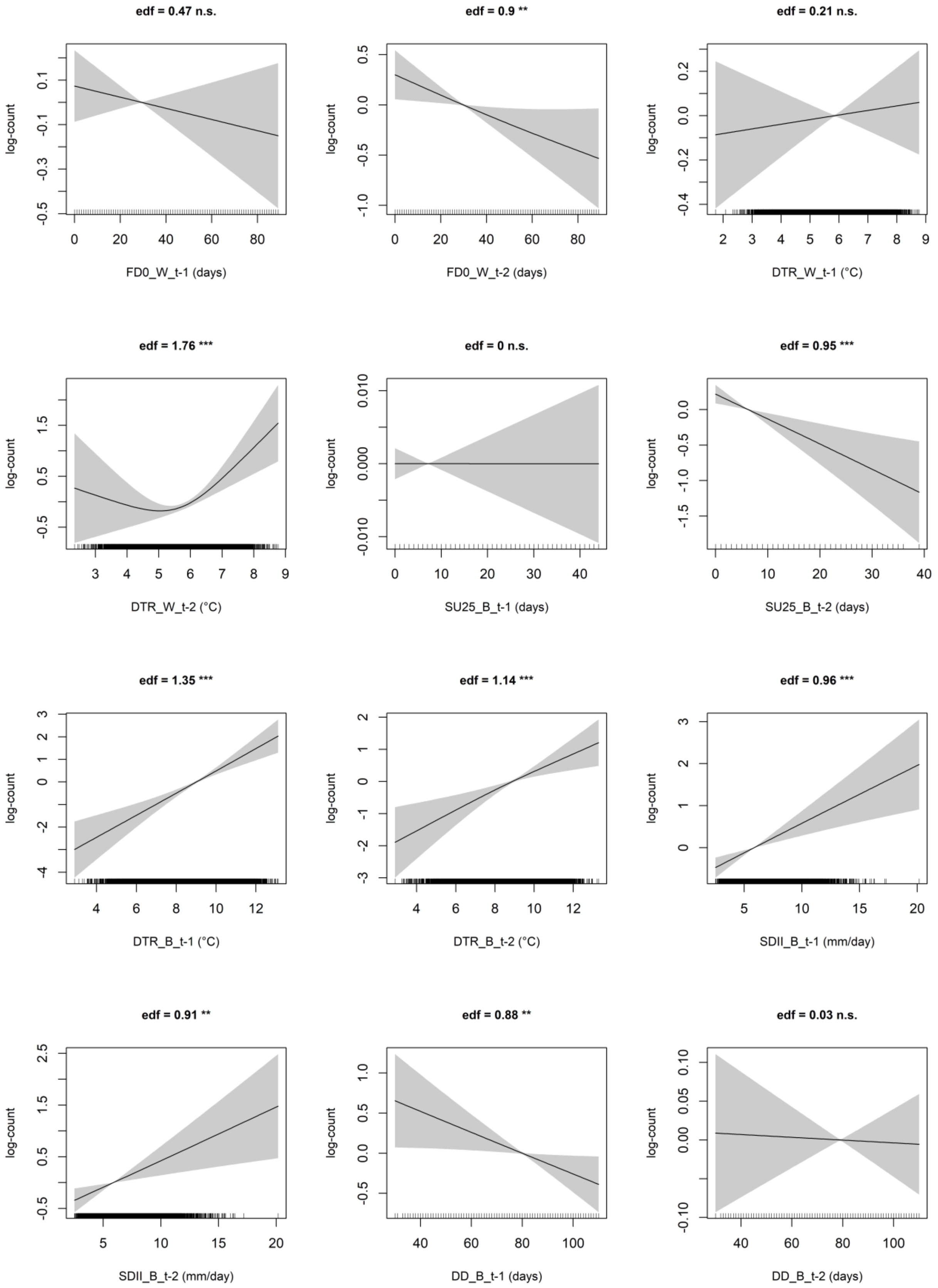

Red Kite *Milvus milvus*

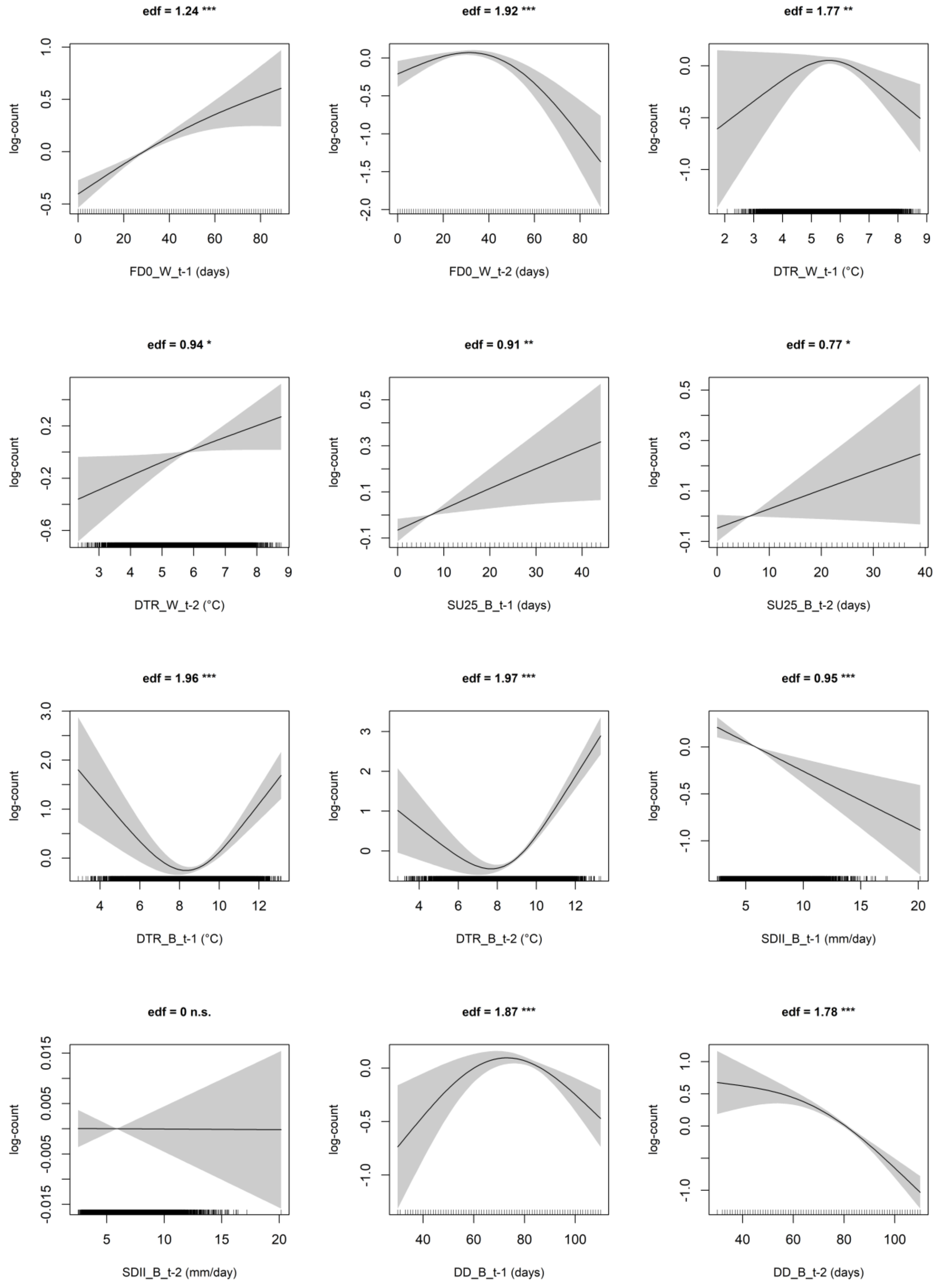

Western Marsh Harrier *Circus aeruginosus*

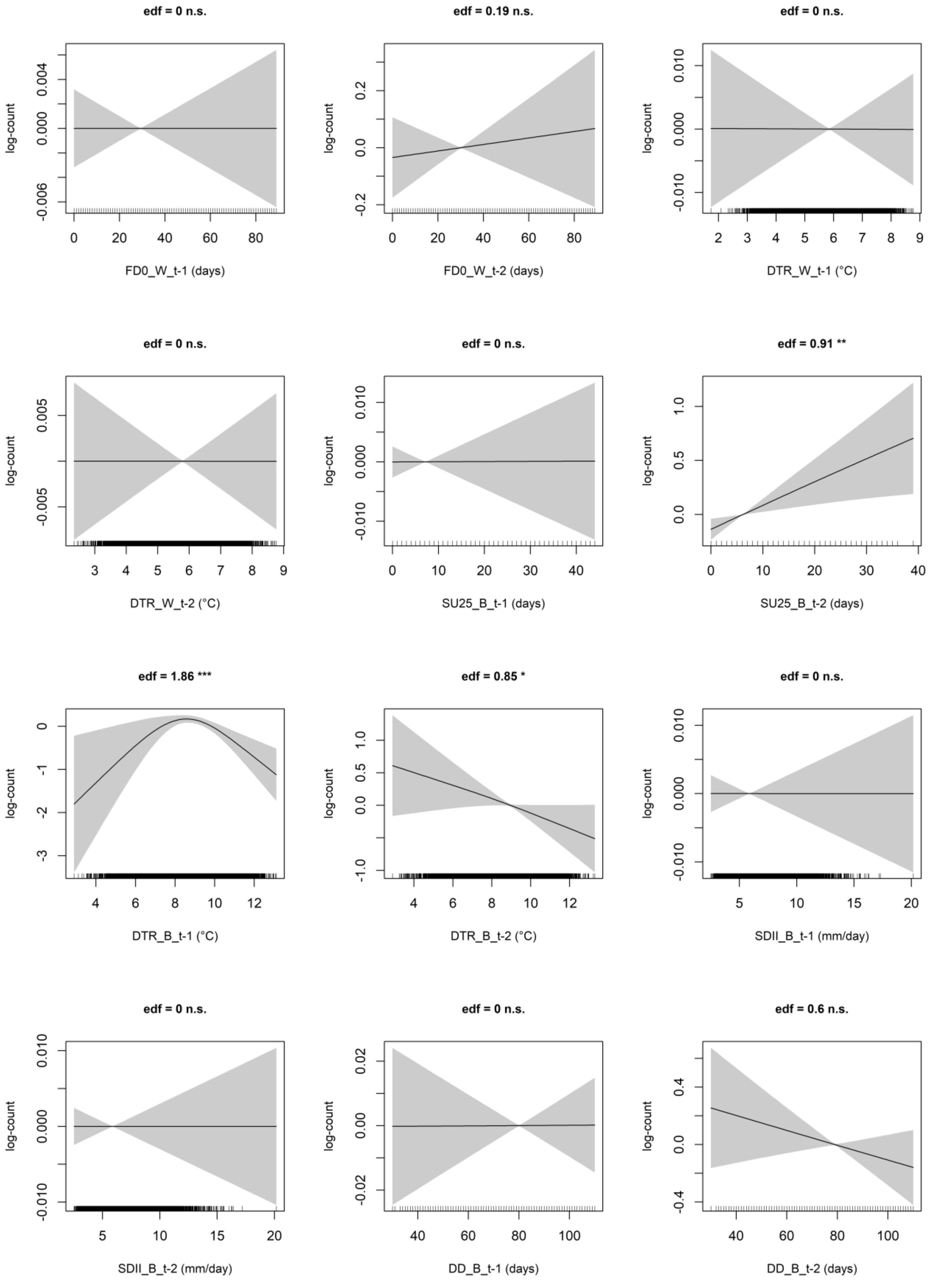

Hen Harrier *Circus cyaneus*

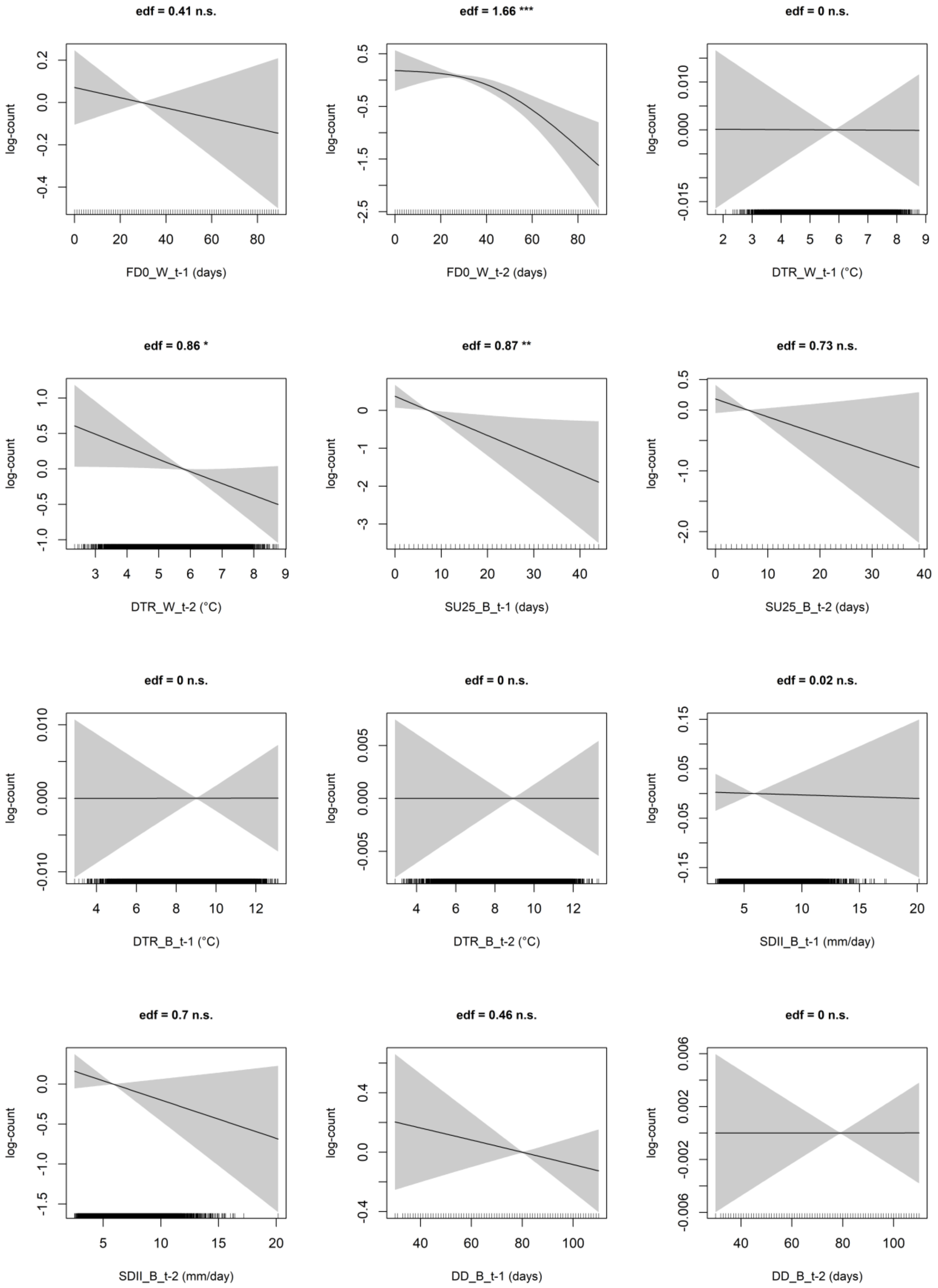

Northern Goshawk *Accipiter gentilis*

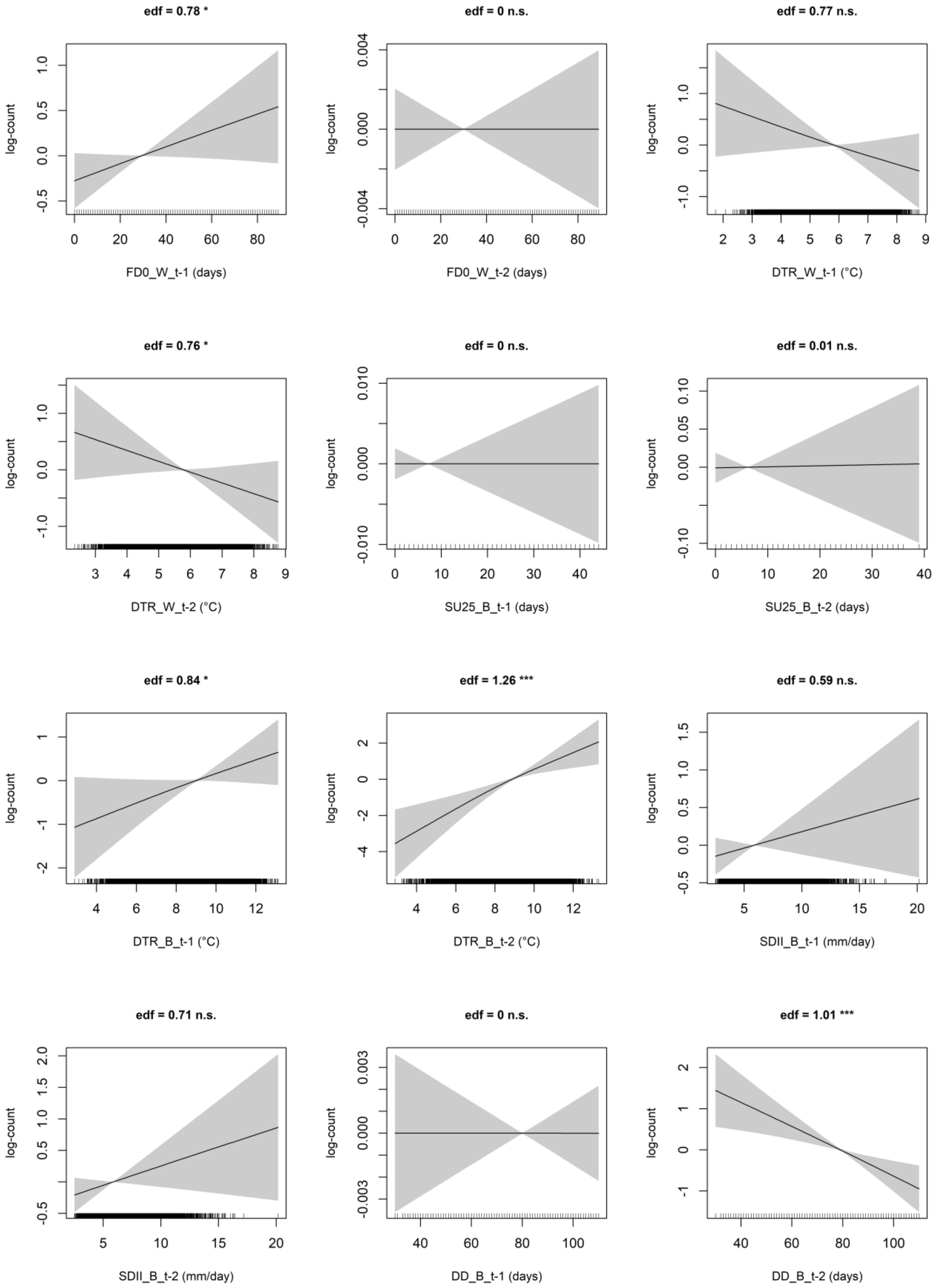

Eurasian Sparrowhawk *Accipiter nisus*

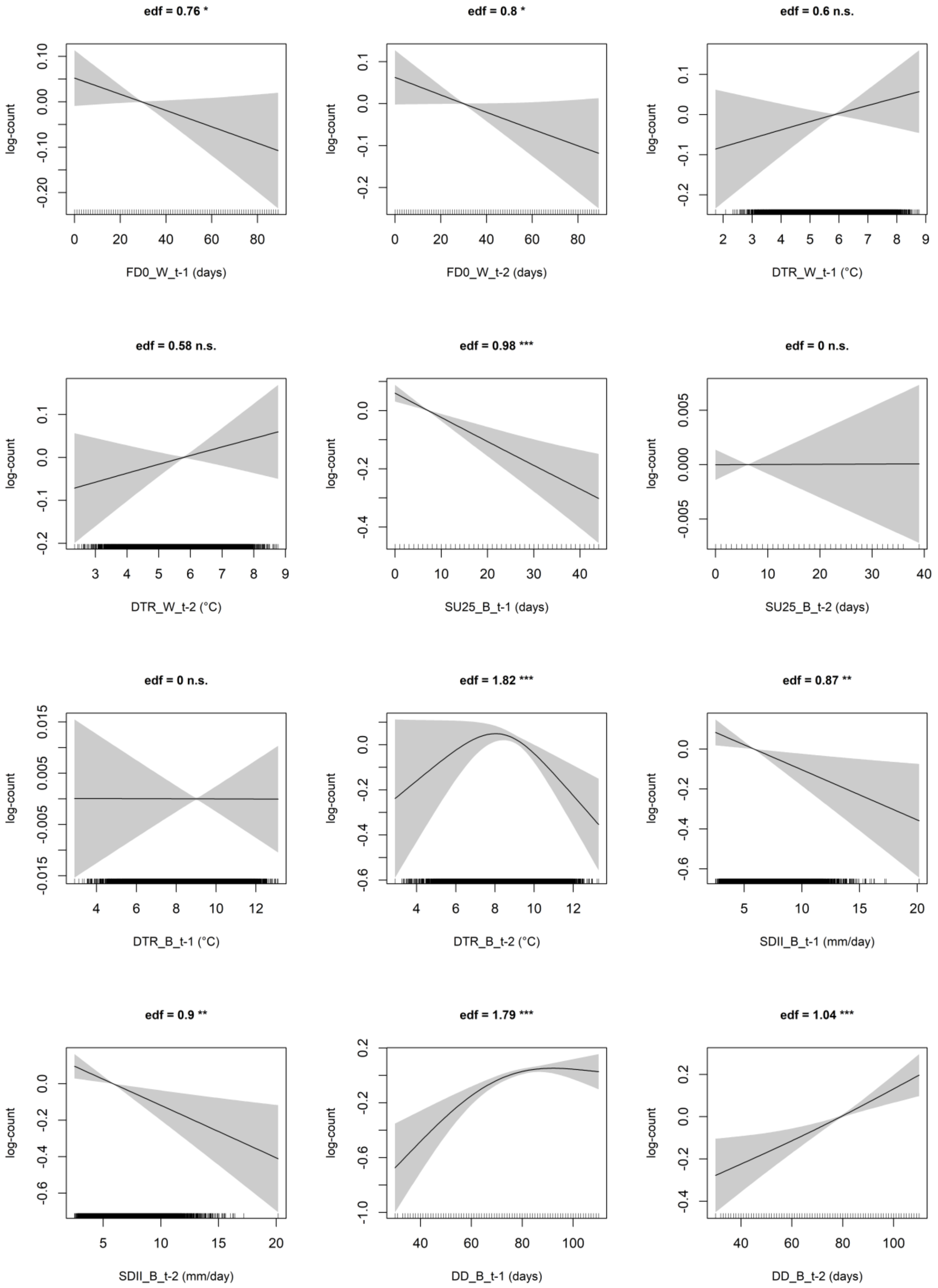

Common Buzzard *Buteo buteo*

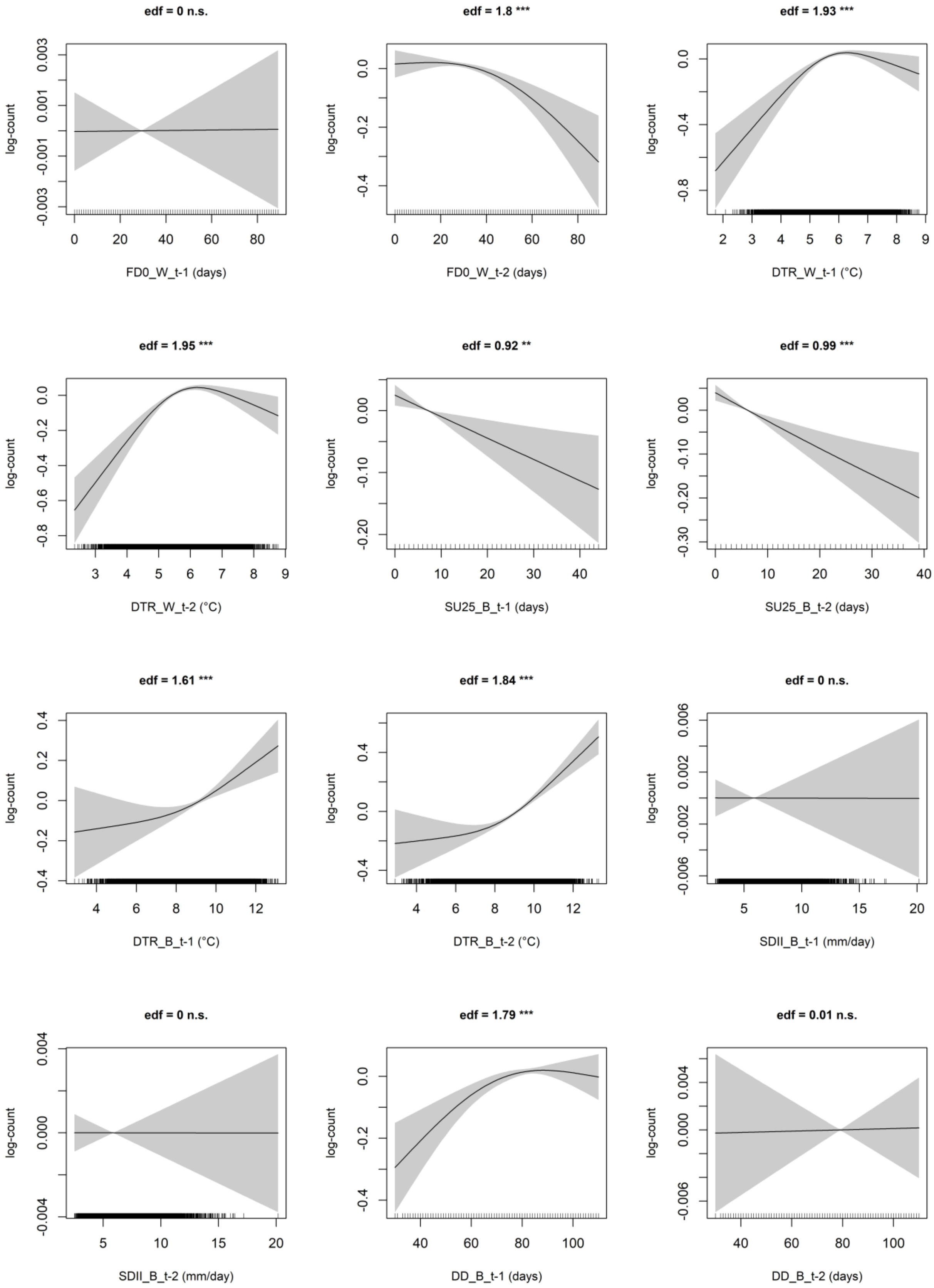

Common Kestrel *Falco tinnunculus*

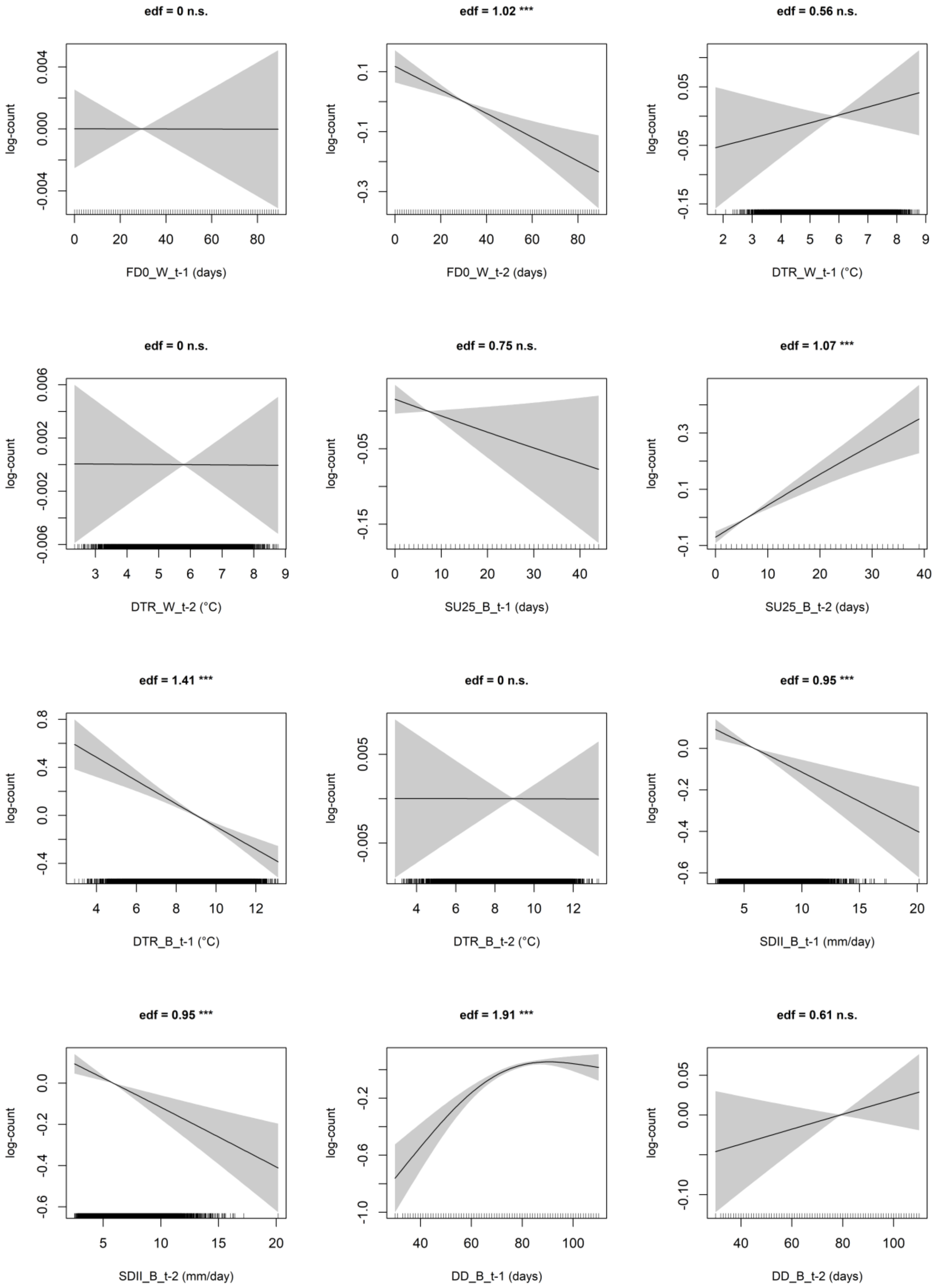

Merlin *Falco columbarius*

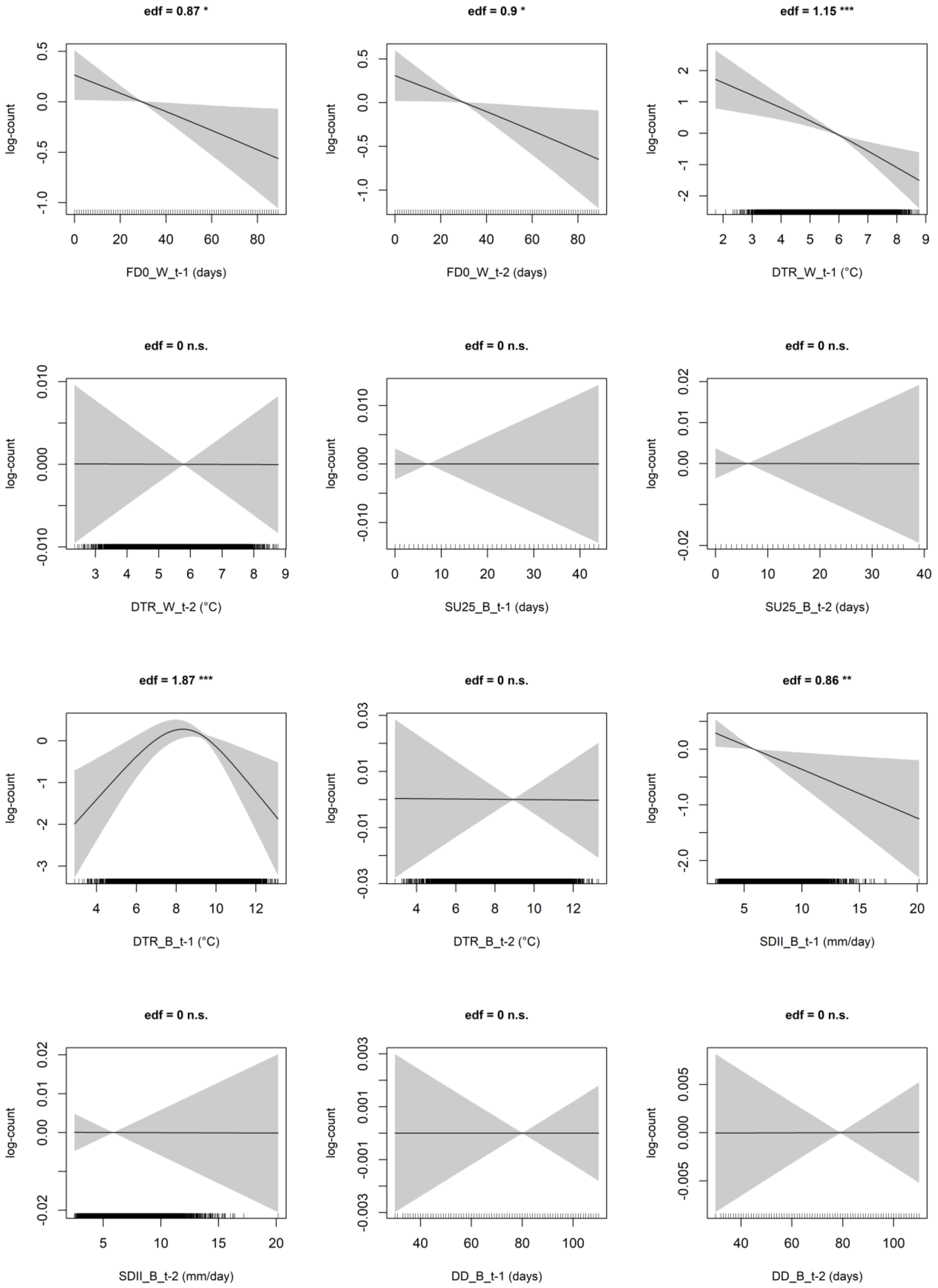

Peregrine Falcon *Falco peregrinus*

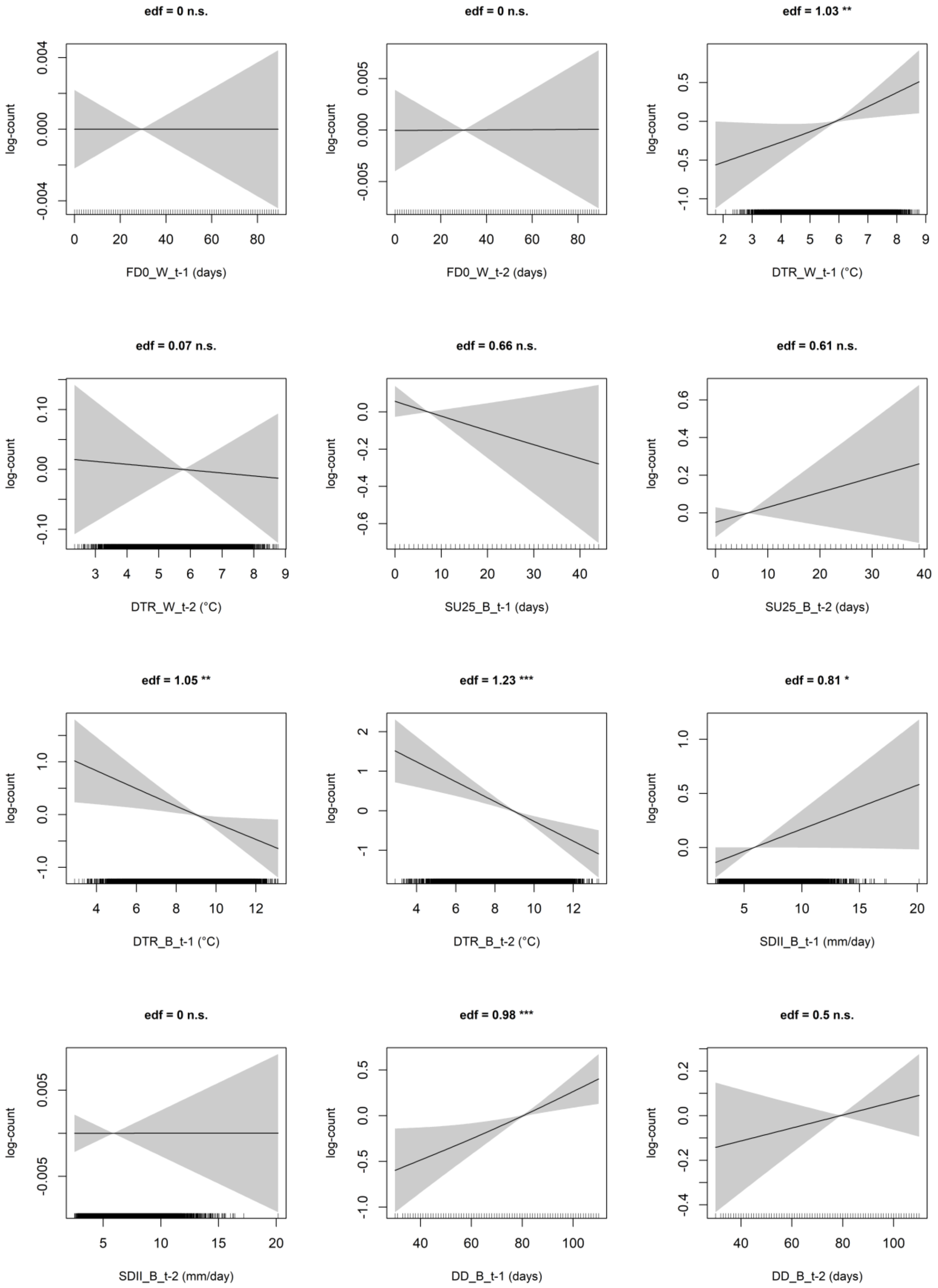

Willow Ptarmigan *Lagopus lagopus*

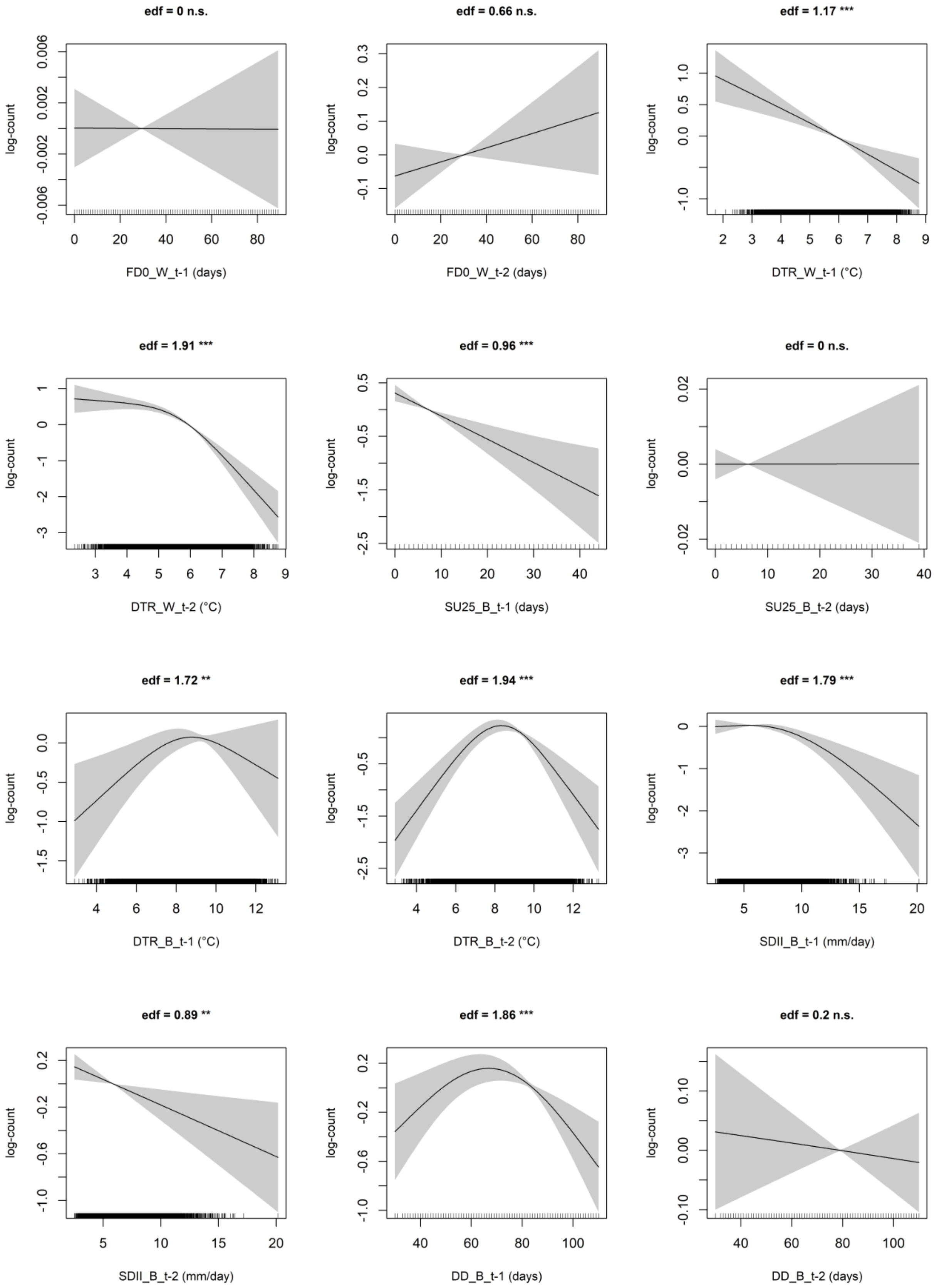

Black Grouse *Lyrurus tetrix*

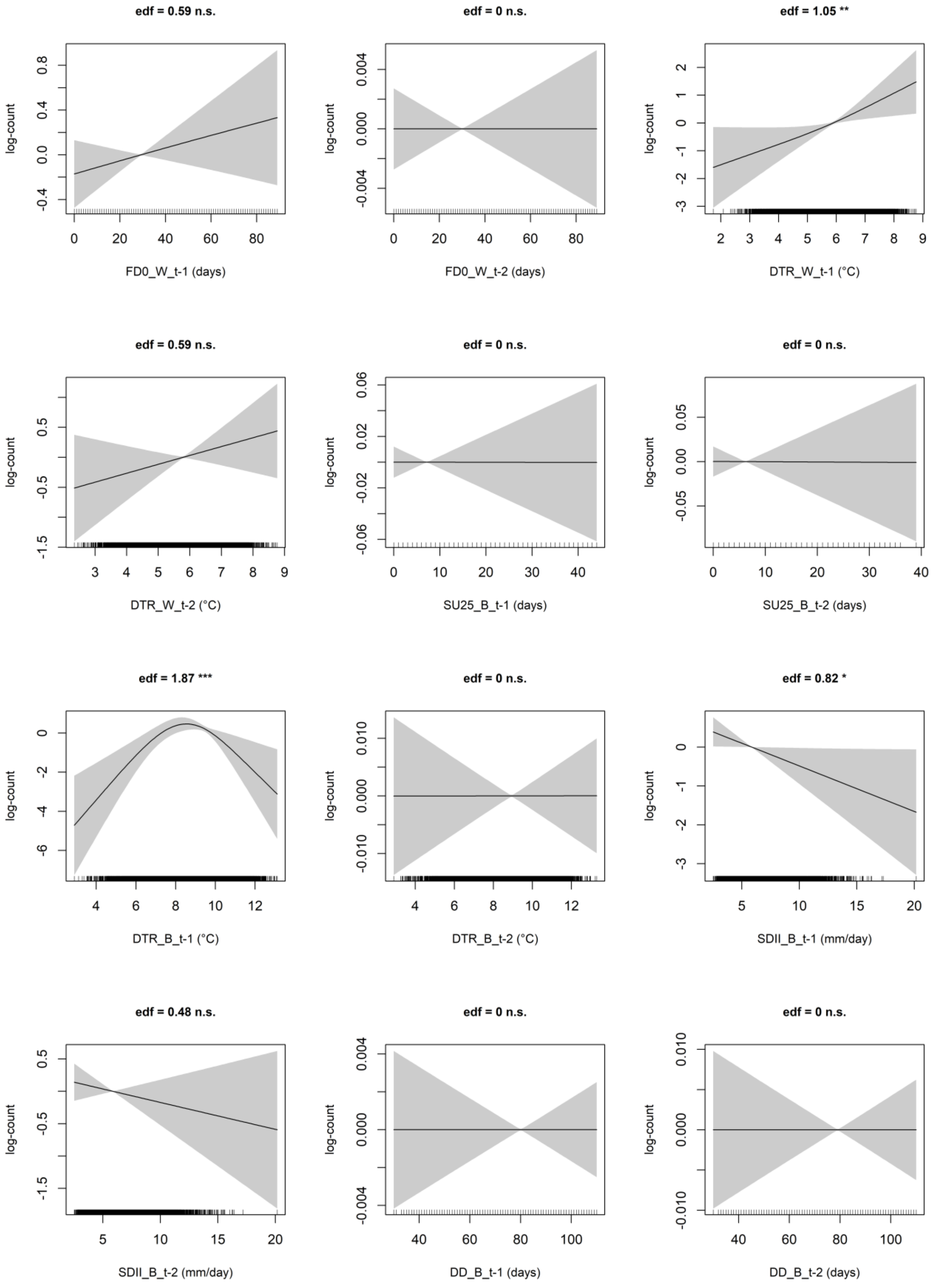

Red-legged Partridge *Alectoris rufa*

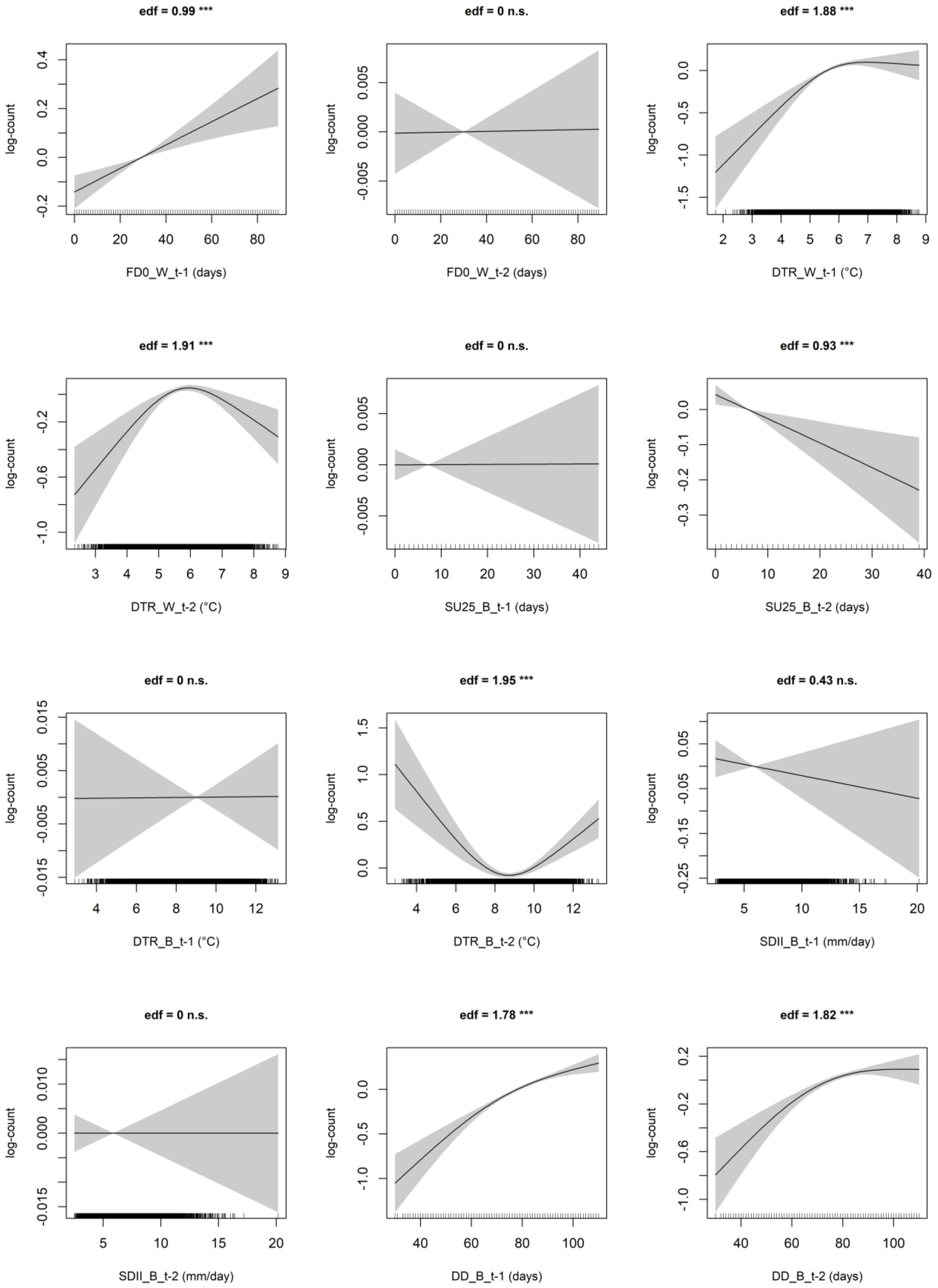

Grey Partridge *Perdix perdix*

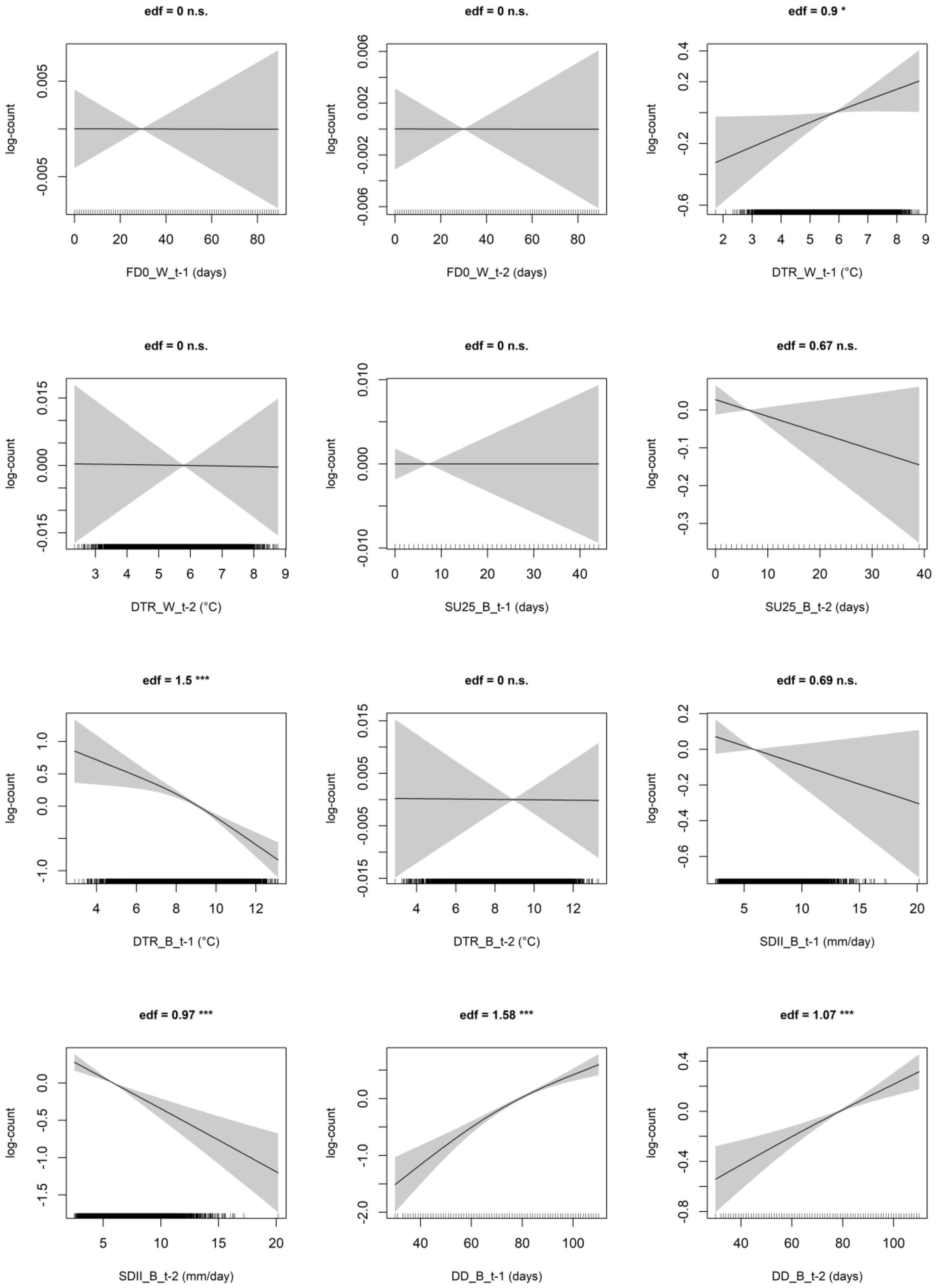

Common Pheasant *Phasianus colchicus*

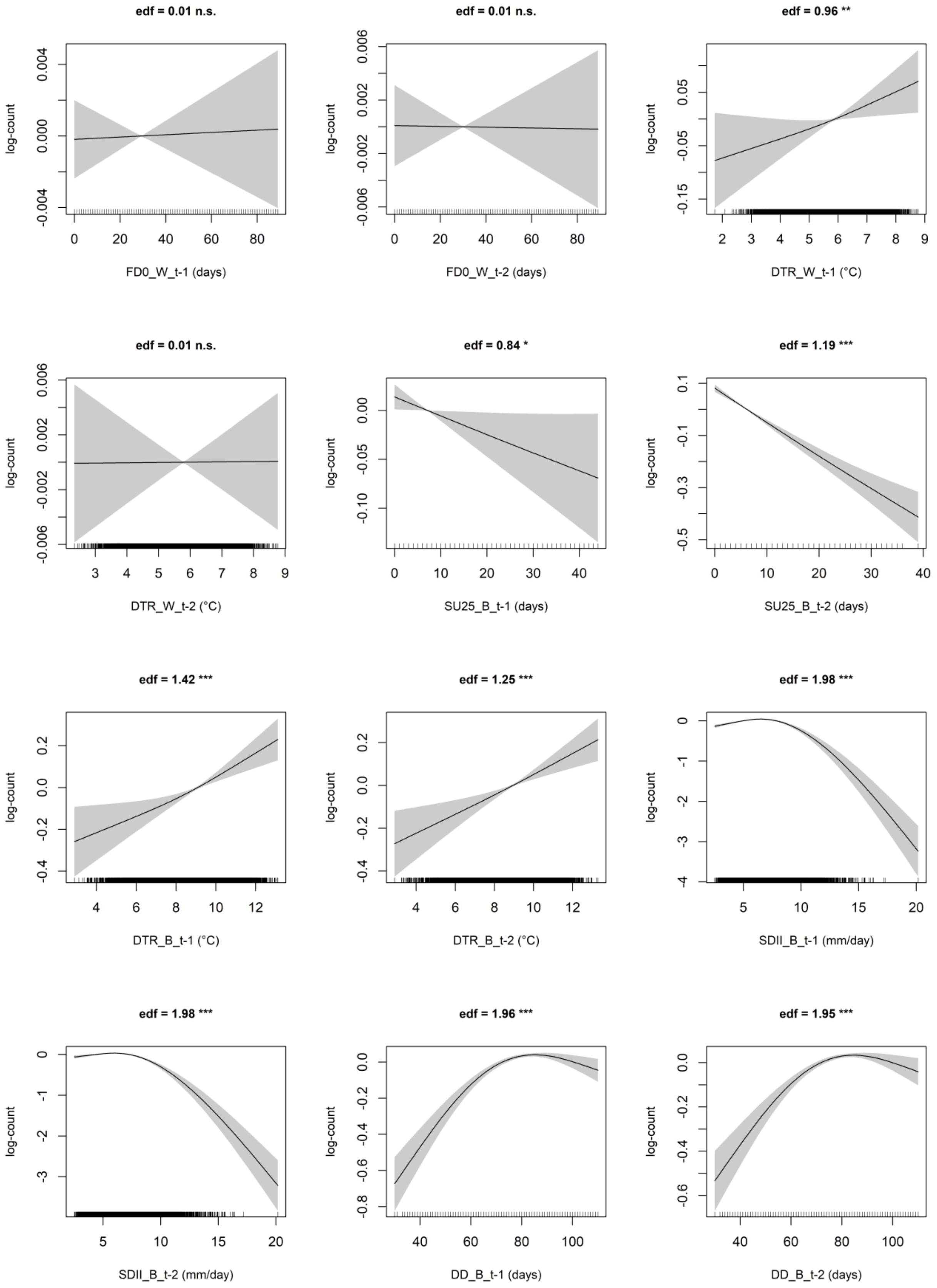

Common Moorhen *Gallinula chloropus*

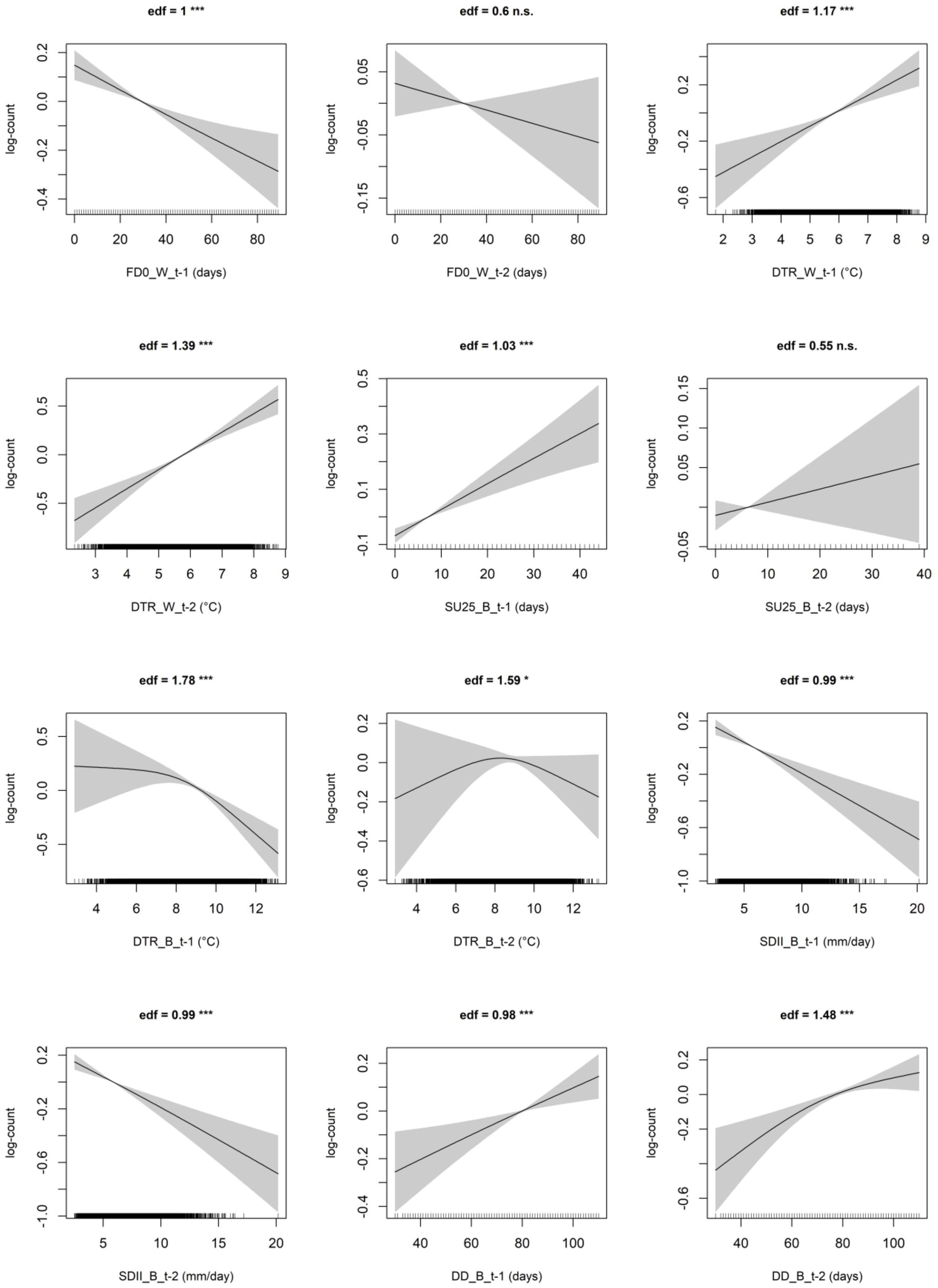

Eurasian Coot *Fulica atra*

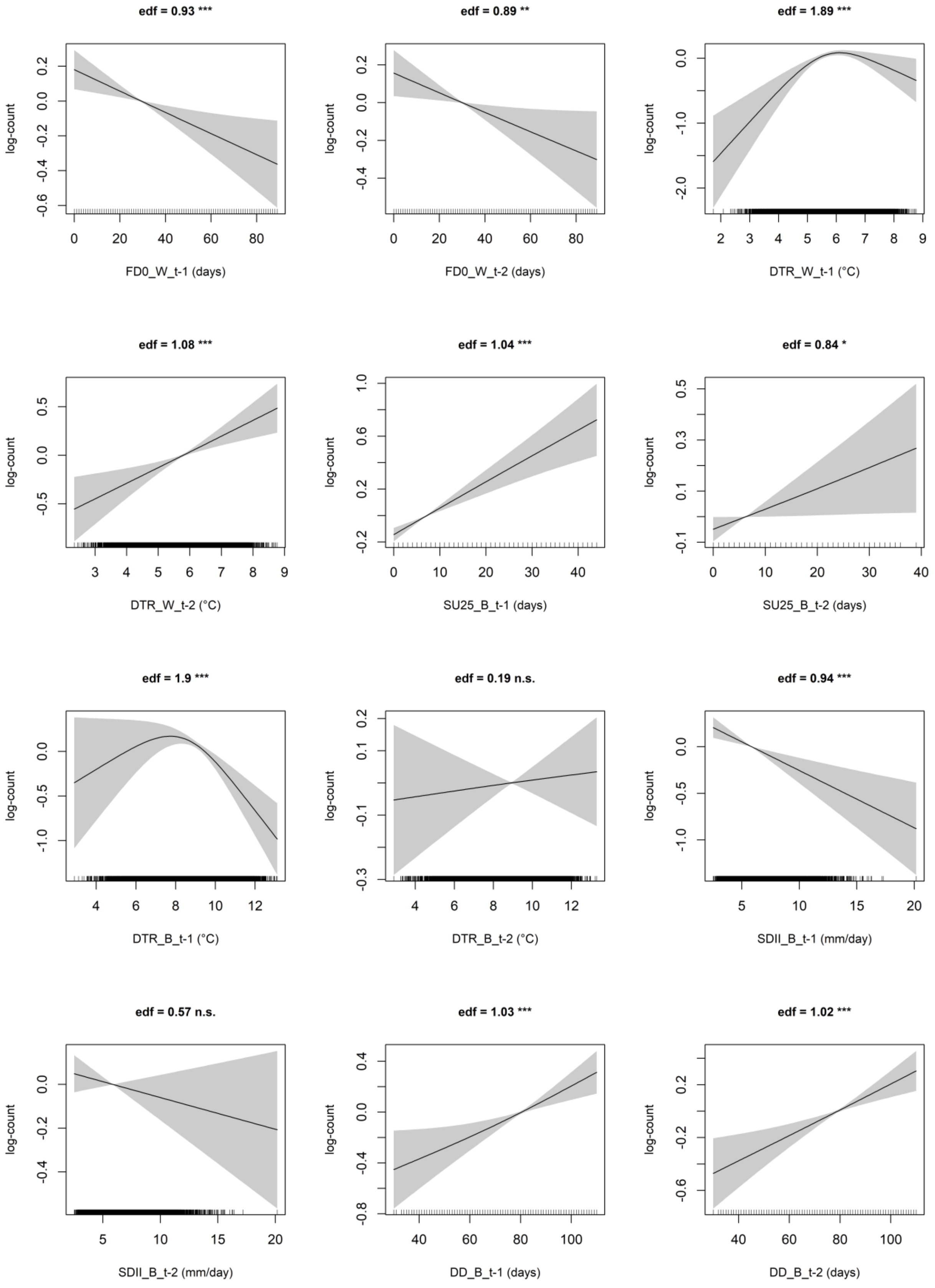

Eurasian Oystercatcher *Haematopus ostralegus*

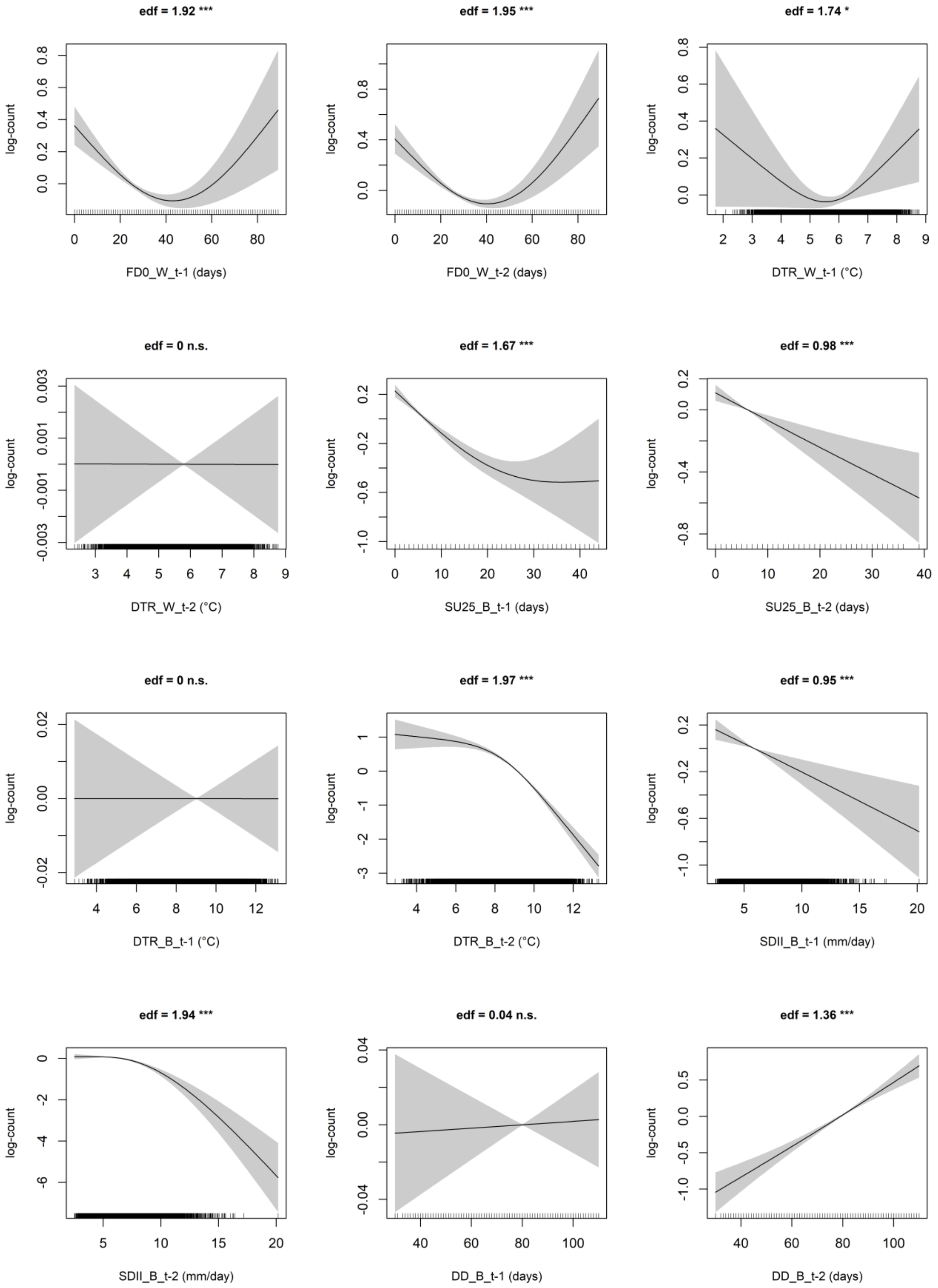

Common Ringed Plover *Charadrius hiaticula*

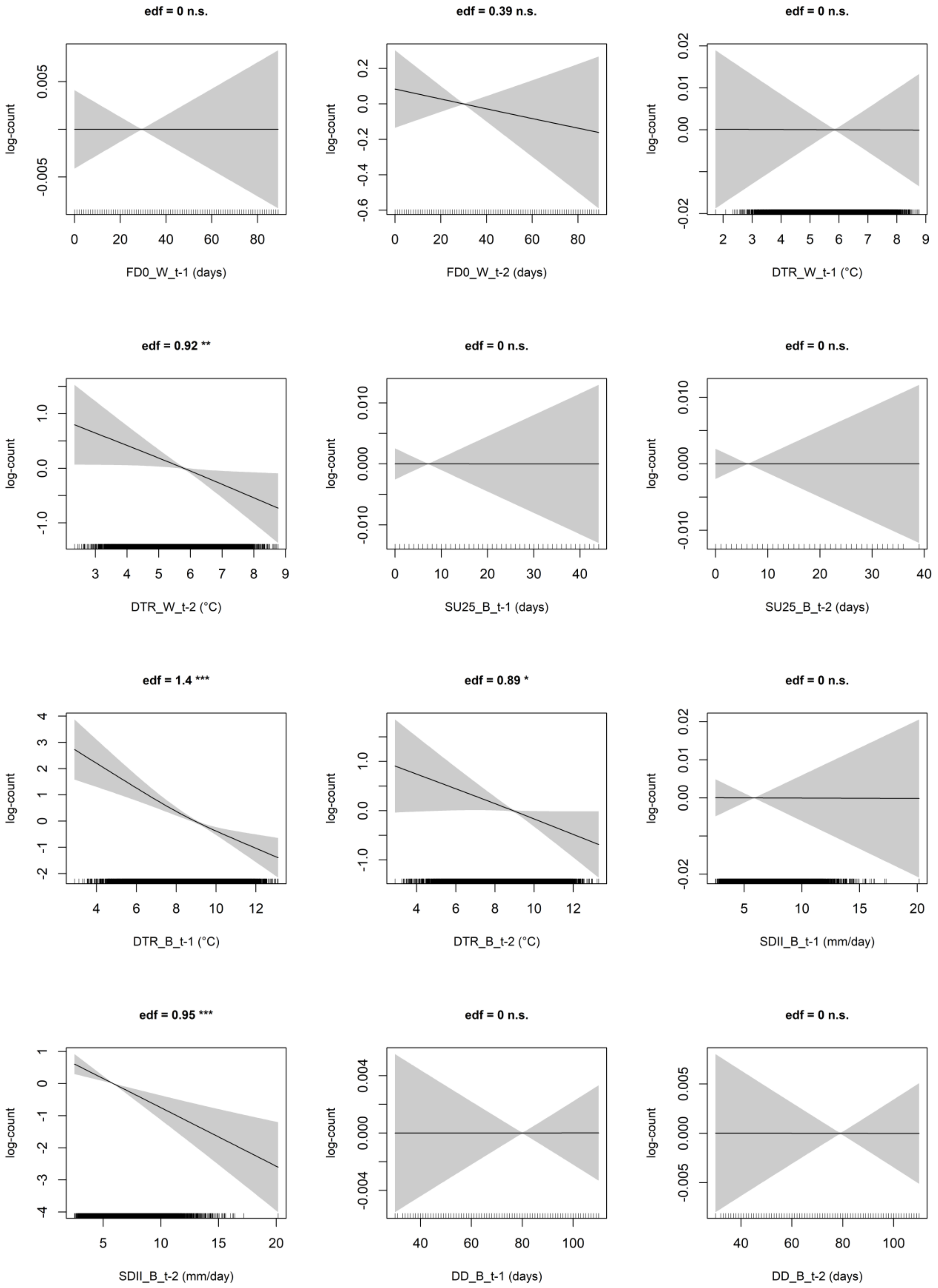

European Golden Plover *Pluvialis apricaria*

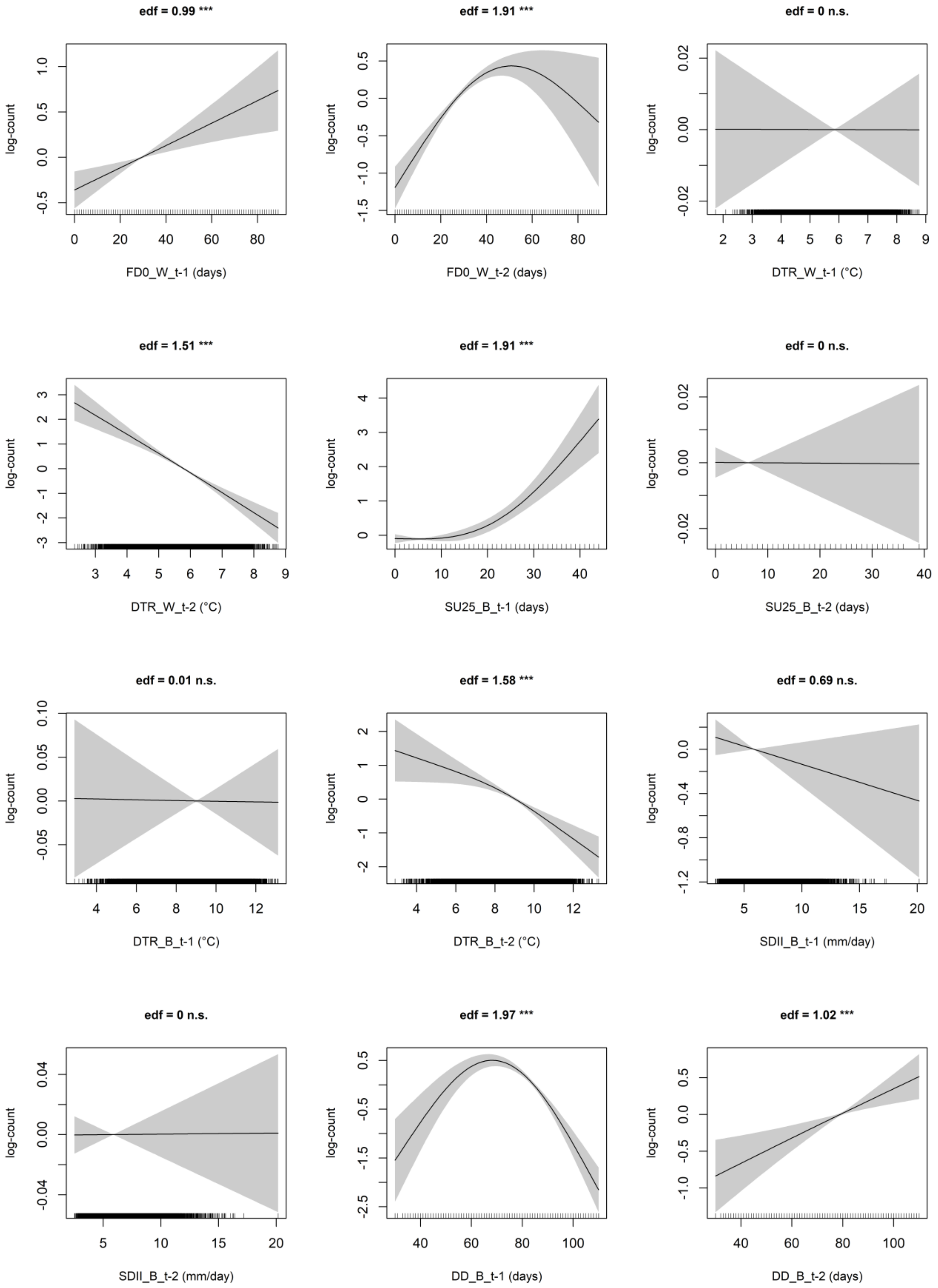

Northern Lapwing *Vanellus vanellus*

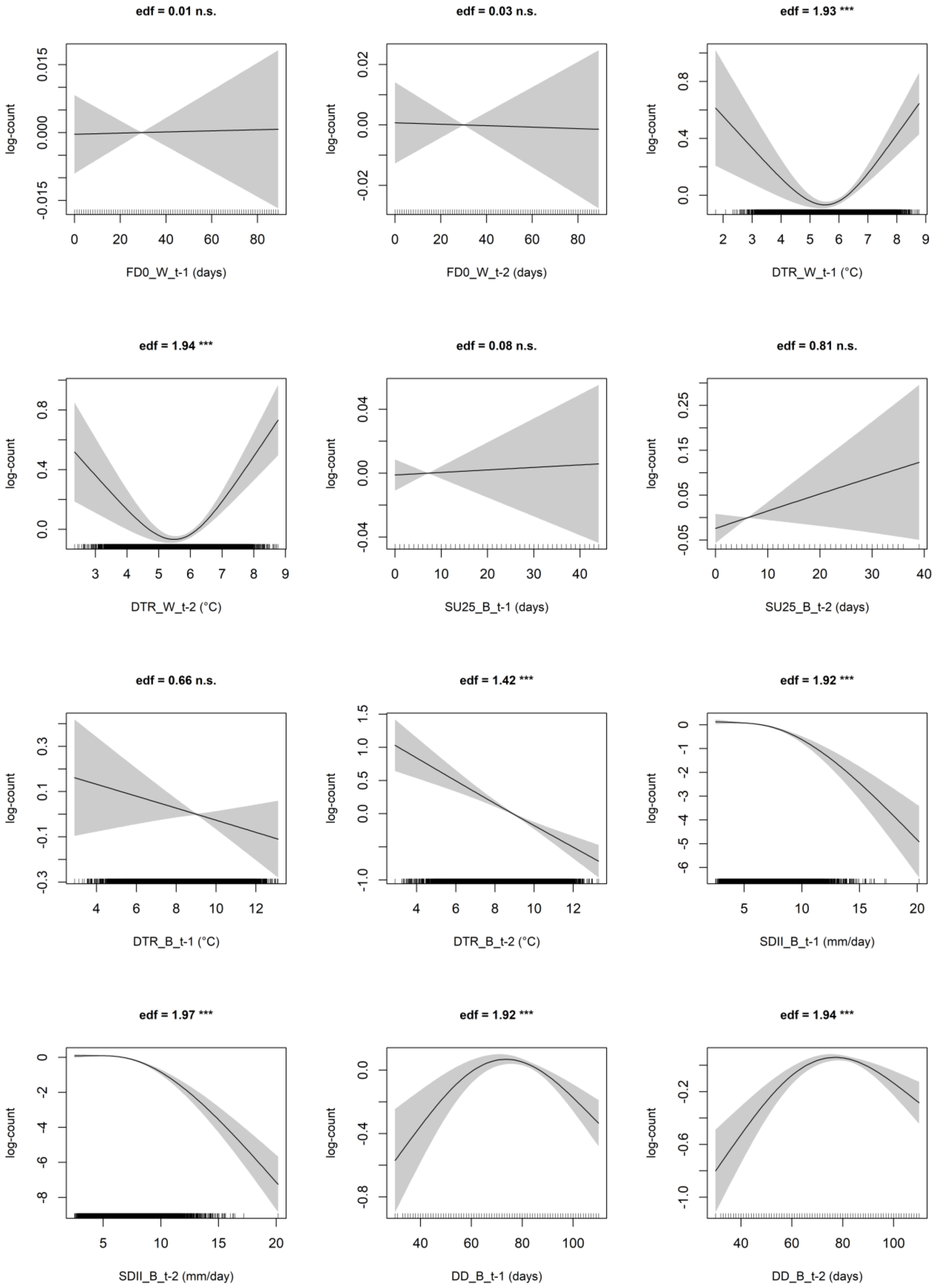

Common Snipe *Gallinago gallinago*

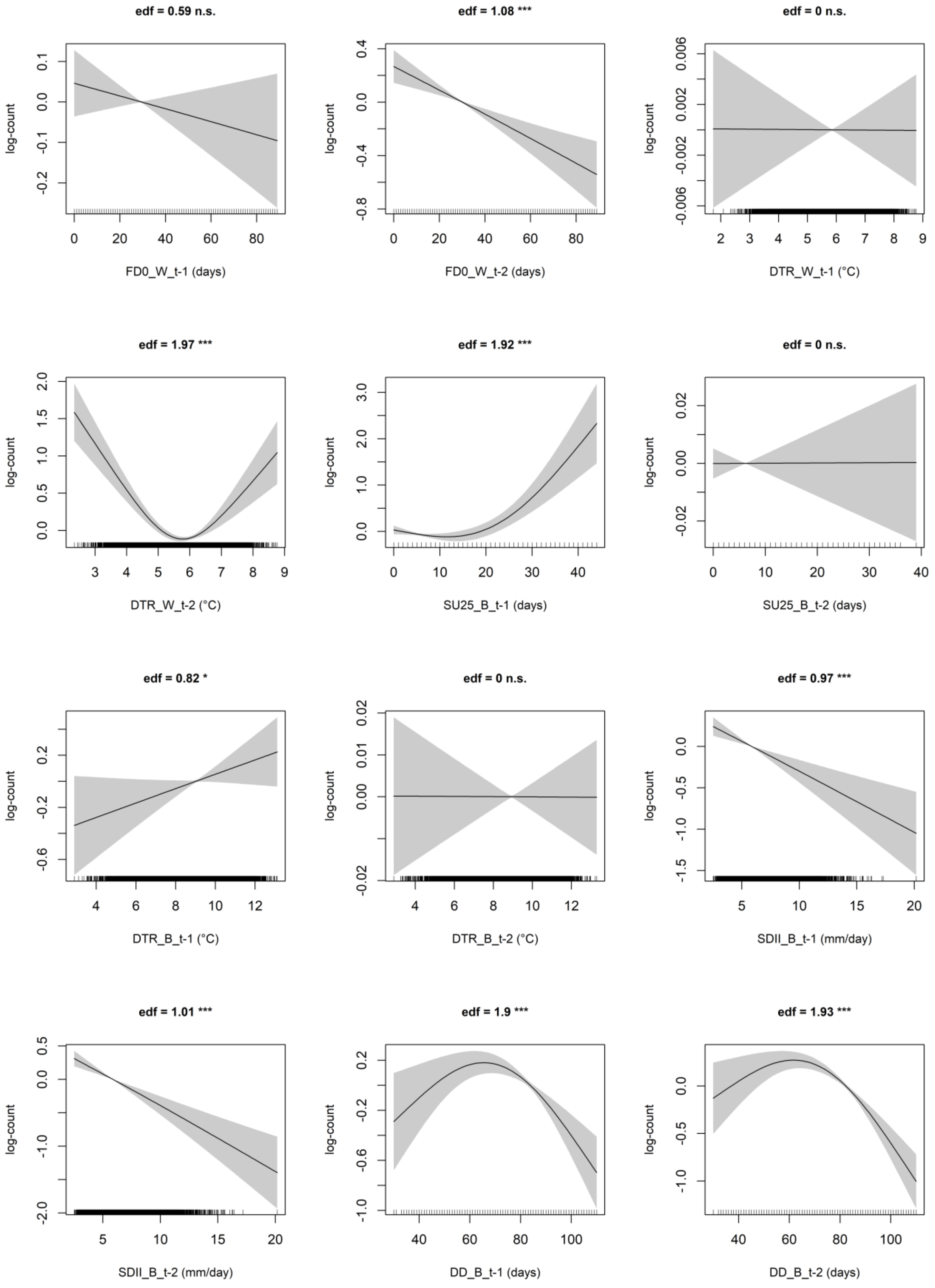

Eurasian Woodcock *Scolopax rusticola*

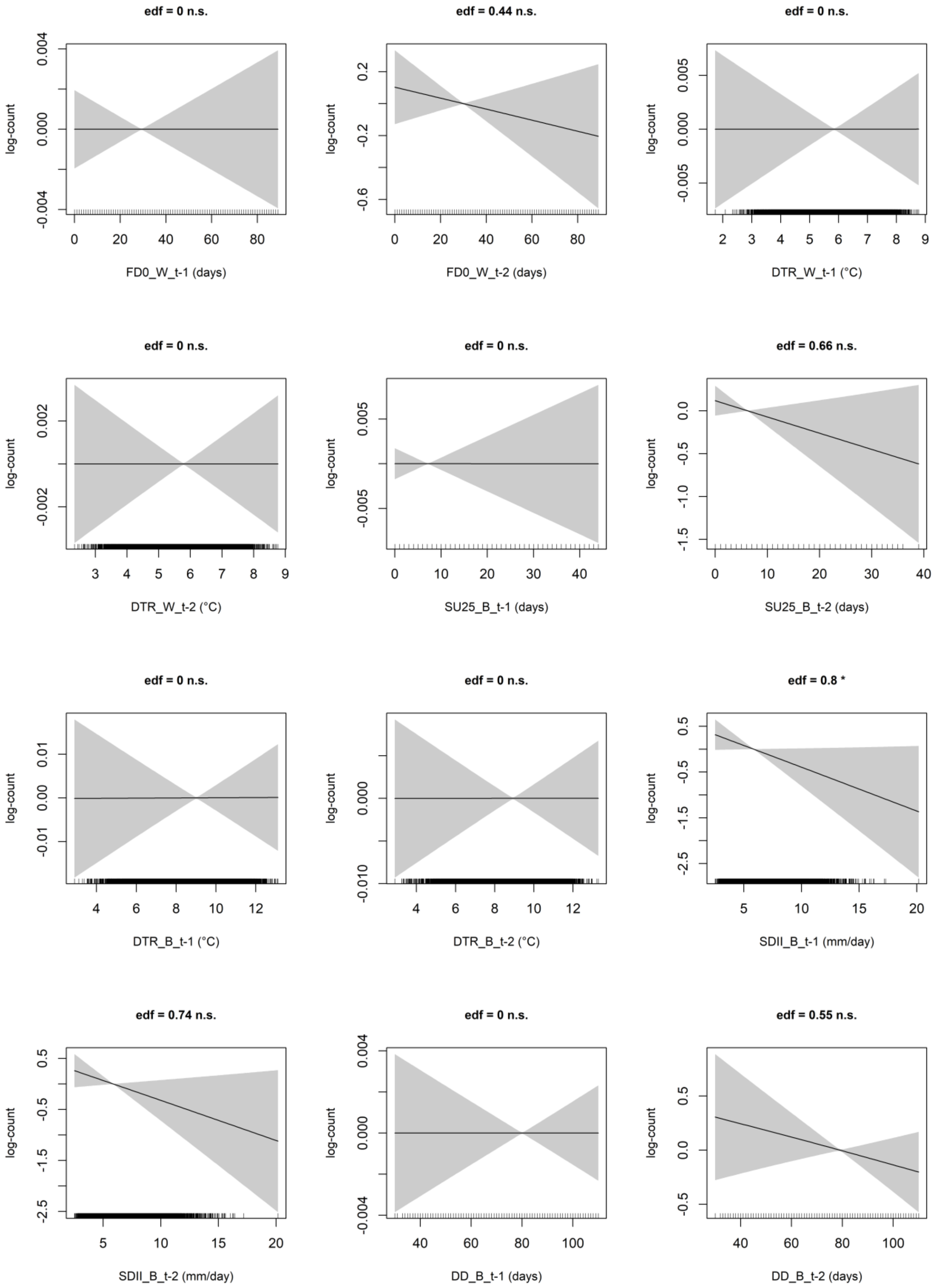

Eurasian Curlew *Numenius arquata*

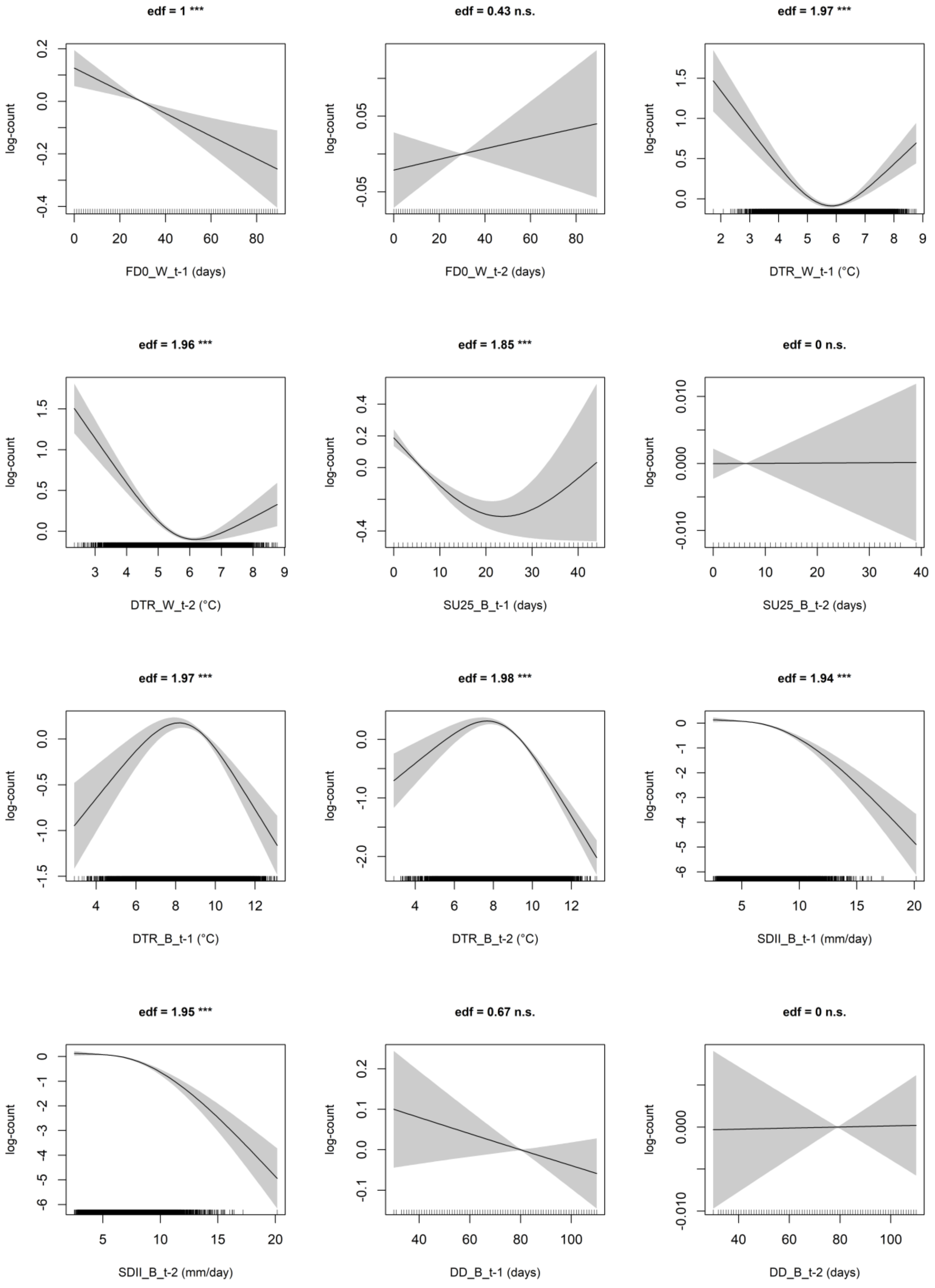

Common Redshank *Tringa totanus*

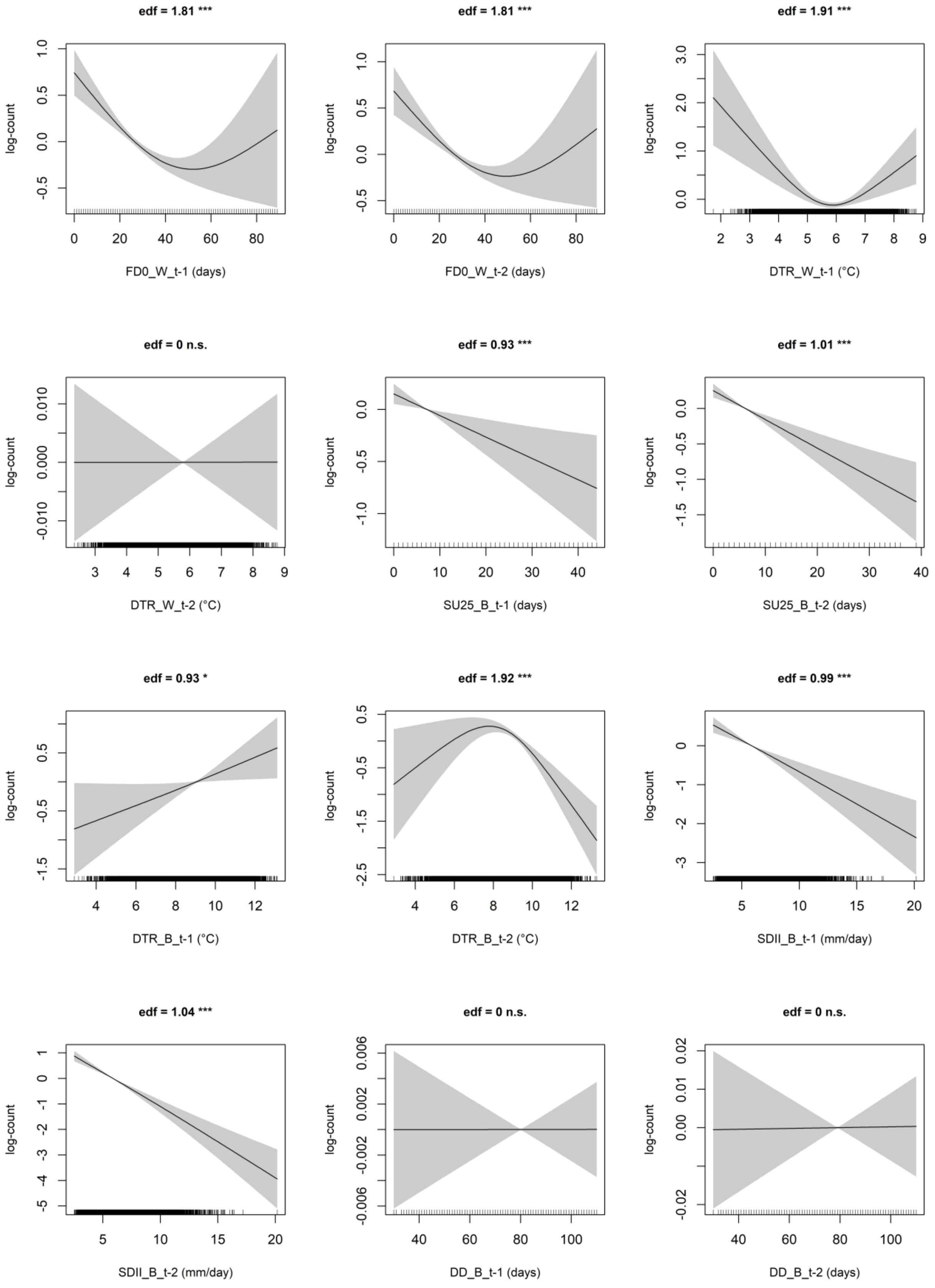

Feral Pigeon *Columba livia*

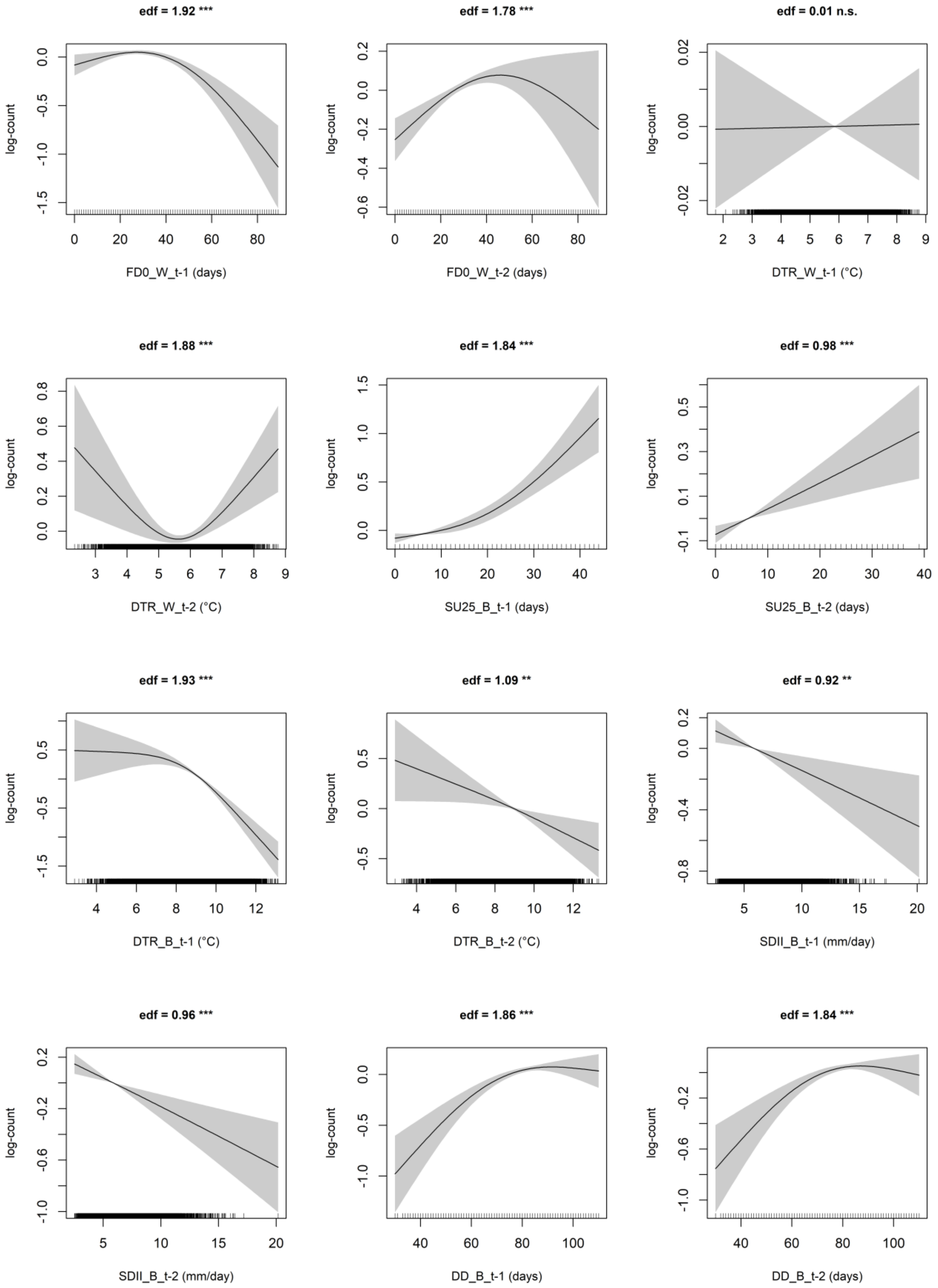

Stock Dove *Columba oenas*

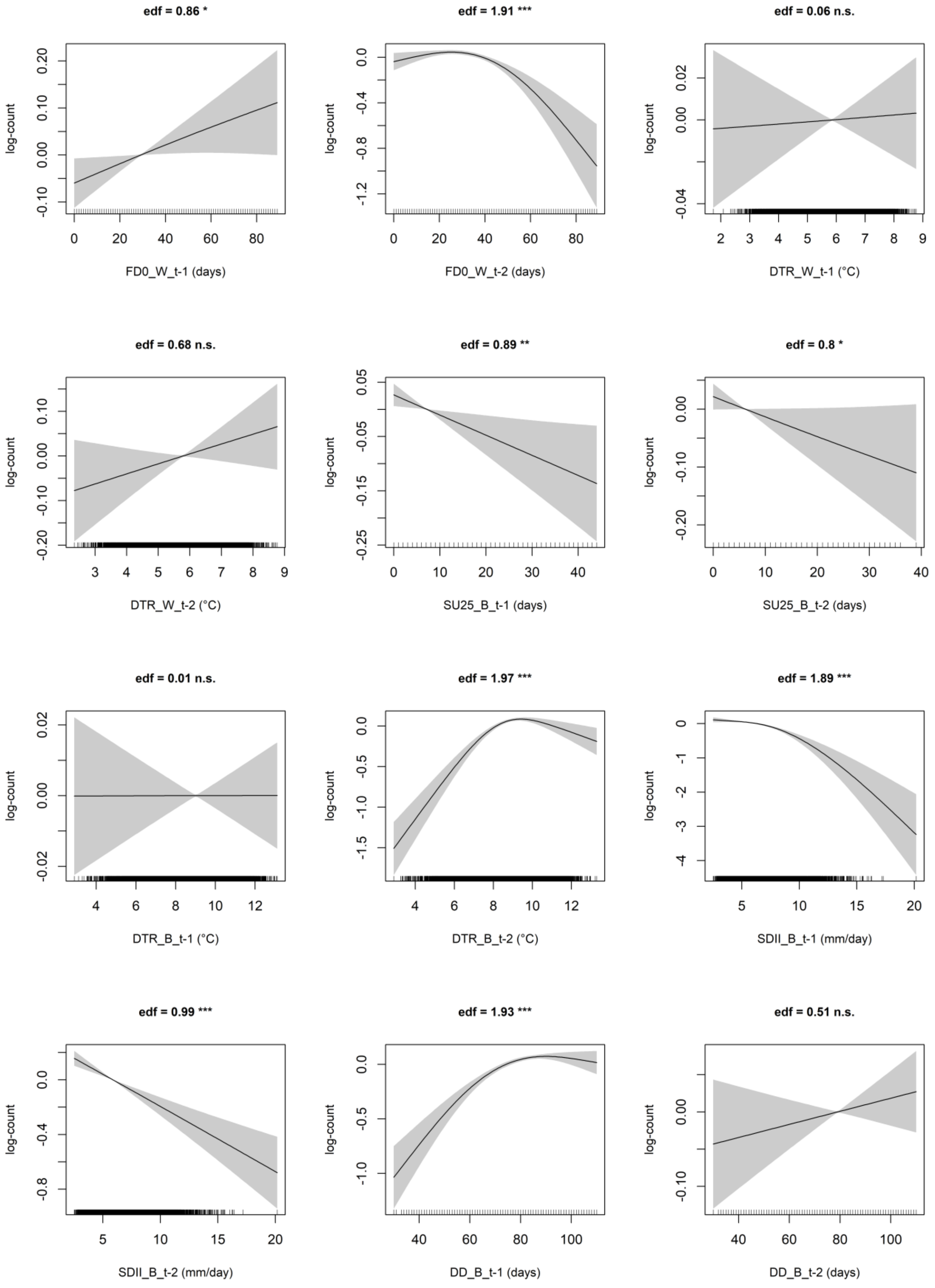

Common Wood Pigeon *Columba palumbus*

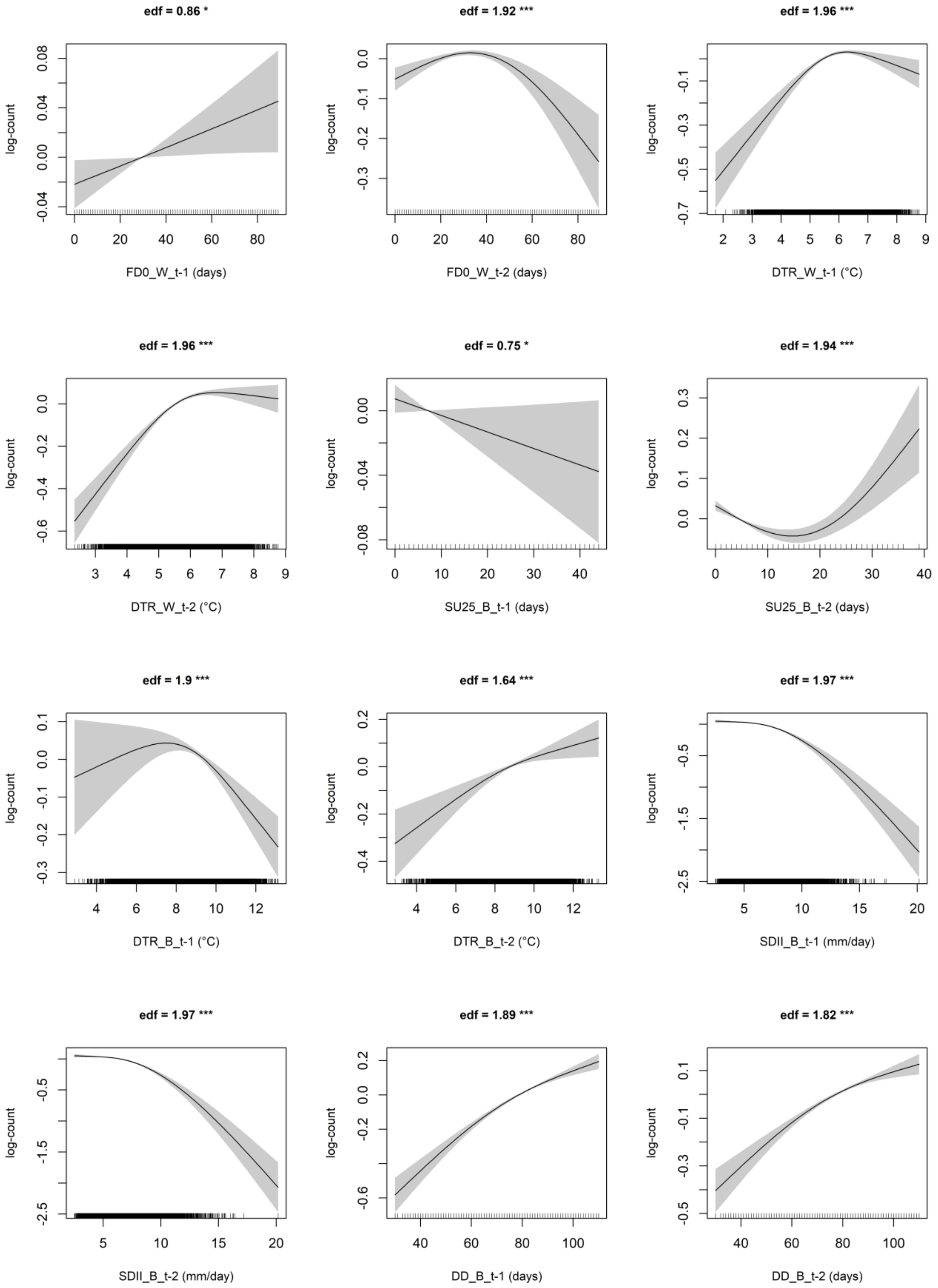

Eurasian Collared Dove *Streptopelia decaocto*

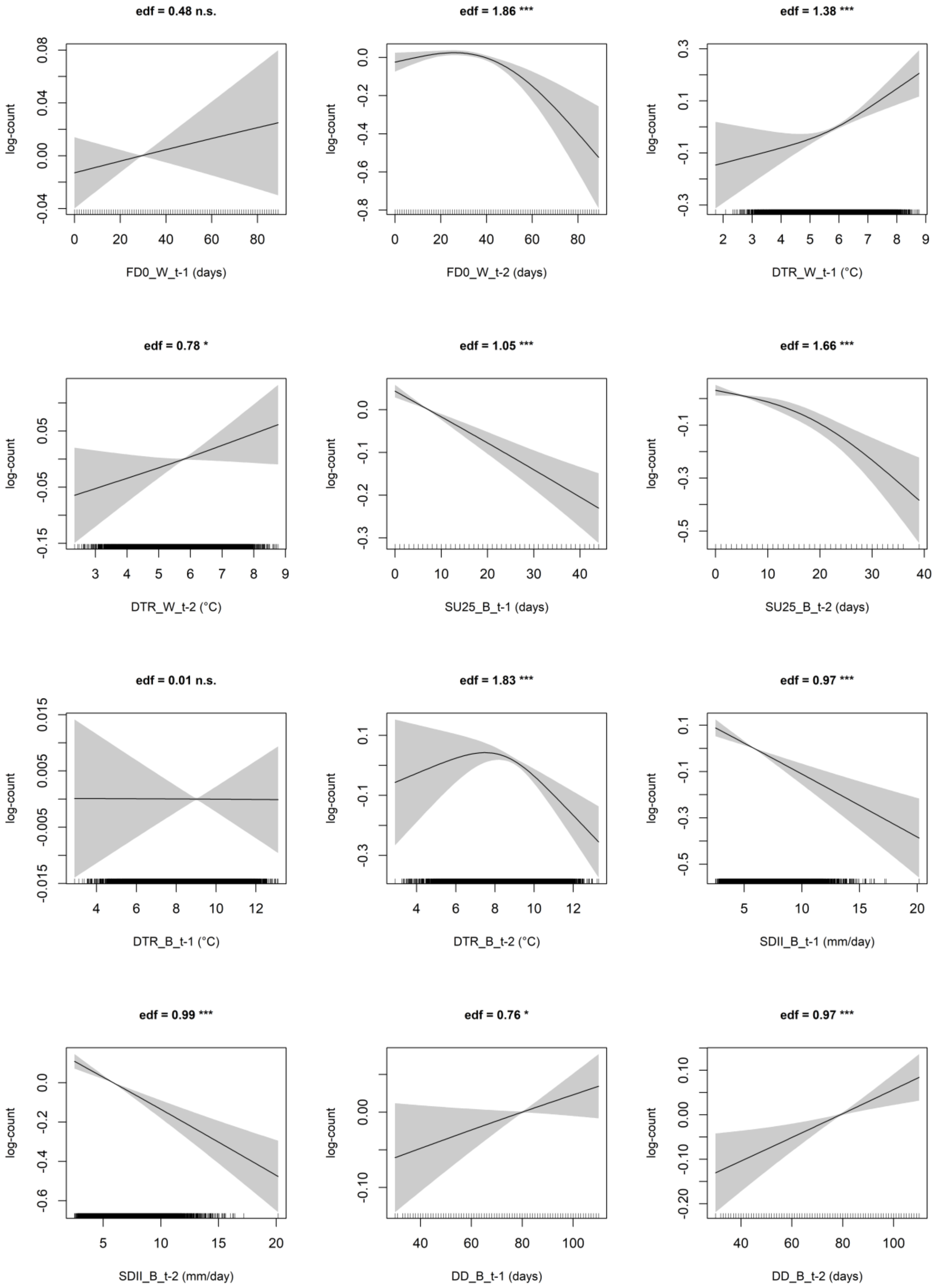

Rose-ringed Parakeet *Psittacula krameri*

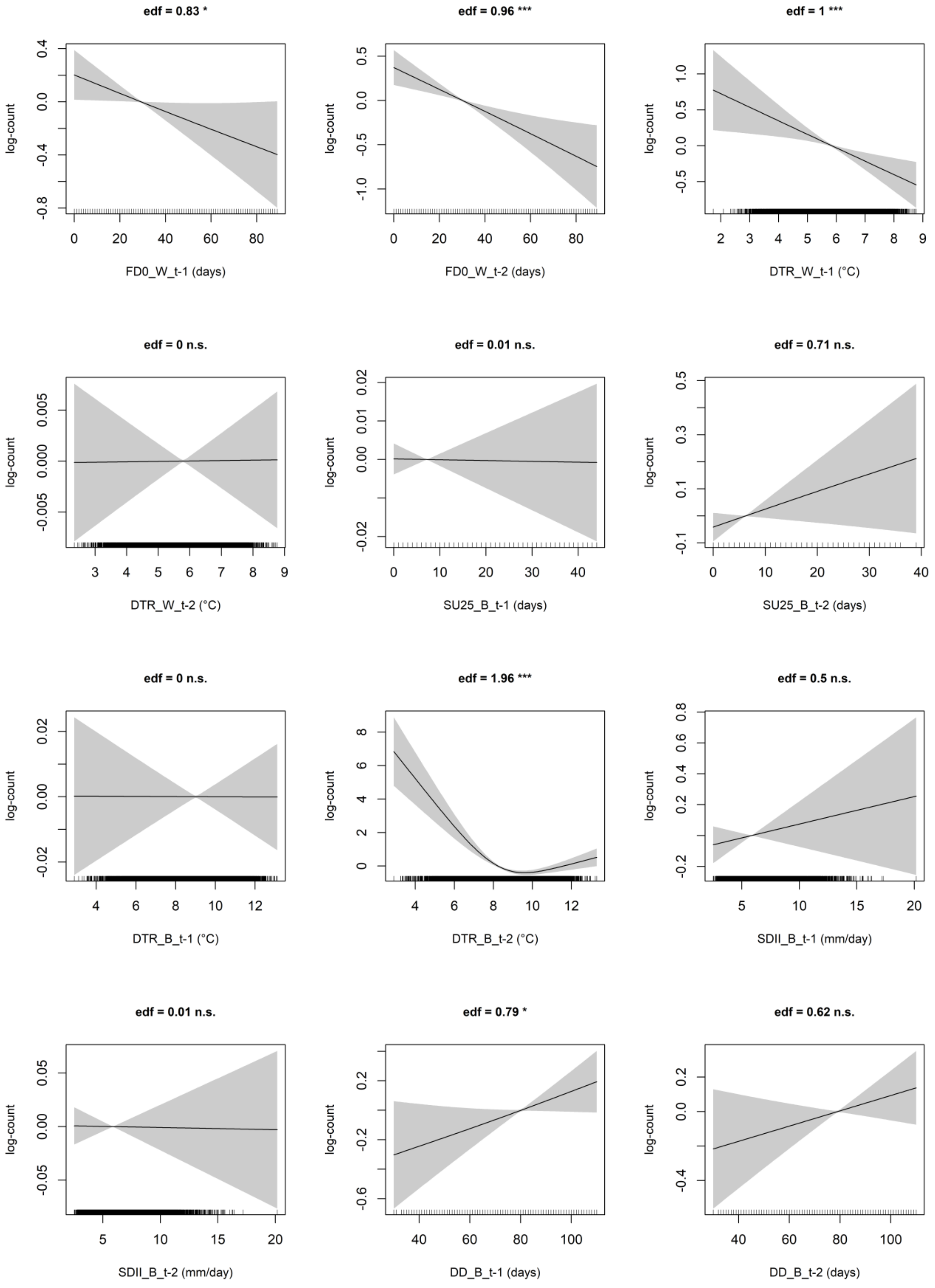

Western Barn Owl *Tyto alba*

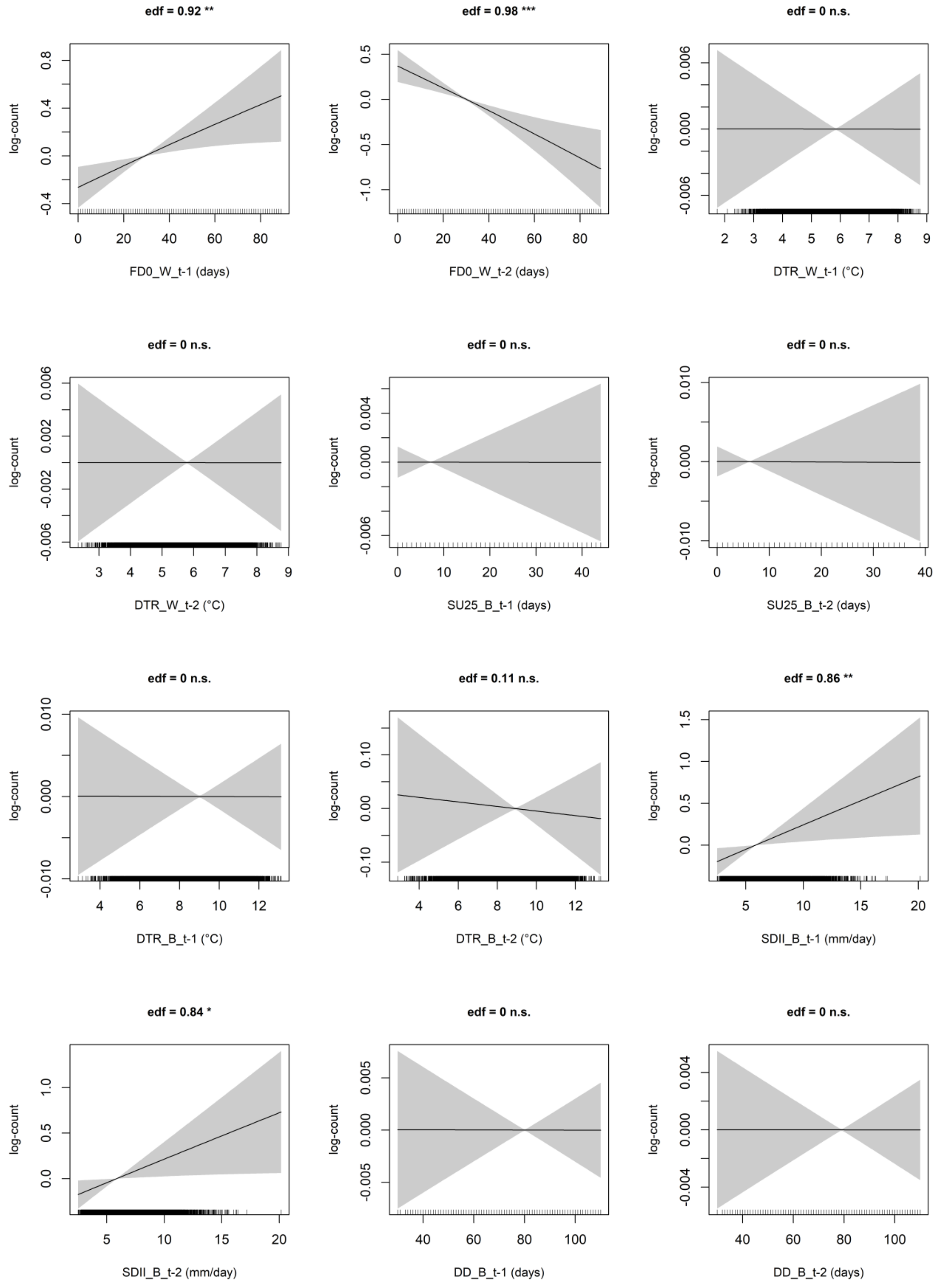

Little Owl *Athene noctua*

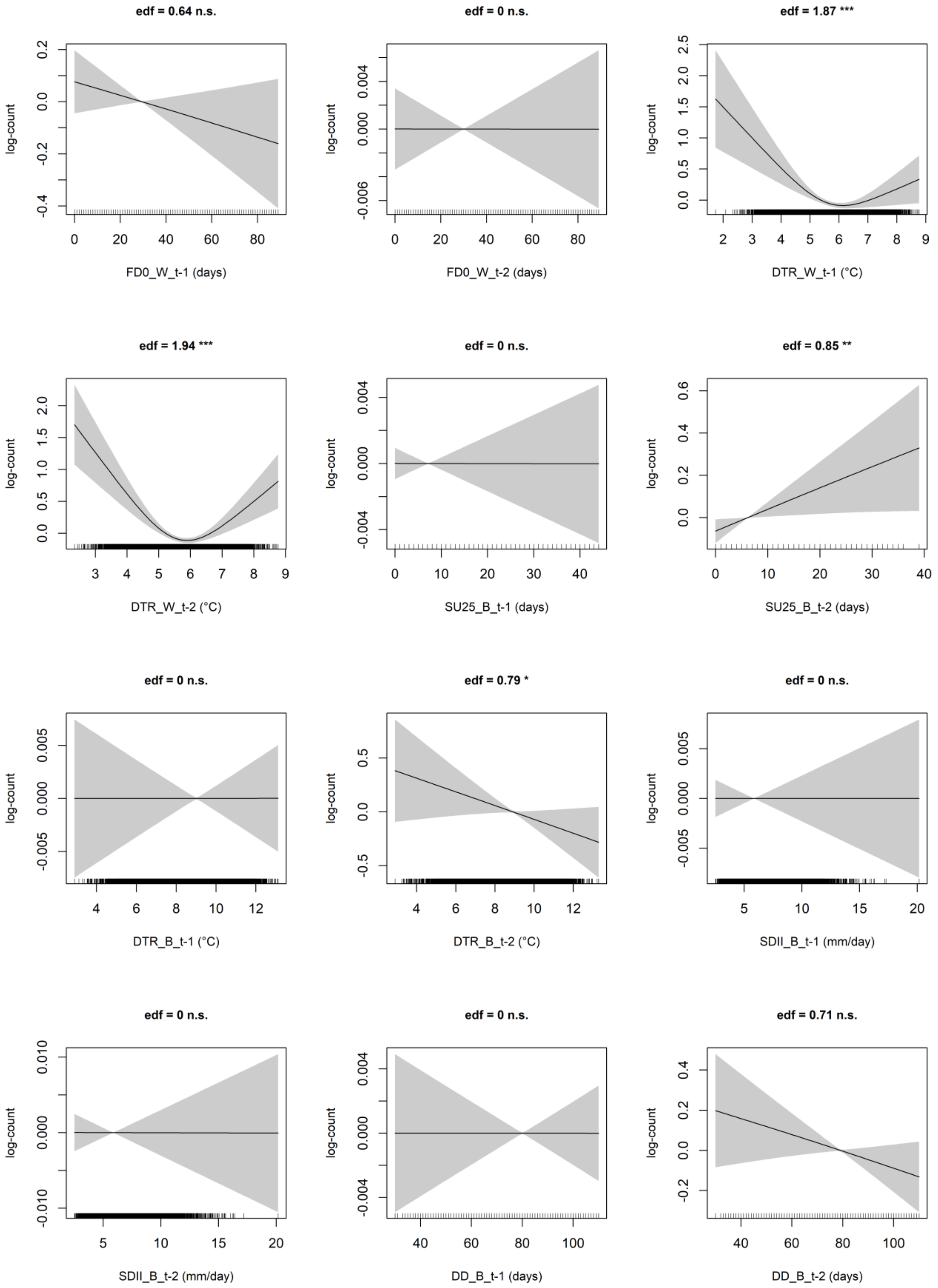

Tawny Owl *Strix aluco*

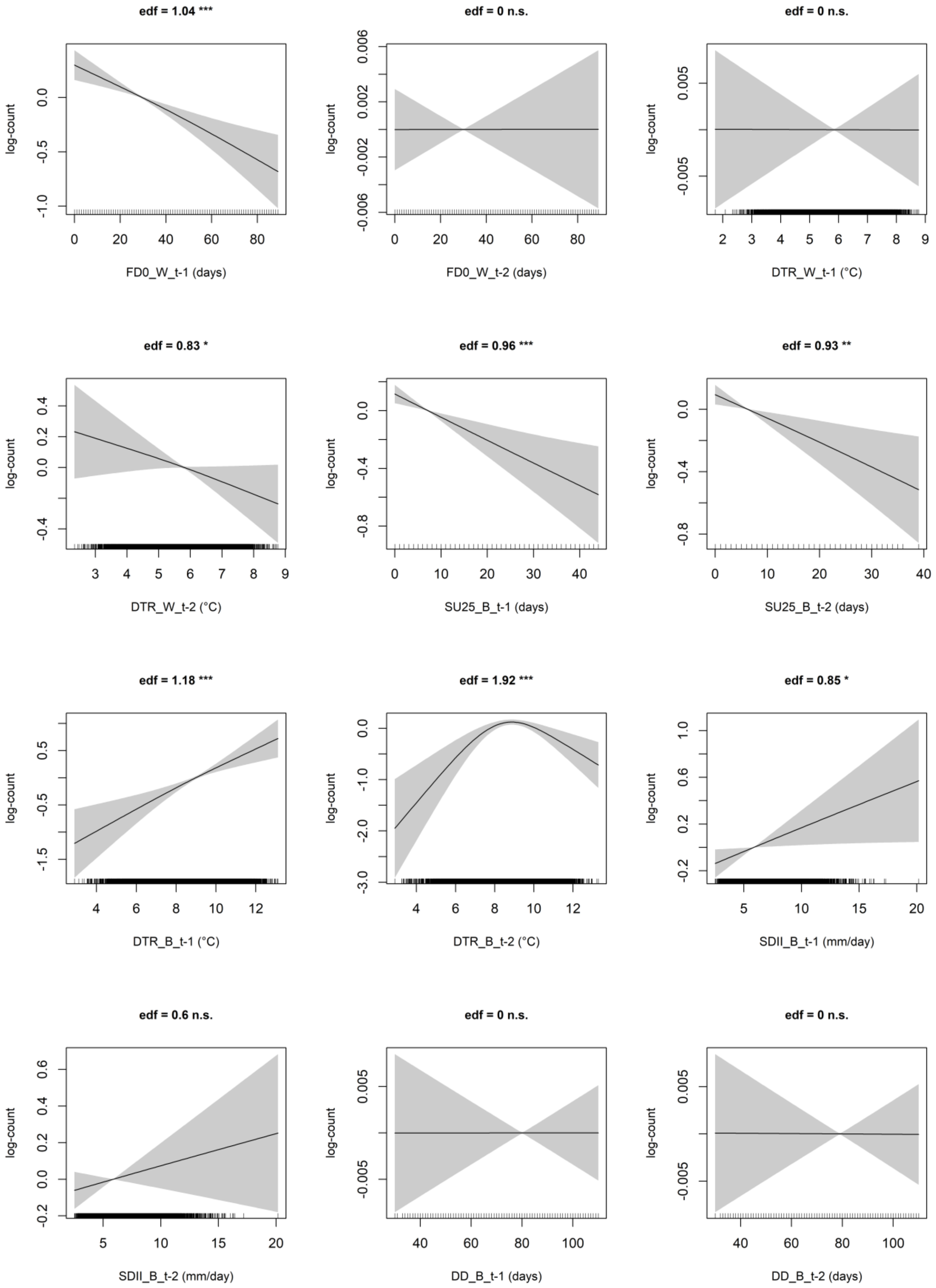

Short-eared Owl *Asio flammeus*

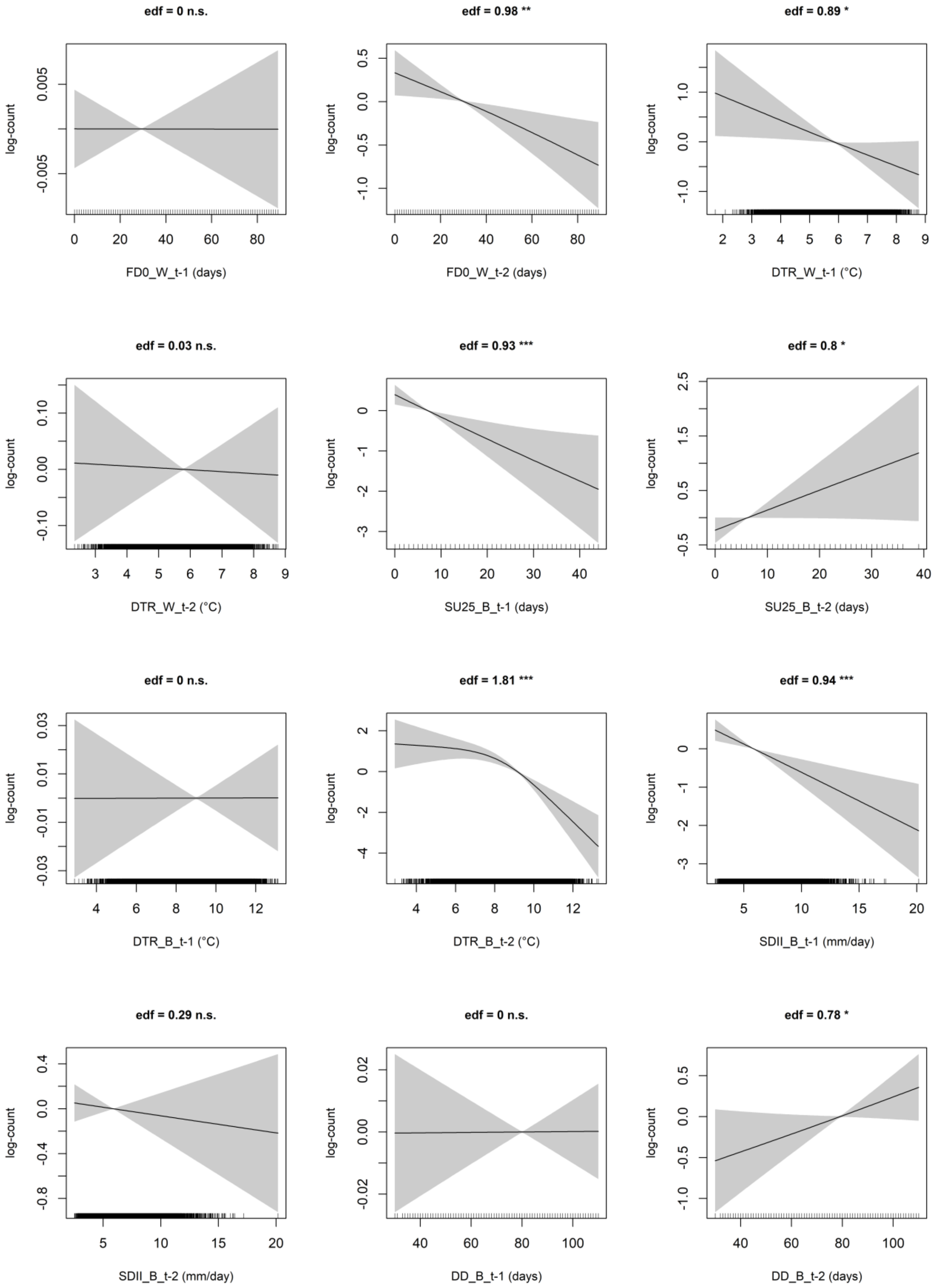

Common Kingfisher *Alcedo atthis*

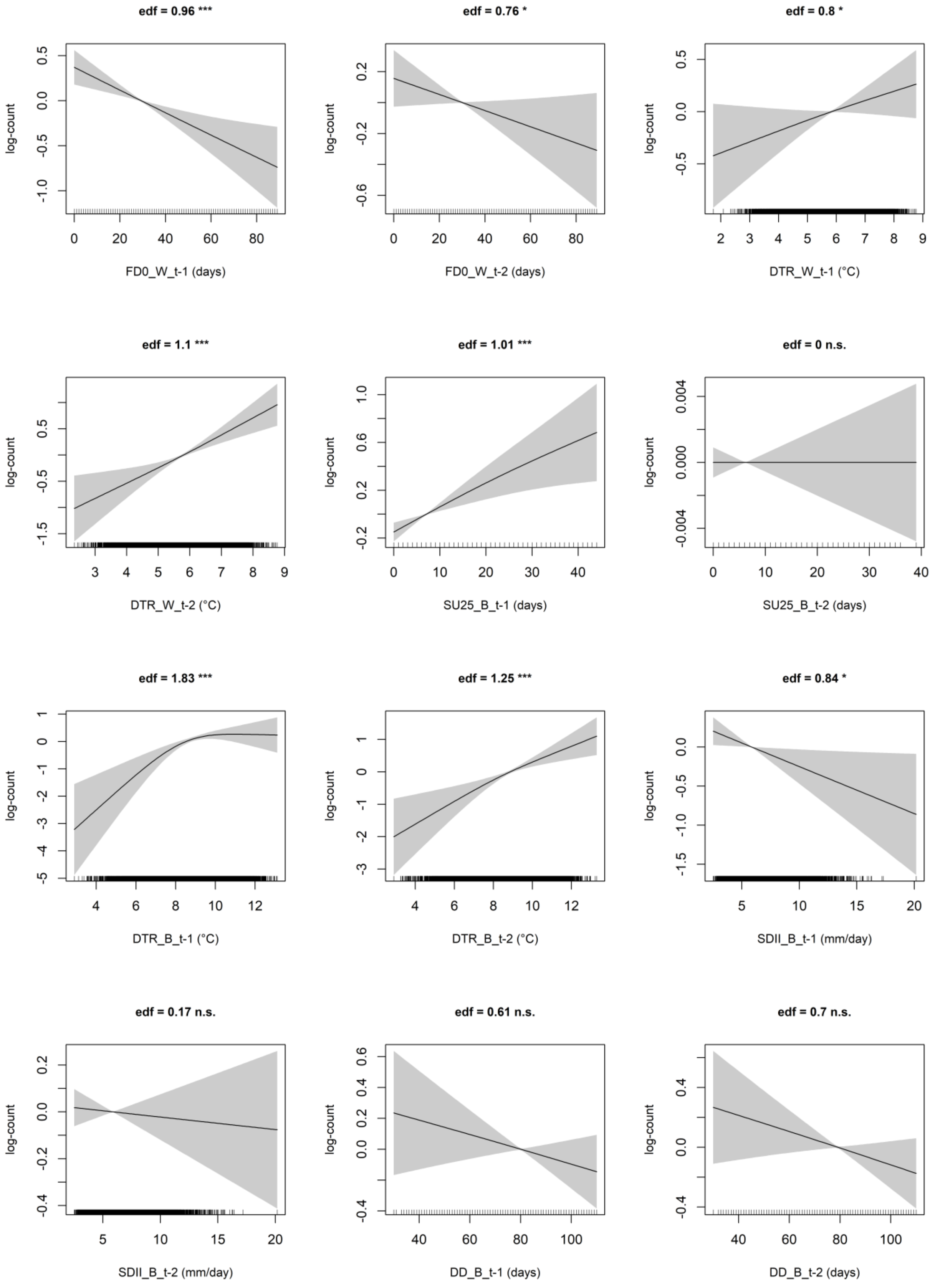

European Green Woodpecker *Picus viridis*

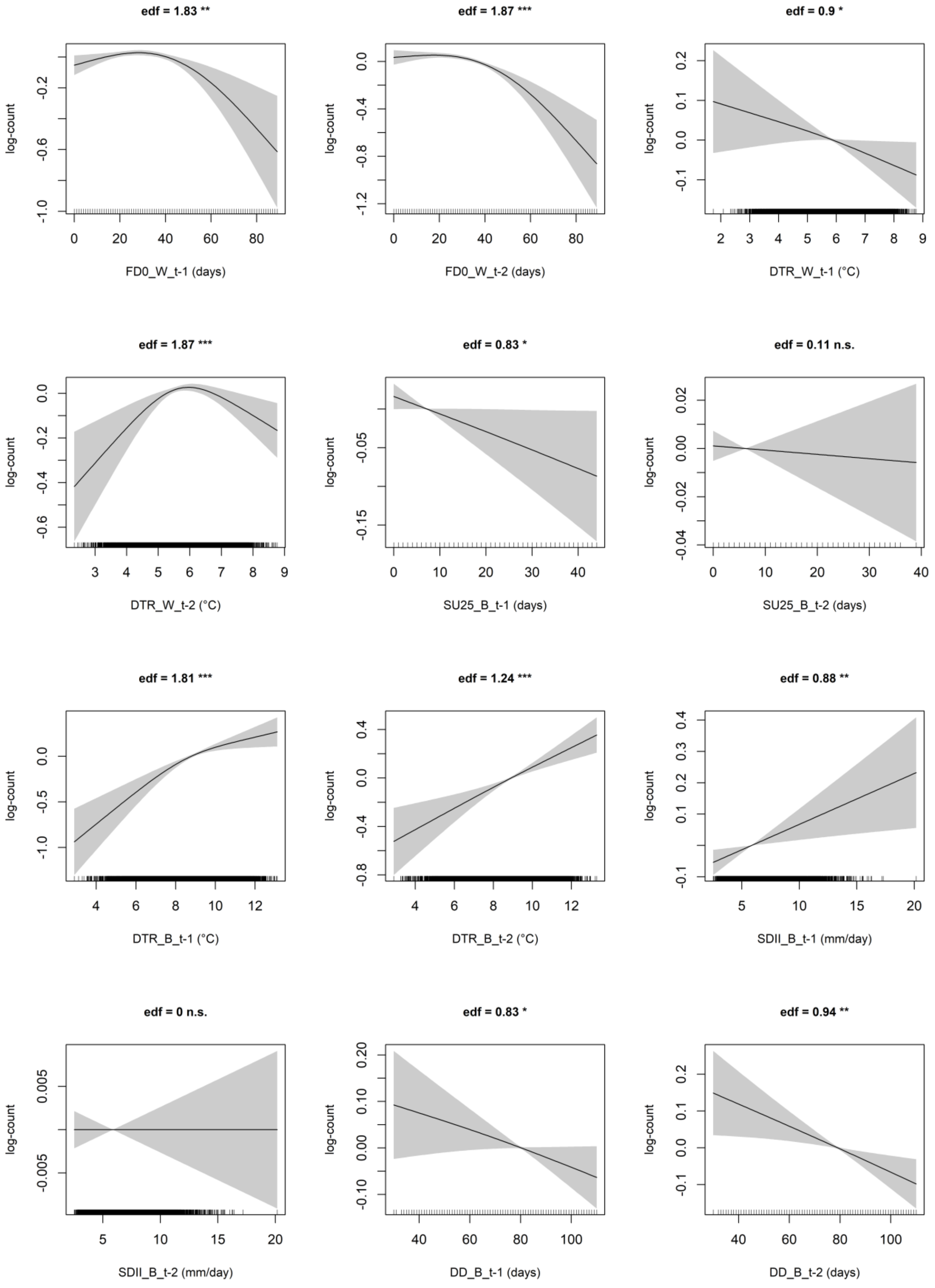

Great Spotted Woodpecker *Dendrocopos major*

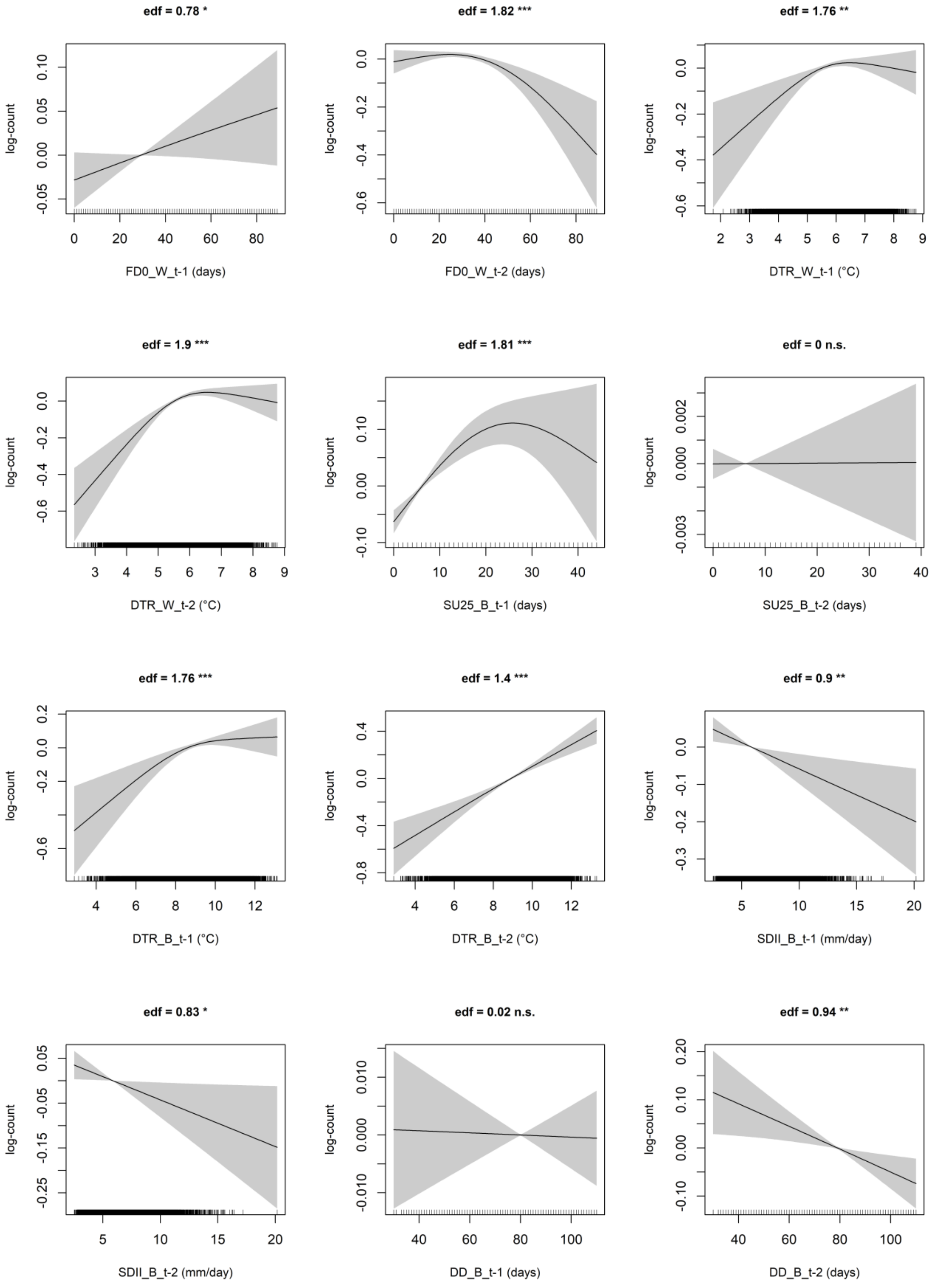

Lesser Spotted Woodpecker *Dryobates minor*

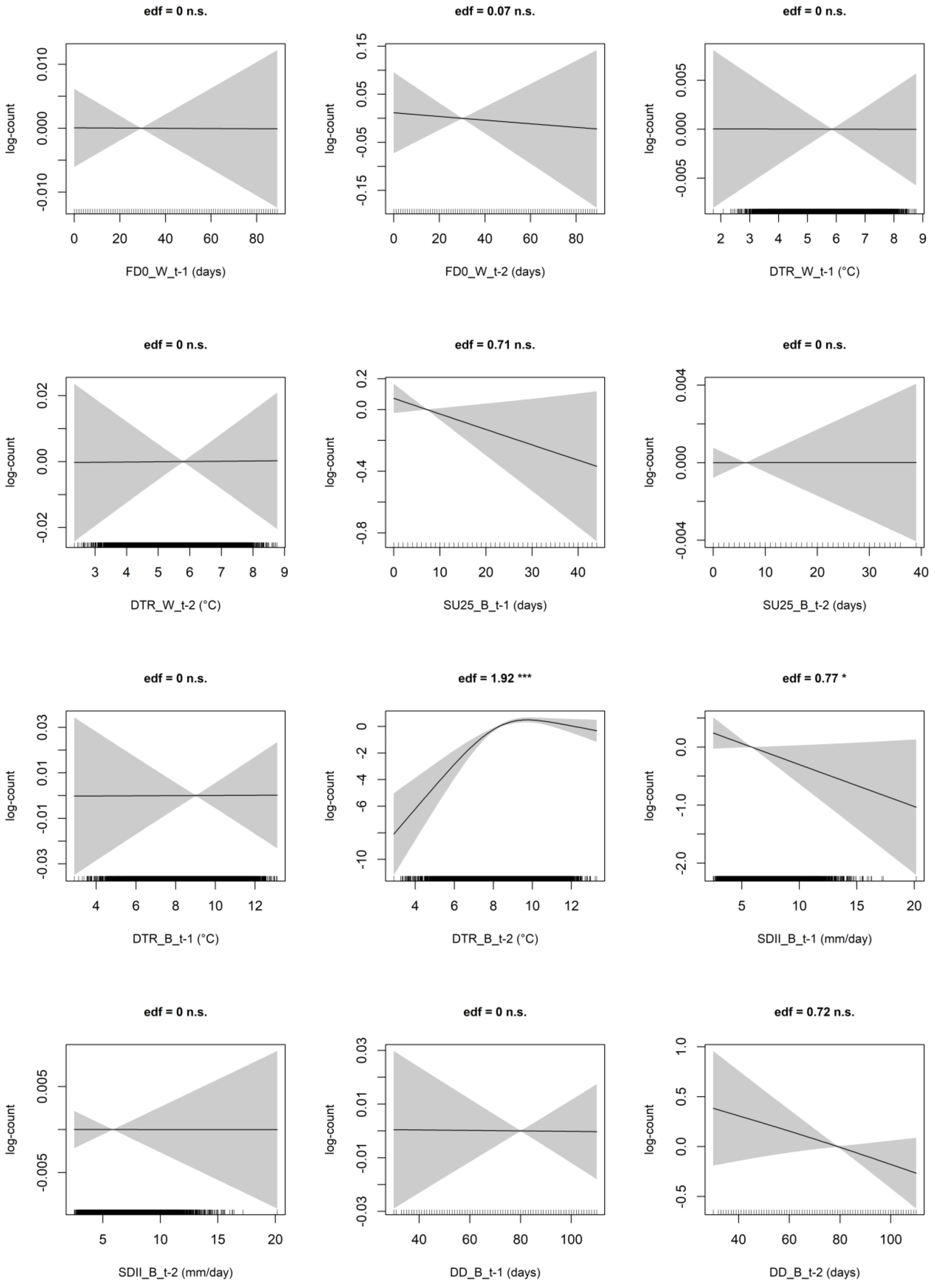

Eurasian Skylark *Alauda arvensis*

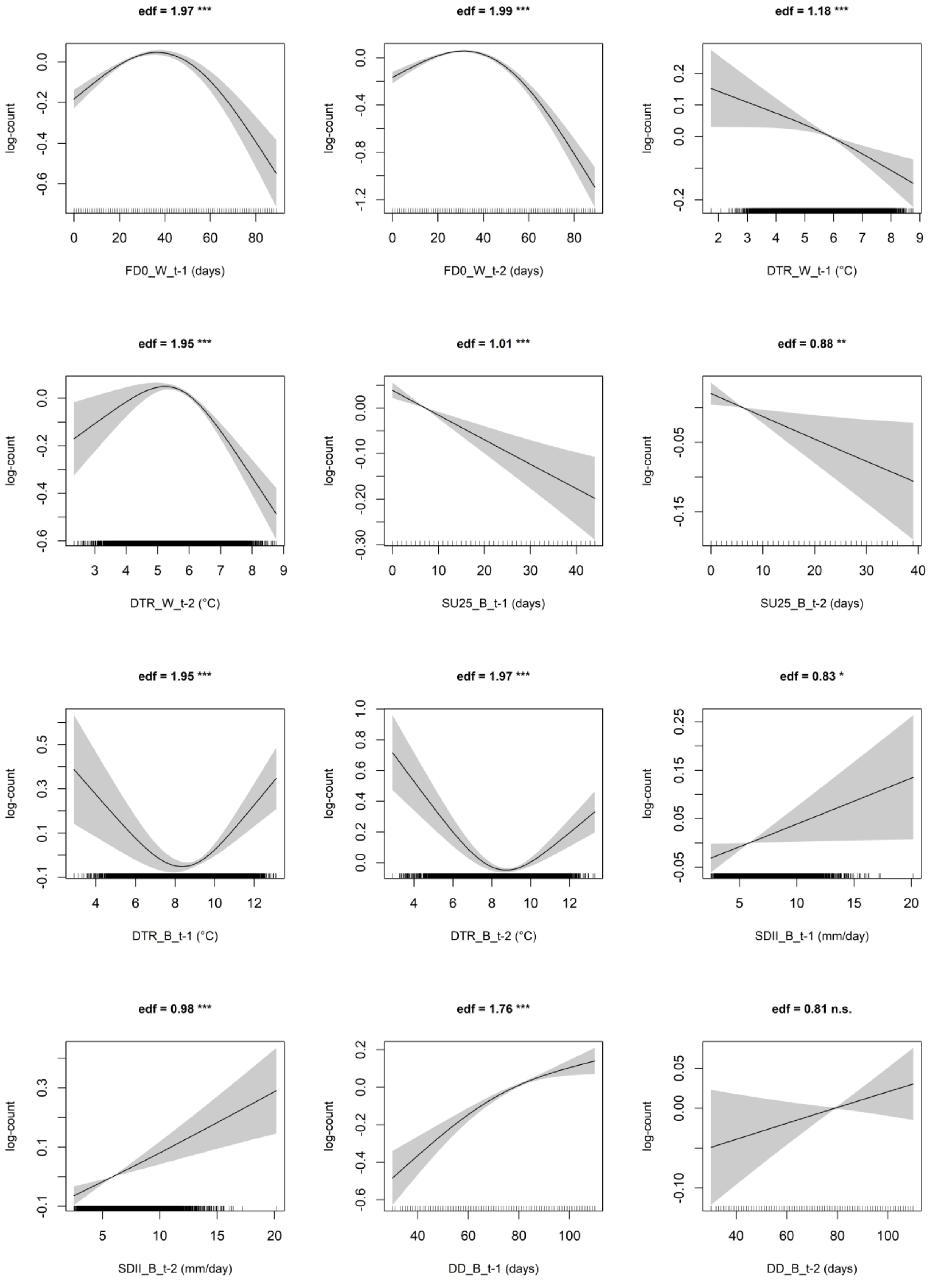

Meadow Pipit *Anthus pratensis*

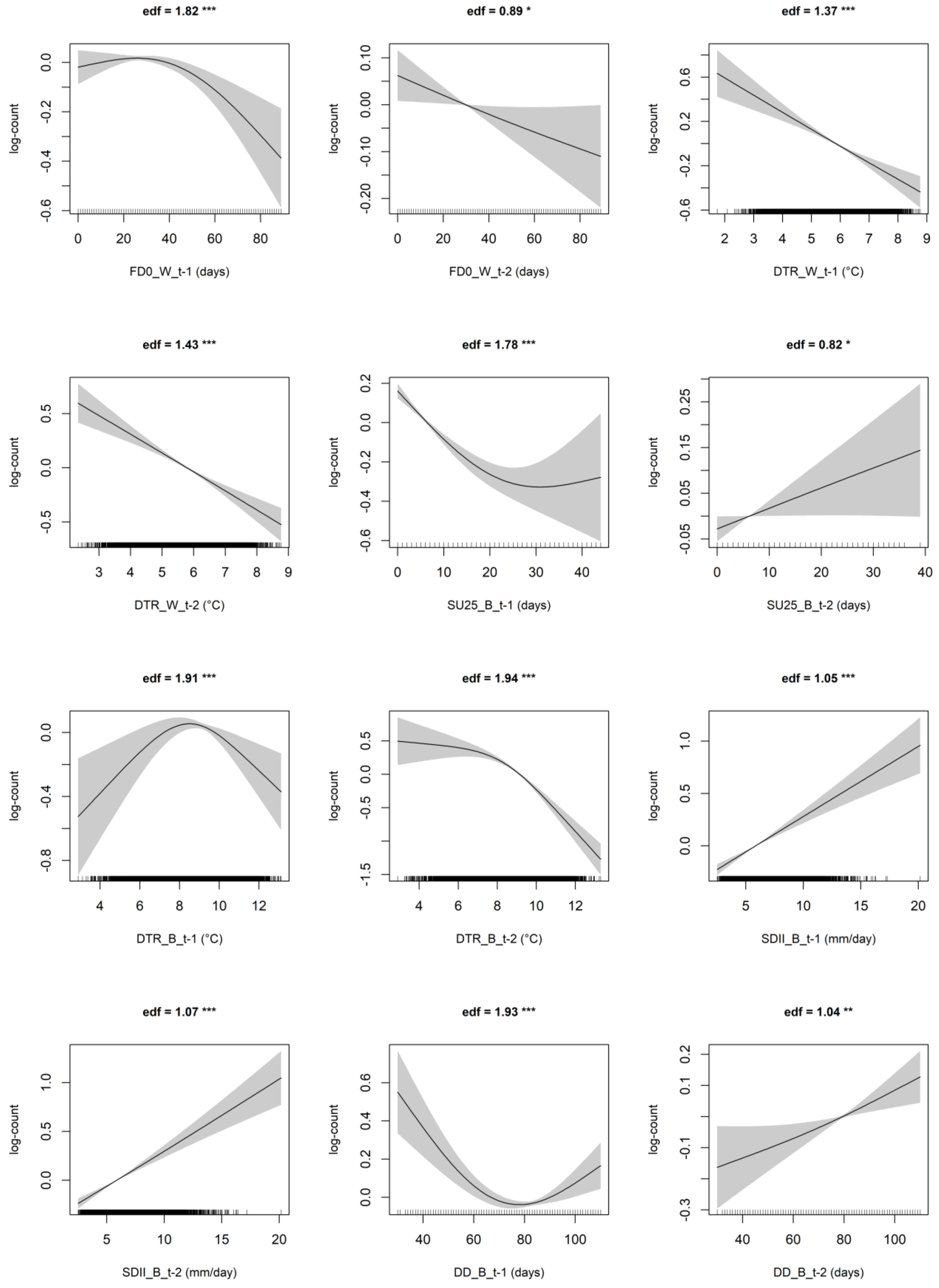

Grey Wagtail *Motacilla cinerea*

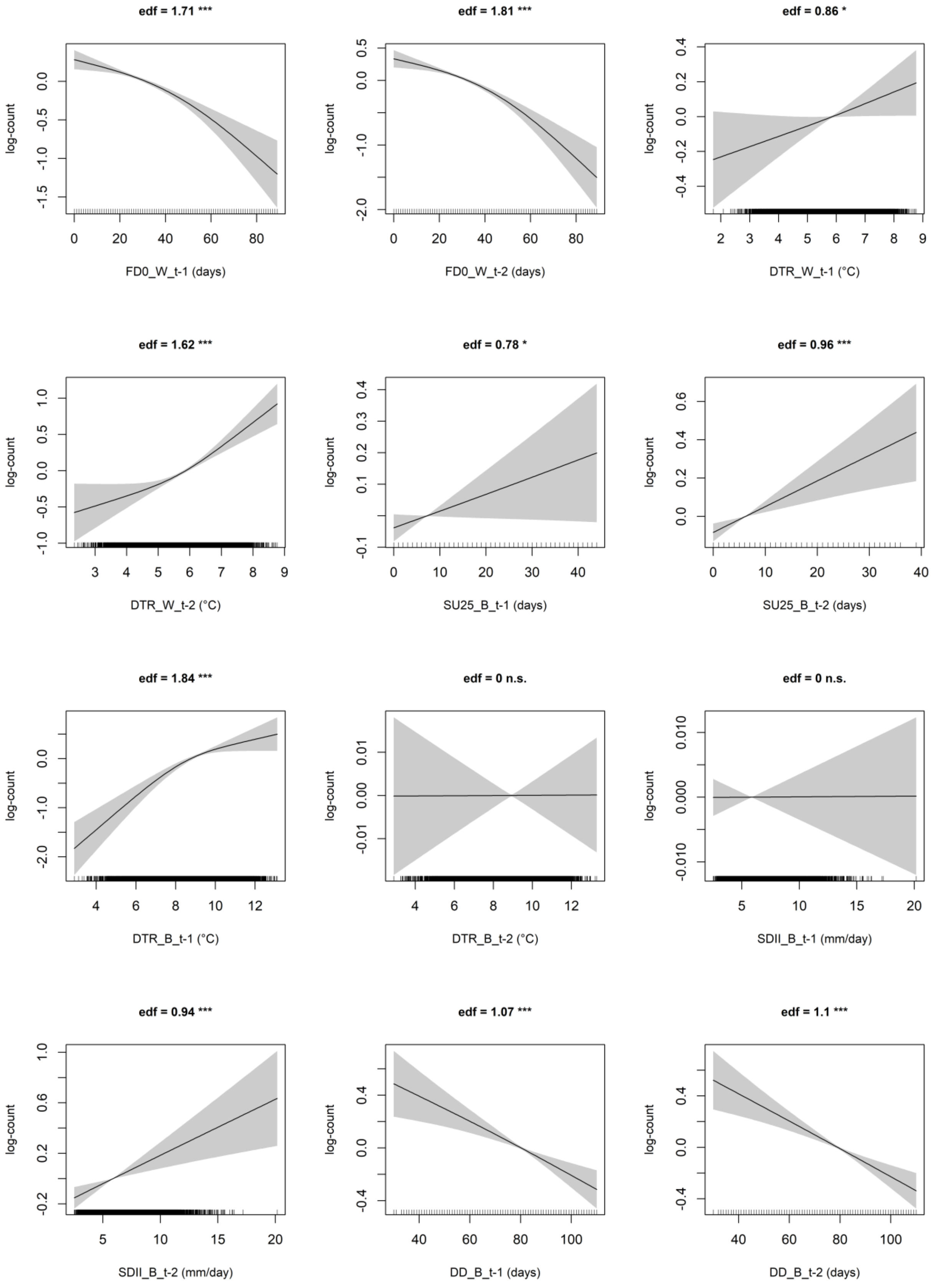

White Wagtail *Motacilla alba*

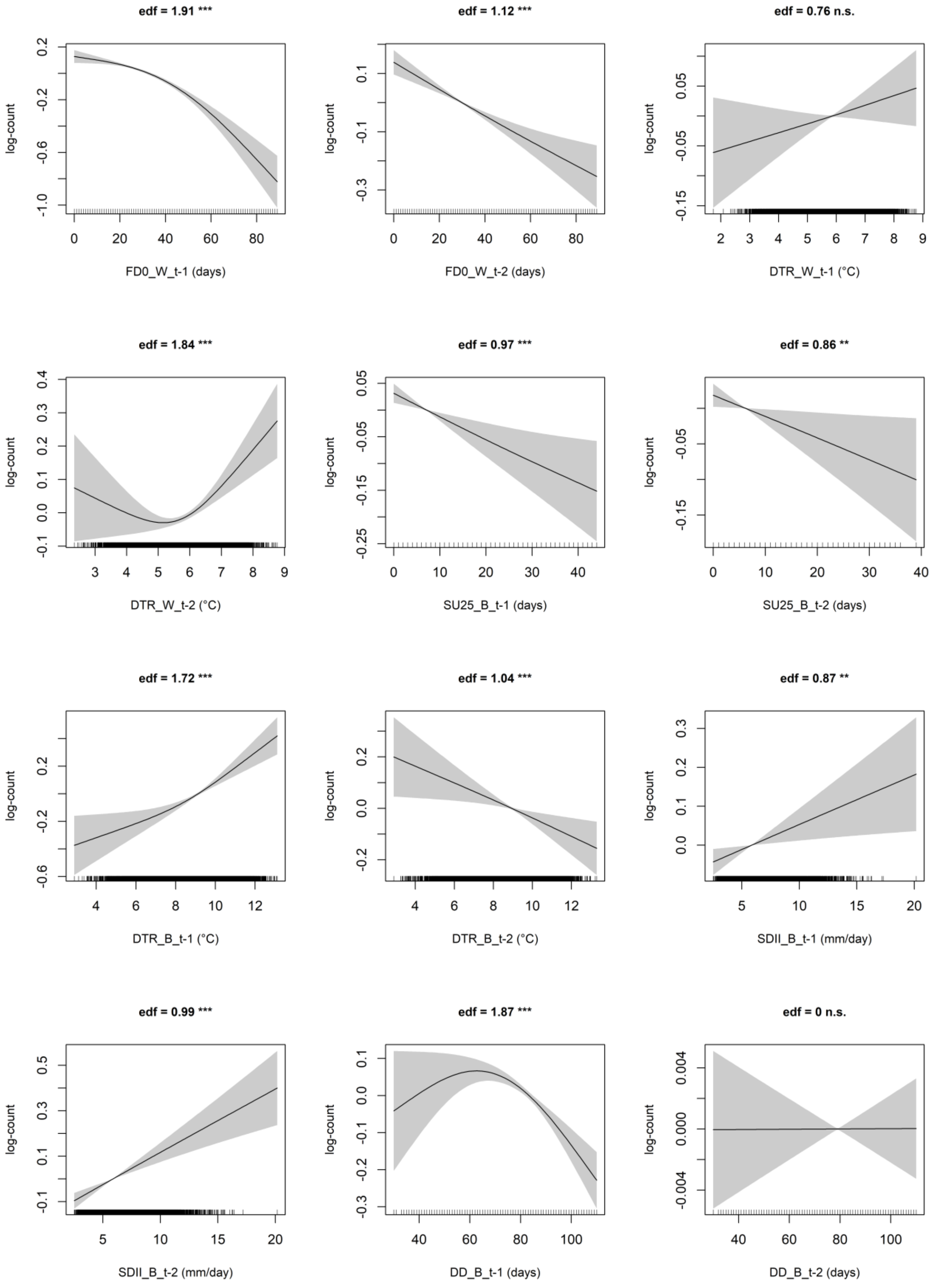

White-throated Dipper *Cinclus cinclus*

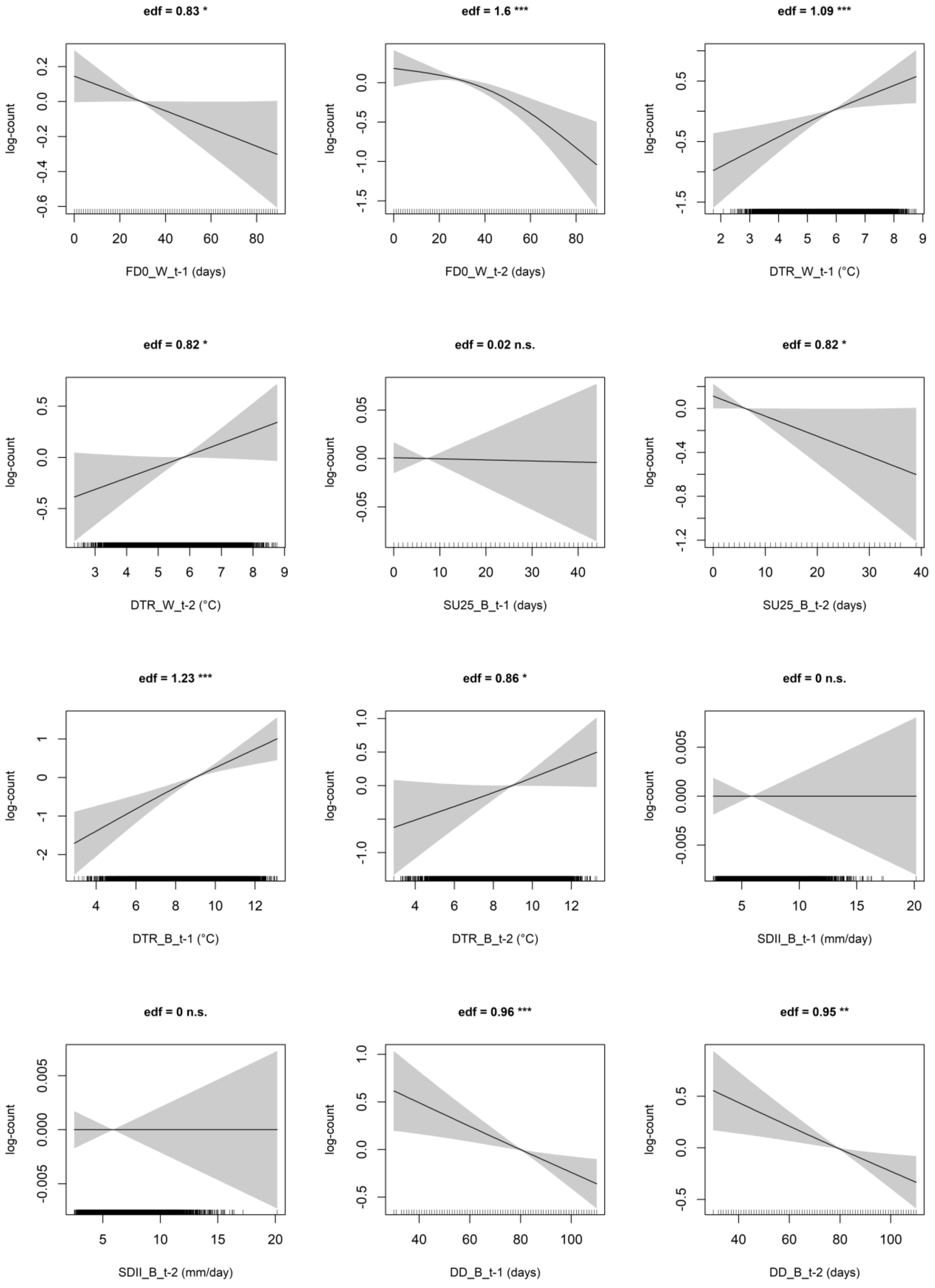

Eurasian Wren *Troglodytes troglodytes*

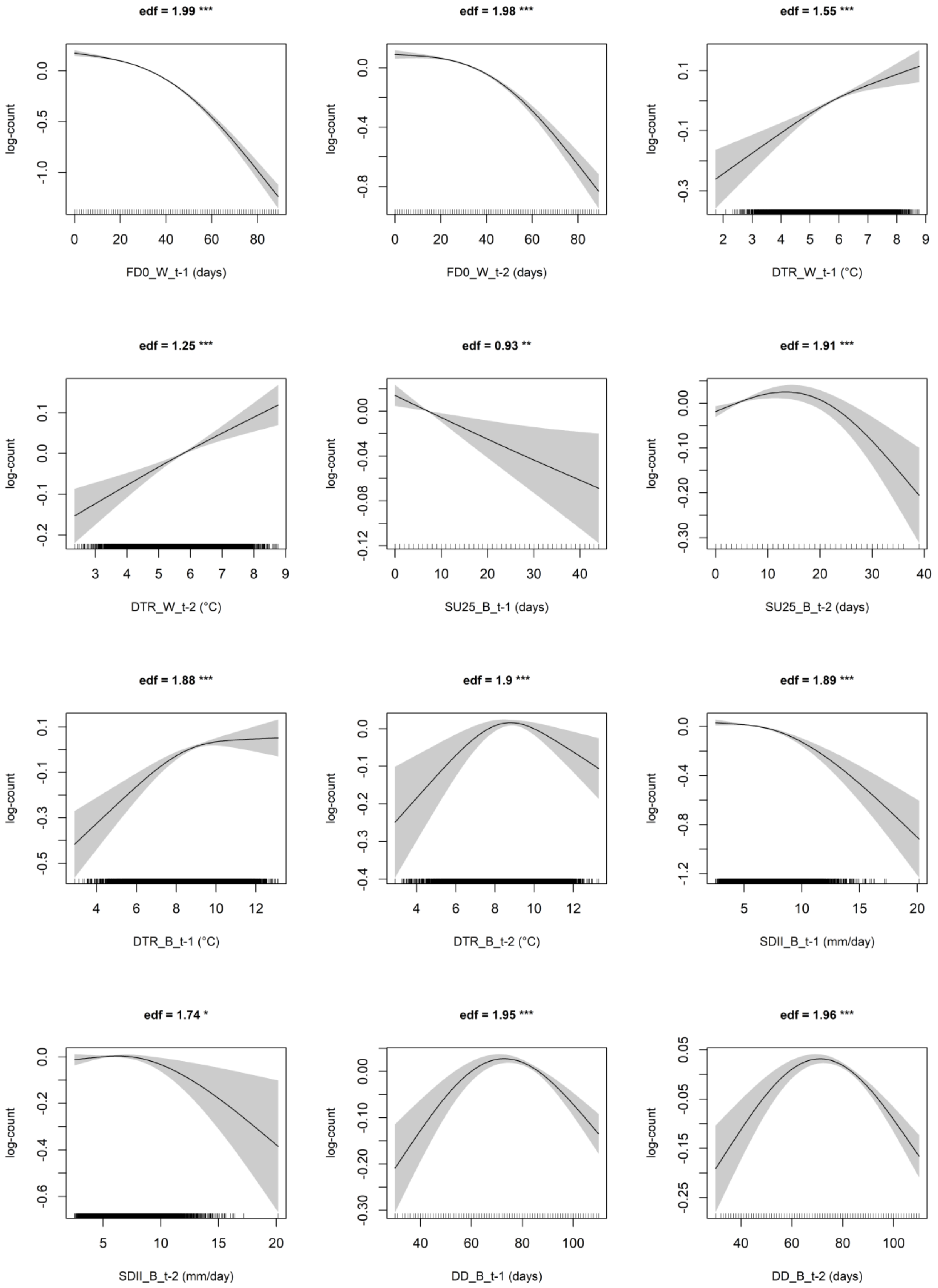

Dunnock *Prunella modularis*

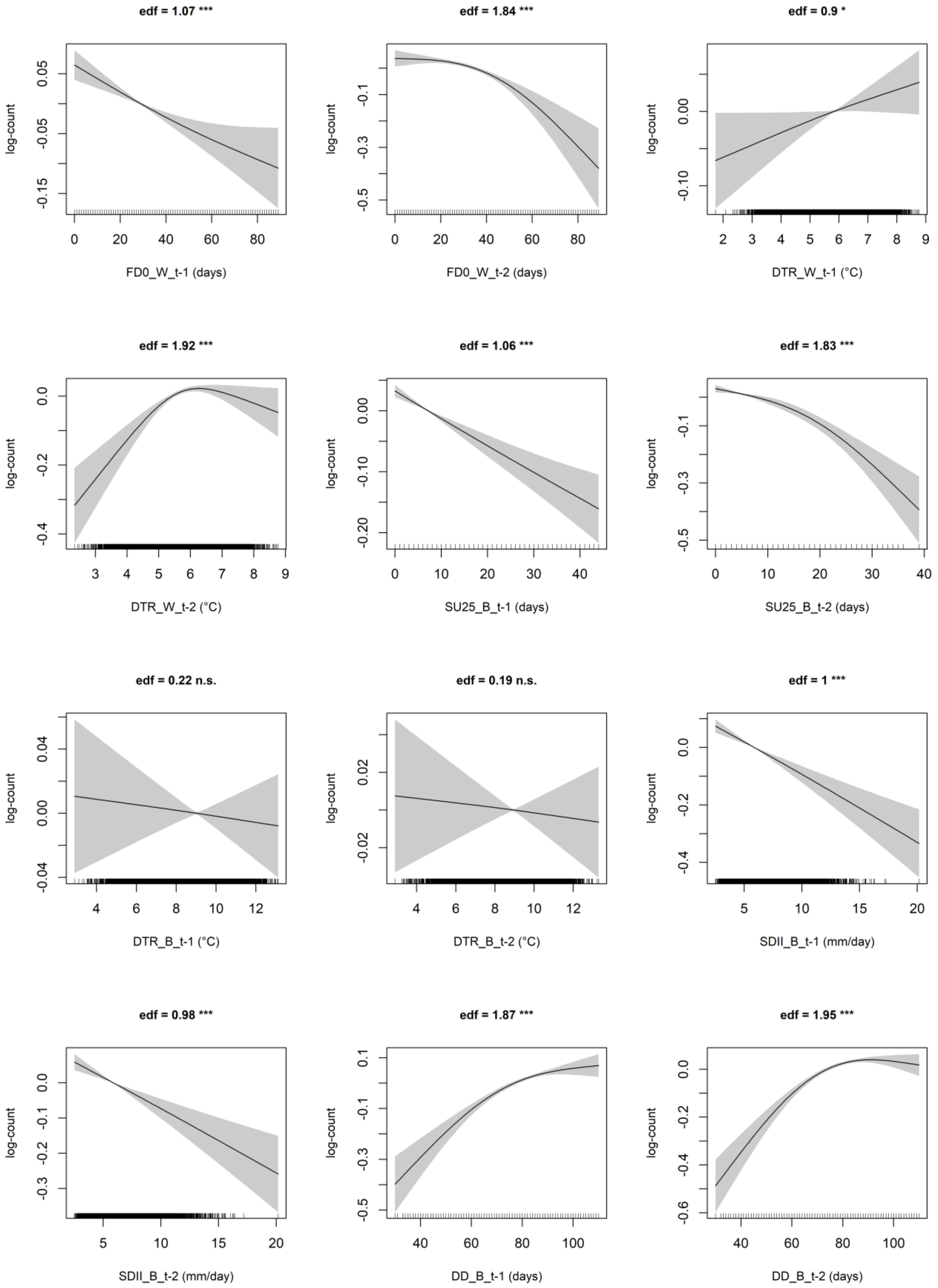

European Robin *Erithacus rubecula*

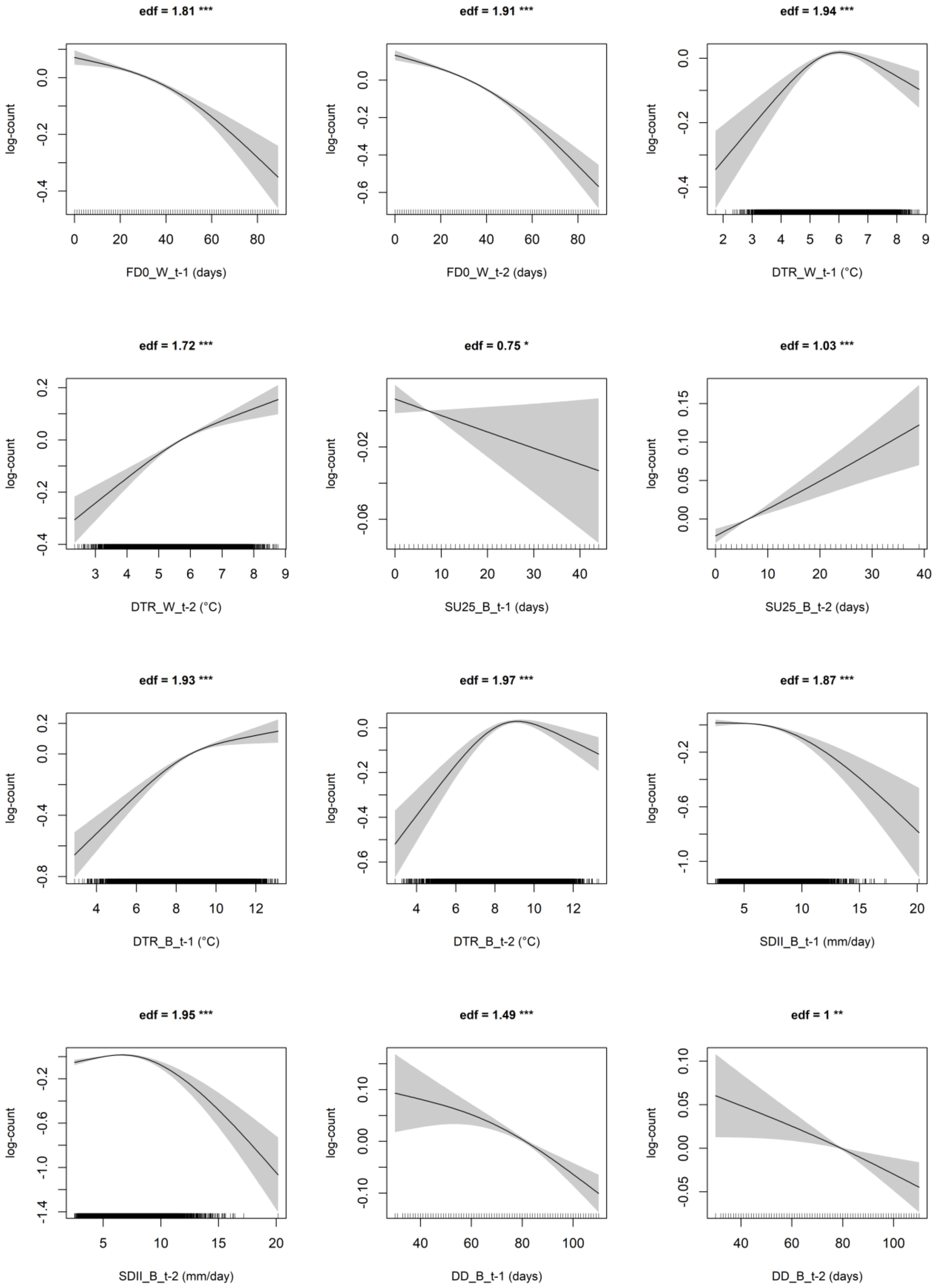

European Stonechat *Saxicola rubicola*

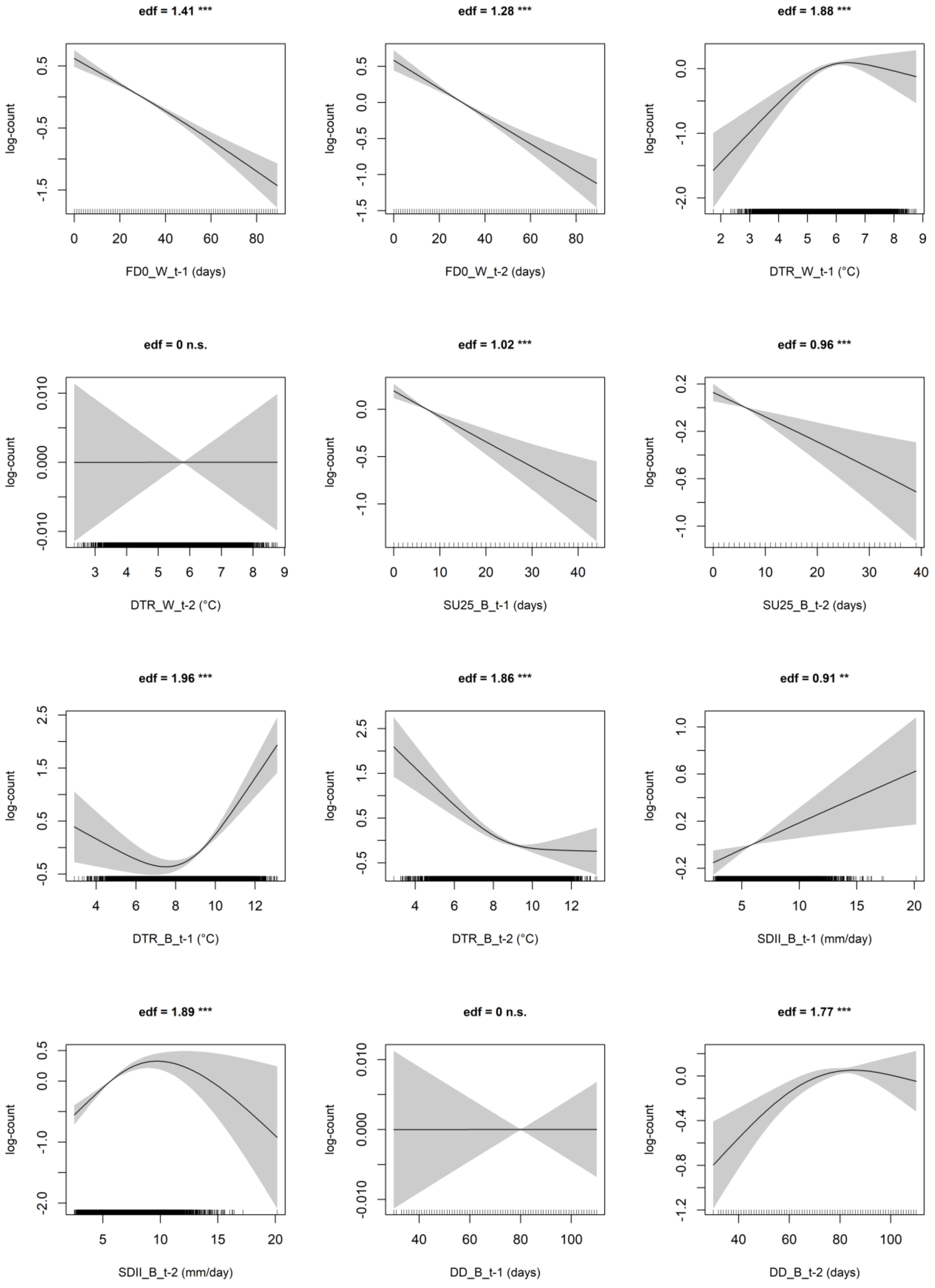

Common Blackbird *Turdus merula*

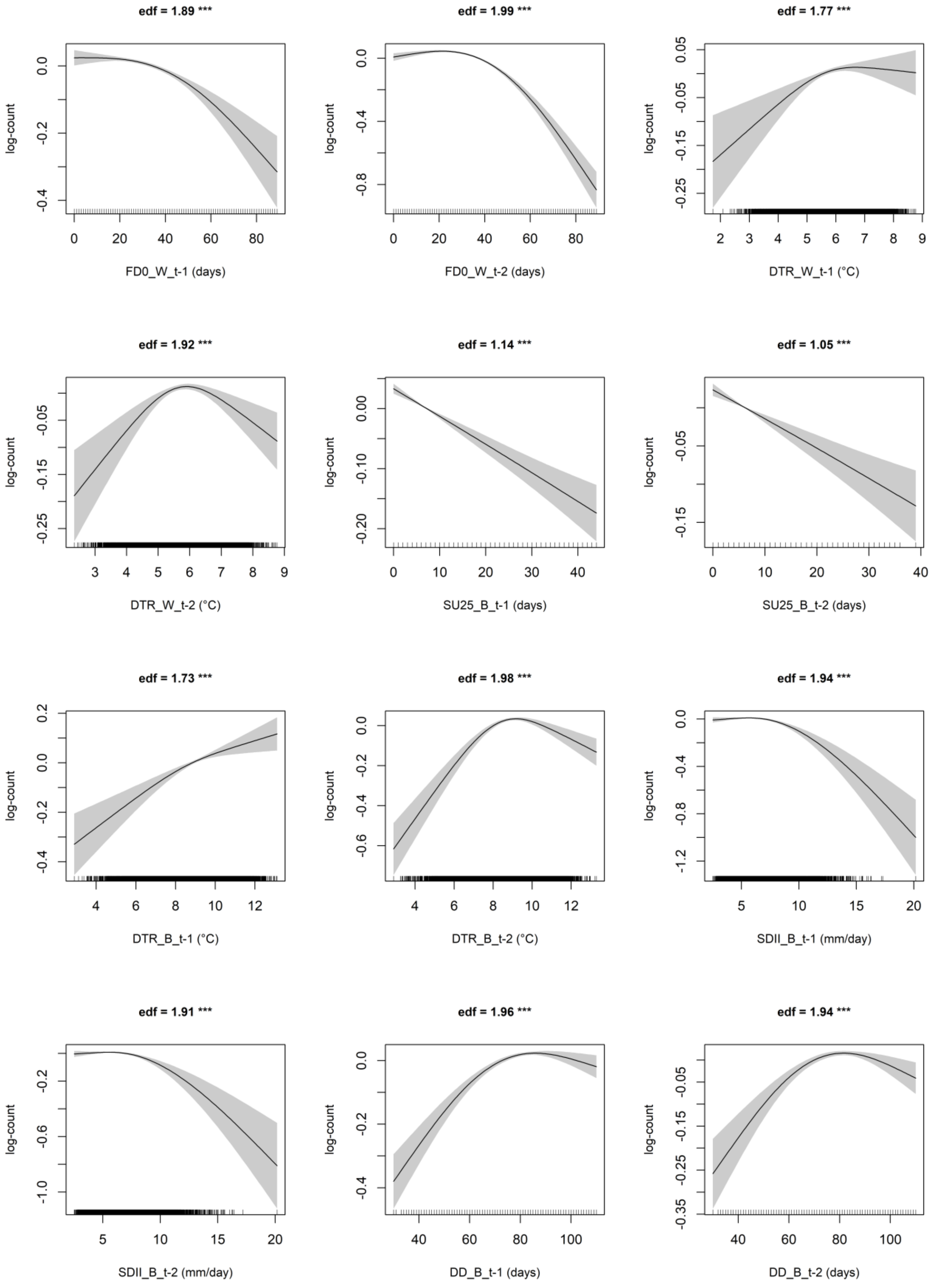

Fieldfare *Turdus pilaris*

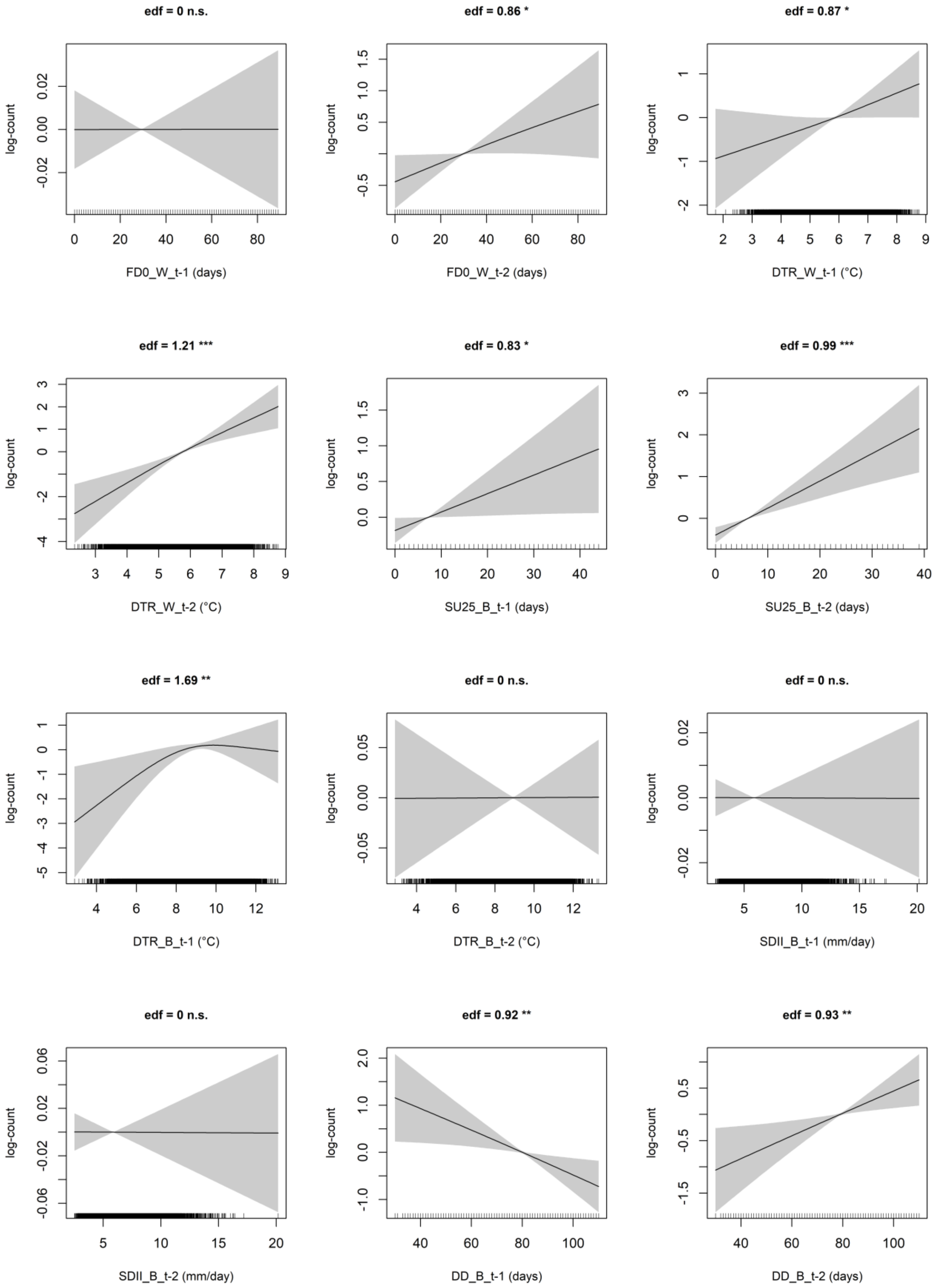

Song Thrush *Turdus philomelos*

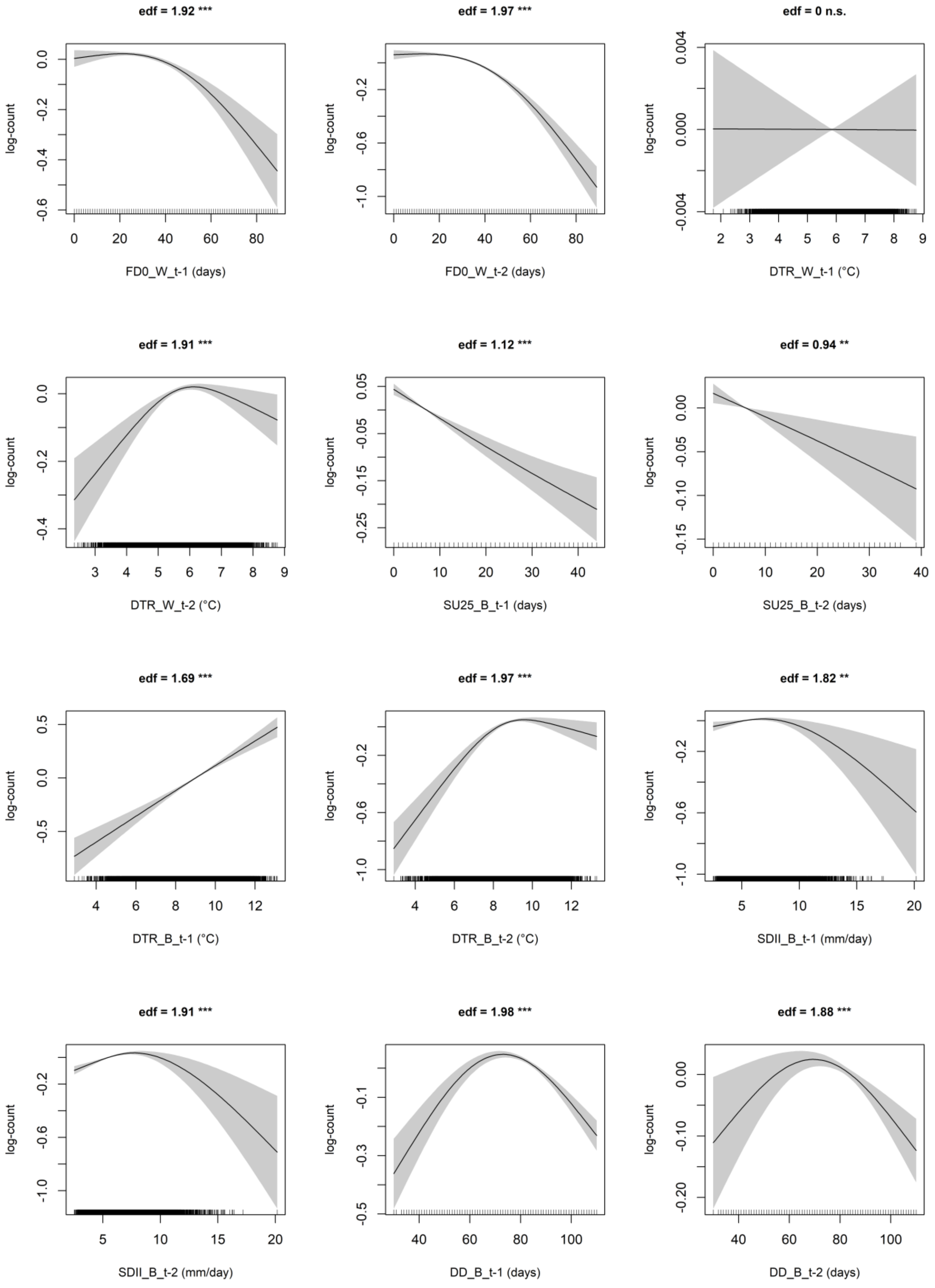

Redwing *Turdus iliacus*

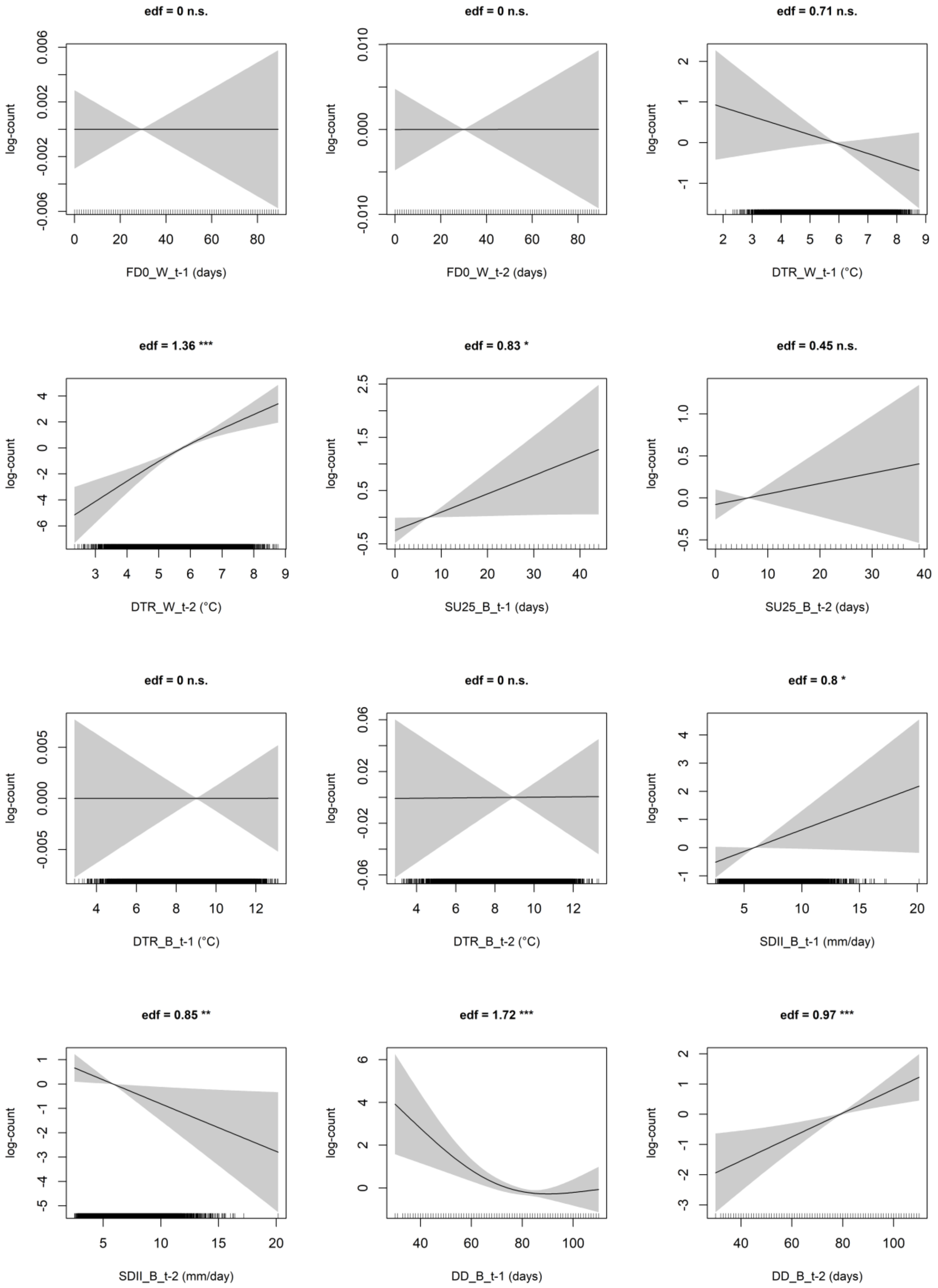

Mistle Thrush *Turdus viscivorus*

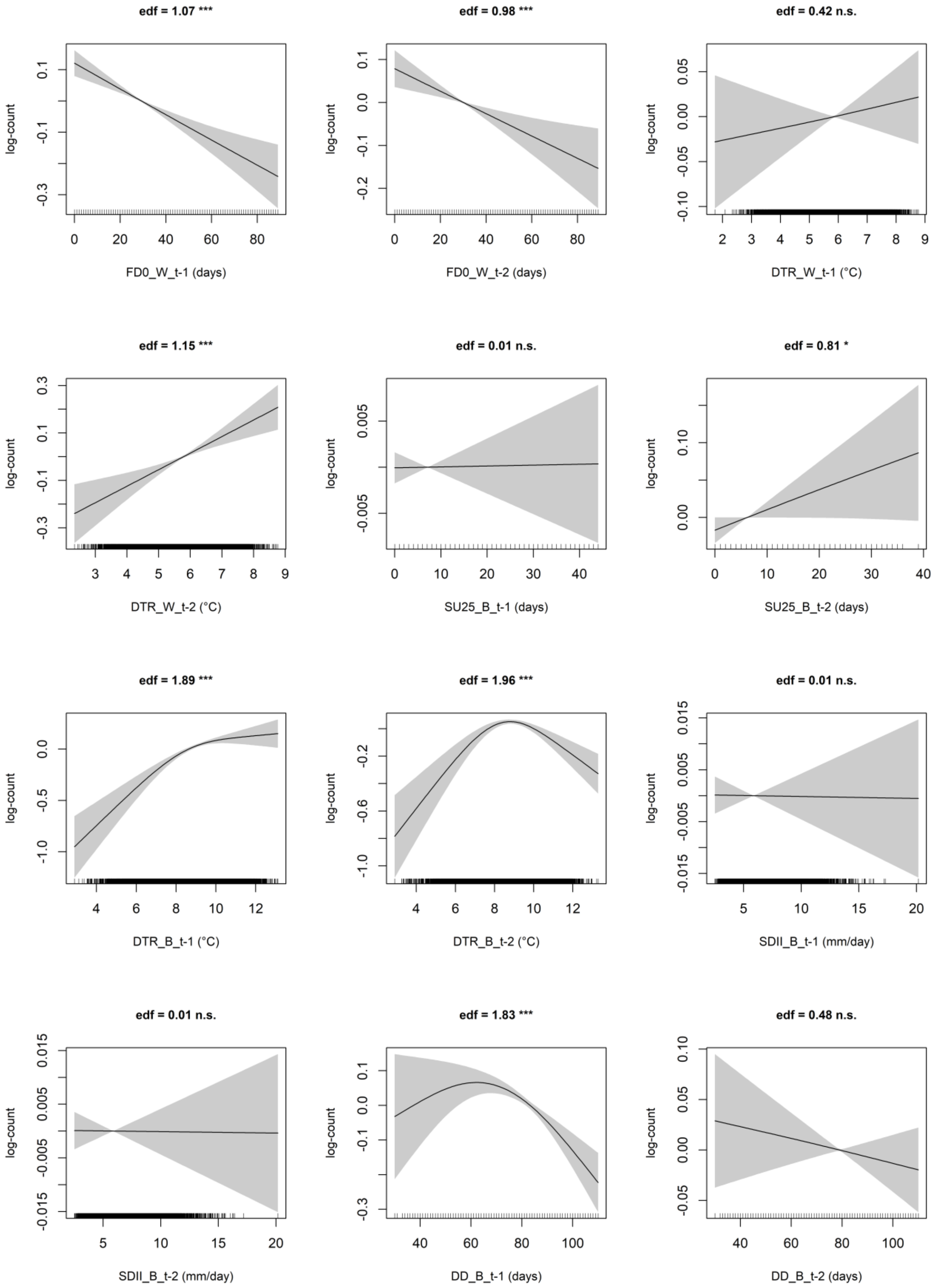

Cetti's Warbler *Cettia cetti*

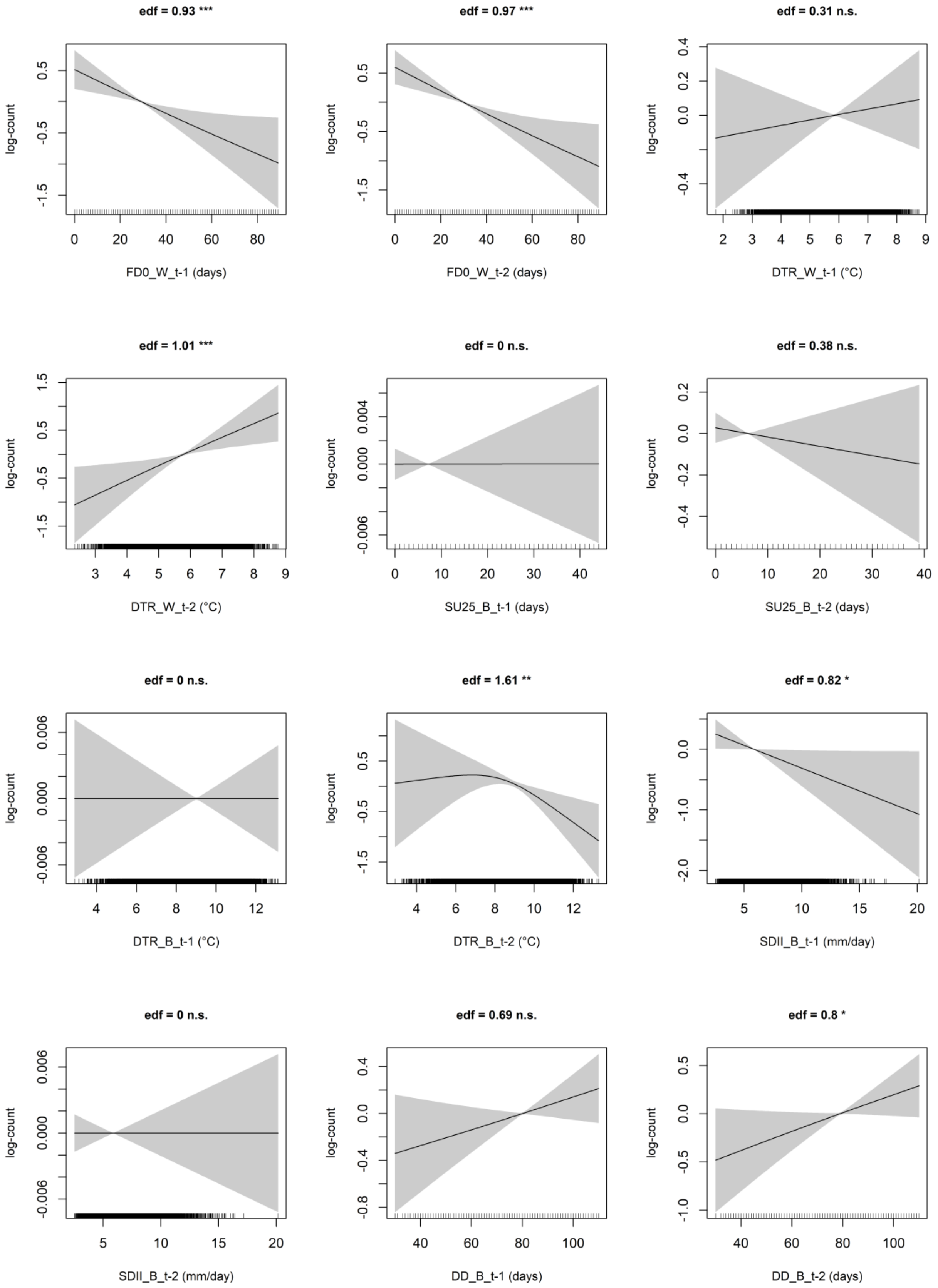

Goldcrest *Regulus regulus*

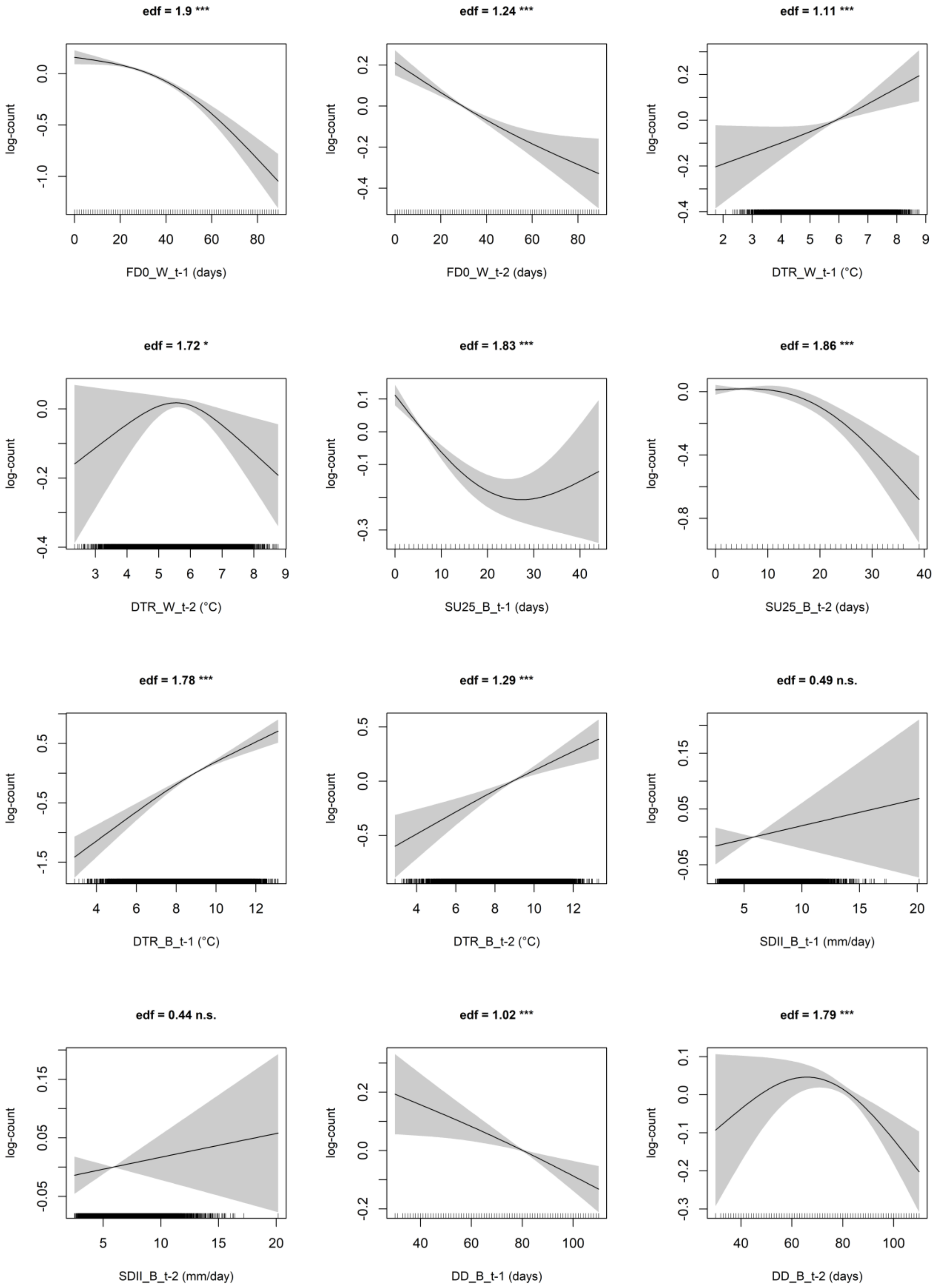

Long-tailed Tit *Aegithalos caudatus*

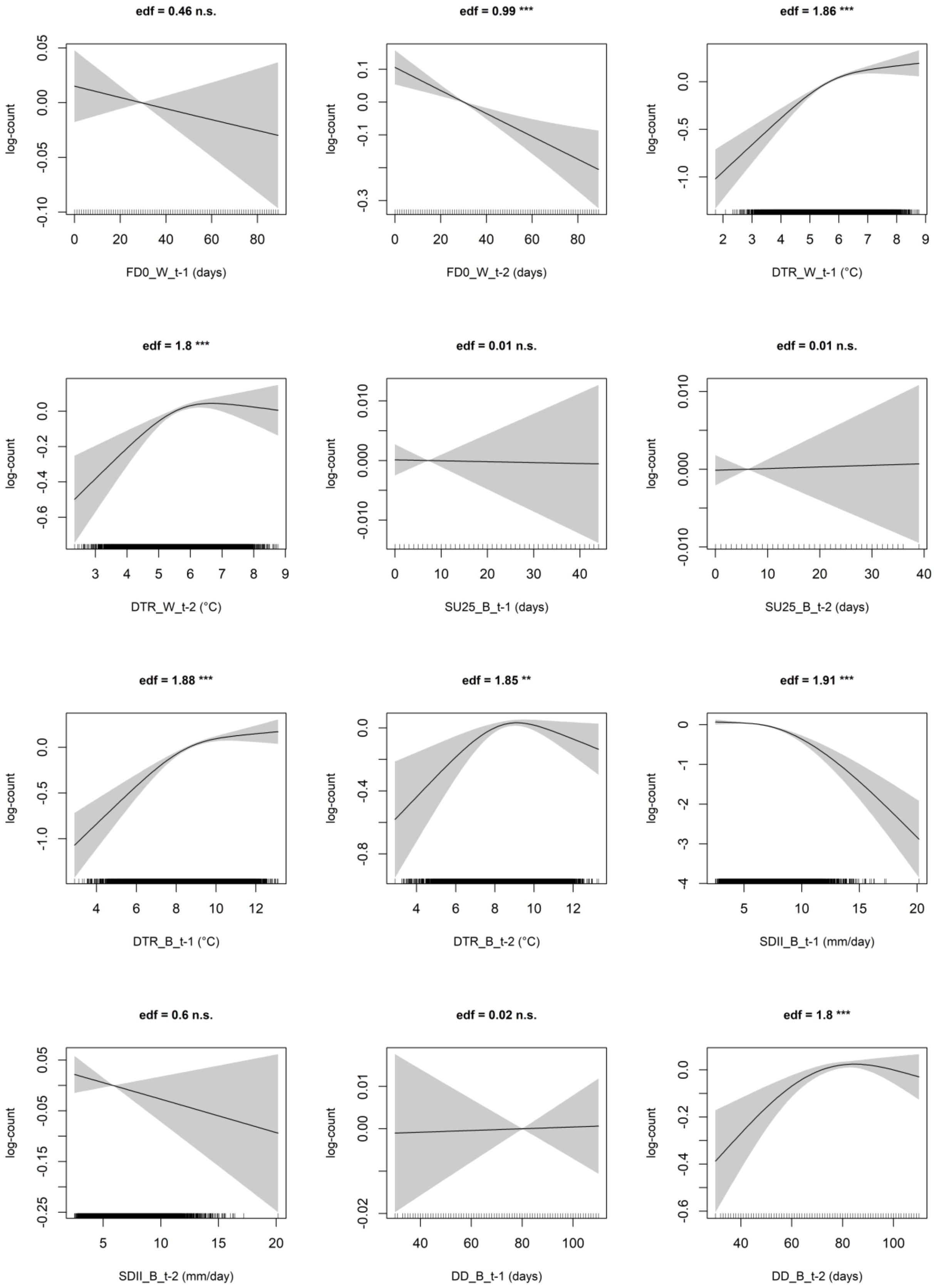

Marsh Tit *Poecile palustris*

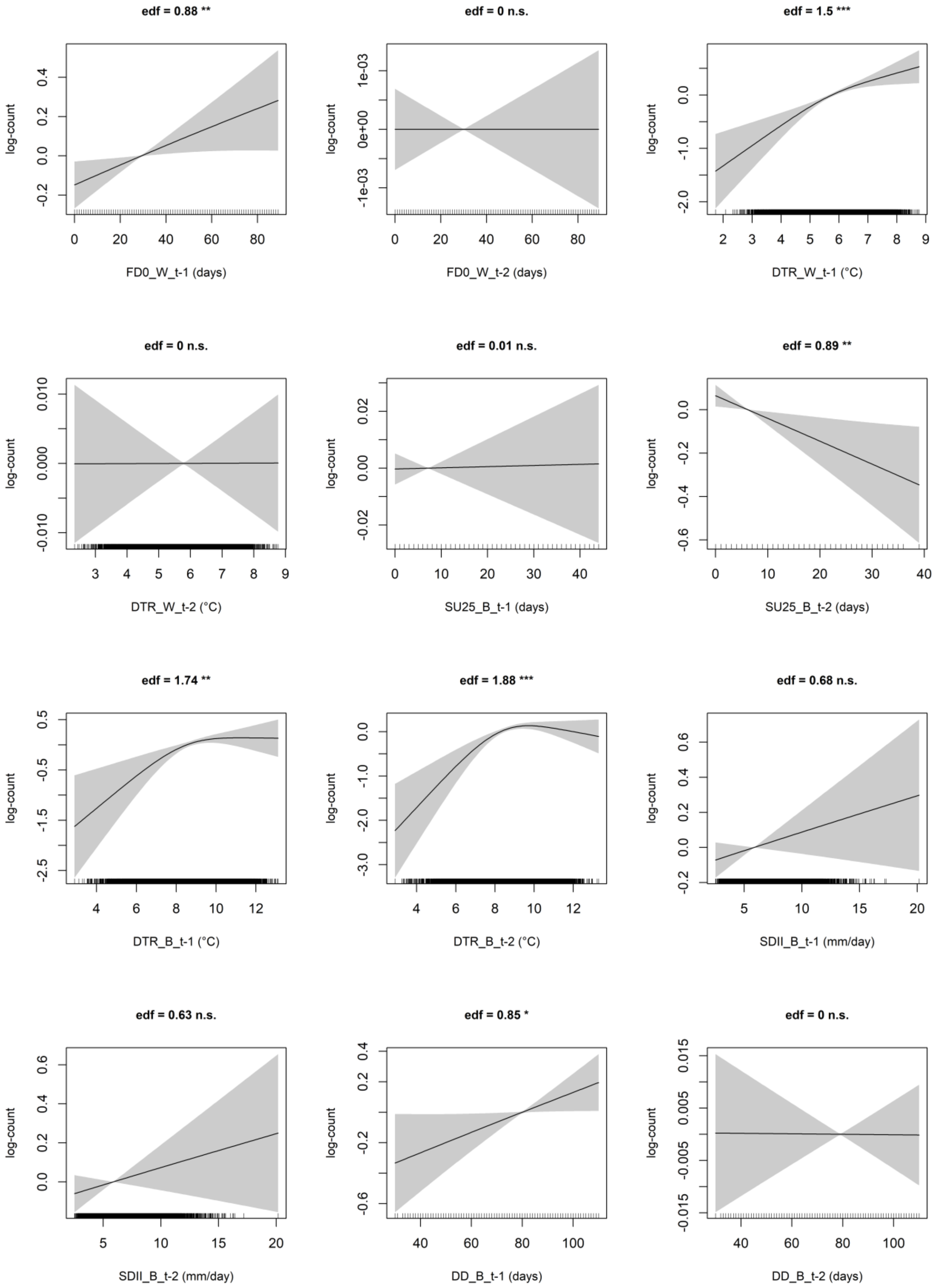

Willow Tit *Poecile montanus*

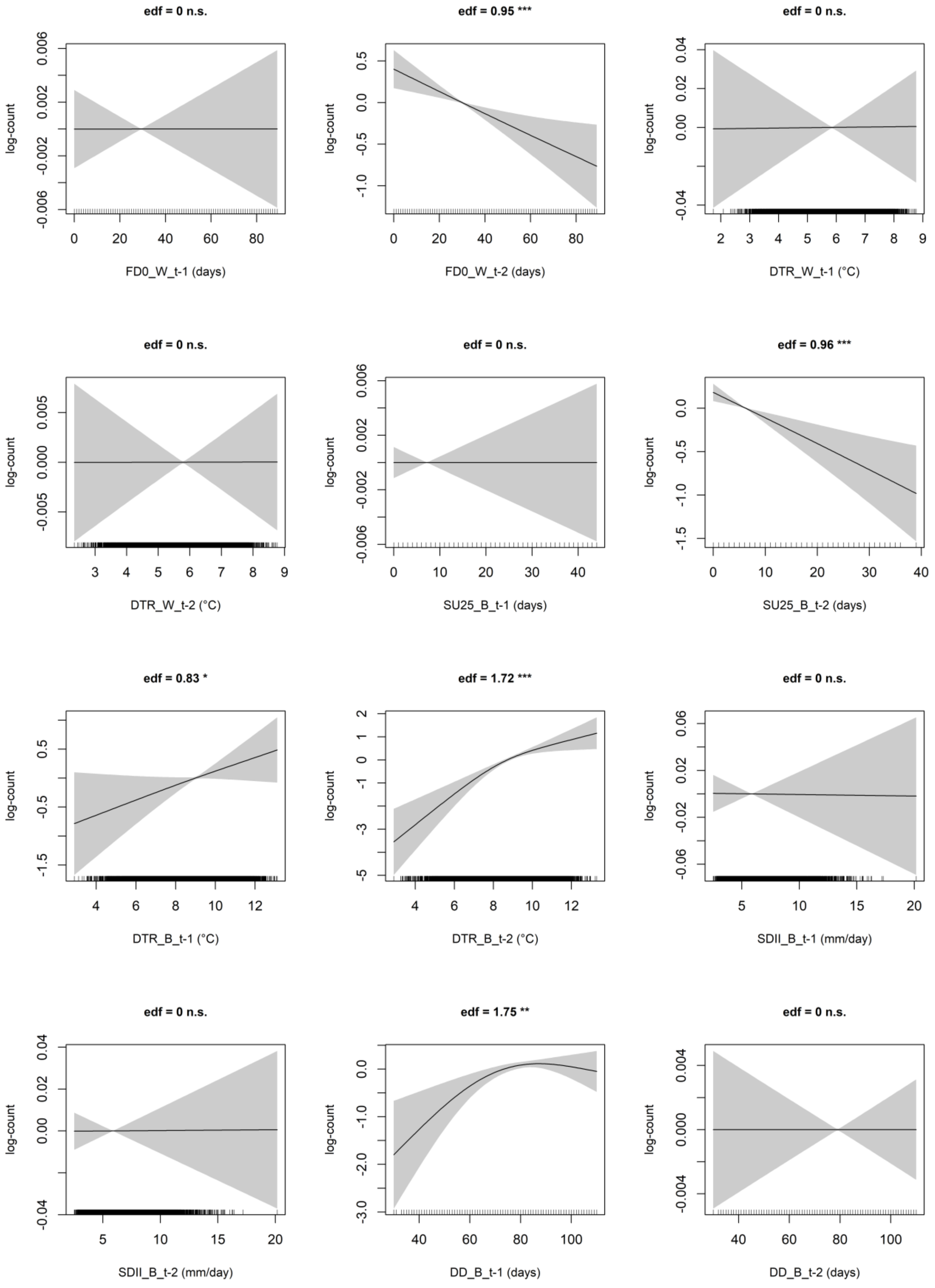

Coal Tit *Periparus ater*

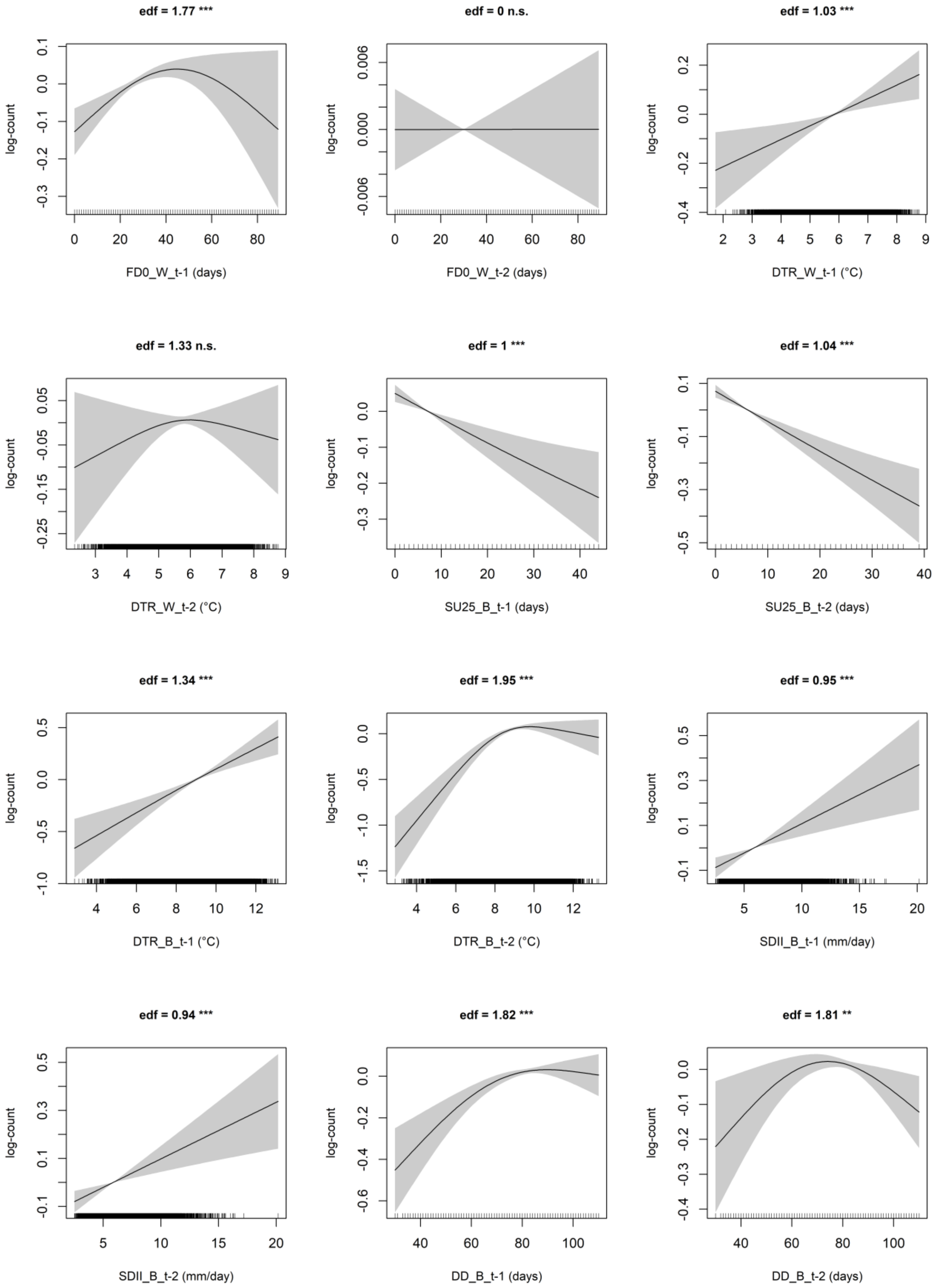

Eurasian Blue Tit *Cyanistes caeruleus*

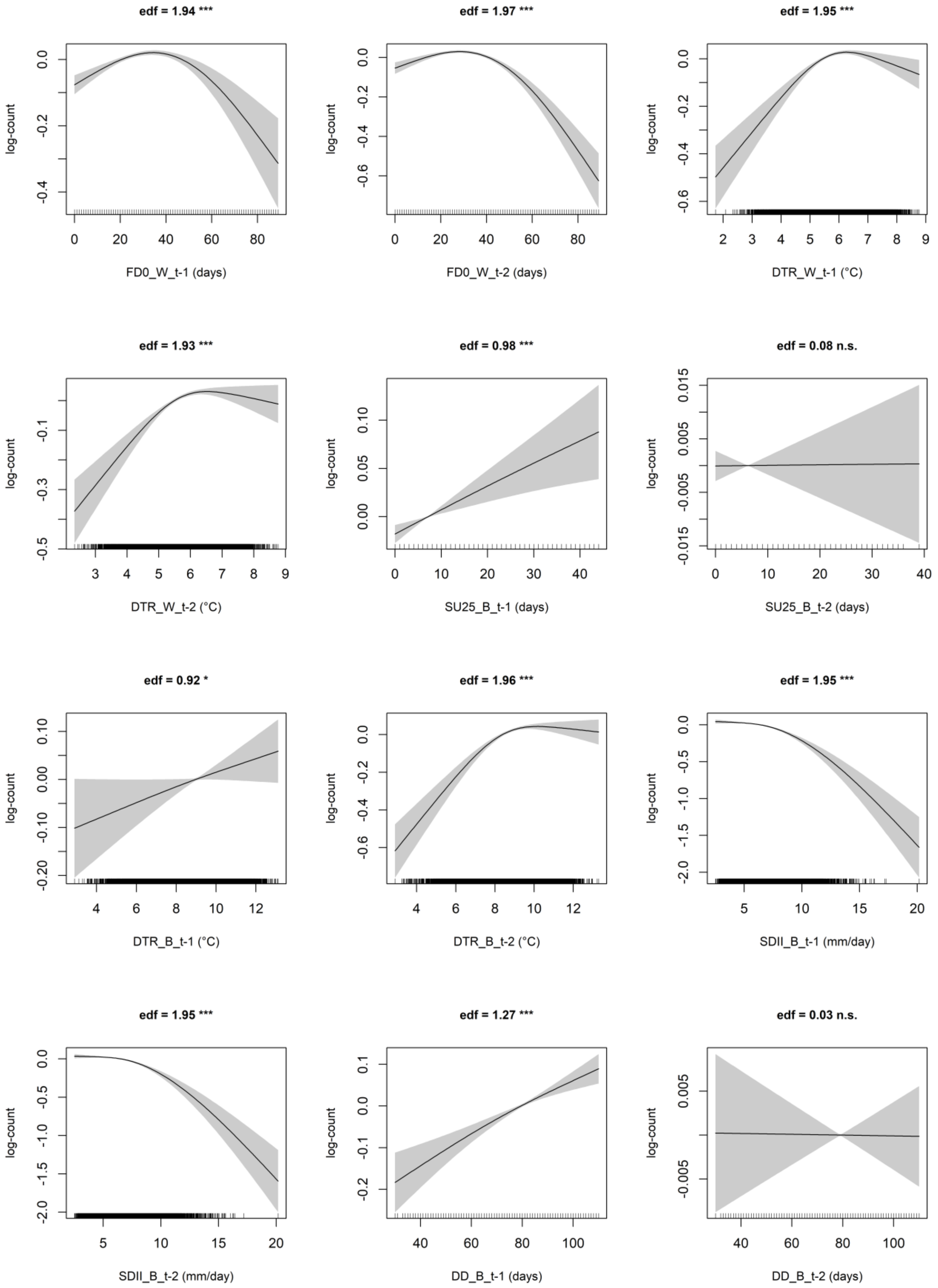

Great Tit *Parus major*

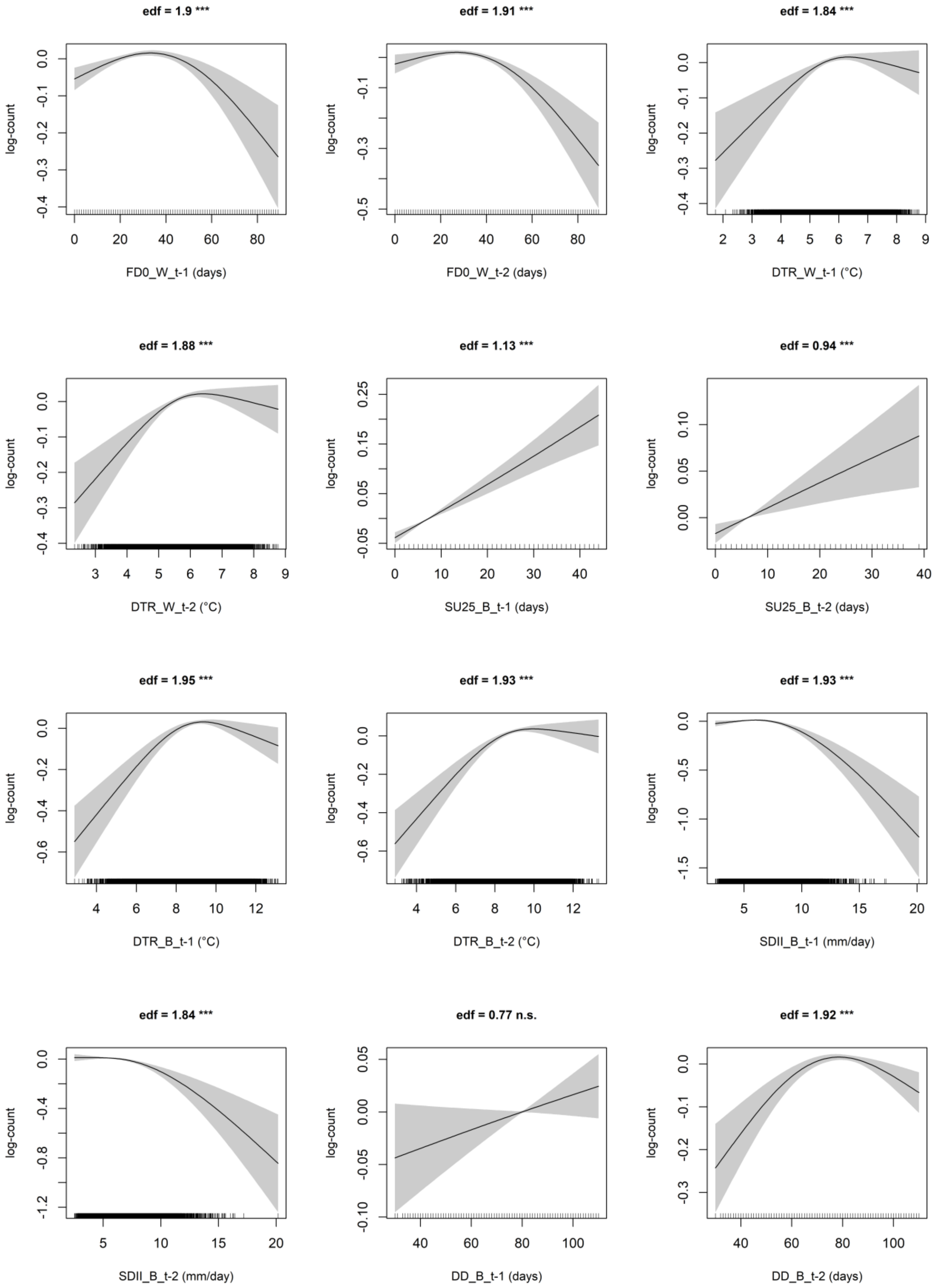

Eurasian Nuthatch *Sitta europaea*

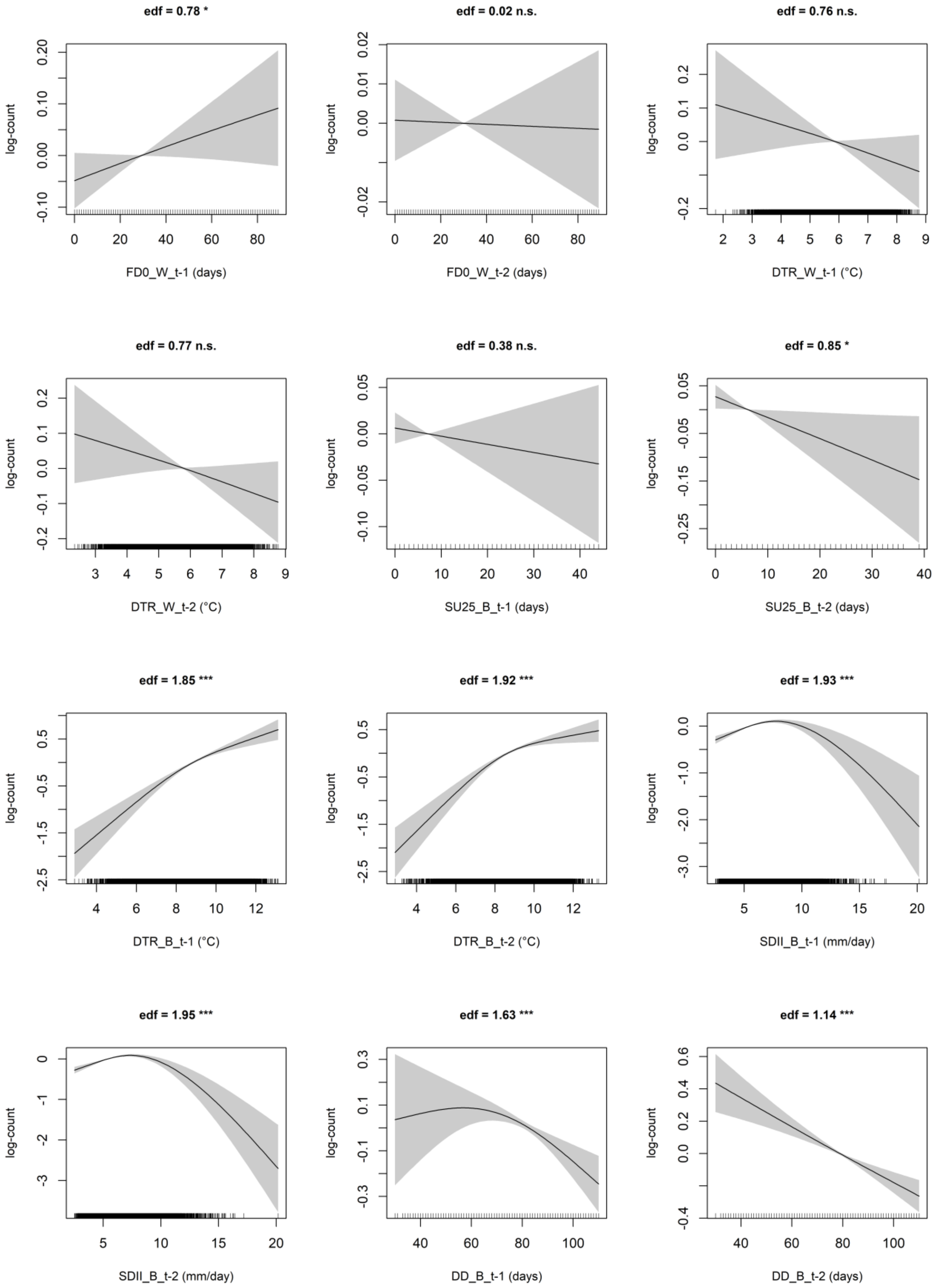

Eurasian Treecreeper *Certhia familiaris*

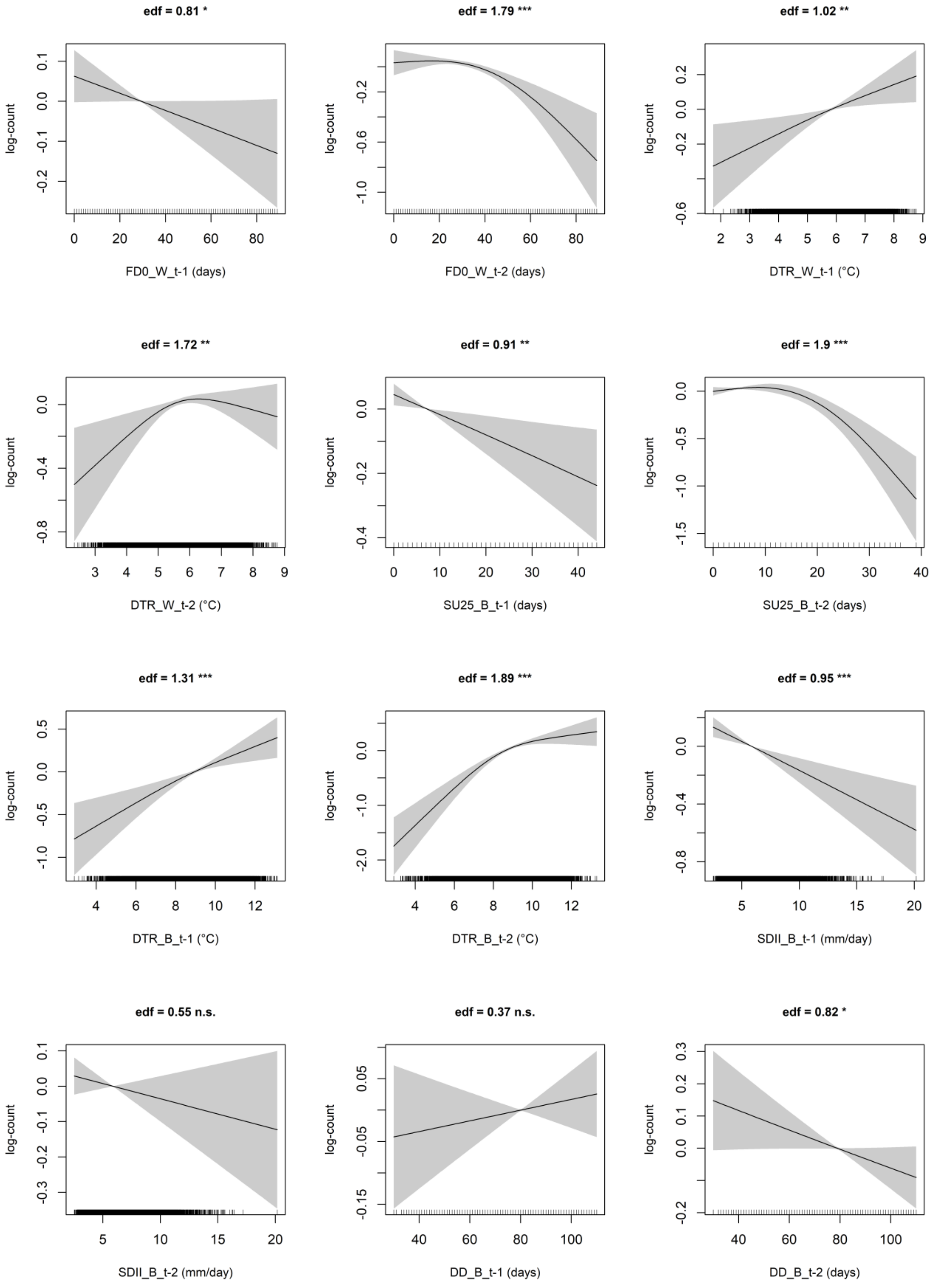

Eurasian Jay *Garrulus glandarius*

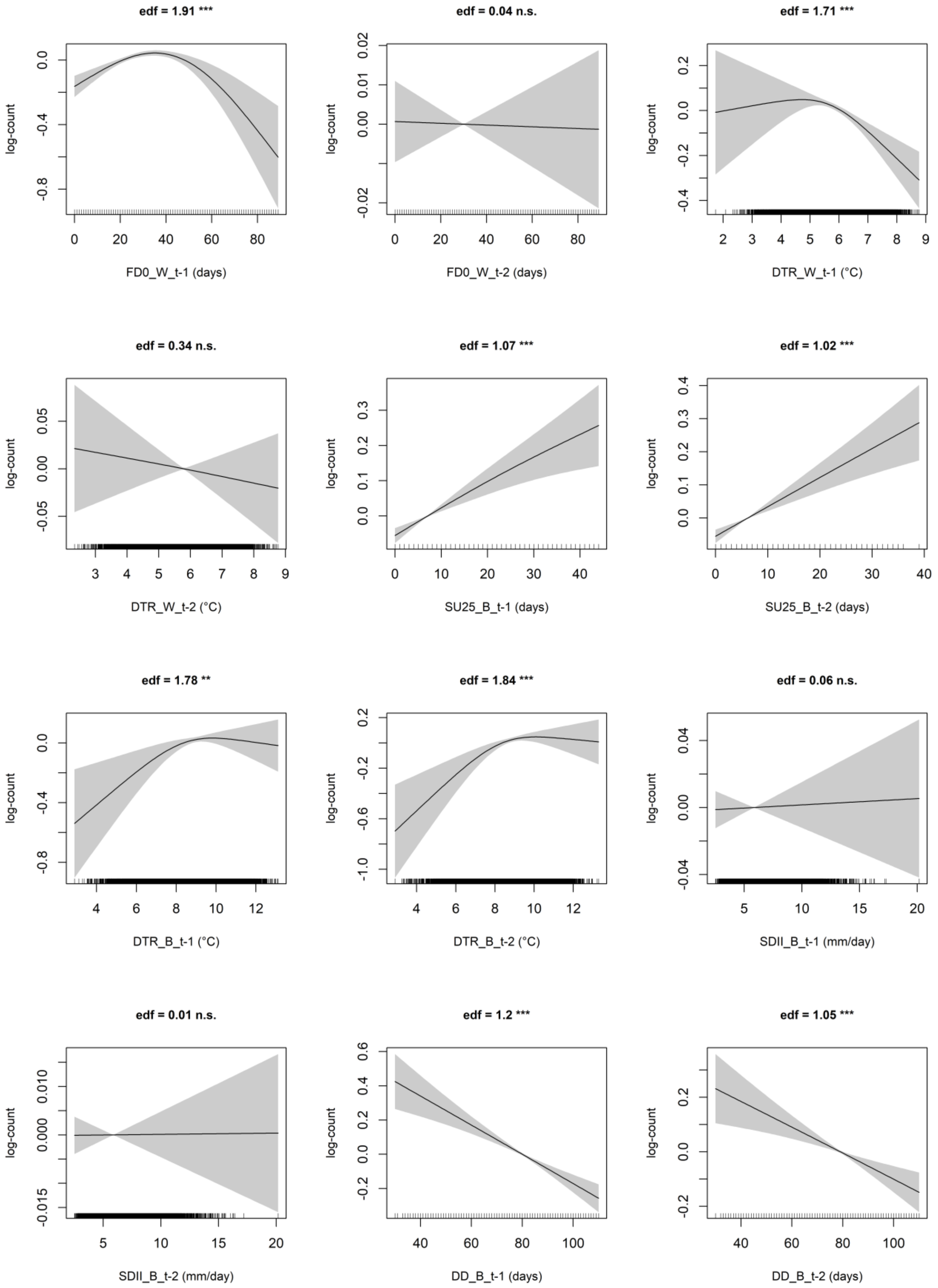

Eurasian Magpie *Pica pica*

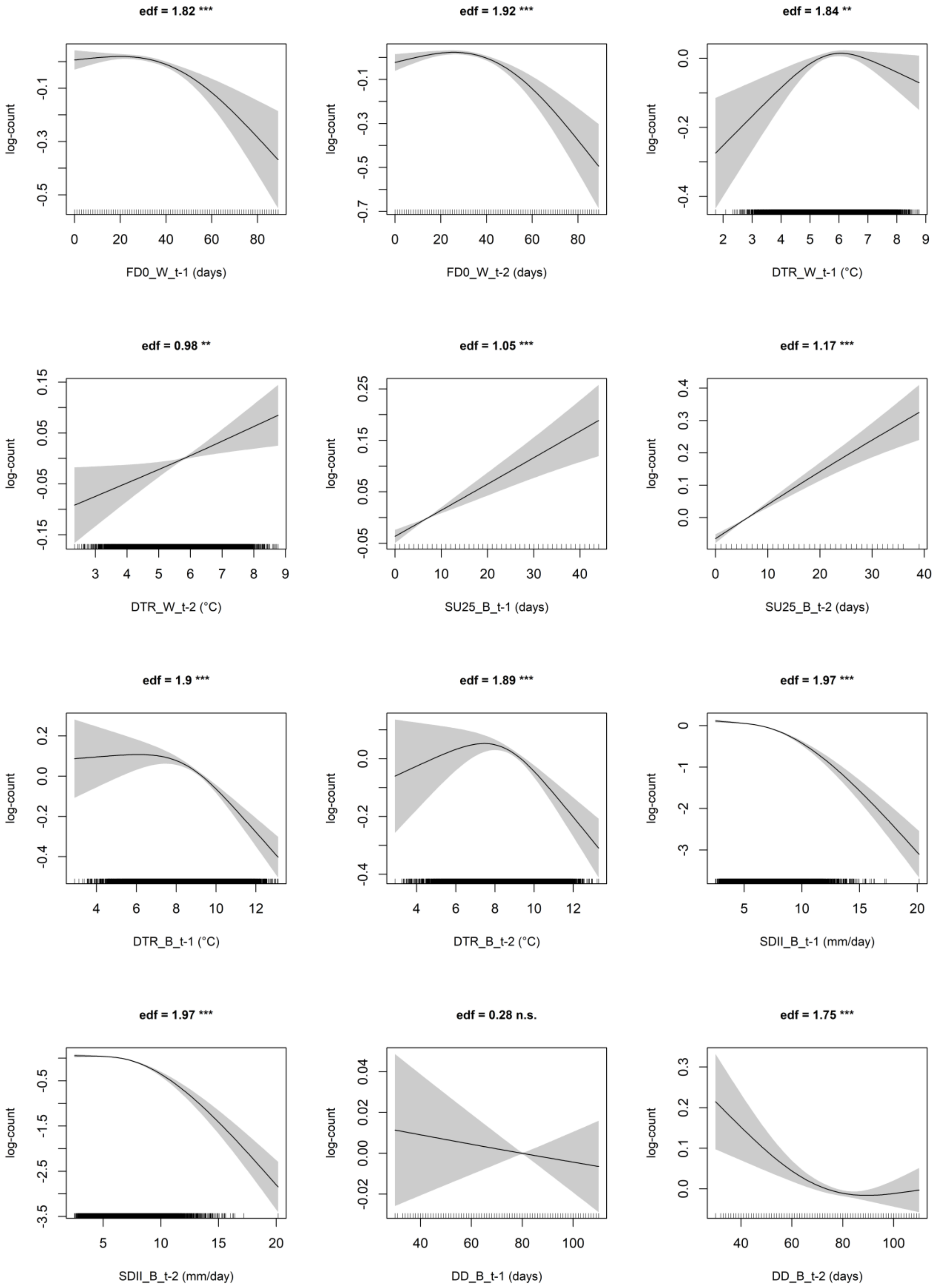

Western Jackdaw *Coloeus monedula*

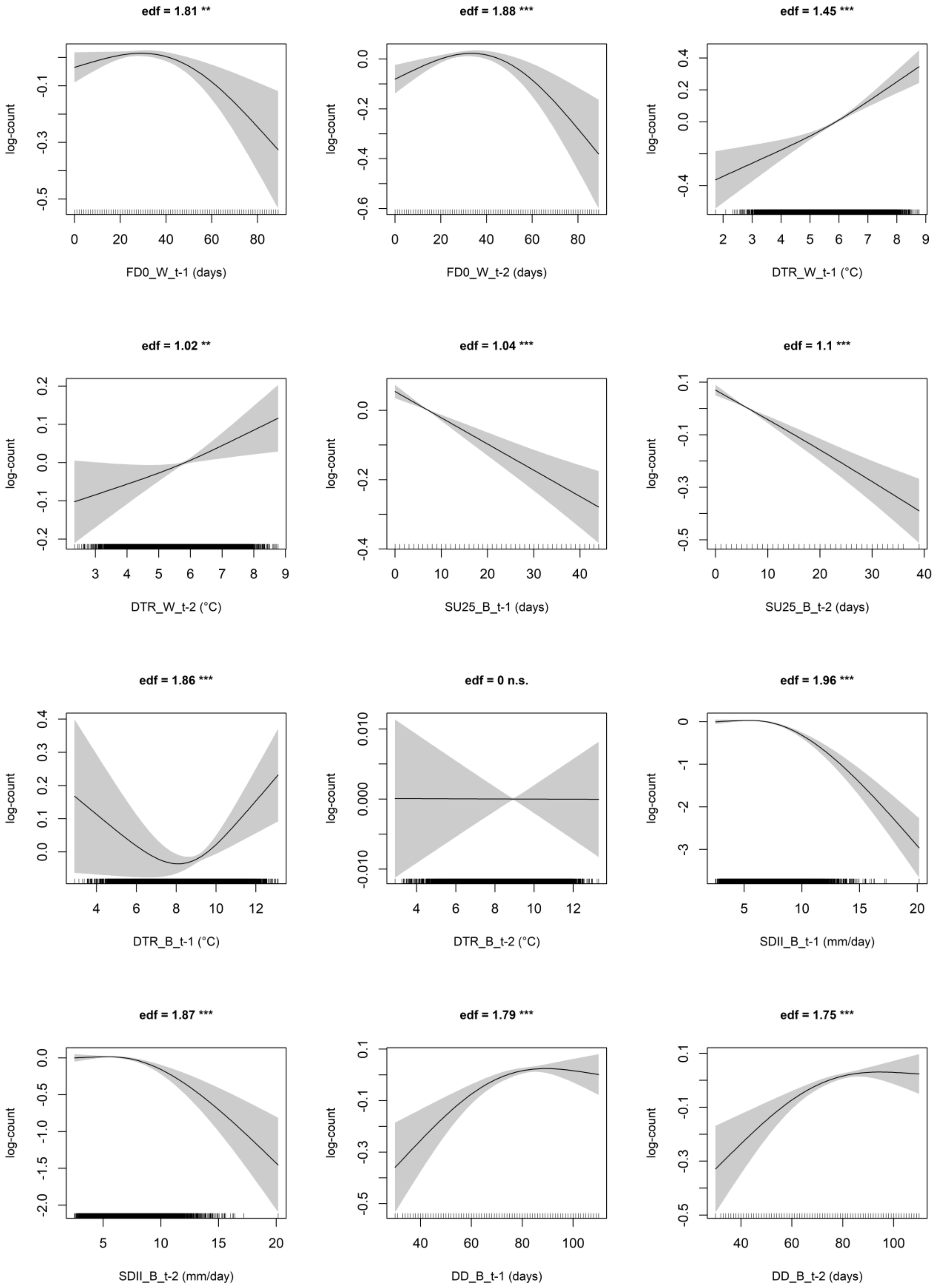

Rook *Corvus frugilegus*

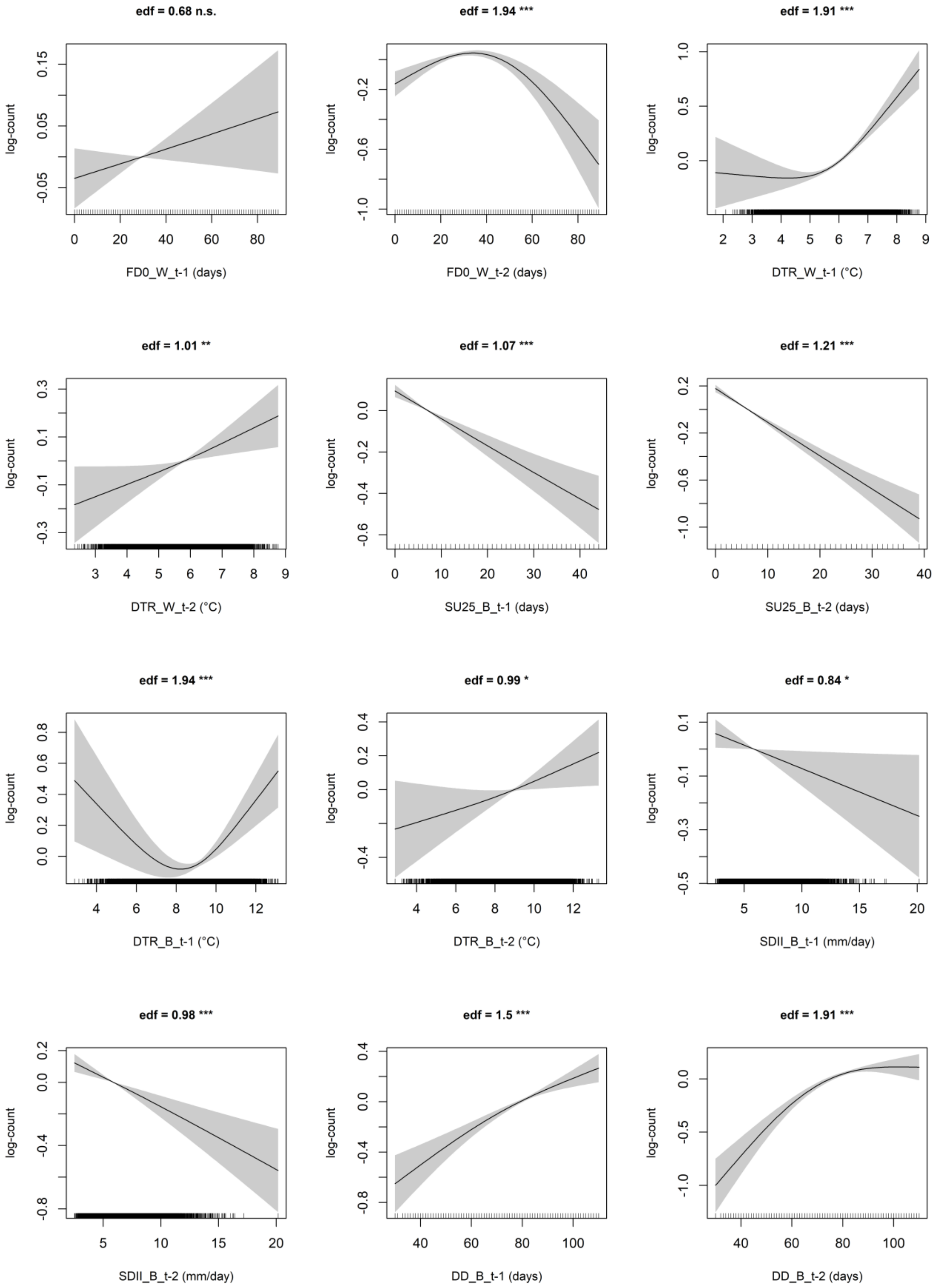

Carrion Crow *Corvus corone*

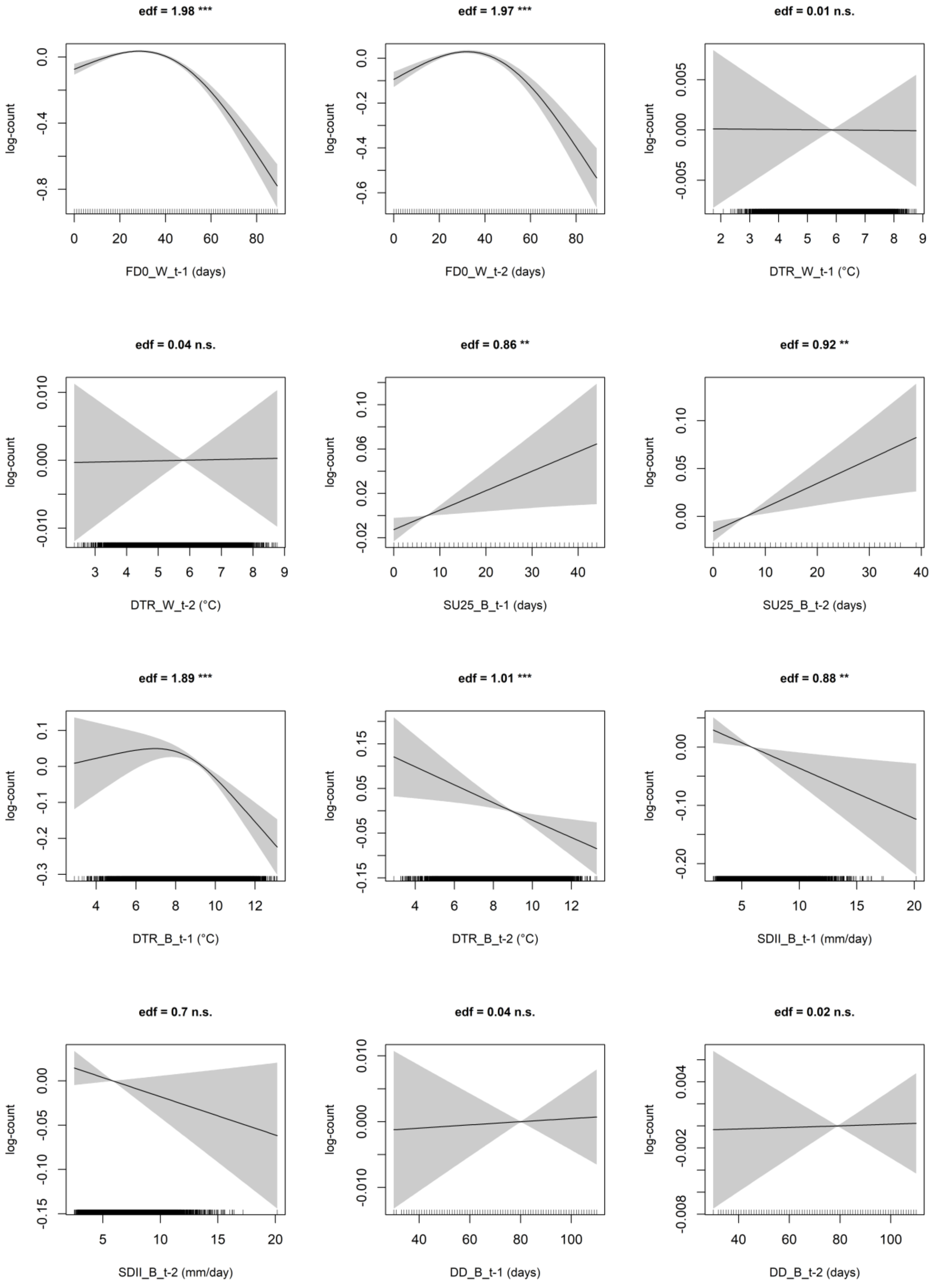

Hooded Crow *Corvus cornix*

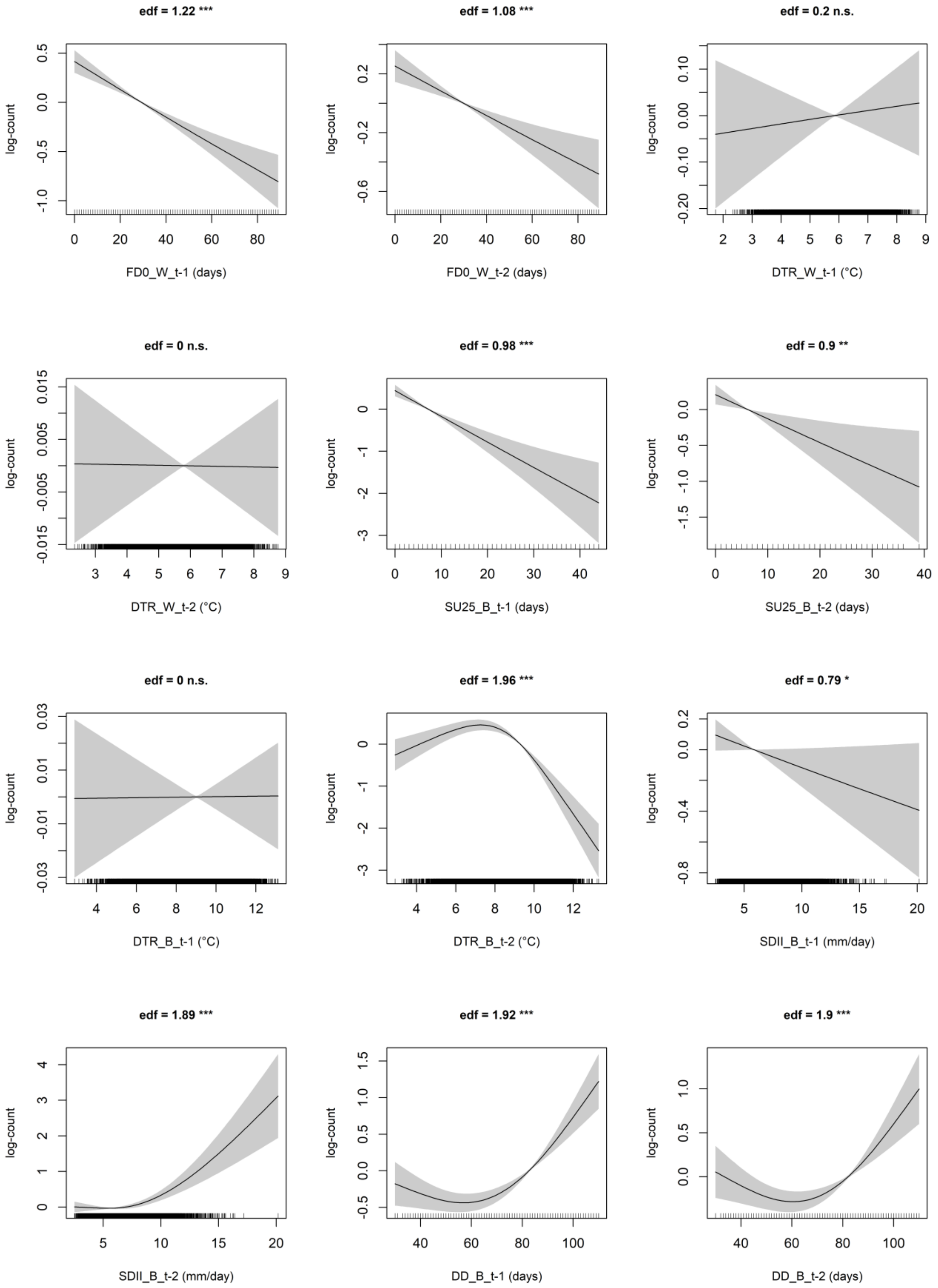

Northern Raven *Corvus corax*

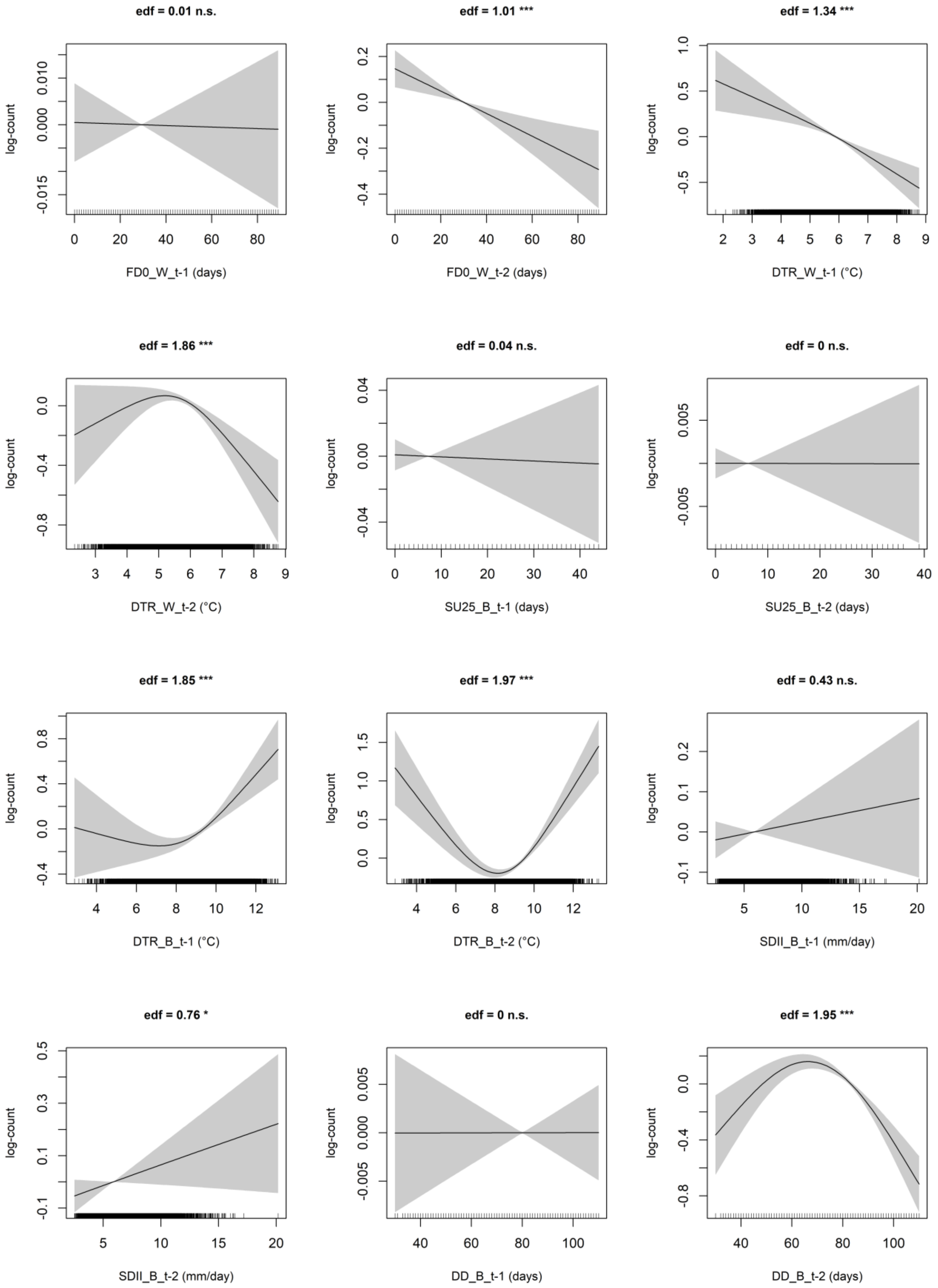

Common Starling *Sturnus vulgaris*

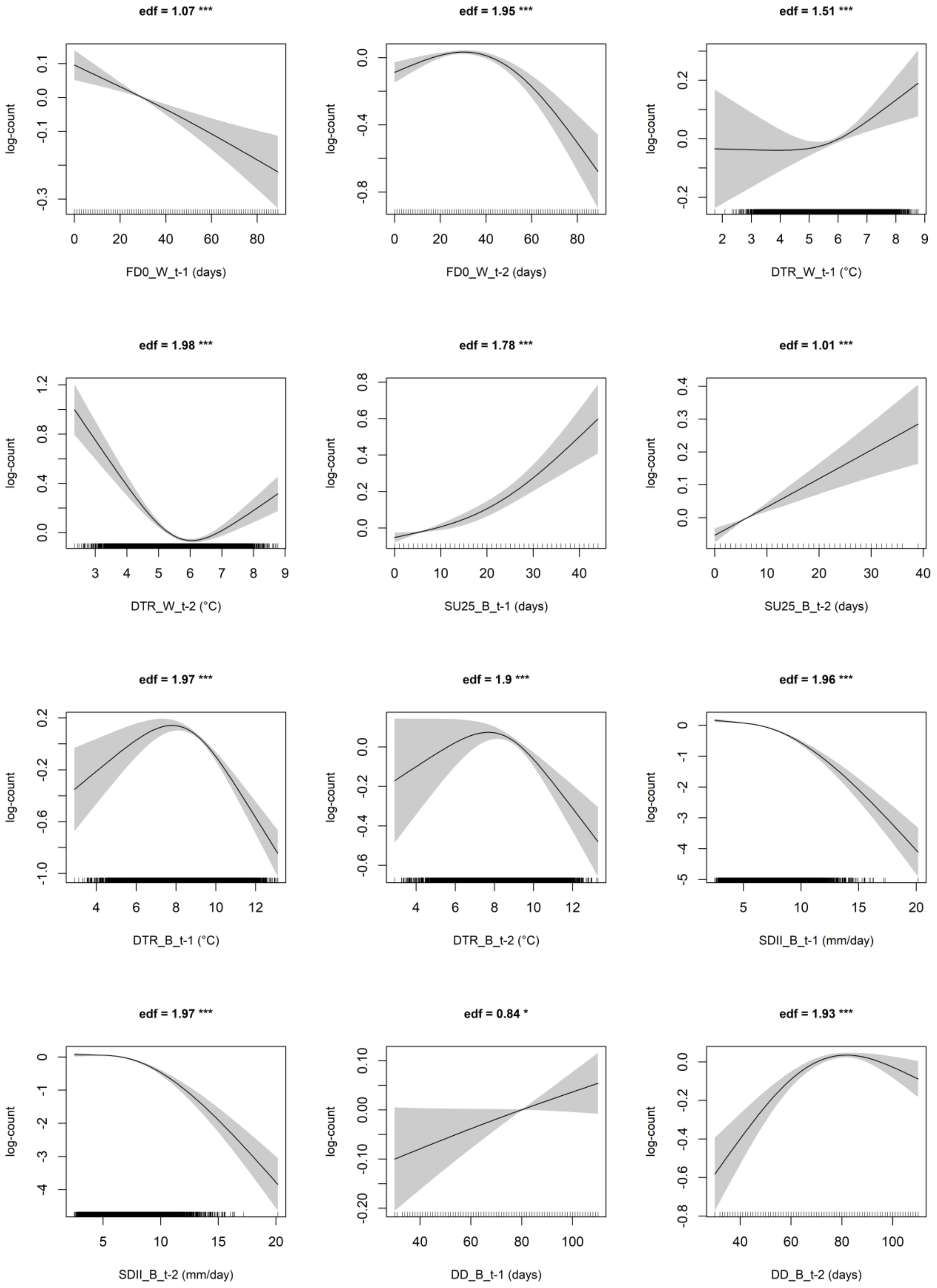

House Sparrow *Passer domesticus*

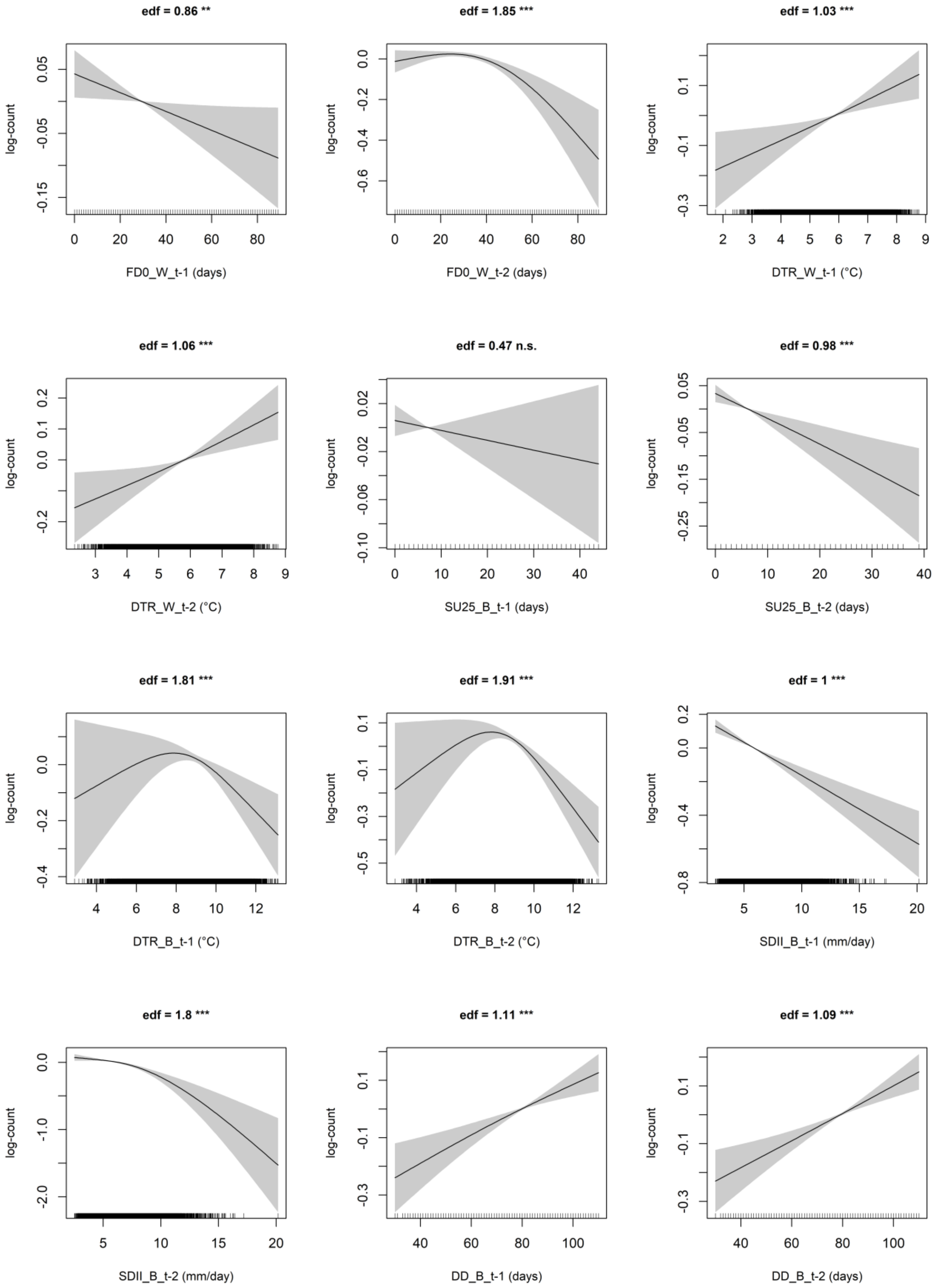

Eurasian Tree Sparrow *Passer montanus*

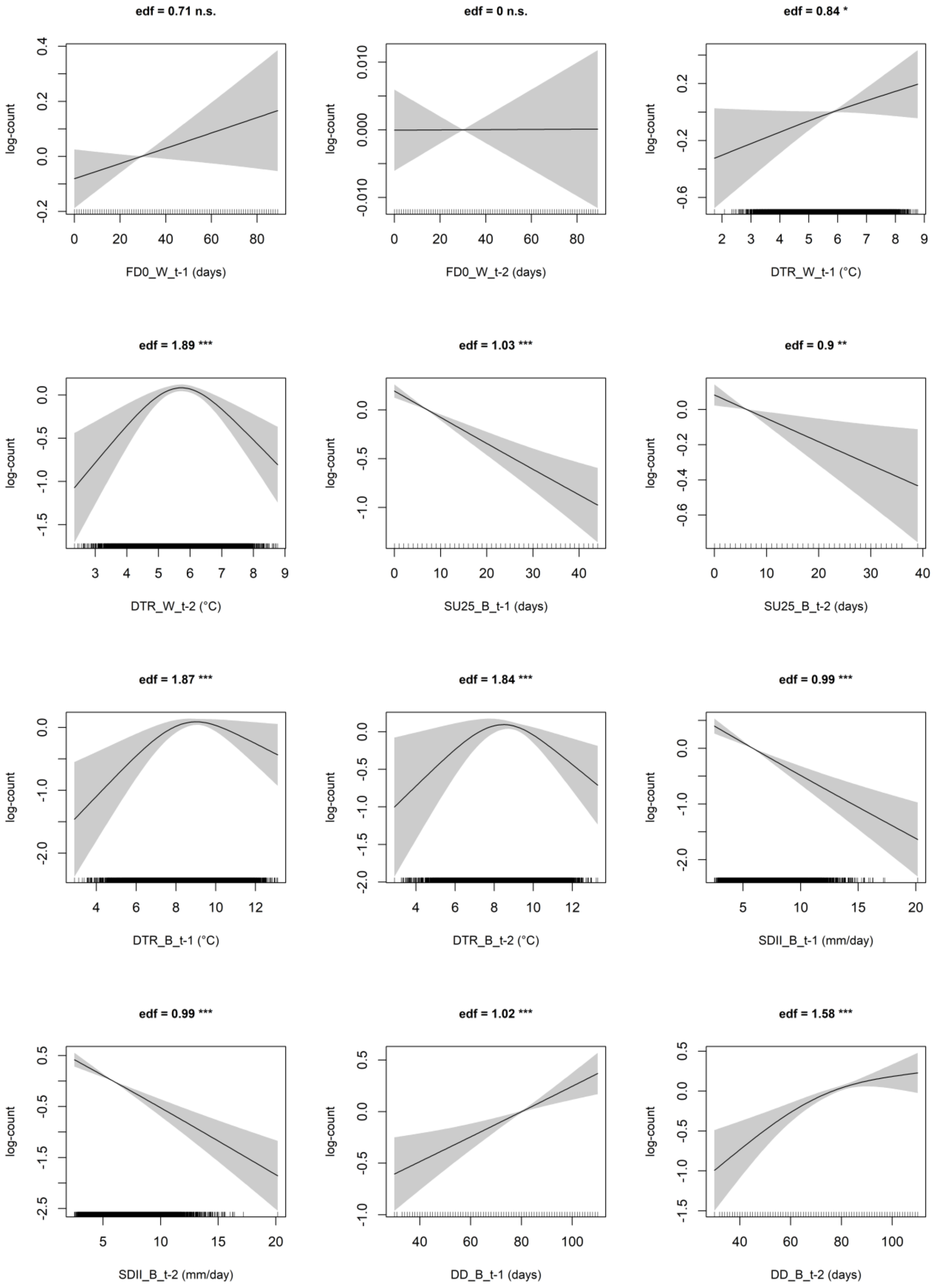

Common Chaffinch *Fringilla coelebs*

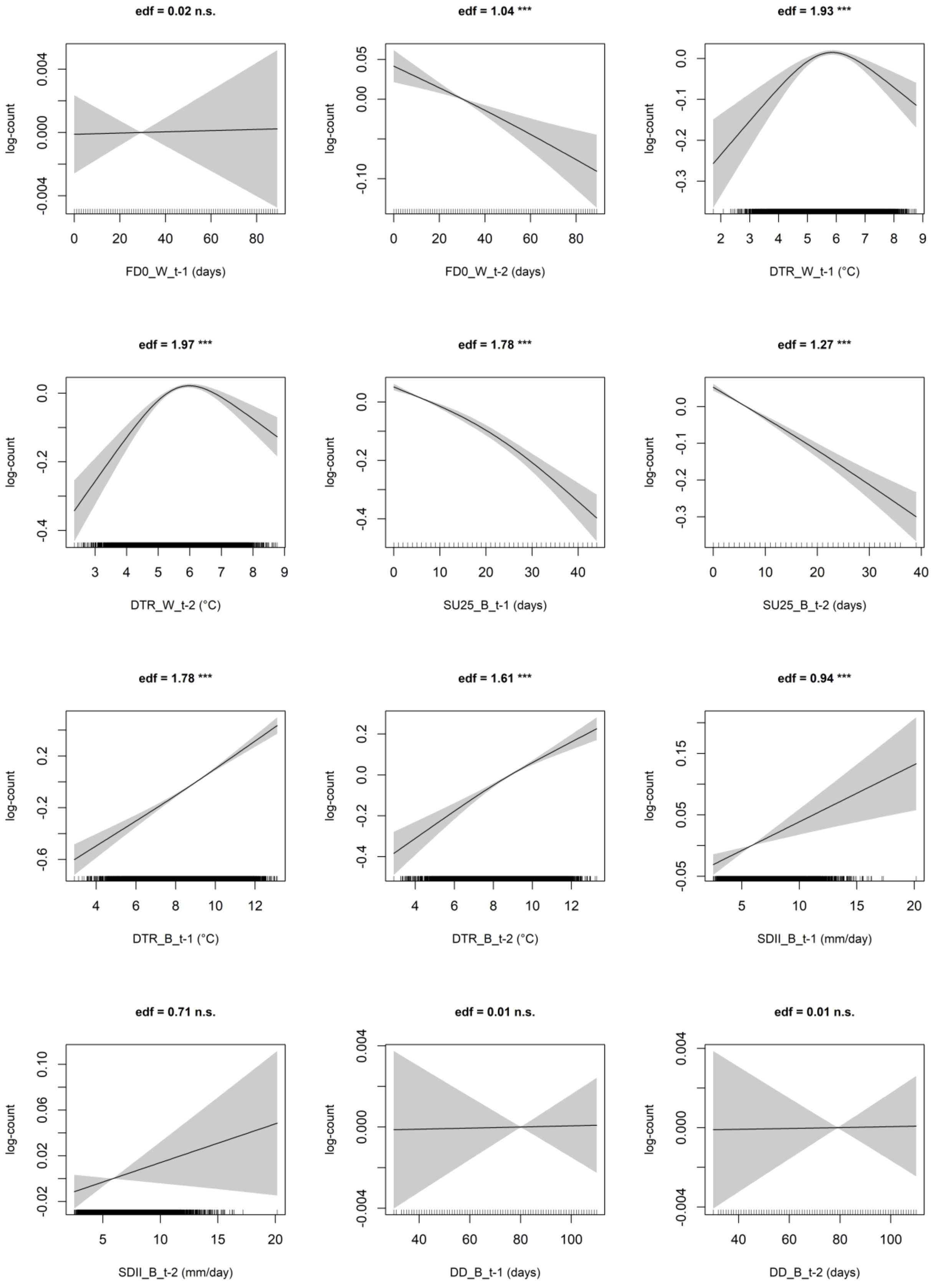

European Greenfinch *Chloris chloris*

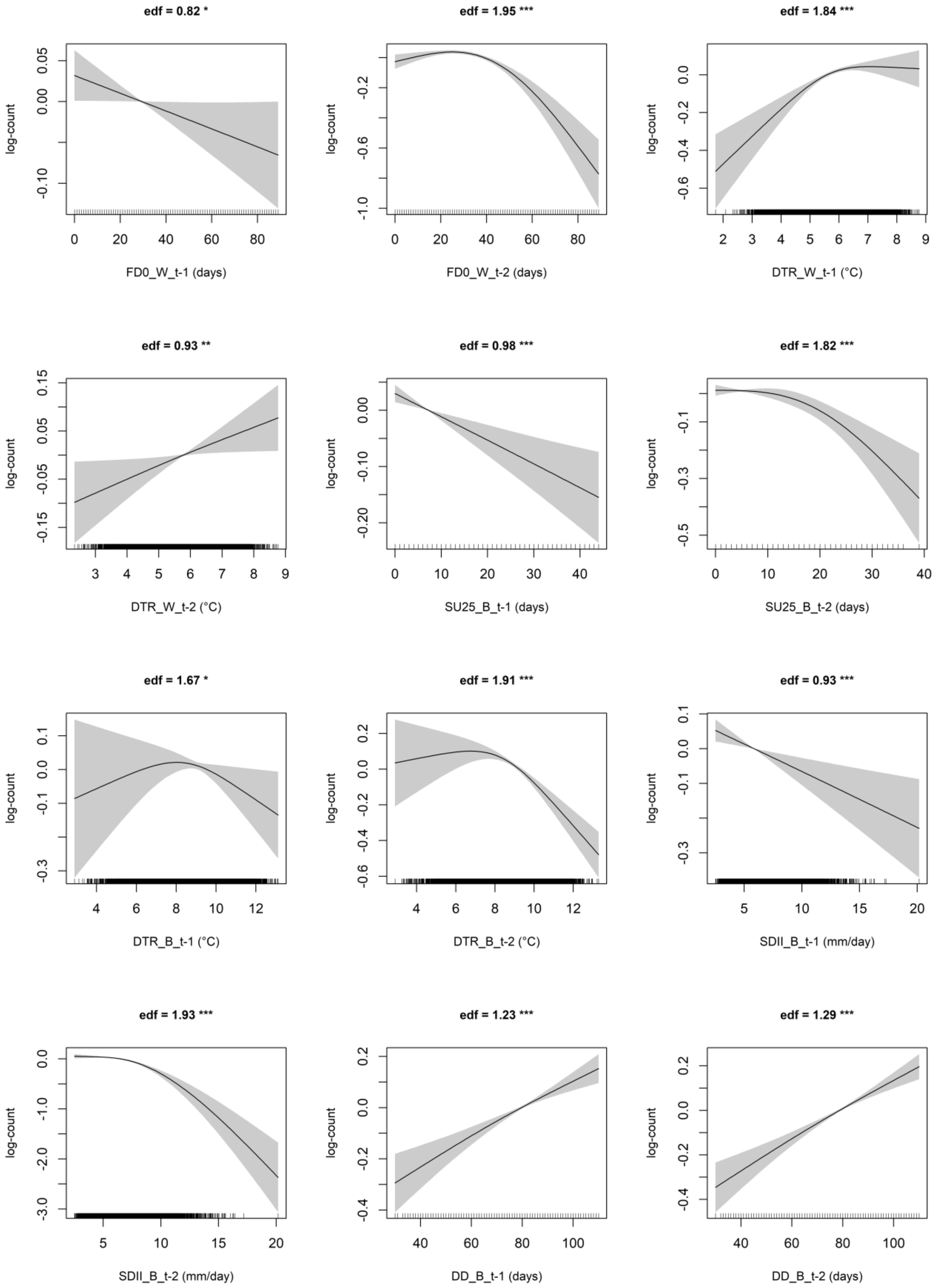

European Goldfinch *Carduelis carduelis*

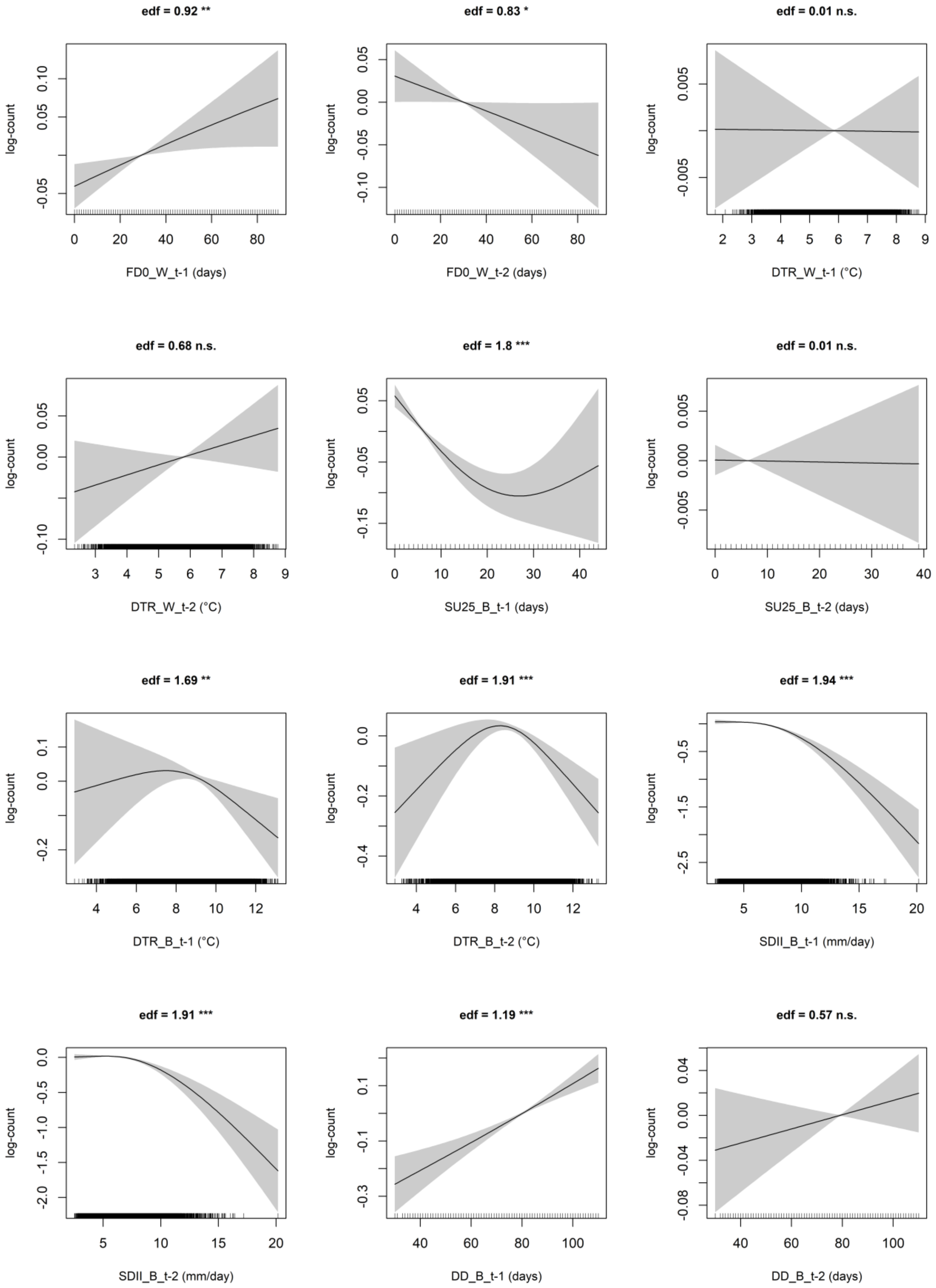

Eurasian Siskin *Spinus spinus*

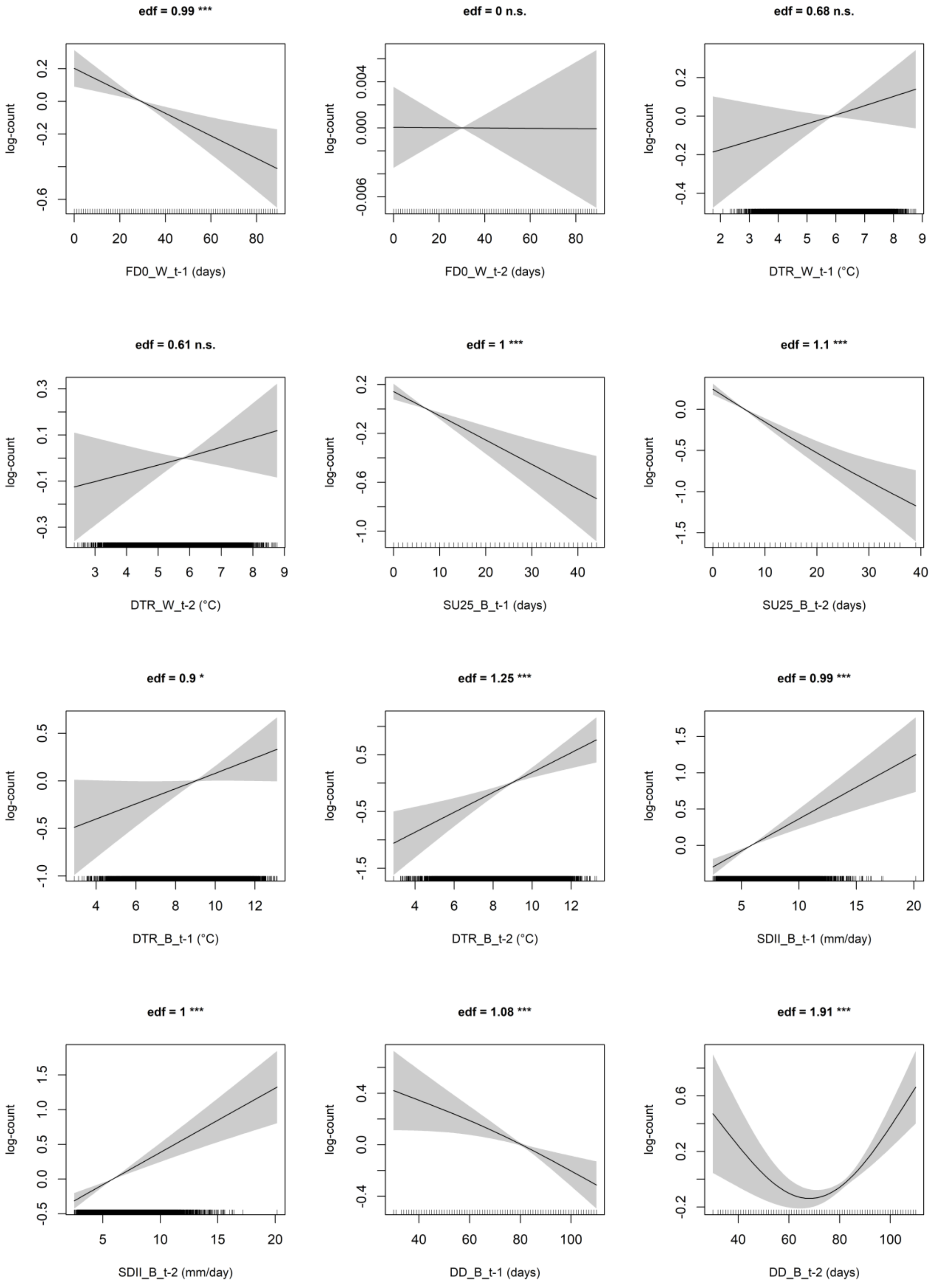

Common Linnet *Linaria cannabina*

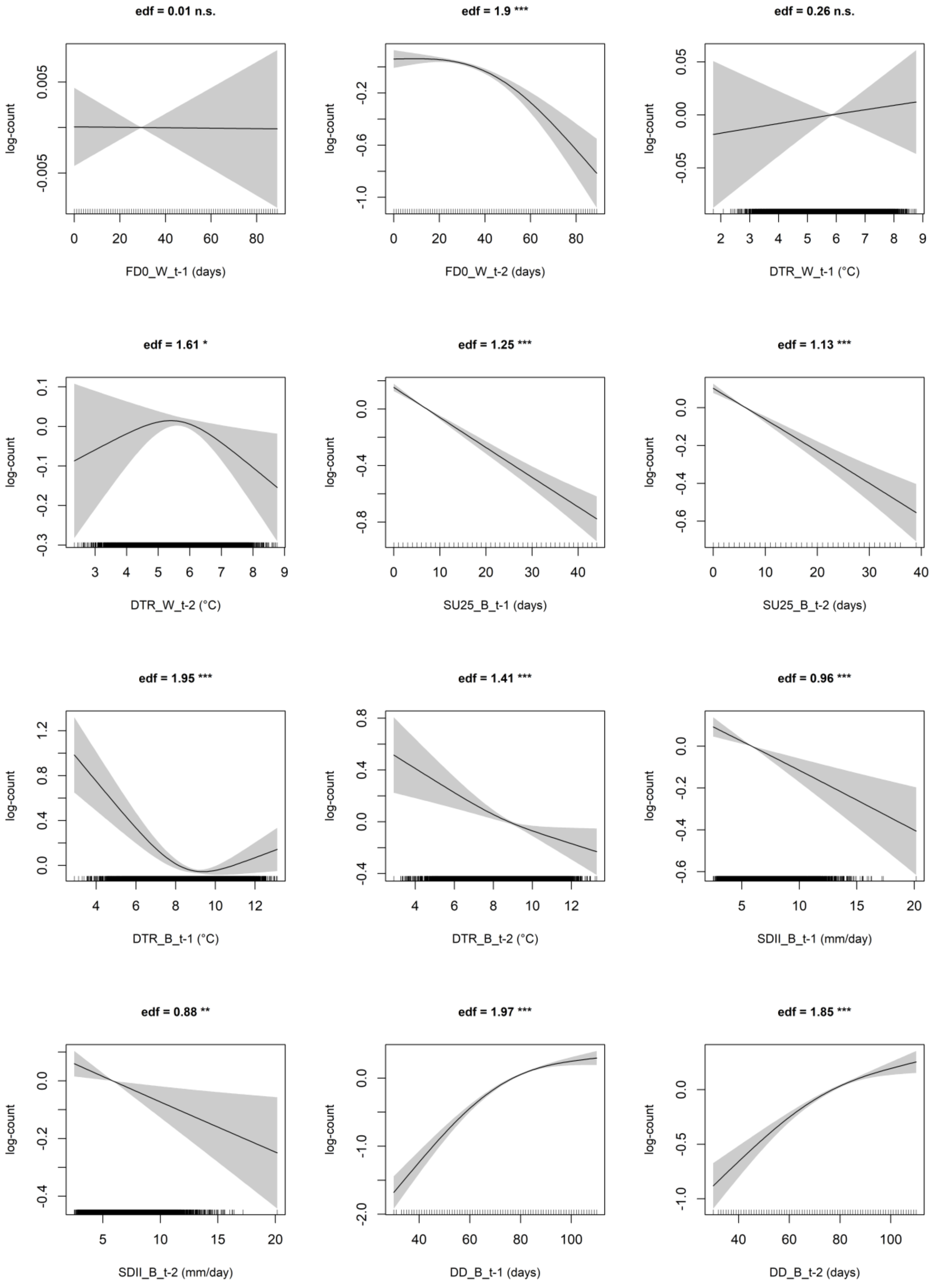

Twite *Linaria flavirostris*

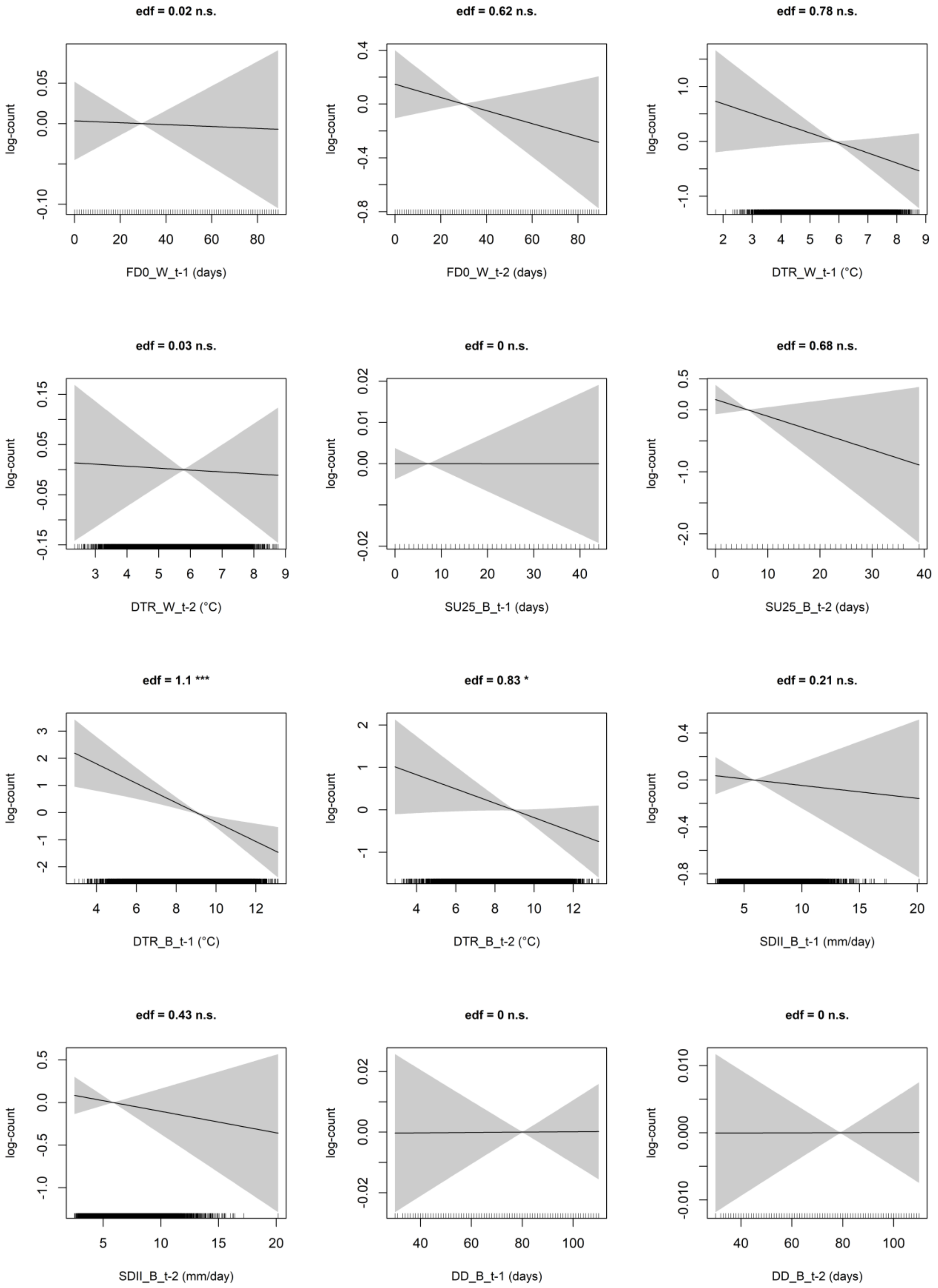

Lesser Redpoll *Acanthis cabaret*

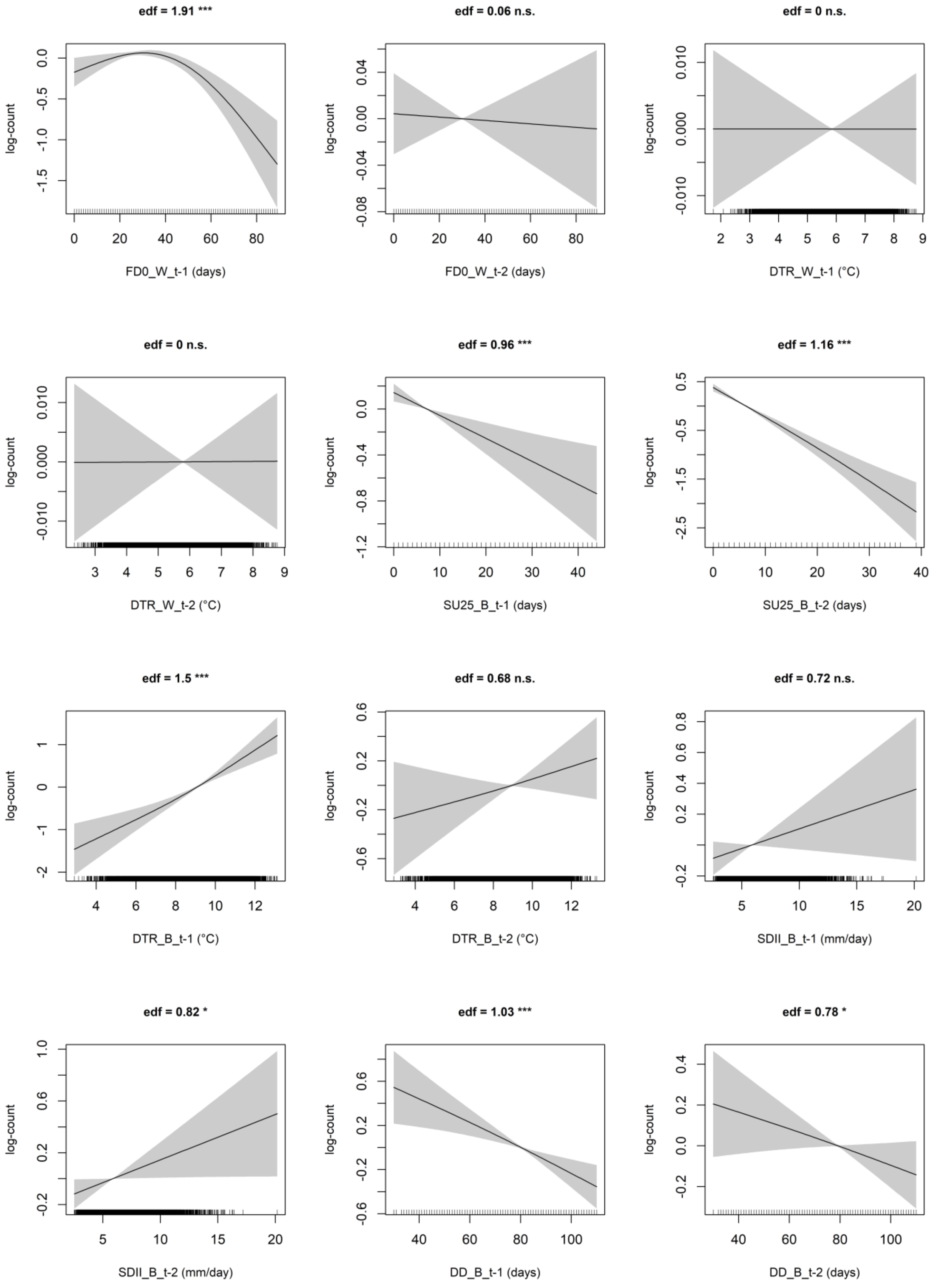

Red Crossbill *Loxia curvirostra*

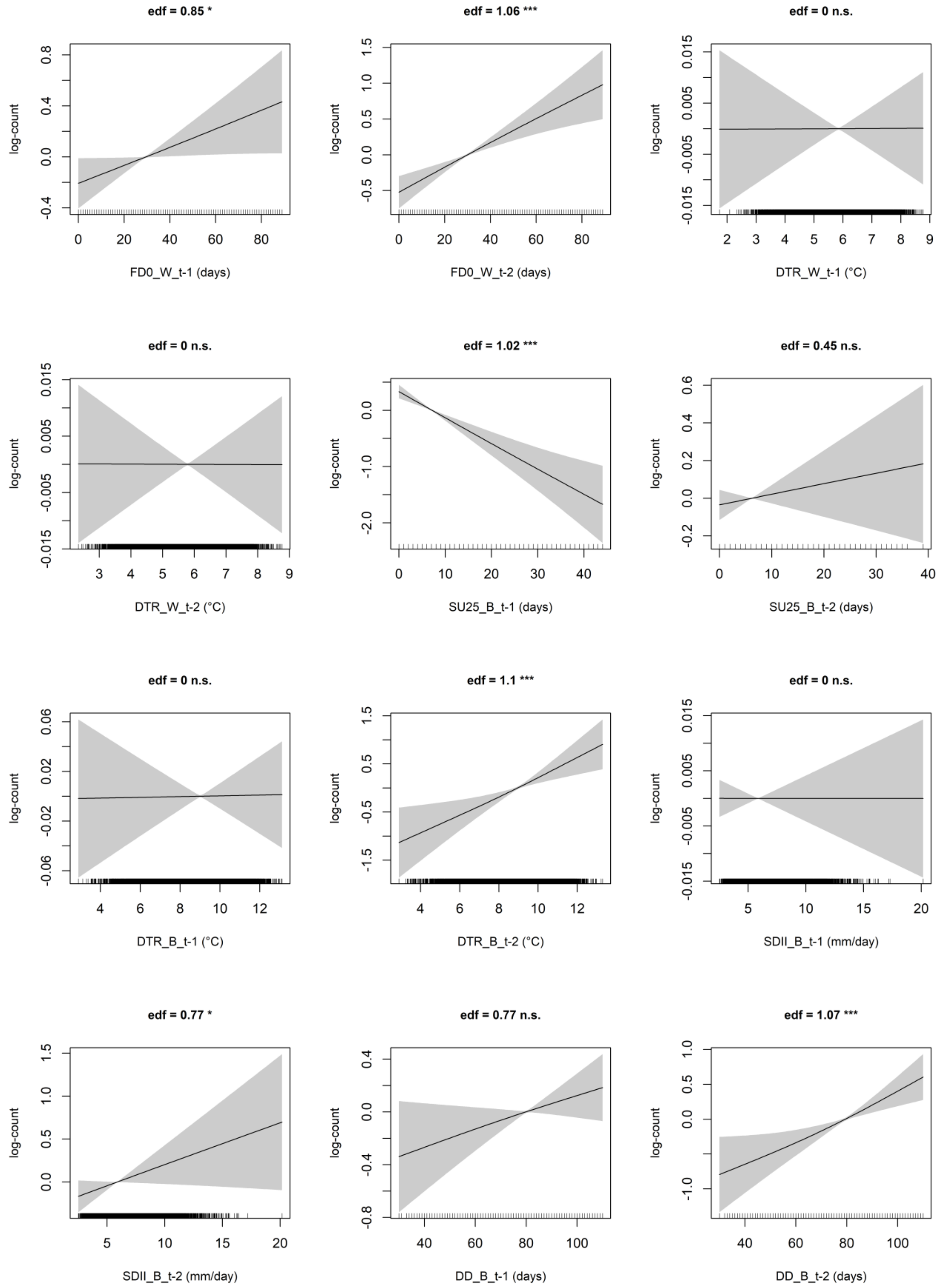

Eurasian Bullfinch *Pyrrhula pyrrhula*

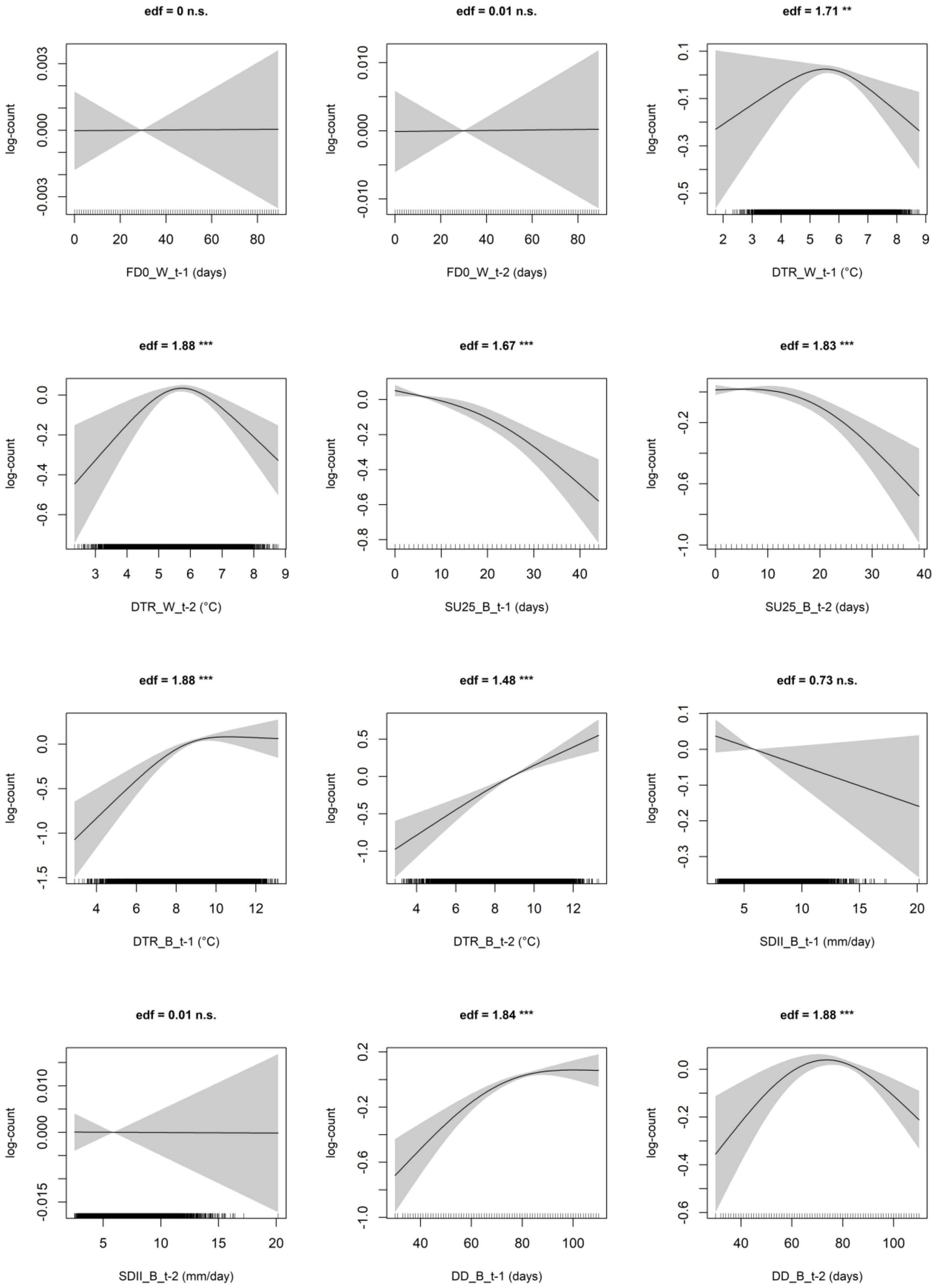

Yellowhammer *Emberiza citronella*

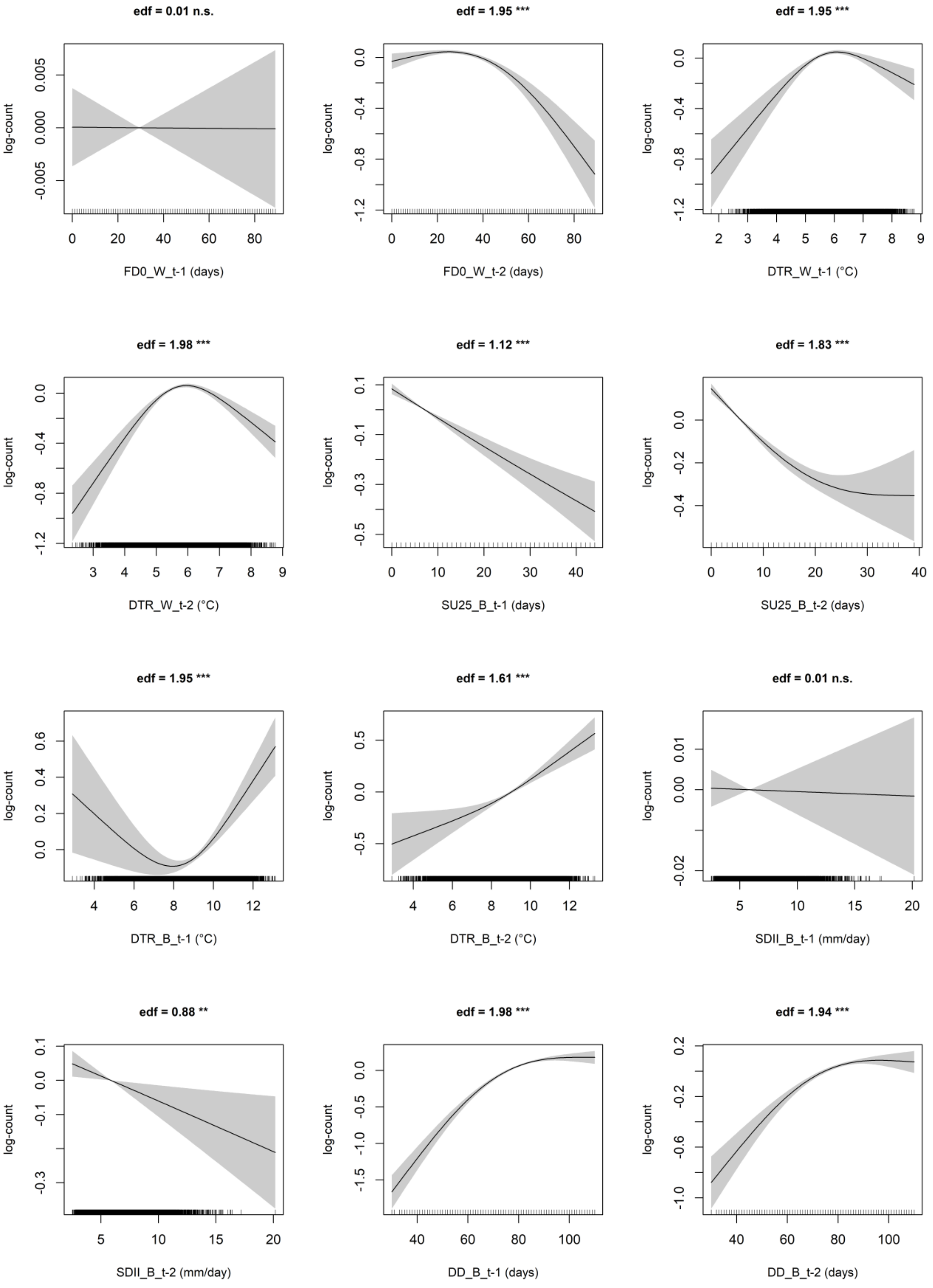

Common Reed Bunting *Emberiza schoeniclus*

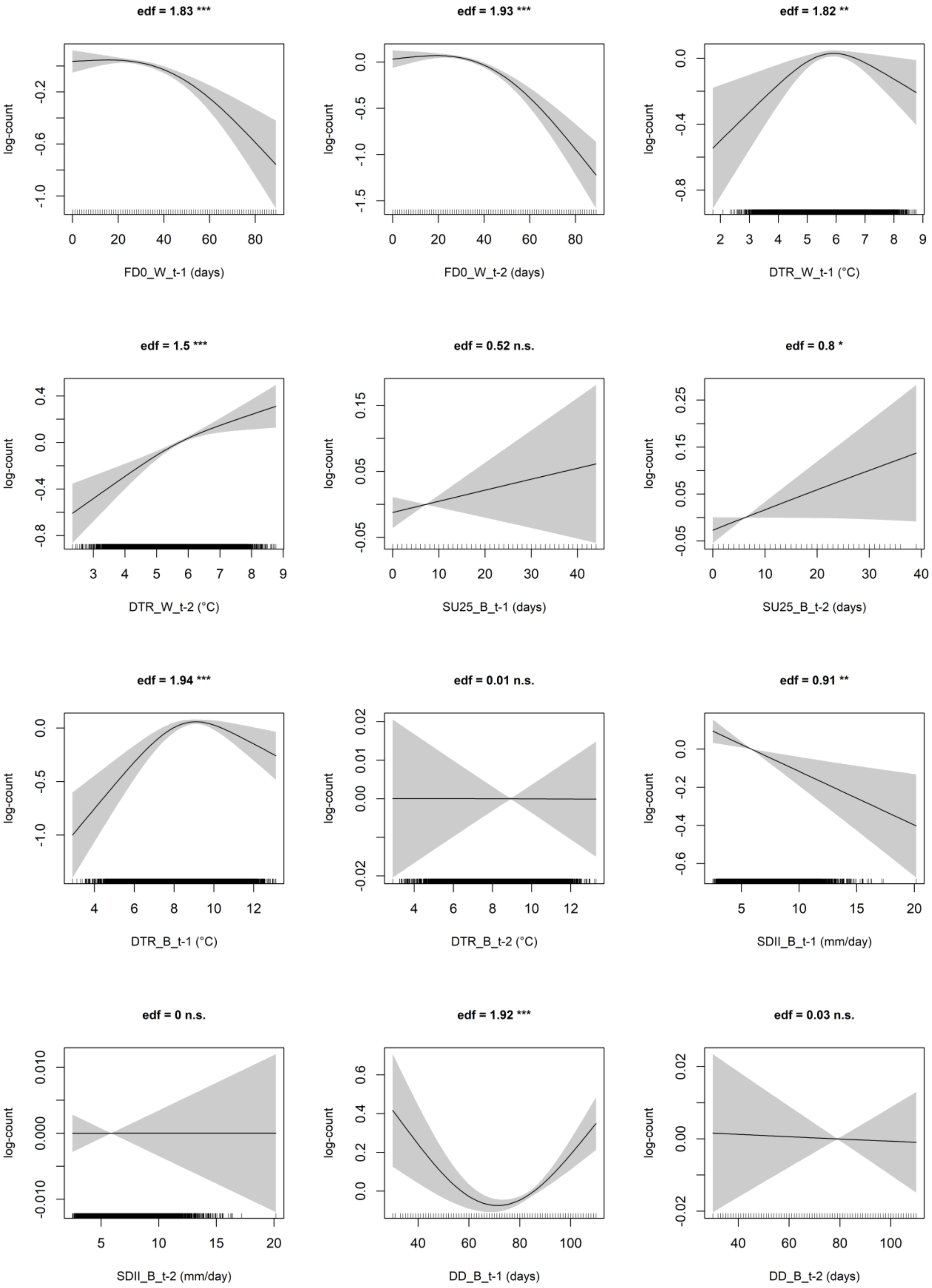

Corn Bunting *Emberiza calandra*

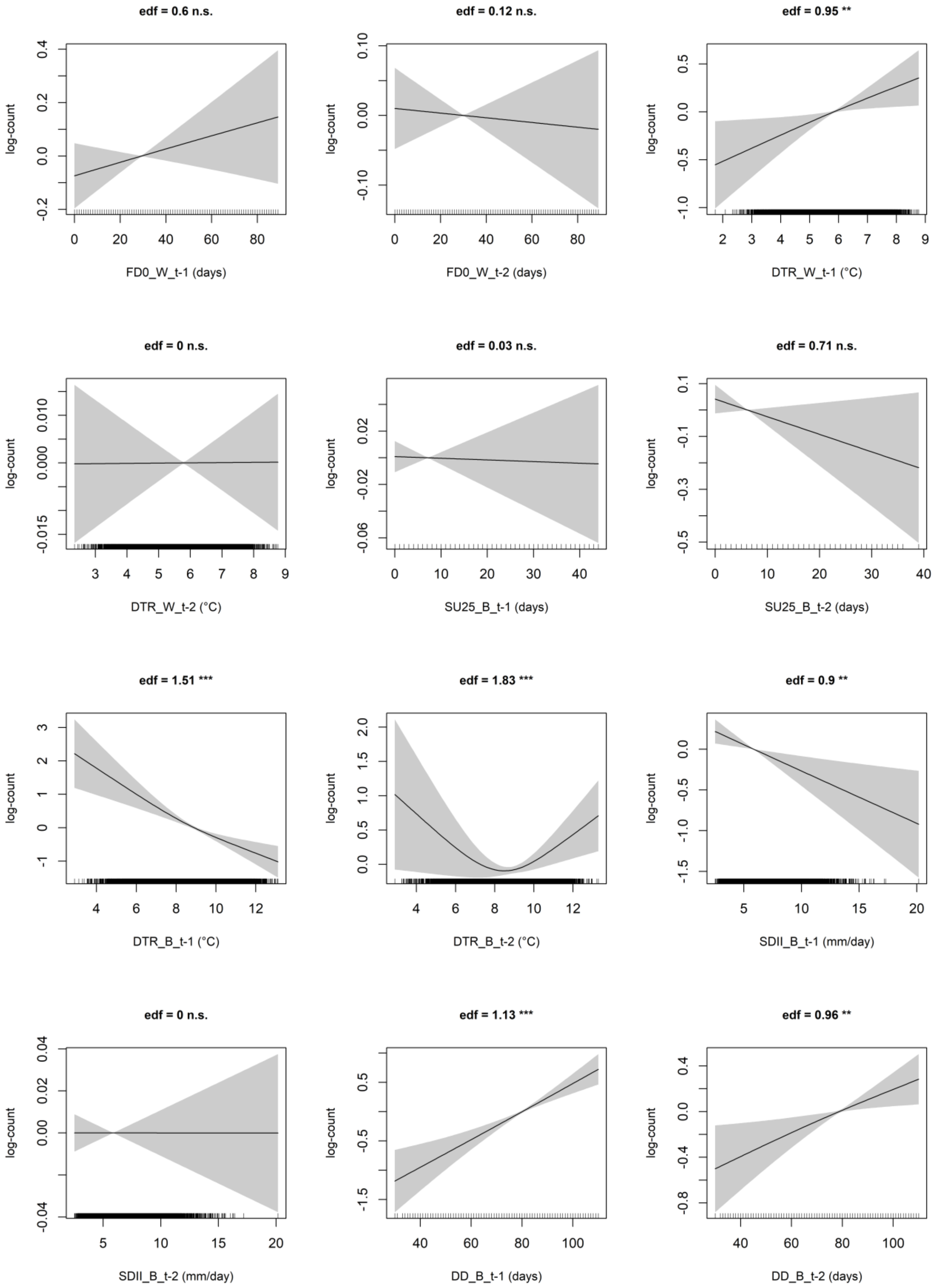

Indian Peafowl *Pavo cristatus*

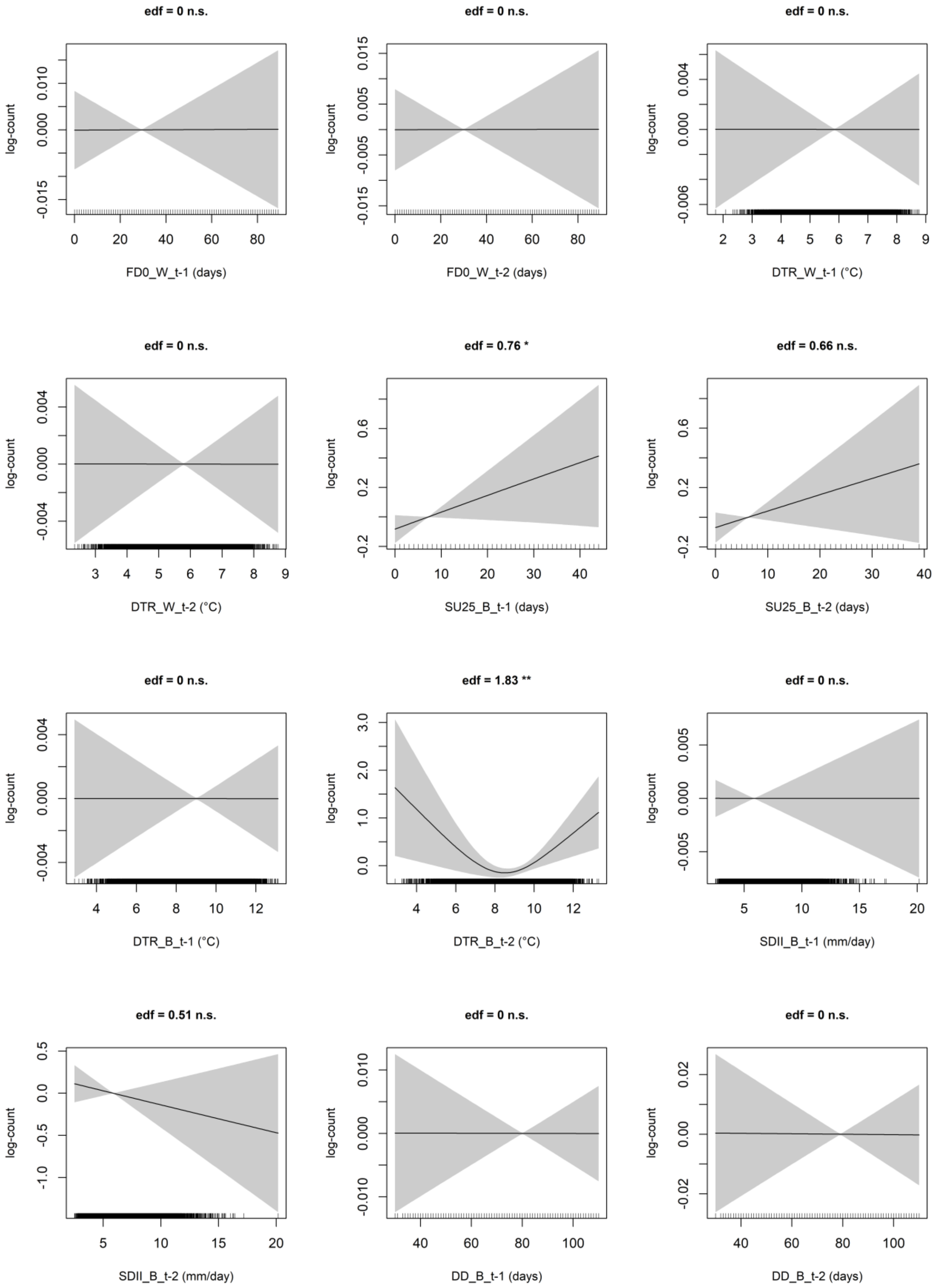

Supplement: Supplementary file 1 — Supplementary file1 (PDF 21894 KB) [file 442_2023_5504_MOESM1_ESM.pdf]
